# Facile Assembly of Structurally Diverse 2*H*-Pyrans Enabled by Chloropalladation-Initiated Carboetherification of Alkenes

**DOI:** 10.3390/molecules31111778

**Published:** 2026-05-22

**Authors:** Fanghua Mao, Bowen Wang, Zhengwang Chen, Yin-Long Lai, Huanfeng Jiang, Jianxiao Li

**Affiliations:** 1 Key Laboratory of Functional Molecular Engineering of Guangdong Province, School of Chemistry and Chemical Engineering, South China University of Technology, Guangzhou 510640, China; 19807394690@163.com (F.M.); wbw430602@163.com (B.W.); jianghf@scut.edu.cn (H.J.); 2 Jiangxi Provincial Key Laboratory of Synthetic Pharmaceutical Chemistry, Gannan Normal University, Ganzhou 341000, China; 3 College of Chemistry and Civil Engineering, Shaoguan University, Shaoguan 512005, China

**Keywords:** alkyne, alkene, palladium catalysis, cyclization reaction, heterocyclic compound

## Abstract

3,6-Dihydro-2*H*-pyran heterocyclic framework is one of the currently developed heterocyclic building blocks in both pharmaceutical chemistry and organic synthesis, but with significant challenges. To overcome these challenges, herein, we report a robust synthetic methodology of palladium-catalyzed carboetherification of alkenes with alkynols for accessing polyfunctionalized 3,6-dihydro-2*H*-pyrans under aerobic oxidative conditions. In particular, this synthetic approach features excellent functional group compatibility, mild reaction conditions, and good step- and atom-economy. Additionally, an array of functional groups such as halogen group, ester, nitrile, aldehyde, phenoxy, and aromatic heterocycles were nicely tolerated, affording the synthetically challenging 2*H*-pyran derivatives in moderate-to-good yields. Notably, the practicability of this protocol is further verified by gram-scale synthesis and the late-stage diversification of pharmaceuticals and biologically active molecules.

## 1. Introduction

**Scheme 1:**
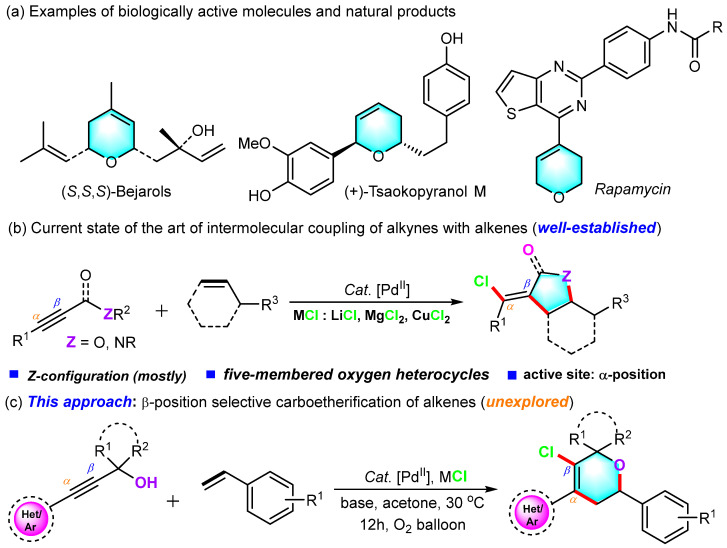
Research background of 2*H*-pyrans and our hypothesis.

The development of original and diversified synthetic strategy for the assembly of useful heterocyclic molecules in a convenient manner is fundamental for vibrant fields in both academic and industrial provinces [1,2,3,4,5]. Among the family of six-membered oxygen heterocycles, 3,6-dihydro-2*H*-pyrans are attractive structural scaffolds owing to their significant biologically active and potential for becoming pharmaceuticals (Scheme 1a) [6]. For instance, the natural product (*S*, *S*, *S*)-Bejarols is the essential oil composition of Achillea clusiana [7]. Of particular significance is the (+)-Tsaokopyranol M, which has been recognized as the α-glucosidase inhibitor for the treatment of diabetes [8]. Rapamycin is a MTOR inhibitor, which has the potential for treating Alzheimer disease [9]. As a result, considerable achievements have been exploited for assembling structurally diverse 3,6-dihydro-2*H*-pyrans in the past few years. Generically, ring-closing metathesis reaction [10,11], Prins-type cyclization reaction [12,13], hetero-Diels–Alder reaction [14,15,16], and transition-metal-free cyclization reaction [17,18,19] have emerged as the efficient synthetic protocols. Moreover, transition metal-catalyzed tandem cyclization approaches have also been well-established for the preparation of synthetically challenging 2*H*-pyran motifs [20,21,22,23]. Despite these advances, some of these approaches suffer from certain limitations, such as multistep synthetic procedures, prefunctionalized substrates, and/or complicated operation and handling. As a consequence, the development of an expeditious and practical catalytic approach for the preparation of structurally diverse 2*H*-pyrans is still highly desirable.

Notably, palladium-catalyzed tandem annulation approaches of unsaturated hydrocarbon have emerged as the guiding tactic for the construction of more elaborated poly- and heterocyclic architectures with high atom and step economy [24,25,26,27]. In this regard, the group of Bäckvall [28,29,30,31], Werz [32,33,34,35], Zhu [36,37,38], Eycken [39,40,41], and Liang [42,43] independently described many elegant palladium-catalyzed regioselective cascade annulation of alkenes, alkynes, and allenes to assembly of bioactive compounds and functional molecules. On the basis of their easy accessibility and distinctive bifunctional nature, functionalized alkynes, such as propargylic alcohols and propargylic amines as the important synthons, play an exceptionally vital role in organic synthesis chemistry and pharmaceutical chemistry [44,45,46]. More importantly, among various nucleopalladation protocols, chloropalladation-initiated cascade cyclization/ functionalization reactions of propargylic compounds with alkenes are especially attractive because they always offered oxygen- and nitrogen-containing heterocycle scaffolds in “one pot” manners. A wide array of aryl alkenes, acrylic derivatives, unactivated straight-chain alkenes, and cycloalkenes were adequately investigated to access five-membered *α*-methylene-*γ*-lactones [47,48,49,50], *γ*-lactams [51,52,53,54] and tetrahydrofuran derivatives [55,56,57,58] (Scheme 1b). However, to the best of our knowledge, there is no report for assembling structurally diverse 3,6-dihydro-2*H*-pyrans *via* chloropalladation-initiated cascade approach from propargylic alcohol derivatives with alkenes. Motivated by our longstanding research interests in palladium-catalyzed heterocyclic chemistry [59,60,61,62,63,64], we herein disclose a novel palladium-catalyzed intermolecular regioselective carboetherification of alkenes with alkynols for the construction of diversified 3,6-dihydro-2*H*-pyran derivatives (Scheme 1c).

## 2. Results and Discussion

In our preliminary experiments, 2-methyl-4-phenylbut-3-yn-2-ol (**1a**) and styrene (**2a**) were selected as the model substrates to screen these carboetherification conditions. After evaluating a variety of reaction parameters, the reaction of **1a** (0.1 mmol) with **2a** (0.15 mmol) in the presence of 10 mol% of Pd(CH_3_CN)_2_Cl_2_, 6 equiv. of CuCl_2_, 1.5 equiv. of LiCl, 60 mol% of DIPEA in acetone at 30 °C for 12 h, the desired product **3a** was detected in 78% GC yield along with 72% isolated yield (Table 1, entry 1). Additionally, we also studied several changes to the “standard conditions” by exploring the different parameters, including palladium catalysts, additives, bases, and solvents, to further understand the boundaries of this catalytic system. Initially, the use of the others palladium catalysts such as PdCl_2_, Pd(CH_3_CN)_2_(BF_4_)_2_, and Pd(PhCN)_2_Cl_2_ instead of Pd(CH_3_CN)_2_Cl_2_ resulted the desired product **3a** only in 41–56% yield (Table 1, entries 2–4). Further explorations indicated that both Pd(COD)_2_Cl_2_ and Pd(dppp)_2_Cl_2_ showed low efficiencies for this approach (Table 1, entry 5). Subsequently, different kinds of solvents such as CH_3_CN, HOAc, DMSO, and DMF were tested instead of acetone, and acetone proved to be the best choice for this transformation (Table 1, entries 6–7). Furthermore, DCE and THF also seemed to be less efficient compared with the acetone under the same catalytic conditions (Table 1, entry 8). Further examination of the bases revealed that DIPEA was the most suitable base, while the others organic and inorganic bases did not show apparent positive effects for this carboetherification reaction (Table 1, entries 9–10). Replacement of the LiCl by MgCl_2_ showed lower reactivity, and the desired product **3a** was detected in 54% GC yield (Table 1, entry 11). Reducing the additive LiCl loading to 1 equivalent could also efficiently promote this reaction, though the yield of **3a** decreased to some extent (Table 1, entry 12). When the reaction was conducted at 40 °C, the yield of **3a** slightly decreased (Table 1, entry 13). Control experiments unequivocally confirmed that both Pd(CH_3_CN)_2_Cl_2_, and CuCl_2_ are essential for this cascade carboetherification reaction (Table 1, entries 14–15).

**Figure 1:**
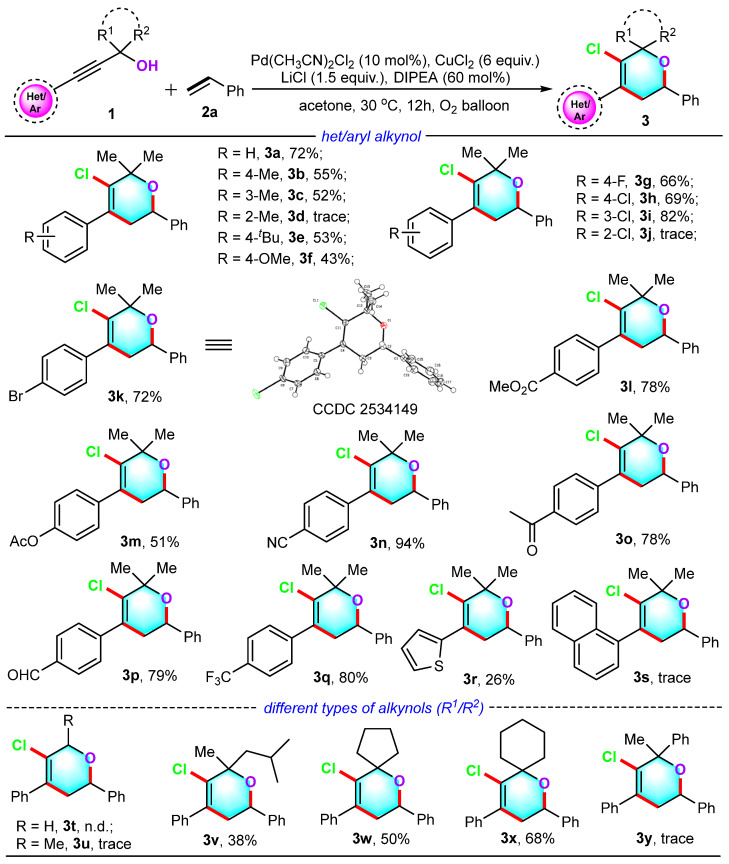
Substrate scope of alkynols.

With the optimized reaction conditions in hand, the substrate scope of this palladium-catalyzed carboetherification was firstly investigated by employing different alkynols, and the representative experimental results were displayed in Figure 1. It is noteworthy that a variety of alkynols bearing electron-neutral (**3a**–**3e**), electron-donating (**3f**), and electron-withdrawing (**3g**–**3q**) substituents on the phenyl ring were well compatible with the optimized reaction conditions, delivering the corresponding 3,6-dihydro-2*H*-pyran derivatives in yields ranging from 43% to 94%. Thus, the reaction of aryl alkynols carrying the substituents at the *para*- or *meta*-positions of the phenyl ring is well tolerated under the optimized catalytic systems, affording the corresponding products **3b** and **3c** in 55% and 52% yields, respectively. However, *ortho*-substituted substrates were the sterically hindered ones for this transformation, and failed to deliver the desired products **3d** and **3j**. Delightfully, various halogen groups on the aromatic ring were also tolerated to this catalytic transformation, generating the expected products **3g**–**3i** in good yields respectively. Moreover, the absolute configuration of products **3k** (CCDC 2534149) was determined unambiguously by single-crystal X-ray diffraction analysis. Notably, an impressive feature of this protocol is its high tolerance for functional groups. Representative examples include aryl alkynols with carboxylate ester group (**3l**), phenol ester group (**3m**), nitrile group (**3n**), acetyl (**3o**), aldehyde group (**3p**), and trifluoromethyl (**3q**), which were nicely accommodated, offering the corresponding products in good to excellent yields. It is worth mentioning that heterocyclic group, such as thiophene ring, was also amenable to this catalytic system, yielded the expected product **3r** in a low yield. As for the different kinds of classification of alkynols, primary alcohol and one methyl, which substituted secondary alcohol, were not participated under our catalytic conditions, and failed to furnish the desired products **3t** and **3u**. Particularly, tertiary alcohol expect for the dimethyl group, 3,5-dimethyl-1-phenylhex-1-yn-3-ol (**1v**), 1-(phenylethynyl)cyclopentan-1-ol (**1w**), and 1-(phenylethynyl)cyclohexan-1-ol (**1x**) were possible coupling partners, producing the corresponding products **3v**, **3w** and **3x** in moderate yields.

**Figure 2:**
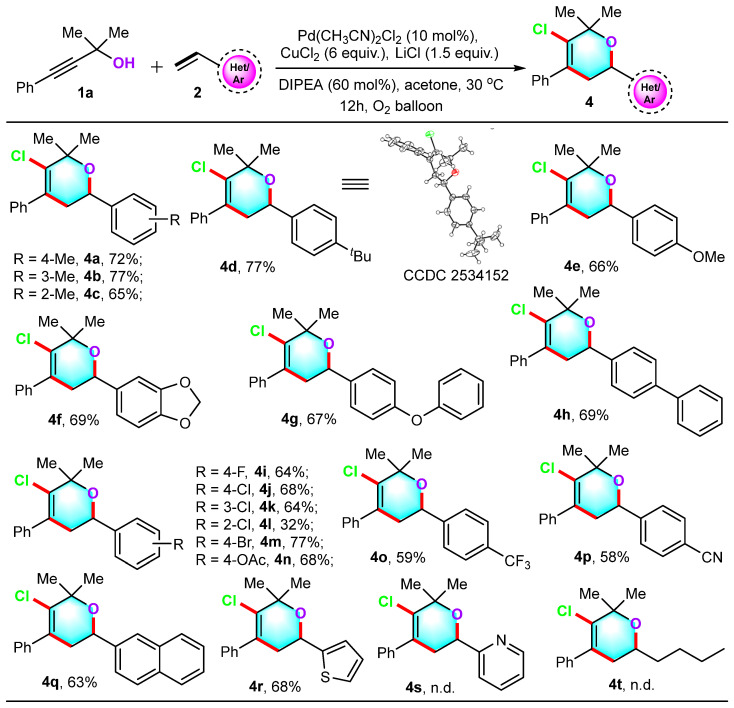
Substrate scope of alkenes.

Subsequently, for further demonstrating the synthetic potential of this approach, the substrate scope with regard to alkenes were next evaluated. As summarized in Figure 2, both electron-donating and electron-withdrawing substituents at *para*-, *meta*-, or *ortho*-position on the phenyl ring of the alkenes were compatible with the current catalytic system, giving the corresponding products in 32–77% yields. The absolute configuration of **4d** (CCDC 2534152) was also confirmed by X-ray diffraction analysis. Exemplified by 1-methoxy-4-vinylbenzene, 5-vinylbenzo[d][1,3]dioxole, and 1-phenoxy-4-vinylbenzene also worked well, producing the corresponding 2*H*-pyran derivatives **4e**, **4f**, and **4g** in 66%, 69%, and 67% yields, respectively. Additionally, substrate bearing a phenyl group proved viable in this reaction, furnishing the functionalized 2*H*-pyran derivative **4h** in good yield (69%). More importantly, the substrates containing a bulky substituent (**4g** and **4h**, 67% and 69%%) did not affect the yields. Diverse halo substituents also proved to be the effective coupling partners in this tandem annulation approach, affording structurally diverse 2*H*-pyran derivatives in high yields (up to 77%), which may provide the opportunity for further transformation and modification *via* palladium-catalyzed coupling protocol [65,66,67]. This tandem annulation proceeded even with a *p*-acetoxyphenyl alkene, furnishing the corresponding product **4n** in 68% yield. Additionally, as for the electron-deficient styrene derivatives **1o** and **1p**, the reaction gave the corresponding products **4o** and **4p** in similar yields. Especially, 2-vinylnaphthalene and heteroaromatic alkene could also be converted into the corresponding products **4q** and **4r** in good yields as well. However, alkyl alkene, such as hex-1-ene, proved completely ineffective, and failed to generate the desired product **4t**.

**Figure 3:**
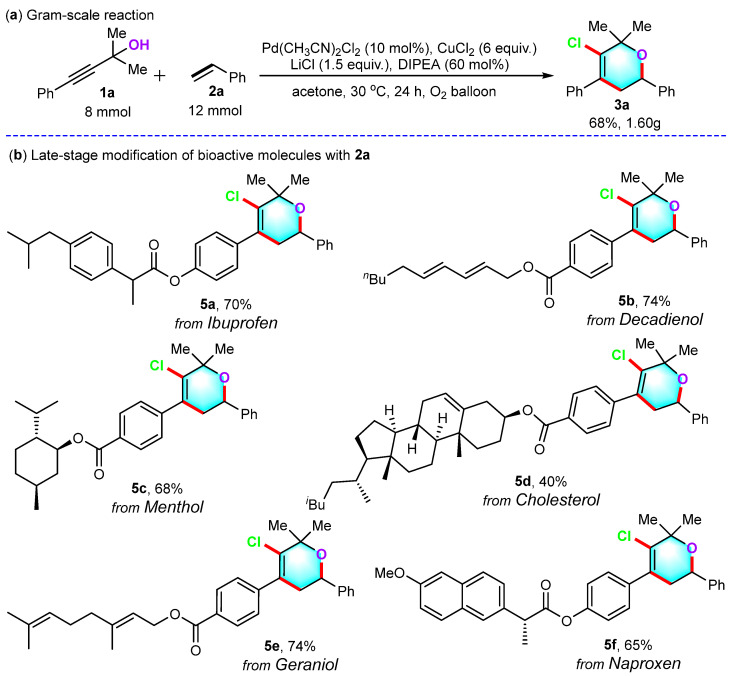
Gram-scale synthesis and late-stage modification of bioactive molecules with **2a**.

To further demonstrate the practicality and scalability of the current catalytic strategy, gram-scale synthesis and synthetic transformations were then conducted in Figure 3. It is particularly noteworthy that this cascade annulation reaction was scalable by the isolation of 1.60 g of **3a** (68% yield) from 8 mmol scale reaction of **1a** under the modified reaction conditions (Figure 3a). It is rather remarkable that this method can be successfully applied to an array of natural product derivatives and pharmaceutical analogs for late-stage functionalization. In particular, several styrene derived from Ibuprofen (**5a**), Decadienol (**5b**), Menthol (**5c**), Cholesterol (**5d**), Geraniol (**5e**), and Naproxen (**5f**) smoothly provided the corresponding 2*H*-pyran derivatives in good yields under the optimized reaction conditions (Figure 3b). Therefore, this synthetic strategy not only presents a straightforward and step-economical synthesis but also provides a versatile opportunity for the derivatization and late-stage functionalization.

**Scheme 2:**
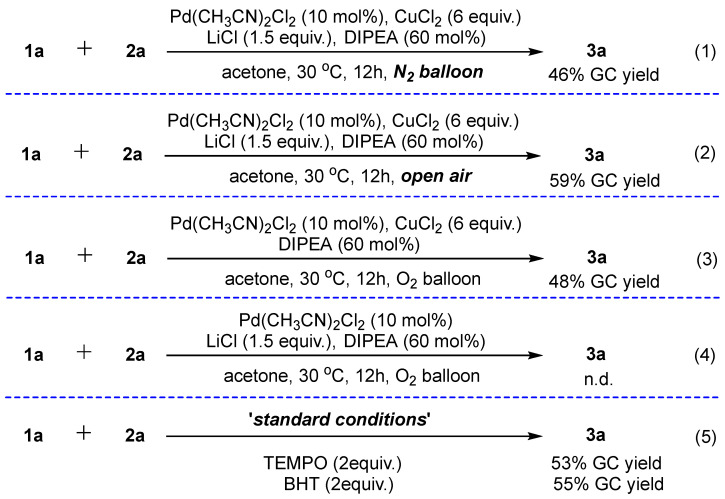
Control experiments.

To elucidate the possible reaction mechanism of this catalytic system, several control experiments were further performed (Scheme 2). When **1a** with **2a** was employed under the N_2_ atmosphere condition, the efficiency of this carboetherification approach was significantly decreased, and the product **3a** was detected in 46% GC yield by GC-MS analysis ((1) in Scheme 2). As expected, when the reaction was carried out in an air atmosphere, the yield of **3a** was slightly increased ((2) in Scheme 2). These observations suggested that oxidative conditions may play a crucial role for this cascade carboetherification reaction. It is interesting to note that the yield of **3a** was significantly decreased in the absence of LiCl under the optimized reaction conditions. Previous work supports that the lithium halides can significantly promote halopalladation-initiated tandem reactions [68,69,70]. In addition, when **1a** with **2a** was allowed to react in the absence of CuCl_2_, the desired product **3a** was not detected by GC-MS analysis ((3) in Scheme 2). This result indicated that the Cu^2+^ as the oxidant played an irreplaceable role in this carboetherification reaction ((4) in Scheme 2). Furthermore, when TEMPO (2,2,6,6-tetramethylpiperidinooxy) and BHT (2,6-di-tbutyl-4-methylphenol) were employed as radical trapping reagents under the “standard conditions”, the formation of **3a** in 53% and 55% yields, respectively. These results revealed that the radical intermediate was unlikely to be involved in this cascade reaction ((5) in Scheme 2).

**Scheme 3:**
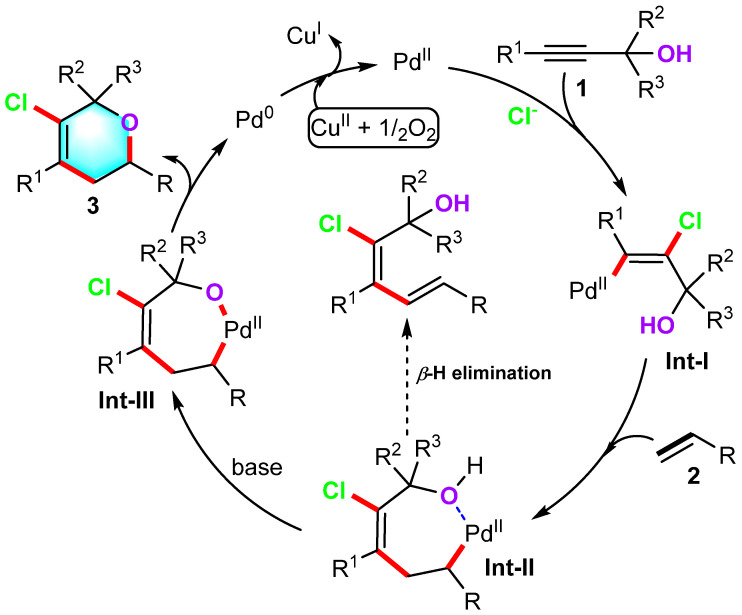
Plausible mechanistic pathways.

On the basis of our experimental observations and the relevant literature precedents, a tentative reaction mechanism for this palladium-catalyzed carboetherification of alkenes is presented in Scheme 3. Initially, in the presence of excess chloride ions, *trans*-chloropalladation of alkynols affords the vinylpalladium intermediate **Int-I** [71,72]. Subsequently, the catalytic species of **Int-I** was then captured by the alkene through the migratory insertion process to give σ-alkyl-Pd catalytic-active species **Int-II** [73]. In our previous studies, we found that the C(sp^3^)-Pd bond would undergo *β*-H elimination reaction in the absence of a coordinating group (the dashed-line arrows) [74,75,76]. The hydroxyl group of alkynols chelates with the Pd^II^ catalytic center to achieve coordination saturation, which may effectively suppress the elimination process [77]. As a result, the coordination interaction of hydroxyl group with palladium produces the seven-membered ring palladacycle intermediate **Int-III** with the assistance of the base. Then, a reductive elimination process of palladacycle intermediate **Int-III** generates the desired product **3** and zero-valent palladium active species. Finally, the Pd^II^ active species is regenerated by the oxidation with the Cu^II^ and O_2,_ completing this catalytic cycle.

## 3. Materials and Methods

### 3.1. Materials

All the reagents were obtained from commercial sources and used directly without further purification. Melting points were measured on an Electrothemal SGW-X4 microscopy digital melting point apparatus (Wagner and Münz, Munich, Germany) and are uncorrected. The IR spectra were measured with an Agilent Cary 630 FT-IR spectrometer (Agilent Technologies, Santa Clara, CA, USA) using potassium bromide (KBr) pellet. ^1^H NMR and ^13^C NMR spectra were recorded on a Bruker AVANCE III 400 MHz (Bruker, Billerica, MA, USA) using CDCl_3_ as a solvent. The chemical shifts are referenced to signals at 0 or 7.26 and 77.0 ppm and chloroform as the solvent, with TMS as the internal standard. Chemical shifts (δ) are reported in ppm and coupling constants (J) are reported in Hertz (Hz). ^19^F NMR spectra were collected at 376 MHz on the same instrument and are referenced to internal C_6_H_5_F at δ −113.15. GC-MS data were obtained with a Thermo Scientific TRACE (Milan, Italy) 1300 and ISQ QD system equipped with a TG-5MS column. MS (ESI) was taken with a Varian 500-MS LC Ion Trap (Varian, Inc., Walnut Creek, CA, USA). High-resolution mass spectra (HRMS) were recorded on a JEOL JMS-600 spectrometer (Waters Corporation, Beijing, China). X-ray diffraction was measured on a ‘Bruker APEX-II CCD’ diffractometer (Bruker, Billerica, MA, USA) with Cu-K_α_ radiation. TLC analysis was performed on pre-coated, glass-backed silica gel plates (Qingdao Haiyang, Qingdao, China) and visualized with UV light. Flash column chromatography was performed on silica gel (200–300 mesh), with petroleum ether and ethyl acetate (Guanghua Corporation, Guangzhou, China) as eluents.

### 3.2. General Methods for the Preparation of Functionalized 2H-Pyran Derivatives

A mixture of Pd(CH_3_CN)Cl_2_ (0.02 mmol, 10 mol %), CuCl_2_ (1.2 mmol, 6 equiv.), LiCl (0.2 mmol, 1.5 equiv.), propargyl alcohol (**1**, 0.2 mmol), and acetone (2 mL) was added to a tube equipped with a stir-bar. Then, DIPEA (1.2 mmol, 60 mol %), and alkenes (**2**, 0.3 mmol, 1.5 equiv.) were added to the tube under oxygen and stirred at 30°C for 12 h. After the reaction was finished, the reaction was quenched by saturated aqueous NH_4_Cl and extracted with EtOAc three times. The combined organic layers were dried over anhydrous Na_2_SO_4_ and evaporated under vacuum. The residue was purified by flash column chromatography on silica gel (hexanes/ethyl acetate) to afford the desired functionalized 2*H*-pyran derivatives.

5-Chloro-6,6-dimethyl-2,4-diphenyl-3,6-dihydro-2H-pyran (**3a**): Alkynol **1a** (0.20 mmol) with styrene **2a** (0.30 mmol, 1.5 equiv.) gave compound **3a** (72%, 42.9 mg) as a yellow solid, mp = 60.5–61.0 °C; ^1^H NMR (400 MHz, CDCl_3_) δ 7.47–7.43 (m, 2H), 7.38 (td, *J* = 7.5, 4.4 Hz, 4H), 7.35–7.27 (m, 4H), 4.96 (dd, *J* = 10.7, 3.2 Hz, 1H), 2.78 (dd, *J* = 16.9, 10.6 Hz, 1H), 2.60 (dd, *J* = 16.9, 3.1 Hz, 1H), 1.62 (d, *J* = 2.1 Hz, 6H); ^13^C NMR (100 MHz, CDCl_3_) δ 141.6, 139.9, 133.5, 131.9, 128.5, 128.2, 128.2, 127.7, 127.5, 126.1, 70.3, 40.9, 28.6, 24.3; ν_max_(KBr)/cm^−1^ 2989, 1953, 1645, 1450, 1079, 962, 698, 578; MS (EI) *m*/*z* 77, 91, 129, 142, 157, 177, 192, 202, 219, 247, 263, 283, 298; HRMS-APCI (*m*/*z*): cal for C_19_H_20_ClO, [M + H]^+^: 299.1197, found 299.1199.

5-Chloro-6,6-dimethyl-2-phenyl-4-(p-tolyl)-3,6-dihydro-2H-pyran (**3b**): Alkynol **1b** (0.20 mmol) with styrene **2a** (0.30 mmol, 1.5 equiv.) gave compound **3b** (55%, 34.3 mg) as a white solid, mp = 65.3–66.0 °C; ^1^H NMR (400 MHz, CDCl_3_) δ 7.34 (d, *J* = 7.6 Hz, 2H), 7.26 (t, *J* = 7.1 Hz, 2H), 7.21–7.14 (m, 2H), 7.05–6.99 (m, 3H), 4.84 (dd, *J* = 10.7, 2.9 Hz, 1H), 2.65 (ddt, *J* = 16.6, 10.7, 3.0 Hz, 1H), 2.52–2.45 (m, 1H), 2.27 (d, *J* = 5.5 Hz, 3H), 1.52 (d, *J* = 4.3 Hz, 6H); ^13^C NMR (100 MHz, CDCl_3_) δ 141.7, 139.9, 137.9, 133.3, 132.0, 128.7, 128.5, 128.2, 128.2, 127.7, 126.1, 125.2, 70.3, 41.0, 28.6, 24.3, 21.5; ν_max_(KBr)/cm^−1^ 2076, 1619, 1355, 1070, 958, 698, 620; MS (EI) *m*/*z* 77, 91, 105, 141, 156, 171, 206, 219, 243, 261, 297, 312; HRMS-APCI (*m*/*z*): calcd for C_20_H_22_ClO, [M + H]^+^: 313.1354, found 313.1360.

5-Chloro-6,6-dimethyl-2-phenyl-4-(m-tolyl)-3,6-dihydro-2H-pyran (**3c**): Alkynol **1c** (0.20 mmol) with styrene **2a** (0.30 mmol, 1.5 equiv.) gave compound **3c** (52%, 32.4 mg) as a white solid, mp = 55.2–56.4 °C; ^1^H NMR (400 MHz, CDCl_3_) δ 7.46 (d, *J* = 7.3 Hz, 2H), 7.39 (t, *J* = 7.6 Hz, 2H), 7.34–7.26 (m, 2H), 7.18–7.11 (m, 3H), 4.96 (dd, *J* = 10.6, 3.3 Hz, 1H), 2.78 (dd, *J* = 16.9, 10.8 Hz, 1H), 2.60 (dd, *J* = 16.9, 3.1 Hz, 1H), 2.39 (s, 3H), 1.63 (d, *J* = 3.0 Hz, 6H); ^13^C NMR (100 MHz, CDCl_3_) δ 155.8, 141.7, 139.9, 137.9, 133.3, 132.0, 128.7, 128.5, 128.2, 128.2, 127.7, 126.1, 125.2, 70.3, 41.0, 28.6, 24.3, 21.5; ν_max_(KBr)/cm^−1^ 2980, 1618, 1369, 1070, 964, 623; MS (EI) *m*/*z* 77, 91, 105, 141, 156, 171, 206, 219, 243, 261, 297, 312; HRMS-APCI (*m*/*z*): calcd for C_20_H_22_ClO, [M + H]^+^: 313.1354, found 313.1364.

4-(4-(Tert-butyl)phenyl)-5-chloro-6,6-dimethyl-2-phenyl-3,6-dihydro-2H-pyran (**3e**): Alkynol **1e** (0.20 mmol) with styrene **2a** (0.30 mmol, 1.5 equiv.) gave compound **3e** (53%, 37.5 mg) as a yellow solid, mp = 61.2–61.7; ^1^H NMR (400 MHz, CDCl_3_) δ 7.37–7.31 (m, 2H), 7.31–7.24 (m, 4H), 7.18 (d, *J* = 8.4 Hz, 3H), 4.85 (dd, *J* = 10.8, 3.1 Hz, 1H), 2.67 (dd, *J* = 16.9, 10.8 Hz, 1H), 2.51 (dd, *J* = 16.9, 3.1 Hz, 1H), 1.52 (d, *J* = 2.9 Hz, 6H), 1.24 (s, 9H); ^13^C NMR (100 MHz, CDCl_3_) δ 150.3, 141.7, 136.8, 133.1, 131.6, 128.5, 127.8, 127.7, 126.1, 125.1, 77.4, 70.3, 40.9, 34.6, 31.4, 28.6, 24.3; ν_max_(KBr)/cm^−1^ 2963, 1617, 1360, 1069, 962, 621; MS (EI) *m*/*z* 77, 91, 105, 141, 157, 192, 205, 248, 283, 339, 354; HRMS-APCI (*m*/*z*): calcd for C_23_H_28_ClO, [M + H]^+^: 355.1823, found 355.1816.

5-Chloro-4-(4-methoxyphenyl)-6,6-dimethyl-2-phenyl-3,6-dihydro-2H-pyran (**3f**): Alkynol **1f** (0.20 mmol) with styrene **2a** (0.30 mmol, 1.5 equiv.) gave compound **3f** (43%, 28.3 mg) as a white solid, mp = 62.5–63.2; ^1^H NMR (400 MHz, CDCl_3_) δ 7.44 (d, *J* = 7.2 Hz, 2H), 7.37 (dd, *J* = 8.5, 6.7 Hz, 2H), 7.32–7.26 (m, 3H), 6.94–6.88 (m, 2H), 4.94 (dd, *J* = 10.7, 3.1 Hz, 1H), 3.83 (s, 3H), 2.75 (dd, *J* = 16.8, 10.7 Hz, 1H), 2.58 (dd, *J* = 16.9, 3.2 Hz, 1H), 1.61 (d, *J* = 2.7 Hz, 6H); ^13^C NMR (100 MHz, CDCl_3_) δ 158.8, 141.7, 133.1, 132.1, 131.3, 129.4, 128.5, 127.7, 126.1, 113.6, 77.4, 70.3, 55.3, 41.0, 28.6, 24.3; ν_max_(KBr)/cm^−1^ 2918, 1619, 1354, 1071, 964, 619; MS (EI) *m*/*z* 77, 91, 105, 141, 172, 207, 249, 293, 313, 328; HRMS-APCI (*m*/*z*): calcd for C_20_H_22_ClO, [M + H]^+^: 329.1303, found 329.1301.

5-Chloro-4-(4-fluorophenyl)-6,6-dimethyl-2-phenyl-3,6-dihydro-2H-pyran (**3g**): Alkynol **1g** (0.20 mmol) with styrene **2a** (0.30 mmol, 1.5 equiv.) gave compound **3g** (66%, 41.5 mg) as a yellow solid, mp = 77.3–77.8; ^1^H NMR (400 MHz, CDCl_3_) δ 7.47–7.42 (m, 2H), 7.41–7.36 (m, 2H), 7.34–7.28 (m, 3H), 7.11–7.03 (m, 2H), 4.95 (dd, *J* = 10.6, 3.1 Hz, 1H), 2.76 (dd, *J* = 16.9, 10.8 Hz, 1H), 2.57 (dd, *J* = 16.9, 3.1 Hz, 1H), 1.62 (d, *J* = 2.4 Hz, 6H); ^13^C NMR (100 MHz, CDCl_3_) δ 162.0 (d, *J* = 246.7 Hz), 141.5, 135.7 (d, *J* = 3.3 Hz), 133.9, 130.9, 129.9 (d, *J* = 8.4 Hz), 128.5, 127.8, 126.1, 115.2 (d, *J* = 21.4 Hz), 77.4, 70.3, 41.0, 28.5, 24.2; ^19^F NMR (376 MHz, CDCl_3_) δ −114.3; ν_max_(KBr)/cm^−1^ 2981, 2096, 1640, 1077, 697, 647; MS (EI) *m*/*z* 77, 91, 105, 133, 160, 175, 210, 247, 266, 301, 316; HRMS-APCI (*m*/*z*): calcd for C_19_H_17_ClFO, [M-H]^−^: 315.0946, found 315.0943.

5-Chloro-4-(4-chlorophenyl)-6,6-dimethyl-2-phenyl-3,6-dihydro-2H-pyran (**3h**): Alkynol **1h** (0.20 mmol) with styrene **2a** (0.30 mmol, 1.5 equiv.) gave compound **3h** (69%, 45.8 mg) as a yellow solid, mp = 66.6–67.2; ^1^H NMR (400 MHz, CDCl_3_) δ 7.41 (d, *J* = 7.3 Hz, 2H), 7.37–7.30 (m, 4H), 7.30–7.22 (m, 3H), 4.92 (dd, *J* = 10.7, 3.2 Hz, 1H), 2.72 (dd, *J* = 16.8, 10.7 Hz, 1H), 2.52 (dd, *J* = 16.9, 3.1 Hz, 1H), 1.59 (d, *J* = 3.4 Hz, 6H); ^13^C NMR (100 MHz, CDCl_3_) δ 141.5, 138.2, 134.2, 133.3, 130.8, 129.6, 128.5, 128.5, 127.8, 126.1, 77.4, 70.3, 40.8, 28.5, 24.3; ν_max_(KBr)/cm^−1^ 2981, 1637, 1082, 576; MS (EI) *m*/*z* 77, 91, 105, 141, 156, 191, 226, 239, 263, 282, 317, 332; HRMS-APCI (*m*/*z*): calcd for C_19_H_19_Cl_2_O, [M + H]^+^: 333.0807, found 333.0802.

5-Chloro-4-(3-chlorophenyl)-6,6-dimethyl-2-phenyl-3,6-dihydro-2H-pyran (**3i**): Alkynol **1i** (0.20 mmol) with styrene **2a** (0.30 mmol, 1.5 equiv.) gave compound **3i** (82%, 54.5 mg) as a yellow solid, mp = 53.6–54.3; ^1^H NMR (400 MHz, CDCl_3_) δ 7.45–7.41 (m, 2H), 7.37 (td, *J* = 7.4, 1.2 Hz, 2H), 7.34–7.26 (m, 4H), 7.21 (dt, *J* = 7.1, 1.7 Hz, 1H), 4.93 (dd, *J* = 10.6, 3.1 Hz, 1H), 2.75 (dd, *J* = 16.9, 10.6 Hz, 1H), 2.55 (dd, *J* = 16.9, 3.1 Hz, 1H), 1.61 (d, *J* = 1.8 Hz, 6H); ^13^C NMR (100 MHz, CDCl_3_) δ 141.6, 141.4, 134.4, 134.1, 130.7, 129.6, 128.5, 128.4, 127.8, 127.6, 126.4, 126.0, 77.4, 70.2, 40.7, 28.5, 24.2; ν_max_(KBr)/cm^−1^ 2982, 1618, 1508, 1069, 836, 621, 512; MS (EI) *m*/*z* 77, 91, 105, 141, 156, 191, 226, 239, 263, 282, 317, 332; HRMS-APCI (*m*/*z*): calcd for C_19_H_19_Cl_2_O, [M + H]^+^: 333.0807, found 333.0800.

4-(4-Bromophenyl)-5-chloro-6,6-dimethyl-2-phenyl-3,6-dihydro-2H-pyran (**3k**): Alkynol **1k** (0.20 mmol) with styrene **2a** (0.30 mmol, 1.5 equiv.) gave compound **3k** (72%, 54.1 mg) as a yellow solid, mp = 113.4–113.9; ^1^H NMR (400 MHz, CDCl_3_) δ 7.40–7.36 (m, 2H), 7.34–7.30 (m, 2H), 7.29–7.24 (m, 2H), 7.21–7.16 (m, 1H), 7.11–7.07 (m, 2H), 4.82 (dd, *J* = 10.7, 3.2 Hz, 1H), 2.63 (dd, *J* = 16.8, 10.7 Hz, 1H), 2.43 (dd, *J* = 16.9, 3.1 Hz, 1H), 1.50 (d, *J* = 4.3 Hz, 6H); ^13^C NMR (100 MHz, CDCl_3_) δ 141.5, 138.7, 134.2, 131.5, 130.9, 130.0, 128.6, 127.8, 126.1, 121.5, 77.4, 70.3, 40.7, 28.5, 24.3; ν_max_(KBr)/cm^−1^ 2926, 2091, 1639, 1074, 753, 574; MS (EI) *m*/*z* 77, 91, 105, 141, 156, 176, 191, 203, 272, 343, 363, 376; HRMS-APCI (*m*/*z*): calcd for C_19_H_19_BrClO, [M + H]^+^: 377.0302, found 377.0297.

Methyl 4-(5-chloro-6,6-dimethyl-2-phenyl-3,6-dihydro-2H-pyran-4-yl)benzoate (**3l**): Alkynol **1l** (0.20 mmol) with styrene **2a** (0.30 mmol, 1.5 equiv.) gave compound **3l** (78%, 55.5 mg) as a yellow solid, mp = 81.3–82.4; ^1^H NMR (400 MHz, CDCl_3_) δ 8.10–8.05 (m, 2H), 7.47–7.42 (m, 3H), 7.42–7.36 (m, 3H), 7.33–7.28 (m, 1H), 4.97 (dd, *J* = 10.6, 3.1 Hz, 1H), 3.93 (s, 3H), 2.79 (dd, *J* = 16.9, 10.6 Hz, 1H), 2.59 (dd, *J* = 16.9, 3.1 Hz, 1H), 1.63 (d, *J* = 3.0 Hz, 6H); ^13^C NMR (100 MHz, CDCl_3_) δ 166.8, 144.6, 141.4, 134.5, 131.2, 129.6, 129.2, 128.5, 128.3, 127.8, 126.1, 77.4, 70.3, 52.2, 40.6, 28.5, 24.3; ν_max_(KBr)/cm^−1^ 2960, 2983, 2074, 1639, 1022, 702, 576; MS (EI) *m*/*z* 77, 91, 105, 141, 156, 191, 215, 235, 263, 309, 325, 341, 356; HRMS-APCI (*m*/*z*): calcd for C_21_H_22_ClO_3_, [M + H]^+^: 357.1252, found 357.1247.

4-(5-Chloro-6,6-dimethyl-2-phenyl-3,6-dihydro-2H-pyran-4-yl)phenyl acetate (**3m**): Alkynol **1m** (0.20 mmol) with styrene **2a** (0.30 mmol, 1.5 equiv.) gave compound **3m** (51%, 38.0 mg) as a colorless oil; ^1^H NMR (400 MHz, CDCl_3_) δ 7.47–7.43 (m, 2H), 7.41–7.35 (m, 4H), 7.34–7.30 (m, 1H), 7.14–7.09 (m, 2H), 4.95 (dd, *J* = 10.7, 3.1 Hz, 1H), 2.77 (dd, *J* = 16.9, 10.7 Hz, 1H), 2.60 (dd, *J* = 16.9, 3.1 Hz, 1H), 2.33 (s, 3H), 1.62 (s, 6H); ^13^C NMR (100 MHz, CDCl_3_) δ 169.5, 149.8, 141.5, 137.3, 133.9, 131.0, 129.4, 128.5, 128.5, 127.8, 126.0, 126.0, 121.3, 77.4, 70.2, 40.8, 28.5, 24.3, 21.2; ν_max_(KBr)/cm^−1^ 3673, 2986, 2902, 1758, 1507, 1391, 1204, 1167, 1053, 911; MS (EI) *m*/*z* 77, 91, 105, 173, 193, 235, 263, 299, 321, 341, 356; HRMS-APCI (*m*/*z*): calcd for C_21_H_25_ClO_3_N_1_, [M + NH_3_ + H]^+^: 374.1517, found 374.1516.

4-(5-Chloro-6,6-dimethyl-2-phenyl-3,6-dihydro-2H-pyran-4-yl)benzonitrile (**3n**): Alkynol **1n** (0.20 mmol) with styrene **2a** (0.30 mmol, 1.5 equiv.) gave compound **3n** (94%, 60.7 mg) as a white solid, mp = 148.2–148.8 °C; ^1^H NMR (400 MHz, CDCl_3_) δ 7.69–7.64 (m, 2H), 7.46–7.40 (m, 4H), 7.40–7.35 (m, 2H), 7.33–7.28 (m, 1H), 4.94 (dd, *J* = 10.6, 3.1 Hz, 1H), 2.76 (dd, *J* = 16.8, 10.6 Hz, 1H), 2.54 (dd, *J* = 16.8, 3.1 Hz, 1H), 1.61 (s, 6H); ^13^C NMR (100 MHz, CDCl_3_) δ 144.5, 141.2, 135.4, 132.1, 130.5, 129.1, 128.6, 127.9, 126.0, 118.7, 111.3, 77.4, 70.2, 40.4, 28.4, 24.2; ν_max_(KBr)/cm^−1^ 2926, 2220, 1602, 1167, 1074, 958, 839, 767, 694; MS (EI) *m*/*z* 77, 91, 105, 140, 167, 182, 202, 217, 244, 288, 308, 323; HRMS-APCI (*m*/*z*): calcd for C_20_H_19_ClNO, [M + H]^+^: 324.1150, found 324.1145.

1-(4-(5-Chloro-6,6-dimethyl-2-phenyl-3,6-dihydro-2H-pyran-4-yl)phenyl)ethan-1-one (**3o**): Alkynol **1o** (0.20 mmol) with styrene **2a** (0.30 mmol, 1.5 equiv.) gave compound **3o** (78%, 53.0 mg) as a yellow oil; ^1^H NMR (400 MHz, CDCl_3_) δ 7.97 (d, *J* = 8.4 Hz, 2H), 7.47–7.41 (m, 4H), 7.37 (t, *J* = 7.5 Hz, 2H), 7.30 (dd, *J* = 8.4, 5.9 Hz, 1H), 4.96 (dd, *J* = 10.7, 3.2 Hz, 1H), 2.78 (dd, *J* = 16.9, 10.6 Hz, 1H), 2.62 (s, 3H), 2.60–2.55 (m, 1H), 1.62 (d, *J* = 1.9 Hz, 6H); ^13^C NMR (100 MHz, CDCl_3_) δ 197.7, 144.7, 141.4, 136.0, 134.6, 131.1, 128.6, 128.5, 128.4, 127.9, 126.1, 77.4, 70.2, 40.6, 28.5, 26.7, 24.3; ν_max_(KBr)/cm^−1^ 2989, 2093, 1682, 1266, 1090, 604; MS (EI) *m*/*z* 77, 91, 105, 141, 156, 176, 199, 234, 283, 305, 325, 340; HRMS-APCI (*m*/*z*): calcd for C_21_H_22_ClO_2_, [M + H]+: 341.1303, found 341.1302.

4-(5-Chloro-6,6-dimethyl-2-phenyl-3,6-dihydro-2H-pyran-4-yl)benzaldehyde (**3p**): Alkynol **1p** (0.20 mmol) with styrene **2a** (0.30 mmol, 1.5 equiv.) gave compound **3p** (79%, 51.5 mg) as a yellow oil; ^1^H NMR (400 MHz, CDCl_3_) δ 10.04 (s, 1H), 7.92 (d, *J* = 6.3 Hz, 2H), 7.52 (d, *J* = 8.3 Hz, 2H), 7.46 (dd, *J* = 8.4, 1.5 Hz, 2H), 7.42–7.37 (m, 2H), 7.35–7.30 (m, 1H), 4.98 (dd, *J* = 10.6, 3.1 Hz, 1H), 2.81 (dd, *J* = 16.8, 10.7 Hz, 1H), 2.60 (dd, *J* = 16.8, 3.1 Hz, 1H), 1.64 (s, 6H); ^13^C NMR (100 MHz, CDCl_3_) δ 191.8, 146.1, 141.3, 135.3, 134.9, 131.0, 129.8, 129.0, 128.6, 127.9, 126.0, 77.5, 70.2, 40.5, 28.4, 24.2; ν_max_(KBr)/cm^−1^ 2984, 1636, 1170, 838, 576; MS (EI) *m*/*z* 77, 91, 105, 141, 157, 192, 247, 275, 311, 326; HRMS-APCI (*m*/*z*): calcd for C_20_H_20_ClO_2_, [M + H]^+^: 327.1146, found 327.1141.

Chloro-6,6-dimethyl-2-phenyl-4-(4-(trifluoromethyl)phenyl)-3,6-dihydro-2H-pyran (**3q**): Alkynol **1q** (0.20 mmol) with styrene **2a** (0.30 mmol, 1.5 equiv.) gave compound **3q** (80%, 58.6mg) as a yellow solid, mp = 68.4–69.6 °C; ^1^H NMR (400 MHz, CDCl_3_) δ 7.66 (d, *J* = 8.3 Hz, 2H), 7.47 (d, *J* = 8.1 Hz, 4H), 7.43–7.37 (m, 2H), 7.36–7.30 (m, 1H), 4.99 (dd, *J* = 10.6, 3.1 Hz, 1H), 2.80 (dd, *J* = 16.9, 10.6 Hz, 1H), 2.59 (dd, *J* = 16.8, 3.2 Hz, 1H), 1.65 (d, *J* = 2.3 Hz, 6H); ^13^C NMR (100 MHz, CDCl_3_) δ 143.5, 141.4, 134.8, 130.9, 129.6 (q, *J* = 33.1 Hz), 128.7, 128.6, 127.9, 126.1, 125.3 (q, *J* = 4.0 Hz), 124.2 (q, *J* = 271.9 Hz), 77.4, 70.2, 40.7, 28.5, 24.2; ^19^F NMR (376 MHz, CDCl_3_) δ −62.5; ν_max_(KBr)/cm^−1^ 2983, 1616, 1325, 1167, 1065, 608; MS (EI) *m*/*z* 77, 91, 105, 141, 156, 191, 210, 225, 245, 260, 287, 315, 351, 366; HRMS-APCI (*m*/*z*): calcd for C_20_H_19_ClF_3_O, [M + H]^+^: 367.1071, found 367.1064.

5-Chloro-6,6-dimethyl-2-phenyl-4-(thiophen-2-yl)-3,6-dihydro-2H-pyran (**3r**): Alkynol **1r** (0.20 mmol) with styrene **2a** (0.30 mmol, 1.5 equiv.) gave compound **3r** (26%, 15.8mg) as a brown solid, mp = 43.8–44.5 °C; ^1^H NMR (400 MHz, CDCl_3_) δ 7.48–7.44 (m, 2H), 7.43–7.37 (m, 2H), 7.36–7.29 (m, 3H), 7.05 (dd, *J* = 5.3, 3.8 Hz, 1H), 4.92 (dd, *J* = 8.5, 5.4 Hz, 1H), 2.89–2.83 (m, 2H), 1.62 (d, *J* = 0.9 Hz, 6H); ^13^C NMR (100 MHz, CDCl_3_) δ 141.6, 140.6, 133.0, 128.6, 127.9, 126.8, 126.3, 126.2, 126.1, 124.2, 77.9, 70.3, 39.9, 28.9, 24.5; ν_max_(KBr)/cm^−1^ 2928, 1619, 1071, 961, 620; MS (EI) *m*/*z* 77, 91, 105, 148, 165, 198, 225, 269, 289, 304; HRMS-APCI (*m*/*z*): calcd for C_17_H_18_ClOS, [M + H]^+^: 305.0761, found 305.0757.

5-Chloro-6-isobutyl-6-methyl-2,4-diphenyl-3,6-dihydro-2H-pyran (**3v**): Alkynol **1v** (0.20 mmol) with styrene **2a** (0.30 mmol, 1.5 equiv.) gave compound **3v** (38%, 25.8mg) as a white oil; ^1^H NMR (400 MHz, CDCl_3_) δ 7.44 (d, *J* = 7.1 Hz, 2H), 7.41–7.34 (m, 4H), 7.34–7.27 (m, 4H), 4.99–4.92 (m, 1H), 2.74 (ddd, *J* = 16.0, 10.6, 5.4 Hz, 1H), 2.63–2.55 (m, 1H), 2.05–1.89 (m, 2H), 1.76–1.63 (m, 1H), 1.58 (d, *J* = 11.1 Hz, 3H), 1.17–0.97 (m, 6H); ^13^C NMR (100 MHz, CDCl_3_) δ 142.0, 141.7, 140.1, 140.0, 134.5, 133.3, 132.6, 131.5, 128.5, 128.4, 128.3, 128.2, 128.1, 128.1, 127.6, 127.5, 127.5, 127.4, 126.0, 125.9, 80.1, 79.6, 77.3, 70.6, 69.7, 48.5, 42.9, 41.5, 41.0, 25.6, 25.3, 24.9, 24.7, 24.7, 24.3, 24.0, 23.9; ν_max_(KBr)/cm^−1^ 2954, 1618, 1071, 962, 622; MS (EI) *m*/*z* 77, 91, 105, 141, 156, 191, 205, 247, 283, 325, 340; HRMS-APCI (*m*/*z*): calcd for C_22_H_26_ClO, [M + H]^+^: 341.1667, found 341.1669.

10-Chloro-7,9-diphenyl-6-oxaspiro[4.5]dec-9-ene (**3w**): Alkynol **1w** (0.20 mmol) with styrene **2a** (0.30 mmol, 1.5 equiv.) gave compound **3w** (50%, 32.4mg) as a white solid, mp = 70.4–71.2 °C; ^1^H NMR (400 MHz, CDCl_3_) δ 7.46–7.34 (m, 8H), 7.34–7.27 (m, 2H), 4.86 (dd, *J* = 10.6, 3.3 Hz, 1H), 2.79 (dd, *J* = 16.8, 10.6 Hz, 1H), 2.65 (dd, *J* = 16.9, 3.3 Hz, 1H), 2.43 (qd, *J* = 10.3, 6.0 Hz, 1H), 2.18–2.02 (m, 2H), 2.01–1.90 (m, 2H), 1.89–1.70 (m, 3H); ^13^C NMR (100 MHz, CDCl_3_) δ 141.8, 140.1, 132.8, 132.1, 128.4, 128.2, 127.5, 127.4, 125.9, 88.0, 70.8, 40.5, 39.3, 35.7, 25.5, 25.3; ν_max_(KBr)/cm^−1^ 2960, 1360, 1167, 1080, 1019, 961, 839, 760, 697, 562; MS (EI) *m*/*z* 77, 91, 115, 141, 167, 183, 218, 246, 271, 289, 306, 324; HRMS-APCI (*m*/*z*): calcd for C_21_H_22_ClO, [M + H]^+^: 325.1354, found 325.1347.

5-Chloro-2,4-diphenyl-1-oxaspiro[5.5]undec-4-ene (**3x**): Alkynol **1x** (0.20 mmol) with styrene **2a** (0.30 mmol, 1.5 equiv.) gave compound **3x** (68%, 46.0mg) as a white solid, mp = 92.3–93.2 °C; ^1^H NMR (400 MHz, CDCl_3_) δ 7.53–7.48 (m, 2H), 7.43–7.36 (m, 4H), 7.36–7.28 (m, 4H), 4.94 (dd, *J* = 9.7, 4.2 Hz, 1H), 2.79–2.66 (m, 2H), 2.24–2.09 (m, 2H), 1.98–1.86 (m, 2H), 1.82 (dd, *J* = 12.9, 2.4 Hz, 1H), 1.76–1.64 (m, 3H), 1.63–1.21 (m, 3H); ^13^C NMR (100 MHz, CDCl_3_) δ 142.1, 140.3, 134.2, 132.4, 128.4, 128.2, 128.2, 127.4, 127.4, 125.7, 77.8, 68.6, 40.8, 35.8, 29.9, 25.3, 21.4, 21.2; ν_max_(KBr)/cm^−1^ 3061, 2930, 1601, 1069, 946, 697, 541; MS (EI) *m*/*z* 77, 91, 115, 155, 197, 232, 246, 281, 303, 338; HRMS-APCI (*m*/*z*): calcd for C_22_H_24_ClO, [M + H]^+^: 339.1510, found 339.1511.

5-Chloro-6,6-dimethyl-4-phenyl-2-(p-tolyl)-3,6-dihydro-2H-pyran (**4a**): Alkynol **1a** (0.20 mmol) with 1-methyl-4-vinylbenzene **2b** (0.30 mmol, 1.5 equiv.) gave compound **4a** (72%, 44.9mg) as a yellow oil; ^1^H NMR (400 MHz, CDCl_3_) δ 7.36 (dd, *J* = 17.0, 7.4 Hz, 7H), 7.19 (d, *J* = 7.8 Hz, 2H), 4.92 (dd, *J* = 10.7, 3.1 Hz, 1H), 2.79 (dd, *J* = 16.9, 10.7 Hz, 1H), 2.58 (dd, *J* = 16.8, 3.1 Hz, 1H), 2.36 (s, 3H), 1.62 (s, 6H); ^13^C NMR (100 MHz, CDCl_3_) δ 140.0, 138.6, 137.4, 133.5, 131.9, 129.2, 128.2, 128.2, 127.4, 126.1, 77.4, 70.2, 40.9, 28., 24.3, 21.2; ν_max_(KBr)/cm^−1^ 2944, 2891, 2865, 1713, 1465, 1444, 1267, 1117, 1093, 998, 771, 747, 694; MS (EI) *m*/*z* 77, 91, 105, 115, 142, 157, 177, 192, 205, 233, 262, 297, 312; HRMS-APCI (*m*/*z*): calcd for C_20_H_22_ClO, [M + H]^+^: 313.1354, found 313.1351.

5-Chloro-6,6-dimethyl-4-phenyl-2-(m-tolyl)-3,6-dihydro-2H-pyran (**4b**): Alkynol **1a** (0.20 mmol) with 1-methyl-3-vinylbenzene **2c** (0.30 mmol, 1.5 equiv.) gave compound **4b** (77%, 48.0mg) as a yellow oil; ^1^H NMR (400 MHz, CDCl_3_) δ 7.29–7.24 (m, 2H), 7.24–7.18 (m, 3H), 7.17–7.11 (m, 3H), 7.00 (d, *J* = 7.0 Hz, 1H), 4.81 (dd, *J* = 10.7, 3.2 Hz, 1H), 2.67 (dd, *J* = 16.9, 10.8 Hz, 1H), 2.47 (dd, *J* = 16.9, 3.1 Hz, 1H), 2.27 (s, 3H), 1.52 (d, *J* = 4.8 Hz, 6H); ^13^C NMR (100 MHz, CDCl_3_) δ 141.6, 140.0, 138.2, 133.5, 132.0, 128.5, 128.4, 128.3, 128.2, 127.5, 126.8, 123.2, 77.4, 70.4, 41.0, 28.6, 24.3, 21.6; ν_max_(KBr)/cm^−1^ 2076, 2860, 2141, 1719, 1616, 1489, 1380, 1279, 1101, 1081, 980, 942, 889, 869, 627; MS (EI) *m*/*z* 77, 91, 105, 115, 142, 157, 177, 192, 205, 233, 262, 297, 312; HRMS-APCI (*m*/*z*): calcd for C_20_H_22_ClO, [M + H]^+^: 313.1354, found 313.1354.

5-Chloro-6,6-dimethyl-4-phenyl-2-(o-tolyl)-3,6-dihydro-2H-pyran (**4c**): Alkynol **1a** (0.20 mmol) with 1-methyl-2-vinylbenzene **2d** (0.30 mmol, 1.5 equiv.) gave compound **4c** (65%, 40.6mg) as a yellow oil; ^1^H NMR (400 MHz, CDCl_3_) δ 7.52 (d, *J* = 7.5 Hz, 1H), 7.40–7.28 (m, 5H), 7.24–7.13 (m, 3H), 5.11 (dd, *J* = 10.8, 3.1 Hz, 1H), 2.80 (dd, *J* = 16.9, 10.8 Hz, 1H), 2.51 (dd, *J* = 16.9, 3.0 Hz, 1H), 2.40 (s, 3H), 1.61 (d, *J* = 6.1 Hz, 6H); ^13^C NMR (100 MHz, CDCl_3_) δ 139.9, 139.4, 135.0, 133.4, 132.1, 130.5, 128.2, 128.2, 127.7, 127.5, 126.5, 126.0, 77.5, 67.4, 39.5, 28.6, 24.3, 19.4; ν_max_(KBr)/cm^−1^ 3020, 2836, 2052, 1637, 1376, 1079, 751, 624; MS (EI) *m*/*z* 77, 91, 105, 115, 142, 157, 177, 192, 205, 233, 262, 297, 312; HRMS-APCI (*m*/*z*): calcd for C_20_H_22_ClO, [M + H]^+^: 313.1354, found 313.1363.

2-(4-(*tert*-Butyl)phenyl)-5-chloro-6,6-dimethyl-4-phenyl-3,6-dihydro-2H-pyran (**4d**): Alkynol **1a** (0.20 mmol) with 1-(*tert*-butyl)-4-vinylbenzene **2e** (0.30 mmol, 1.5 equiv.) gave compound **4d** (77%, 54.5mg) as a white solid, mp = 93.4–94.0 °C; ^1^H NMR (400 MHz, CDCl_3_) δ 7.41–7.33 (m, 6H), 7.33–7.27 (m, 3H), 4.91 (dd, *J* = 10.8, 3.1 Hz, 1H), 2.79 (dd, *J* = 16.8, 10.7 Hz, 1H), 2.56 (dd, *J* = 16.9, 3.1 Hz, 1H), 1.59 (s, 6H), 1.30 (s, 9H); ^13^C NMR (100 MHz, CDCl_3_) δ 150.7, 140.0, 138.5, 133.5, 132.0, 128.2, 128.2, 127.4, 125.9, 125.4, 77.4, 70.2, 40.7, 34.6, 31.4, 28.6, 24.3; ν_max_(KBr)/cm^−1^ 2963, 1637, 1368, 1084, 622; MS (EI) *m*/*z* 77, 91, 105, 115, 142, 157, 192, 205, 247, 263, 283, 319, 339, 354; HRMS-APCI (*m*/*z*): calcd for C_23_H_28_ClO, [M + H]^+^: 355.1823, found 355.1816.

5-Chloro-2-(4-methoxyphenyl)-6,6-dimethyl-4-phenyl-3,6-dihydro-2H-pyran (**4e**): Alkynol **1a** (0.20 mmol) with 1-methoxy-4-vinylbenzene **2f** (0.30 mmol, 1.5 equiv.) gave compound **4e** (66%, 43.3mg) as a white solid, mp = 72.2–72.7 °C; ^1^H NMR (400 MHz, CDCl_3_) δ 7.43–7.39 (m, 2H), 7.38–7.35 (m, 3H), 7.35–7.29 (m, 2H), 6.95–6.90 (m, 2H), 4.92 (dd, *J* = 10.8, 3.1 Hz, 1H), 3.82 (s, 3H), 2.80 (dd, *J* = 16.9, 10.6 Hz, 1H), 2.58 (dd, *J* = 16.9, 3.1 Hz, 1H), 1.63 (d, *J* = 2.0 Hz, 6H); ^13^C NMR (100 MHz, CDCl_3_) δ 159.2, 140.0, 133.8, 133.5, 132.0, 128.2, 128.2, 127.5, 113.9, 77.4, 70.0, 55.4, 40.9, 28.6, 24.3; ν_max_(KBr)/cm^−1^ 2918, 1616, 1248, 1082, 623; MS (EI) *m*/*z* 77, 91, 121, 142, 157, 192, 205, 249, 291, 313, 328; HRMS-APCI (*m*/*z*): calcd for C_20_H_22_ClO, [M + H]^+^: 329.1128, found 329.1122.

5-(5-Chloro-6,6-dimethyl-4-phenyl-3,6-dihydro-2H-pyran-2-yl)benzo[d][1,3]dioxole (**4f**): Alkynol **1a** (0.20 mmol) with 5-vinylbenzo[d][1,3]dioxole **2g** (0.30 mmol, 1.5 equiv.) gave compound **4f** (69%, 47.2 mg) as a yellow oil; ^1^H NMR (400 MHz, CDCl_3_) δ 7.43–7.37 (m, 2H), 7.36–7.29 (m, 3H), 6.99 (d, *J* = 1.9 Hz, 1H), 6.89 (dd, *J* = 8.0, 1.8 Hz, 1H), 6.81 (d, *J* = 8.0 Hz, 1H), 5.95 (q, *J* = 1.5 Hz, 2H), 4.88 (dd, *J* = 10.6, 3.1 Hz, 1H), 2.77 (dd, *J* = 16.9, 10.6 Hz, 1H), 2.56 (dd, *J* = 16.9, 3.1 Hz, 1H), 1.62 (s, 6H); ^13^C NMR (100 MHz, CDCl_3_) δ 147.9, 147.1, 139.9, 135.6, 133.4, 131.8, 128.3, 128.2, 127.5, 119.6, 108.2, 106.9, 101.1, 77.5, 70.2, 41.0, 28.6, 24.3; ν_max_(KBr)/cm^−1^ 2980, 1617, 1248, 1039, 626; MS (EI) *m*/*z* 77, 91, 115, 142, 157, 192, 205, 219, 248, 291, 327, 342; HRMS-APCI (*m*/*z*): calcd for C_20_H_20_ClO_3_, [M + H]^+^: 343.1095, found 343.1091.

5-Chloro-6,6-dimethyl-2-(4-phenoxyphenyl)-4-phenyl-3,6-dihydro-2H-pyran (**4g**): Alkynol **1a** (0.20 mmol) with 1-phenoxy-4-vinylbenzene **2h** (0.30 mmol, 1.5 equiv.) gave compound **4g** (67%, 52.3 mg) as a yellow oil; ^1^H NMR (400 MHz, CDCl_3_) δ 7.48–7.42 (m, 4H), 7.41–7.33 (m, 5H), 7.16 (tt, *J* = 7.0, 1.1 Hz, 1H), 7.07 (ddd, *J* = 8.6, 2.7, 1.6 Hz, 4H), 5.00 (dd, *J* = 10.6, 3.1 Hz, 1H), 2.84 (dd, *J* = 16.9, 10.8 Hz, 1H), 2.65 (dd, *J* = 16.9, 3.1 Hz, 1H), 1.68 (s, 6H); ^13^C NMR (100 MHz, CDCl_3_) δ 157.3, 156.7, 139.9, 136.6, 133.5, 131.9, 129.8, 128.3, 128.2, 127.7, 127.5, 123.3, 119.0, 118.8, 77.5, 70.0, 41.0, 28.6, 24.3; ν_max_(KBr)/cm^−1^ 2980, 2032, 1592, 1488, 1240, 628; MS (EI) *m*/*z* 77, 91, 115, 142, 157, 192, 202, 245, 281, 297, 311, 339, 375, 390; HRMS-APCI (*m*/*z*): calcd for C_25_H_24_ClO_2_, [M + H]^+^: 391,1459, found 391.1465.

2-([1,1’-Biphenyl]-4-yl)-5-chloro-6,6-dimethyl-4-phenyl-3,6-dihydro-2H-pyran (**4h**): Alkynol **1a** (0.20 mmol) with 4-vinyl-1,1’-biphenyl **2i** (0.30 mmol, 1.5 equiv.) gave compound **4h** (69%, 51.6mg) as a gray solid, mp = 108.3–108.9 °C; ^1^H NMR (400 MHz, CDCl_3_) δ 7.65–7.60 (m, 4H), 7.56–7.52 (m, 2H), 7.47 (td, *J* = 6.7, 1.7 Hz, 2H), 7.44–7.31 (m, 6H), 5.03 (dd, *J* = 10.8, 3.1 Hz, 1H), 2.85 (dd, *J* = 16.8, 10.7 Hz, 1H), 2.66 (dd, *J* = 16.9, 3.1 Hz, 1H), 1.67 (d, *J* = 2.0 Hz, 6H);^13^C NMR (100 MHz, CDCl_3_) δ 141.0, 140.8, 140.7, 139.9, 133.6, 131.9, 128.8, 128.3, 128.2, 127.5, 127.3, 127.2, 126.6, 77.5, 70.2, 40.9, 28.6, 24.3; ν_max_(KBr)/cm^−1^ 2984, 2928, 2891, 1617, 1355, 1072, 624; MS (EI) *m*/*z* 77, 91, 115, 141, 157, 177, 192, 205, 265, 324, 359, 374; HRMS-APCI (*m*/*z*): calcd for C_25_H_24_ClO, [M + H]^+^: 375.1510, found 375.1499.

5-Chloro-2-(4-fluorophenyl)-6,6-dimethyl-4-phenyl-3,6-dihydro-2H-pyran (**4i**): Alkynol **1a** (0.20 mmol) with 1-fluoro-4-vinylbenzene **2j** (0.30 mmol, 1.5 equiv.) gave compound **4i** (64%, 40.2 mg) as a white solid, mp = 70.0–71.2 °C; ^1^H NMR (400 MHz, CDCl_3_) δ 7.44–7.36 (m, 4H), 7.35–7.28 (m, 3H), 7.09–7.03 (m, 2H), 4.94 (dd, *J* = 10.6, 3.1 Hz, 1H), 2.74 (dd, *J* = 16.9, 10.6 Hz, 1H), 2.58 (dd, *J* = 16.8, 3.2 Hz, 1H), 1.62 (d, *J* = 1.3 Hz, 6H); ^13^C NMR (100 MHz, CDCl_3_) δ 162.3 (d, *J* = 245.5 Hz), 139.8, 137.4 (d, *J* = 3.1 Hz), 133.5, 128.3, 128.1, 127.8 (d, *J* = 8.0 Hz), 127.5, 115.3 (d, *J* = 21.3 Hz), 77.5, 69.7, 41.0, 28.5, 24.3. ^19^F NMR (376 MHz, CDCl_3_) δ −114.8; ν_max_(KBr)/cm^−1^ 2984, 2902, 1510, 1394, 1225, 1156, 1080, 1051, 908, 834, 757; MS (EI) *m*/*z* 77, 95, 109, 123, 142, 157, 177, 192, 220, 237, 266, 281, 301, 316; HRMS-APCI (*m*/*z*): calcd for C_19_H_17_ClFO, [M-H]^−^: 315.0957, found 315.0961.

5-Chloro-2-(4-chlorophenyl)-6,6-dimethyl-4-phenyl-3,6-dihydro-2H-pyran (**4j**): Alkynol **1a** (0.20 mmol) with 1-chloro-4-vinylbenzene **2k** (0.30 mmol, 1.5 equiv.) gave compound **4j** (68%, 44.9 mg) as a white solid, mp = 68.1–69.2 °C; ^1^H NMR (400 MHz, CDCl_3_) δ 7.41–7.35 (m, 4H), 7.35–7.28 (m, 5H), 4.92 (dd, *J* = 10.6, 3.3 Hz, 1H), 2.71 (dd, *J* = 16.8, 10.6 Hz, 1H), 2.57 (dd, *J* = 16.9, 3.3 Hz, 1H), 1.60 (d, *J* = 3.3 Hz, 6H); ^13^C NMR (100 MHz, CDCl_3_) δ 140.2, 139.7, 133.5, 133.4, 131.6, 128.6, 128.3, 128.1, 127.5, 127.4, 77.5, 69.6, 40.8, 28.5, 24.3; ν_max_(KBr)/cm^−1^ 2981, 2902, 1391, 1227, 1080, 1051, 900, 723; MS (EI) *m*/*z* 77, 115, 125, 141, 157, 177, 192, 202, 282, 297, 317, 332; HRMS-APCI (*m*/*z*): calcd for C_19_H_17_Cl_2_O, [M − H]^+^: 331.0651, found 331.0646.

5-Chloro-2-(3-chlorophenyl)-6,6-dimethyl-4-phenyl-3,6-dihydro-2H-pyran (**4k**): Alkynol **1a** (0.20 mmol) with 1-chloro-3-vinylbenzene **2l** (0.30 mmol, 1.5 equiv.) gave compound **4k** (64%, 44.9 mg) as a white oil; ^1^H NMR (400 MHz, CDCl_3_) δ 7.47 (d, *J* = 1.8 Hz, 1H), 7.42–7.37 (m, 2H), 7.35–7.27 (m, 6H), 4.94 (dd, *J* = 10.5, 3.3 Hz, 1H), 2.73 (dd, *J* = 16.8, 10.6 Hz, 1H), 2.60 (dd, *J* = 16.8, 3.3 Hz, 1H), 1.63 (d, *J* = 8.1 Hz, 6H);^13^C NMR (100 MHz, CDCl_3_) δ 143.7, 139.7, 134.4, 133.5, 131.6, 129.8, 128.3, 128.1, 127.8, 127.6, 126.3, 124.2, 77.5, 69.6, 40.8, 28.5, 24.3; ν_max_(KBr)/cm^−1^ 2984, 2902, 1394, 1225, 1051; MS (EI) *m*/*z* 77, 115, 157, 192, 218, 253, 281, 297, 317, 332; HRMS-APCI (*m*/*z*): calcd for C_19_H_19_Cl_2_O, [M + H]^+^: 333.0807, found 333.0793.

5-Chloro-2-(2-chlorophenyl)-6,6-dimethyl-4-phenyl-3,6-dihydro-2H-pyran (**4l**): Alkynol **1a** (0.20 mmol) with 1-chloro-2-vinylbenzene **2m** (0.30 mmol, 1.5 equiv.) gave compound **4l** (32%, 21.2mg) as a yellow oil; ^1^H NMR (400 MHz, CDCl_3_) δ 7.69 (d, *J* = 7.8 Hz, 1H), 7.37 (p, *J* = 7.8 Hz, 7H), 7.29–7.24 (m, 1H), 5.35 (dd, *J* = 10.6, 3.2 Hz, 1H), 2.79 (dd, *J* = 16.9, 3.3 Hz, 1H), 2.59 (dd, *J* = 16.9, 10.5 Hz, 1H), 1.65 (d, *J* = 4.6 Hz, 6H); ^13^C NMR (100 MHz, CDCl_3_) δ 139.7, 139.3, 133.2, 131.9, 131.8, 129.3, 128.7, 128.2, 127.5, 127.4, 127.3, 77.6, 67.2, 39.4, 28.5, 24.3; ν_max_(KBr)/cm^−1^ 2981, 2902, 1394, 1230, 1053, 752, 702; MS (EI) *m*/*z* 77, 115, 157, 192, 218, 253, 281, 297, 317, 332; HRMS-APCI (*m*/*z*): calcd for C_19_H_19_Cl_2_O, [M + H]^+^: 333.0807, found 333.0796.

4-Bromo-4’-chloro-5’,5’-dimethyl-1’,2’,5’,6’-tetrahydro-1,1’:3’,1’’-terphenyl (**4m**): Alkynol **1a** (0.20 mmol) with 1-bromo-4-vinylbenzene **2n** (0.30 mmol, 1.5 equiv.) gave compound **4m** (77%, 57.6 mg) as a white solid, mp = 77.2–78.2 °C; ^1^H NMR (400 MHz, CDCl_3_) δ 7.50 (d, *J* = 8.5 Hz, 2H), 7.39 (t, *J* = 7.5 Hz, 2H), 7.36–7.29 (m, 5H), 4.92 (dd, *J* = 10.5, 3.4 Hz, 1H), 2.72 (dd, *J* = 16.8, 10.6 Hz, 1H), 2.58 (dd, *J* = 16.9, 3.4 Hz, 1H), 1.62 (d, *J* = 4.8 Hz, 6H); ^13^C NMR (100 MHz, CDCl_3_) δ 140.7, 139.7, 133.5, 131.6, 131.6, 128.3, 128.1, 127.8, 127.6, 121.5, 77.5, 69.7, 40.8, 28.5, 24.3; ν_max_(KBr)/cm^−1^ 2989, 2226, 1513, 1243, 1167, 1022, 961, 823, 755, 694; MS (EI) *m*/*z* 77, 115, 142, 157, 177, 192, 202, 229, 341, 362, 376; HRMS-APCI (*m*/*z*): calcd for C_19_H_17_BrCl_1_O, [M-H]^+^: 375.0146, found 375.0146.

4-(5-Chloro-6,6-dimethyl-4-phenyl-3,6-dihydro-2H-pyran-2-yl)phenyl acetate (**4n**): Alkynol **1a** (0.20 mmol) with 4-vinylphenyl acetate **2o** (0.30 mmol, 1.5 equiv.) gave compound **4n** (68%, 48.4 mg) as a white oil; ^1^H NMR (400 MHz, CDCl_3_) δ 7.45 (d, *J* = 8.5 Hz, 2H), 7.38 (t, *J* = 7.3 Hz, 2H), 7.34–7.29 (m, 3H), 7.09 (d, *J* = 8.6 Hz, 2H), 4.95 (dd, *J* = 10.7, 3.3 Hz, 1H), 2.75 (dd, *J* = 16.8, 10.7 Hz, 1H), 2.59 (dd, *J* = 16.9, 3.3 Hz, 1H), 2.30 (s, 3H), 1.61 (d, *J* = 3.3 Hz, 6H); ^13^C NMR (100 MHz, CDCl_3_) δ 169.5, 150.1, 139.8, 139.3, 133.5, 131.7, 128.3, 128.1, 127.5, 127.2, 121.6, 77.4, 69.8, 40.9, 28.5, 24.3, 21.2; ν_max_(KBr)/cm^−1^ 2976, 2868, 1759, 1592, 1488, 1367, 1190, 1163, 1082, 909, 855, 761, 698; MS (EI) *m*/*z* 77, 91, 107, 115, 142, 157, 192, 205, 235, 263, 299, 341, 356; HRMS-APCI (*m*/*z*): calcd for C_21_H_22_ClO_3_, [M + H]^+^: 357.1252, found 357.1247.

5-Chloro-6,6-dimethyl-4-phenyl-2-(4-(trifluoromethyl)phenyl)-3,6-dihydro-2H-pyran (**4o**): Alkynol **1a** (0.20 mmol) with 1-(trifluoromethyl)-4-vinylbenzene **2p** (0.30 mmol, 1.5 equiv.) gave compound **4o** (59%, 42.9 mg) as a yellow oil; ^1^H NMR (400 MHz, CDCl_3_) δ 7.64 (d, *J* = 8.3 Hz, 2H), 7.57 (d, *J* = 8.3 Hz, 2H), 7.39 (d, *J* = 7.3 Hz, 2H), 7.33 (d, *J* = 7.4 Hz, 3H), 5.02 (dd, *J* = 10.4, 3.6 Hz, 1H), 2.73 (dd, *J* = 16.9, 10.5 Hz, 1H), 2.63 (dd, *J* = 16.8, 3.6 Hz, 1H), 1.63 (d, *J* = 7.5 Hz, 6H); ^13^C NMR (100 MHz, CDCl_3_) δ 145.6, 139.6, 133.5, 131.5, 129.9 (q, *J* = 32.3 Hz), 128.3, 128.1, 127.6, 126.3, 125.4 (q, *J* = 3.8 Hz), 124.2 (q, *J* = 271.0 Hz), 77.5, 69.7, 40.8, 28.5, 24.3; ^19^F NMR (376 MHz, CDCl_3_) δ −62.5; ν_max_(KBr)/cm^−1^ 2929, 1622, 1492, 1413, 1322, 1163, 1065, 1017, 965, 850, 830, 760, 697; MS (EI) *m*/*z* 77, 91, 115, 142, 157, 177, 192, 202, 233, 287, 331, 351, 366; HRMS-APCI (*m*/*z*): calcd for C_20_H_17_ClF_3_O, [M − H]^+^: 365.0915, found 365.0909.

4-(5-Chloro-6,6-dimethyl-4-phenyl-3,6-dihydro-2H-pyran-2-yl)benzonitrile (**4p**): Alkynol **1a** (0.20 mmol) with 4-vinylbenzonitrile **2q** (0.30 mmol, 1.5 equiv.) gave compound **4p** (58%, 37.5 mg) as a white solid, mp = 73.5–74.2 °C; ^1^H NMR (400 MHz, CDCl_3_) δ 7.68 (d, *J* = 8.4 Hz, 2H), 7.57 (d, *J* = 8.4 Hz, 2H), 7.44–7.37 (m, 2H), 7.36–7.30 (m, 3H), 5.03 (dd, *J* = 9.8, 4.3 Hz, 1H), 2.73–2.61 (m, 2H), 1.64 (d, *J* = 9.5 Hz, 6H); ^13^C NMR (100 MHz, CDCl_3_) δ 147.0, 139.5, 133.5, 132.3, 131.3, 128.3, 128.1, 127.7, 126.6, 118.9, 111.4, 77.6, 69.5, 40.7, 28.5, 24.3; ν_max_(KBr)/cm^−1^ 2926, 2223, 1365, 1169, 1082, 1019, 963, 834, 760, 697, 559; MS (EI) *m*/*z* 77, 102, 116, 157, 192, 230, 266, 288, 308, 323; HRMS-APCI (*m*/*z*): calcd for C_20_H_19_ClNO, [M + H]^+^: 324.1150, found 324.1147.

5-Chloro-6,6-dimethyl-2-(naphthalen-2-yl)-4-phenyl-3,6-dihydro-2H-pyran (**4q**): Alkynol **1a** (0.20 mmol) with 2-vinylnaphthalene **2r** (0.30 mmol, 1.5 equiv.) gave compound **4q** (63%, 43.8 mg) as a white solid, mp = 104.0–105.2 °C; ^1^H NMR (400 MHz, CDCl_3_) δ 7.95 (s, 1H), 7.93–7.87 (m, 3H), 7.62 (dd, *J* = 8.5, 1.9 Hz, 1H), 7.53 (dt, *J* = 6.0, 2.6 Hz, 2H), 7.46–7.35 (m, 5H), 5.18 (dd, *J* = 10.7, 3.2 Hz, 1H), 2.93 (dd, *J* = 16.8, 10.7 Hz, 1H), 2.75 (dd, *J* = 16.9, 3.3 Hz, 1H), 1.73 (d, *J* = 4.1 Hz, 6H); ^13^C NMR (100 MHz, CDCl_3_) δ 140.0, 139.0, 133.6, 133.4, 133.1, 131.9, 128.3, 128.2, 128.1, 127.7, 127.5, 126.2, 125.9, 124.8, 124.3, 77.5, 70.4, 40.9, 28.6, 24.4; ν_max_(KBr)/cm^−1^ 2928, 1362, 1161, 1072, 1029, 958, 821, 763, 699, 567; MS (EI) *m*/*z* 77, 91, 127, 141, 157, 192, 205, 239, 252, 269, 297, 333, 348; HRMS-APCI (*m*/*z*): calcd for C_23_H_22_ClO, [M + H]^+^: 349.1354, found 349.1353.

5-Chloro-6,6-dimethyl-4-phenyl-2-(thiophen-2-yl)-3,6-dihydro-2H-pyran (**4r**): Alkynol **1a** (0.20 mmol) with 2-vinylthiophene **2s** (0.30 mmol, 1.5 equiv.) gave compound **4r** (68%, 41.3 mg) as a white solid, mp = 58.2–58.9 °C; ^1^H NMR (400 MHz, CDCl_3_) δ 7.42–7.37 (m, 2H), 7.33 (d, *J* = 7.4 Hz, 3H), 7.28 (dd, *J* = 5.0, 1.3 Hz, 1H), 7.06–6.97 (m, 2H), 5.21 (dd, *J* = 10.6, 3.1 Hz, 1H), 2.94 (dd, *J* = 16.8, 10.6 Hz, 1H), 2.70 (dd, *J* = 16.8, 3.1 Hz, 1H), 1.62 (d, *J* = 10.1 Hz, 6H); ^13^C NMR (100 MHz, CDCl_3_) δ 144.8, 139.7, 133.5, 131.5, 128.3, 128.2, 127.6, 126.6, 125.0, 124.1, 77.8, 66.8, 40.9, 28.5, 24.3; ν_max_(KBr)/cm^−1^ 2931, 1656, 1167, 1072, 1014, 963, 905, 757, 694; MS (EI) *m*/*z* 77, 97, 115, 142, 157, 192, 205, 225, 253, 269, 289, 304; HRMS-APCI (*m*/*z*): calcd for C_17_H_18_ClOS, [M + H]^+^: 305.0761, found 305.0755.

4-(5-Chloro-6,6-dimethyl-2-phenyl-3,6-dihydro-2H-pyran-4-yl)phenyl 2-(4-isobutylphenyl)propanoate (**5a**): Alkynol **1a** (0.20 mmol) with styrene derivative **2t** (0.30 mmol, 1.5 equiv.) gave compound **5a** (70%, 70.4 mg) as a colorless oil; ^1^H NMR (400 MHz, CDCl_3_) δ 7.45 (dd, *J* = 8.4, 1.4 Hz, 2H), 7.42–7.36 (m, 2H), 7.32 (dt, *J* = 8.6, 6.4 Hz, 5H), 7.20–7.16 (m, 2H), 7.07–7.02 (m, 2H), 4.95 (dd, *J* = 10.6, 3.1 Hz, 1H), 3.97 (q, *J* = 7.1 Hz, 1H), 2.76 (dd, *J* = 16.9, 10.6 Hz, 1H), 2.58 (dd, *J* = 16.9, 3.3 Hz, 1H), 2.51 (d, *J* = 7.1 Hz, 2H), 1.91 (dp, *J* = 13.6, 6.8 Hz, 1H), 1.67–1.60 (m, 9H), 0.95 (d, *J* = 6.6 Hz, 6H); ^13^C NMR (100 MHz, CDCl_3_) δ 173.2, 150.0, 141.6, 140.9, 137.3, 137.2, 133.9, 131.1, 129.6, 129.3, 128.5, 127.8, 127.3, 126.1, 121.2, 77.4, 70.3, 45.3, 45.1, 40.8, 30.2, 28.6, 24.3, 22.5, 18.5; ν_max_(KBr)/cm^−1^ 3068, 2984, 2902, 1391, 1080, 1053, 752, 702;HRMS-APCI (*m*/*z*): calcd for C_32_H_36_ClO_3_, [M + H]^+^: 503.2347, found 503.2343.

(2E,4E)-Deca-2,4-dien-1-yl 4-(5-chloro-6,6-dimethyl-2-phenyl-3,6-dihydro-2H-pyran-4-yl)benzoate (**5b**): Alkynol **1a** (0.20 mmol) with styrene derivative **2u** (0.30 mmol, 1.5 equiv.) gave compound **5b** (74%, 70.7 mg) as a colorless oil; ^1^H NMR (400 MHz, CDCl_3_) δ 8.11–8.04 (m, 2H), 7.48–7.36 (m, 6H), 7.34–7.28 (m, 1H), 5.53–5.46 (m, 1H), 5.12 (tt, *J* = 6.8, 1.4 Hz, 1H), 4.97 (dd, *J* = 10.6, 3.1 Hz, 1H), 4.87 (d, *J* = 7.0 Hz, 2H), 2.78 (dd, *J* = 16.9, 10.6 Hz, 1H), 2.59 (dd, *J* = 16.8, 3.2 Hz, 1H), 2.12 (tq, *J* = 9.4, 4.7 Hz, 4H), 1.79 (d, *J* = 1.4 Hz, 3H), 1.70 (d, *J* = 1.4 Hz, 3H), 1.63 (d, *J* = 2.8 Hz, 8H), 1.38–1.28 (m, 1H); ^13^C NMR (100 MHz, CDCl_3_) δ 166.3, 144.4, 142.4, 141.4, 134.5, 131.9, 131.3, 129.6, 129.6, 128.5, 128.2, 127.8, 126.1, 123.8, 118.5, 77.4, 70.3, 62.0, 40.6, 39.6, 28.5, 26.4, 25.7, 24.3, 17.8, 16.6; ν_max_(KBr)/cm^−1^ 3064, 2984, 2899, 1391, 1230, 1082, 1051, 755, 697; HRMS-APCI (*m*/*z*): calcd for C_30_H_36_ClO_3_, [M + H]^+^: 479.2347, found 479.2341.

(1S,2R,5S)-2-Isopropyl-5-methylcyclohexyl 4-(5-chloro-6,6-dimethyl-2-phenyl-3,6-dihydro-2H-pyran-4-yl)benzoate (**5c**): Alkynol **1a** (0.20 mmol) with styrene derivative **2v** (0.30 mmol, 1.5 equiv.) gave compound **5c** (68%, 65.2 mg) as a colorless oil; ^1^H NMR (400 MHz, CDCl_3_) δ 8.07 (d, *J* = 8.3 Hz, 2H), 7.53–7.27 (m, 7H), 4.96 (ddd, *J* = 10.8, 6.9, 3.8 Hz, 2H), 2.78 (ddd, *J* = 16.9, 10.7, 2.5 Hz, 1H), 2.62–2.53 (m, 1H), 2.15 (dd, *J* = 10.4, 6.0 Hz, 1H), 1.99 (pd, *J* = 7.0, 2.8 Hz, 1H), 1.75 (dd, *J* = 14.2, 3.1 Hz, 2H), 1.66–1.54 (m, 8H), 1.20–1.07 (m, 2H), 0.94 (dd, *J* = 6.8, 3.2 Hz, 7H), 0.82 (d, *J* = 7.0 Hz, 3H); ^13^C NMR (100 MHz, CDCl_3_) δ 165.7, 144.4, 141.4, 134.5, 131.3, 131.3, 129.9, 129.6, 128.5, 128.2, 127.8, 126.1, 126.0, 77.4, 74.9, 70.2, 47.3, 41.0, 40.7, 40.6, 34.4, 31.5, 28.5, 26.5, 24.3, 23.7, 22.1, 20.8, 16.6; ν_max_(KBr)/cm^−1^ 3056, 2981, 2897, 1705, 1407, 1262, 1051, 734; HRMS-APCI (*m*/*z*): calcd for C_30_H_38_ClO_3_, [M + H]^+^: 481.2504, found 481.2494.

(3S,8S,9S,10R,13R,14S,17R)-10,13-dimethyl-17-((R)-6-methylheptan-2-yl)-–2,3,4,7,8,9,10,11,12,13,14,15,16,17-tetradecahydro-1H-cyclopenta[a]phenanthren-3-yl 4-(5-chloro-6,6-dimethyl-2-phenyl-3,6-dihydro-2H-pyran-4-yl)benzoate (**5d**): Alkynol **1a** (0.20 mmol) with styrene derivative **2w** (0.30 mmol, 1.5 equiv.) gave compound **5d** (40%, 56.8 mg) as a yellow solid, mp = 158.6–159.2 °C; ^1^H NMR (400 MHz, CDCl_3_) δ 8.08–8.02 (m, 2H), 7.43 (d, *J* = 7.2 Hz, 2H), 7.41–7.31 (m, 5H), 5.45–5.41 (m, 1H), 4.95 (dd, *J* = 10.6, 3.1 Hz, 1H), 4.87 (dtd, *J* = 12.4, 8.3, 4.5 Hz, 1H), 2.77 (dd, *J* = 16.8, 10.7 Hz, 1H), 2.57 (dd, *J* = 16.9, 3.1 Hz, 1H), 2.47 (d, *J* = 8.0 Hz, 2H), 2.06–2.00 (m, 2H), 1.98 (d, *J* = 4.7 Hz, 1H), 1.92 (dt, *J* = 13.6, 3.4 Hz, 1H), 1.78–1.68 (m, 2H), 1.61 (s, 6H), 1.56–1.50 (m, 3H), 1.47 (d, *J* = 4.6 Hz, 1H), 1.35 (d, *J* = 7.6 Hz, 3H), 1.28 (dd, *J* = 11.0, 3.1 Hz, 2H), 1.23–1.18 (m, 2H), 1.17–1.10 (m, 4H), 1.08 (s, 3H), 1.06–0.99 (m, 3H), 0.93 (d, *J* = 6.5 Hz, 3H), 0.88 (dd, *J* = 6.6, 1.8 Hz, 6H), 0.70 (s, 3H); ^13^C NMR (100 MHz, CDCl_3_) δ 165.7, 144.3, 141.4, 139.7, 134.5, 131.3, 129.9, 129.6, 129.5, 128.5, 128.2, 127.8, 126.0, 122.8, 77.4, 74.7, 70.2, 56.7, 56.2, 50.1, 42.4, 40.6, 39.8, 39.6, 38.3, 37.1, 36.7, 36.2, 35.8, 32.0, 31.9, 28.5, 28.3, 28.0, 27.9, 24.3, 24.3, 23.9, 22.9, 22.6, 21.1, 19.4, 18.8, 11.9; ν_max_(KBr)/cm^−1^ 2944, 1713, 1267, 768, 691; HRMS-APCI (*m*/*z*): calcd for C_47_H_64_ClO_3_, [M + H]^+^: 711.4539, found 711.4536.

(E)-3,7-Dimethylocta-2,6-dien-1-yl 4-(5-chloro-6,6-dimethyl-2-phenyl-3,6-dihydro-2H-pyran-4-yl)benzoate (**5e**): Alkynol **1a** (0.20 mmol) with styrene derivative **2x** (0.30 mmol, 1.5 equiv.) gave compound **5e** (74%, 70.6 mg) as a colorless oil; ^1^H NMR (400 MHz, CDCl_3_) δ 8.08 (d, *J* = 8.5 Hz, 2H), 7.47–7.36 (m, 6H), 7.34–7.28 (m, 1H), 5.50 (tt, *J* = 7.1, 1.3 Hz, 1H), 5.16–5.10 (m, 1H), 4.97 (dd, *J* = 10.6, 3.1 Hz, 1H), 4.87 (d, *J* = 7.1 Hz, 2H), 2.79 (dd, *J* = 16.9, 10.6 Hz, 1H), 2.59 (dd, *J* = 16.9, 3.1 Hz, 1H), 2.19–2.07 (m, 4H), 1.80 (d, *J* = 1.4 Hz, 3H), 1.71 (d, *J* = 1.3 Hz, 3H), 1.64 (d, *J* = 3.1 Hz, 9H); ^13^C NMR (100 MHz, CDCl_3_) δ 166.3, 144.4, 142.4, 141.4, 134.5, 131.9, 131.3, 129.6, 129.6, 128.6, 128.3, 127.8, 126.1, 123.8, 118.5, 77.4, 70.3, 62.0, 40.6, 39.6, 28.5, 26.4, 25.8, 24.3, 17.8, 16.6; ν_max_(KBr)/cm^−1^ 3076, 2986, 2902, 1391, 1259, 1061, 755, 697; HRMS-APCI (*m*/*z*): calcd for C_30_H_36_ClO_3_, [M + H]^+^: 479.2347, found 479.2342.

4-(5-chloro-6,6-dimethyl-2-phenyl-3,6-dihydro-2H-pyran-4-yl)phenyl (S)-2-(6-methoxynaphthalen-2-yl)propanoate (**5f**): Alkynol **1a** (0.20 mmol) with styrene derivative **2y** (0.30 mmol, 1.5 equiv.) gave compound **5f** (65%, 68.4 mg) as a colorless oil; ^1^H NMR (400 MHz, CDCl_3_) δ 7.84–7.77 (m, 3H), 7.56 (dd, *J* = 8.4, 1.9 Hz, 1H), 7.46 (d, *J* = 7.2 Hz, 2H), 7.41 (t, *J* = 7.5 Hz, 2H), 7.36–7.31 (m, 3H), 7.24–7.18 (m, 2H), 7.09–7.02 (m, 2H), 4.96 (dd, *J* = 10.7, 3.1 Hz, 1H), 4.15 (q, *J* = 7.1 Hz, 1H), 3.96 (s, 3H), 2.77 (dd, *J* = 16.9, 10.7 Hz, 1H), 2.58 (dd, *J* = 16.9, 3.1 Hz, 1H), 1.75 (d, *J* = 7.1 Hz, 3H), 1.64 (d, *J* = 3.1 Hz, 6H); ^13^C NMR (100 MHz, CDCl_3_) δ 173.2, 157.8, 150.0, 141.5, 137.3, 135.1, 133.9, 133.9, 131.1, 129.4, 129.3, 129.0, 128.5, 127.8, 127.5, 126.2, 126.2, 126.1, 121.2, 119.2, 105.7, 77.4, 70.3, 55.4, 45.6, 40.8, 28.5, 24.3, 18.6; ν_max_(KBr)/cm^−1^ 2980, 1754, 1605, 1167, 1070, 541; HRMS-APCI (*m*/*z*): calcd for C_33_H_32_ClO_4_, [M + H]^+^: 527.1984, found 527.1978.

## 4. Conclusions

In conclusion, we have established a robust and novel palladium-catalyzed intermolecular carboetherification of alkenes with alkynols under aerobic oxidative conditions for accessing structurally diverse 3,6-dihydro-2*H*-pyrans. To the best of our knowledge, this catalytic system presented the first example in which the aryl alkenes can directly couple with alkynols through the formation of new C-Cl, C-C and, C-O bonds. Under the optimized conditions, an array of functional groups such as halogen group, ester, nitrile, aldehyde, phenoxy, aromatic heterocycles, etc., were suitable in the developed synthetic strategy with moderate to good yields and good regioselectivities. Of particular significance is that the 1-(phenylethynyl)cyclopentan-1-ol and 1-(phenylethynyl)- cyclohexan-1-ol could also be compatible with the developed catalytic system. More importantly, this catalytic approach is further verified by gram-scale experiment and the late-stage derivatization of pharmaceuticals and biologically active molecules. Studies of asymmetric synthesis for the rapid assembly of chirality 3,6-dihydro-2*H*-pyrans is ongoing in our laboratory.

## Figures and Tables

**Table 1:** Optimization of the reaction conditions ^a^.

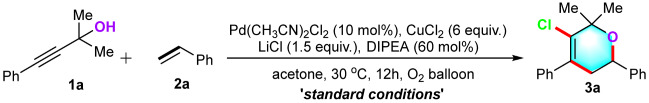		
Entry	Variation from Standard Conditions	Yield ^b^
1	none	78 (72)
2	PdCl_2_ instead of Pd(CH_3_CN)_2_Cl_2_	41
3	Pd(CH_3_CN)_2_(BF_4_)_2_ instead of Pd(CH_3_CN)_2_Cl_2_	56
4	Pd(PhCN)_2_Cl_2_ instead of Pd(CH_3_CN)_2_Cl_2_	47
5	Pd(COD)_2_Cl_2_ or Pd(dppp)_2_Cl_2_ instead of Pd(CH_3_CN)_2_Cl_2_	trace
6	CH_3_CN or HOAc instead of acetone	trace
7	DMSO or DMF instead of acetone	N.D.
8	DCE/THF instead of acetone	36/28
9	*^t^*BuOK/TMG instead of DIPEA	68/68
10	Cs_2_CO_3_/DBU/DABCO instead of DIPEA	51/65/43
11	MgCl_2_ instead of LiCl	54
12	LiCl (1 equiv.)/LiCl (2 equiv.)	70/78
13	40 °C instead of 30 °C	72
14	Without Pd(CH_3_CN)_2_Cl_2_	N.D.
15	Without CuCl_2_	N.D.

^a^ Conditions: All reactions were performed with **1a** (0.10 mmol), **2a** (0.15 mmol), Pd(CH_3_CN)_2_Cl_2_ (10 mol%), CuCl_2_ (6 equiv.), LiCl (1.5 equiv.), DIPEA (60 mol%), acetone (1 mL) at 30°C for 12 h. N.D. = not detected. ^b^ Determined by GC-MS using dodecane as the internal standard. The value in parentheses is the yield of the isolated product.

## Data Availability

Data are contained within the article and Supplementary Materials.

## Supplementary Materials

The following supporting information can be downloaded at https://www.mdpi.com/article/10.3390/molecules31111778/s1, X-ray Crystallographic analysis of **3k** and **4d**; the NMR spectra, IR spectra, and HR-MS spectra of the desired products.
