# Supplementary material for: Facile Assembly of Structurally Diverse 2H-Pyrans Enabled by Chloropalladation-Initiated Carboetherification of Alkenes
Source: Molecules. 2026 May 22;31(11):1778. doi: 10.3390/molecules31111778 (PMC13257514; doi:10.3390/molecules31111778)

checkCIF/PLATON report

You have not supplied any structure factors. As a result the full set of tests cannot be run.

THIS REPORT IS FOR GUIDANCE ONLY. IF USED AS PART OF A REVIEW PROCEDURE FOR PUBLICATION, IT SHOULD NOT REPLACE THE EXPERTISE OF AN EXPERIENCED CRYSTALLOGRAPHIC REFEREE.

No syntax errors found. CIF dictionary Interpreting this report

Datablock: mfh4a_0m_4

```
Bond precision:   C-C = 0.0064 A          Wavelength=0.71073

Cell:           a=10.1041(18)     b=13.308(2)      c=16.926(3)
                alpha=71.017(6)   beta=76.649(6)   gamma=72.312(6)
Temperature:    170 K
```

	Calculated	Reported
Volume	2028.3(6)	2028.2(6)
Space group	P -1	P-1
Hall group	-P 1	-P 1
Moiety formula	C23 H27 Cl O	C23 H27 Cl O
Sum formula	C23 H27 Cl O	C23 H27 Cl O
Mr	354.90	354.89
Dx,g cm-3	1.162	1.162
Z	4	4
Mu (mm-1)	0.196	0.196
F000	760.0	760.0
F000’	760.86	
h,k,lmax	12,16,21	0,0,0
Nref	8398	8207
Tmin,Tmax	0.986,0.990	0.515,0.745
Tmin’	0.977	

```
Correction method= # Reported T Limits: Tmin=0.515 Tmax=0.745
AbsCorr = MULTI-SCAN

Data completeness= 0.977          Theta(max)= 26.473

R(reflections)= 0.0876( 4953)            wR2(reflections)= 0.2239( 8207)

S = 1.062                 Npar= 461
```

The following ALERTS were generated. Each ALERT has the format
test-name_ALERT_alert-type_alert-level.
Click on the hyperlinks for more details of the test.

Alert level C

```
PLAT213_ALERT_2_C Atom C37             has ADP max/min Ratio .....         3.1 prolat
PLAT220_ALERT_2_C NonSolvent   Resd 2  C   Ueq(max)/Ueq(min) Range         3.9 Ratio
PLAT222_ALERT_3_C NonSolvent Resd 2  H   Uiso(max)/Uiso(min) Range         4.7 Ratio
PLAT242_ALERT_2_C Low    'MainMol' Ueq as Compared to Neighbors of          C1 Check
PLAT242_ALERT_2_C Low    'MainMol' Ueq as Compared to Neighbors of         C35 Check
PLAT340_ALERT_3_C Low Bond Precision on  C-C Bonds ..............     0.00635 Ang.
```

Alert level G

```
PLAT012_ALERT_1_G No       _shelx_res_checksum Found in CIF ......      Please Check
PLAT154_ALERT_1_G The s.u.'s on the Cell Angles are Equal ..(Note)       0.006 Degree
PLAT793_ALERT_4_G Model has Chirality at C8          (Centro SpGr)           S Verify
PLAT793_ALERT_4_G Model has Chirality at C28         (Centro SpGr)           S Verify
PLAT881_ALERT_1_G No Datum for _diffrn_reflns_av_R_equivalents ...      Please Do !
```

0 **ALERT level A** = Most likely a serious problem - resolve or explain
0 **ALERT level B** = A potentially serious problem, consider carefully
6 **ALERT level C** = Check. Ensure it is not caused by an omission or oversight
5 **ALERT level G** = General information/check it is not something unexpected

3 ALERT type 1 CIF construction/syntax error, inconsistent or missing data
4 ALERT type 2 Indicator that the structure model may be wrong or deficient
2 ALERT type 3 Indicator that the structure quality may be low
2 ALERT type 4 Improvement, methodology, query or suggestion
0 ALERT type 5 Informative message, check

It is advisable to attempt to resolve as many as possible of the alerts in all categories. Often the minor alerts point to easily fixed oversights, errors and omissions in your CIF or refinement strategy, so attention to these fine details can be worthwhile. In order to resolve some of the more serious problems it may be necessary to carry out additional measurements or structure refinements. However, the purpose of your study may justify the reported deviations and the more serious of these should normally be commented upon in the discussion or experimental section of a paper or in the "special_details" fields of the CIF. checkCIF was carefully designed to identify outliers and unusual parameters, but every test has its limitations and alerts that are not important in a particular case may appear. Conversely, the absence of alerts does not guarantee there are no aspects of the results needing attention. It is up to the individual to critically assess their own results and, if necessary, seek expert advice.

Publication of your CIF in IUCr journals

A basic structural check has been run on your CIF. These basic checks will be run on all CIFs submitted for publication in IUCr journals (*Acta Crystallographica*, *Journal of Applied Crystallography*, *Journal of Synchrotron Radiation*); however, if you intend to submit to *Acta Crystallographica Section C* or *E* or *IUCrData*, you should make sure that full publication checks are run on the final version of your CIF prior to submission.

Publication of your CIF in other journals

Please refer to the *Notes for Authors* of the relevant journal for any special instructions relating to CIF submission.

PLATON version of 04/06/2025; check.def file version of 30/05/2025

Datablock mfh4a_0m_4 - ellipsoid plot

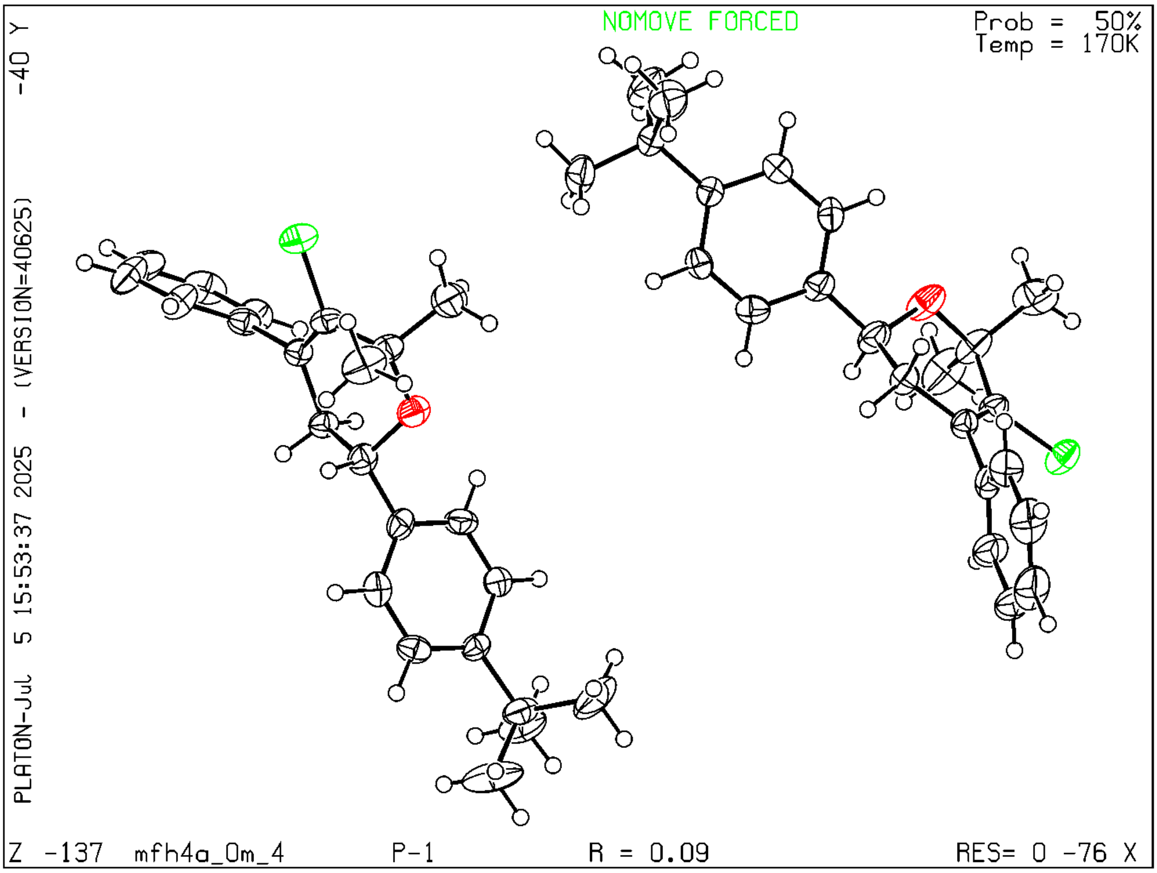

Supplement: Supplementary file 1 [file molecules-31-01778-s001.zip › checkcif-4d.pdf]
