# Supplementary material for: Facile Assembly of Structurally Diverse 2H-Pyrans Enabled by Chloropalladation-Initiated Carboetherification of Alkenes
Source: Molecules. 2026 May 22;31(11):1778. doi: 10.3390/molecules31111778 (PMC13257514; doi:10.3390/molecules31111778)
Supplement: Supplementary file 1 [file molecules-31-01778-s001.zip › molecules-4335398-supplementary resubmit.pdf]

Supporting Information

Facile Assembly of Structurally Diverse 2H-Pyrans Enabled by Chloropalladation-Initiated Carboetherification of Alkenes

Fanghua Mao[1], Bowen Wang[1], Zhengwang Chen[2*], Yin-Long Lai[3*], Huanfeng Jiang[1], Jianxiao Li[1, 3*]

[1] Key Lab of Functional Molecular Engineering of Guangdong Province, School of Chemistry and Chemical Engineering, South China University of Technology, Guangzhou 510640, P. R. China

[2] Jiangxi Provincial Key Laboratory of Synthetic Pharmaceutical Chemistry, Gannan Normal University, Ganzhou 341000, China

[3] College of Chemistry and Civil Engineering, Shaoguan University, Shaoguan 512005, China

* Correspondence: chenzwang2021@163.com (Z.C.); chemlaiyinlong@163.com (Y.-L.L.); cejxli@scut.edu.cn (J.L.)

Table of Contents

X-ray Crystallographic analysis....S2

NMR Spectra for all the compounds....S4

IR Spectra for all the compounds....S52

HRMS Spectra for all the compounds S74

X-ray Crystallographic analysis for product 3k

The CCDC number of the compound **3k** is 2534149.

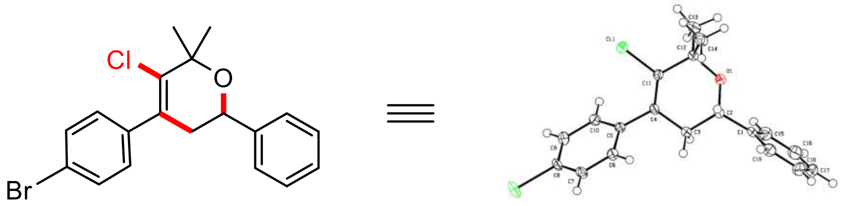

Crystal Data and Structure Refinement for Product **3k**

Empirical formula	$C_{19}H_{18}BrClO$
Formula weight	377.69
Temperature	170 K
Wavelength	0.71073 Å
Crystal system	triclinic
Space group	P-1
Unit cell dimensions	a = 5.8690(2) Å, α= 104.8380(10)°
	b= 10.1522(4) Å, β= 94.280(2)°
	c= 14.6162(6) Å, γ= 96.306(2)°
Density (calculated)	1.508
Absorption coefficient	0.083
F(000)	384.0
Crystal size(mm³)	0.15 × 0.06 × 0.05
Theta range for data collection	4.188 to 52.852
Index ranges	$-7 \leqslant h \leqslant 7, -12 \leqslant k \leqslant 12, -18 \leqslant l \leqslant 18$
Reflections collected	10135
Independent reflections	3421
Completeness to theta = 29.55°	98.03%
Absorption correction	multi-scan
Refinement method	Full-matrix least-squares on F^2
Data/restraints/parameters	3421/0/201
Goodness-of-fit on F^2	1.031
Final R indices [I>2sigma(I)]	$R1 = 0.0362, wR_2 = 0.0857$
R indices (all data)	$R1 = 0.0452, wR_2 = 0.0910$

X-ray Crystallographic analysis for product 4d

The CCDC number of the compound **4d** is 2534152.

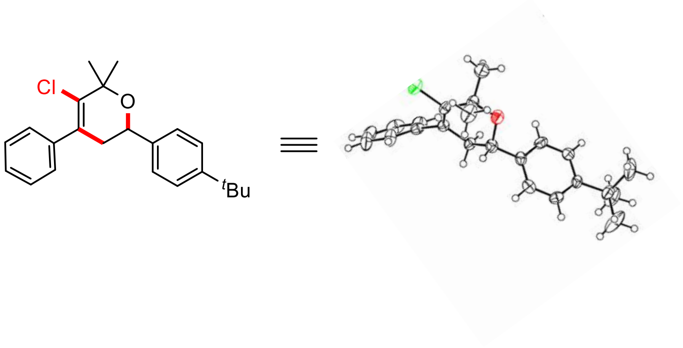

Crystal Data and Structure Refinement for Product **4d**

Empirical formula	$C_{23}H_{27}ClO$
Formula weight	354.89
Temperature	170 K
Wavelength	0.71073 Å
Crystal system	triclinic
Space group	P-1
Unit cell dimensions	a = 10.1041(18) Å, α= 71.017(6)°
	b= 13.308(2) Å, β= 76.649(6)°
	c= 16.926(3) Å, γ= 72.312(6)°
Density (calculated)	1.162
Absorption coefficient	0.083
F(000)	760.0
Crystal size	0.12 × 0.06 × 0.05
Theta range for data collection	4.658 to 52.946
Index ranges	$-12 \leq h \leq 12$, $-16 \leq k \leq 16$, $-21 \leq l \leq 21$
Reflections collected	8207
Independent reflections	8207
Completeness to theta = 29.55°	98.20%
Absorption correction	multi-scan
Refinement method	Full-matrix least-squares on F^2
Data/restraints/parameters	8207/0/461
Goodness-of-fit on F^2	1.062
Final R indices [I>2sigma(I)]	R1 = 0.0876, wR_2 = 0.1948
R indices (all data)	R1 = 0.1565, wR_2 = 0.2239

^{1}H and ^{13}C NMR spectra of compounds 3

^{1}H NMR of 3a

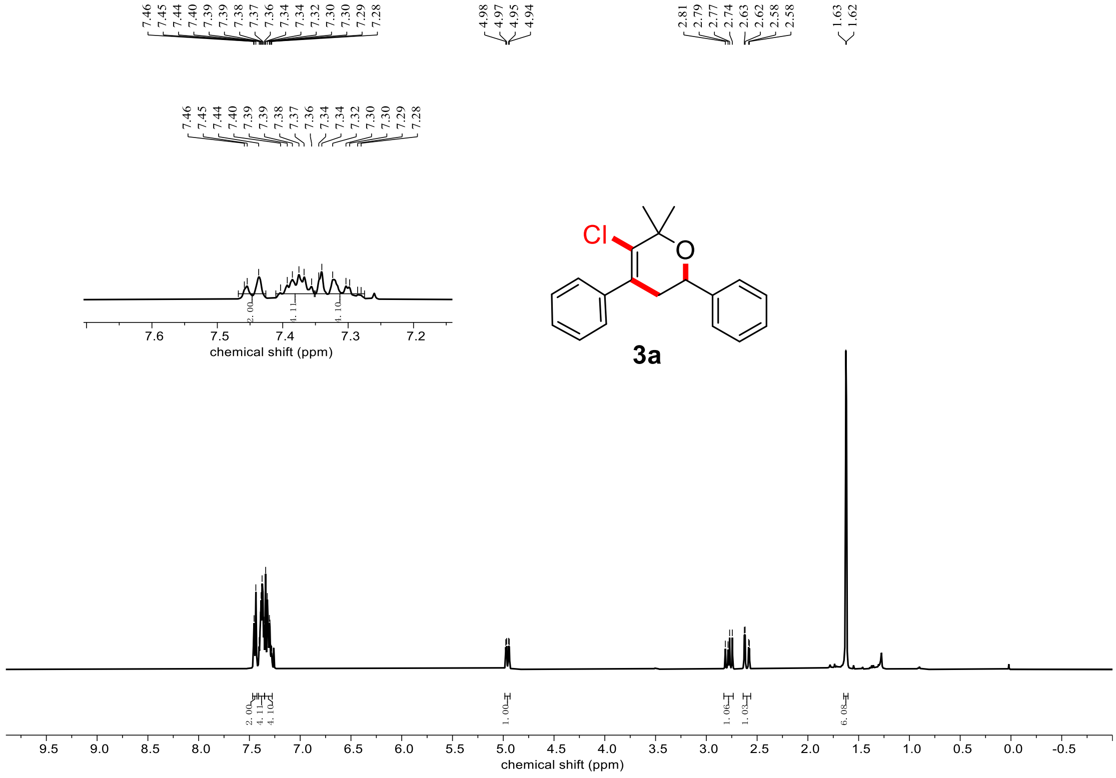

^{13}C NMR of 3a

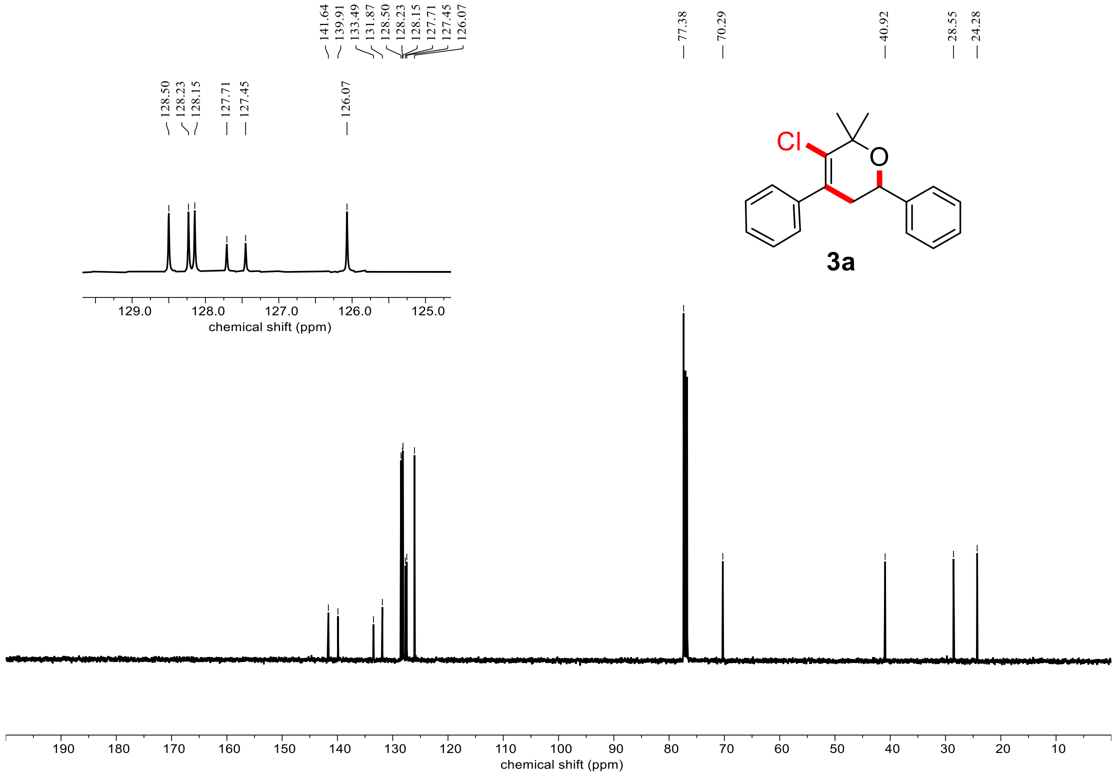

^{1}H NMR of 3b

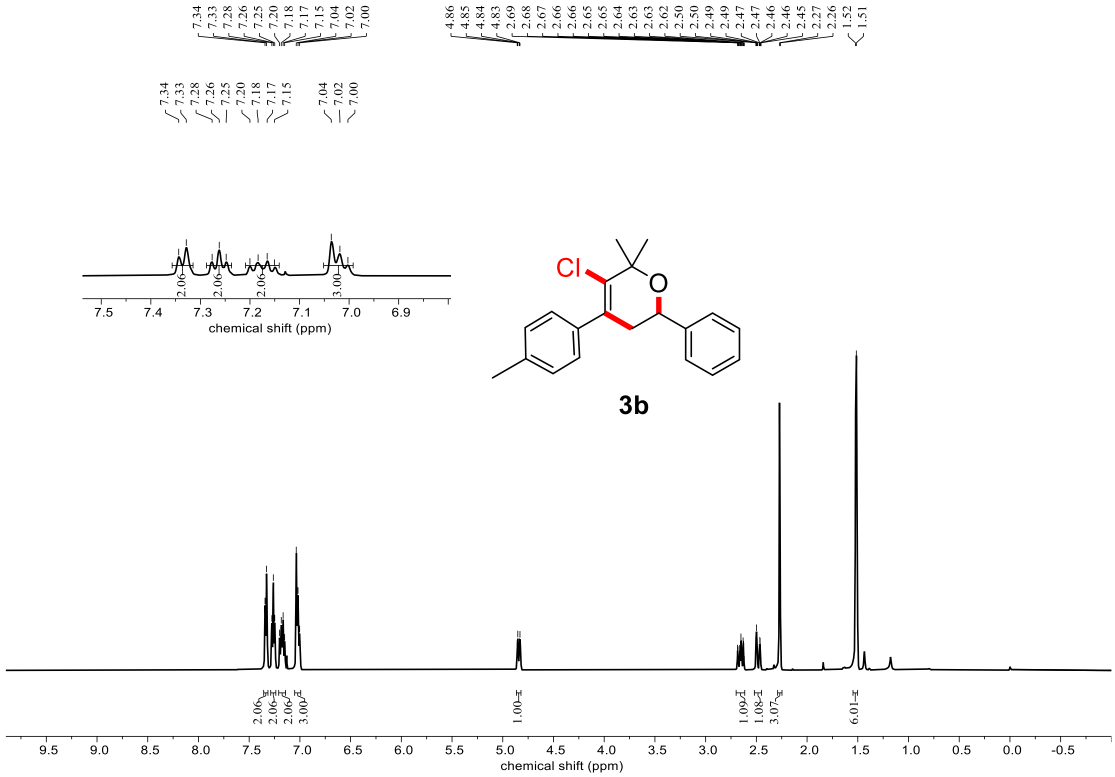

^{13}C NMR of 3b

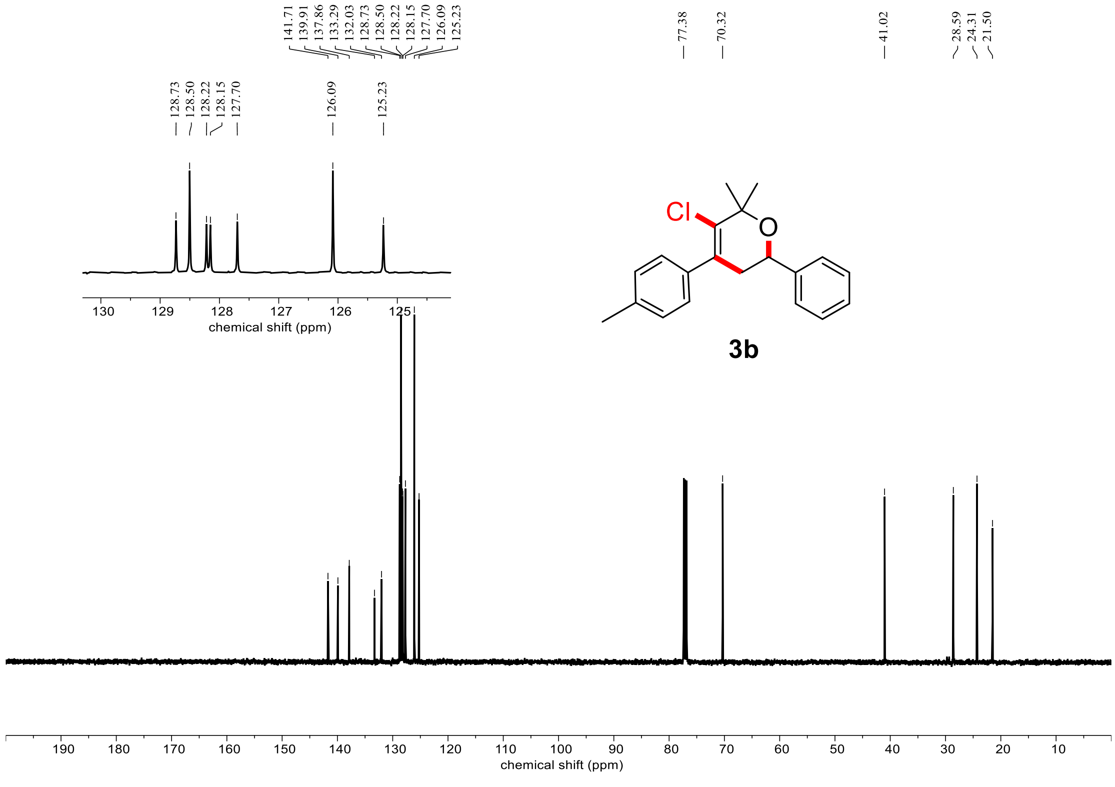

^{1}H NMR of 3c

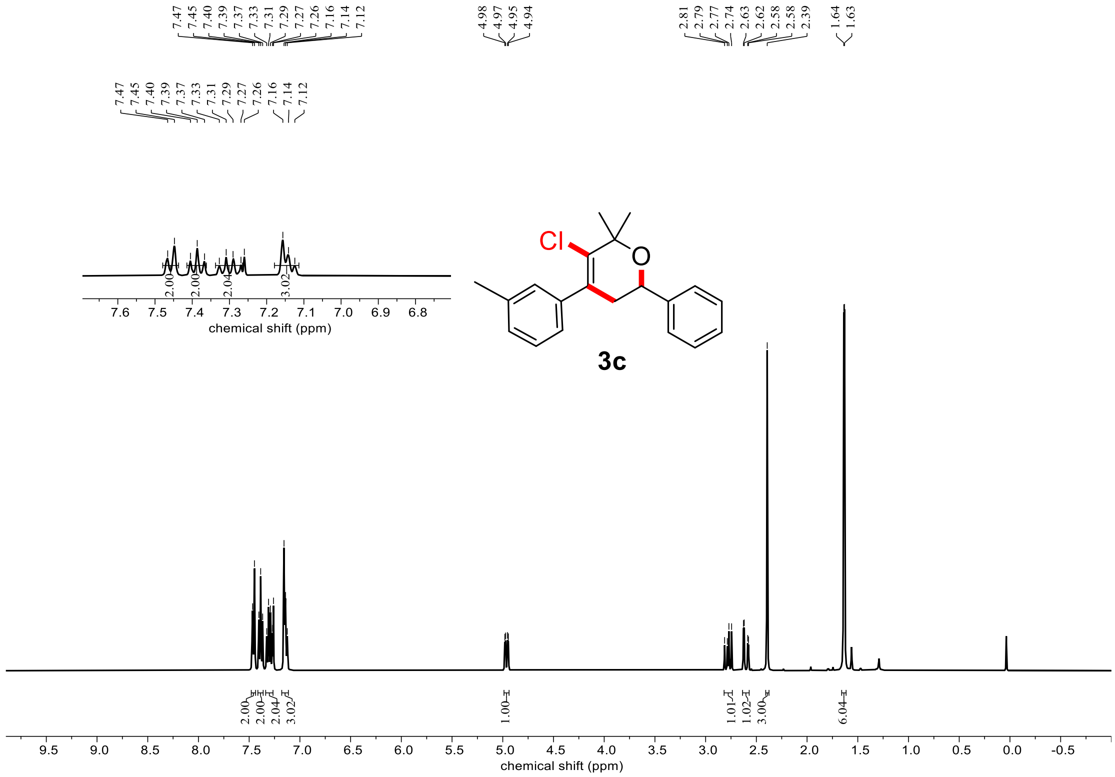

^{13}C NMR of 3c

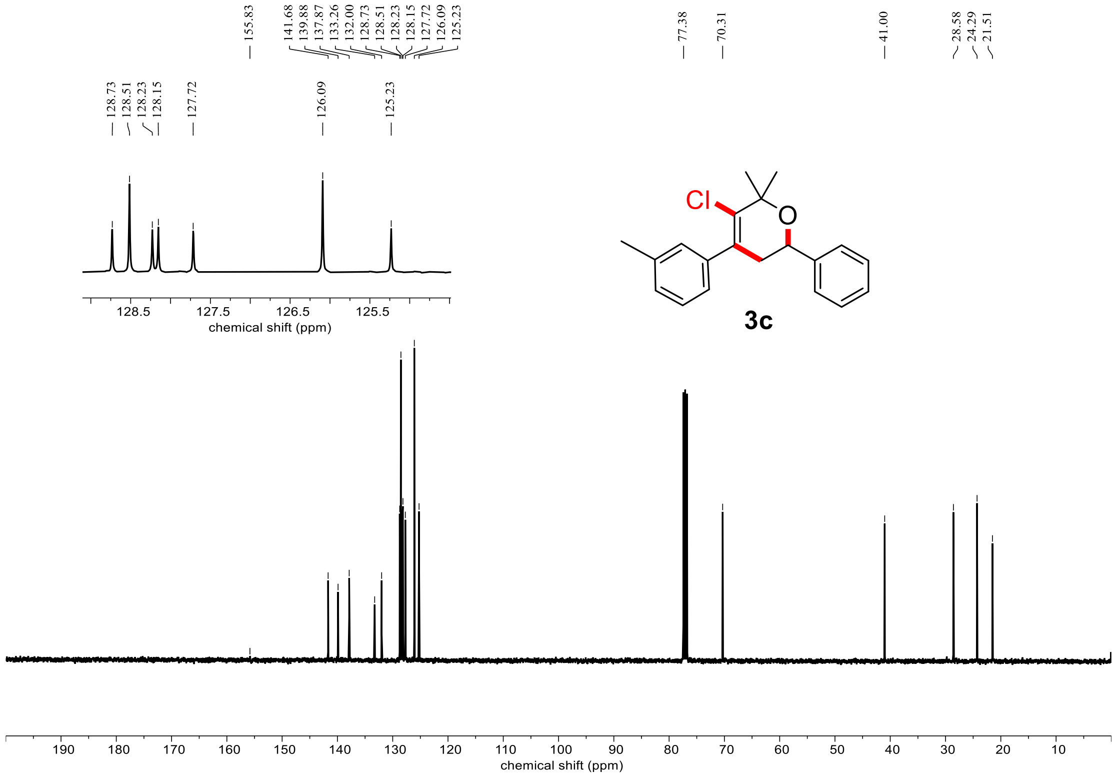

^{1}H NMR of 3d

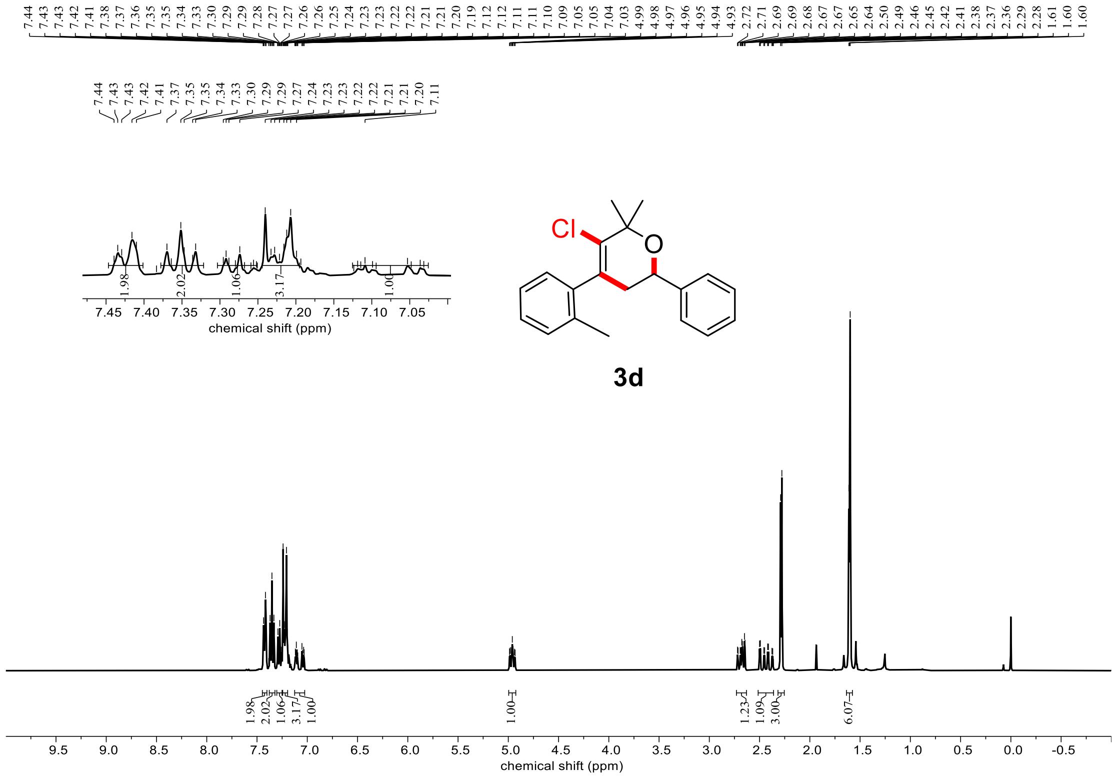

^{13}C NMR of 3d

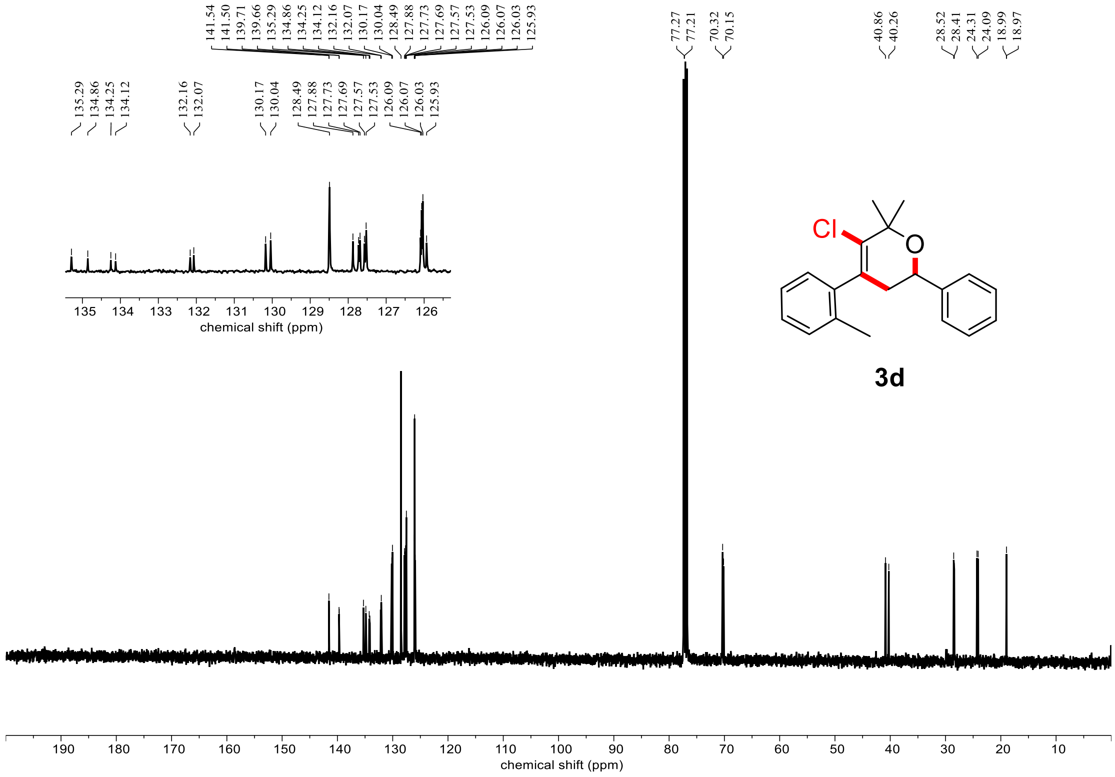

^{1}H NMR of 3e

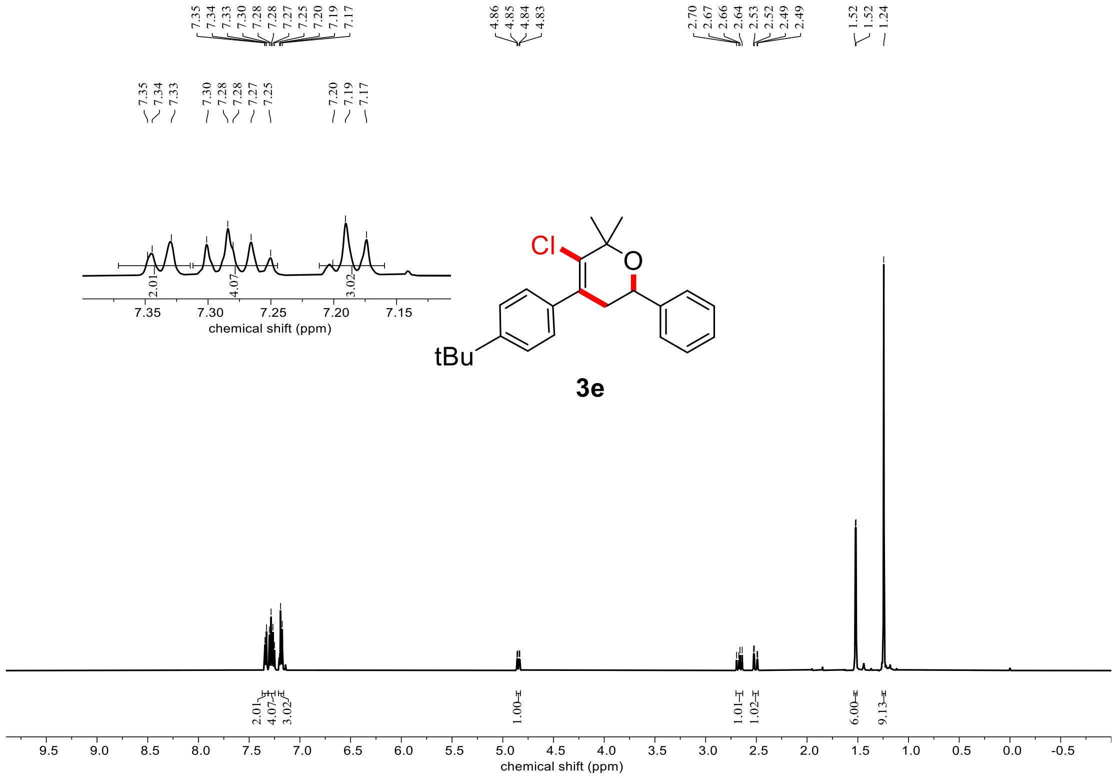

^{13}C NMR of 3e

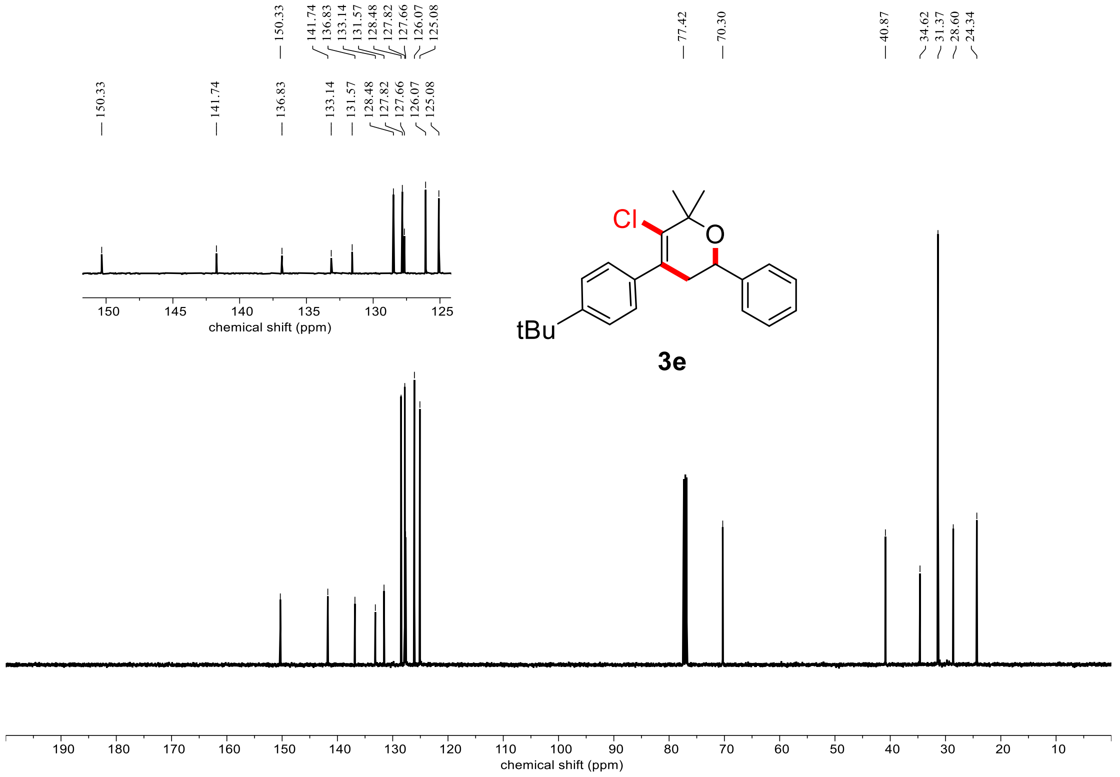

^{1}H NMR of 3f

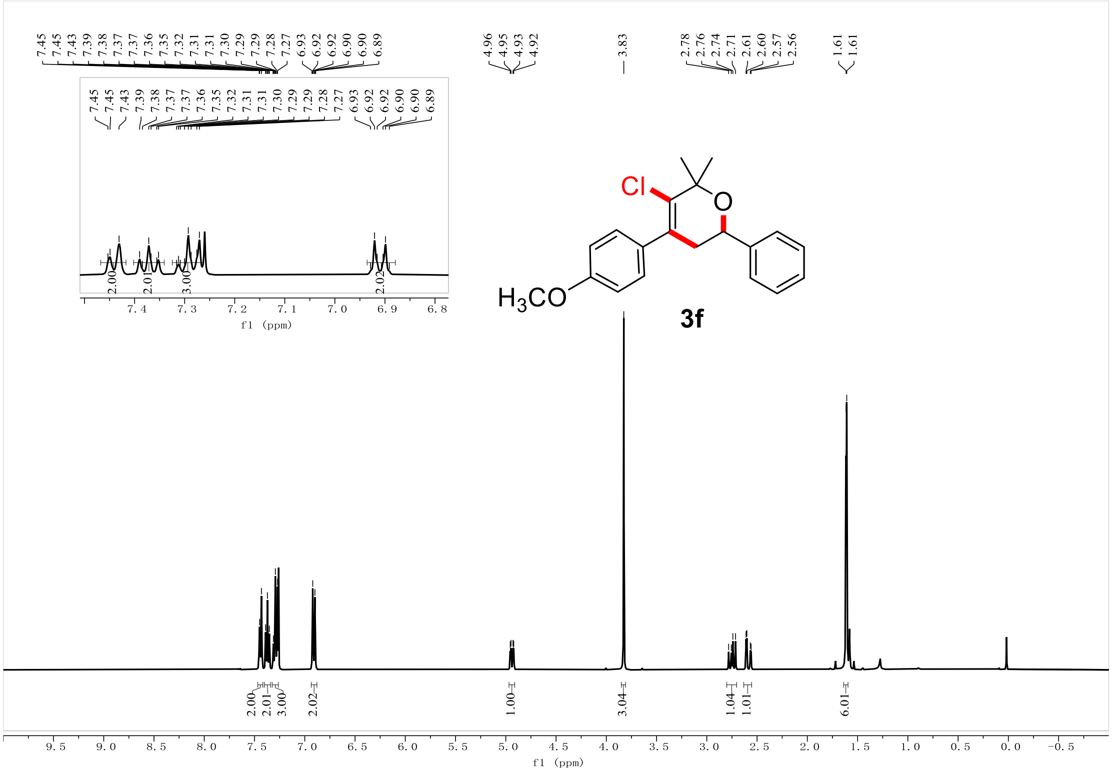

^{13}C NMR of 3f

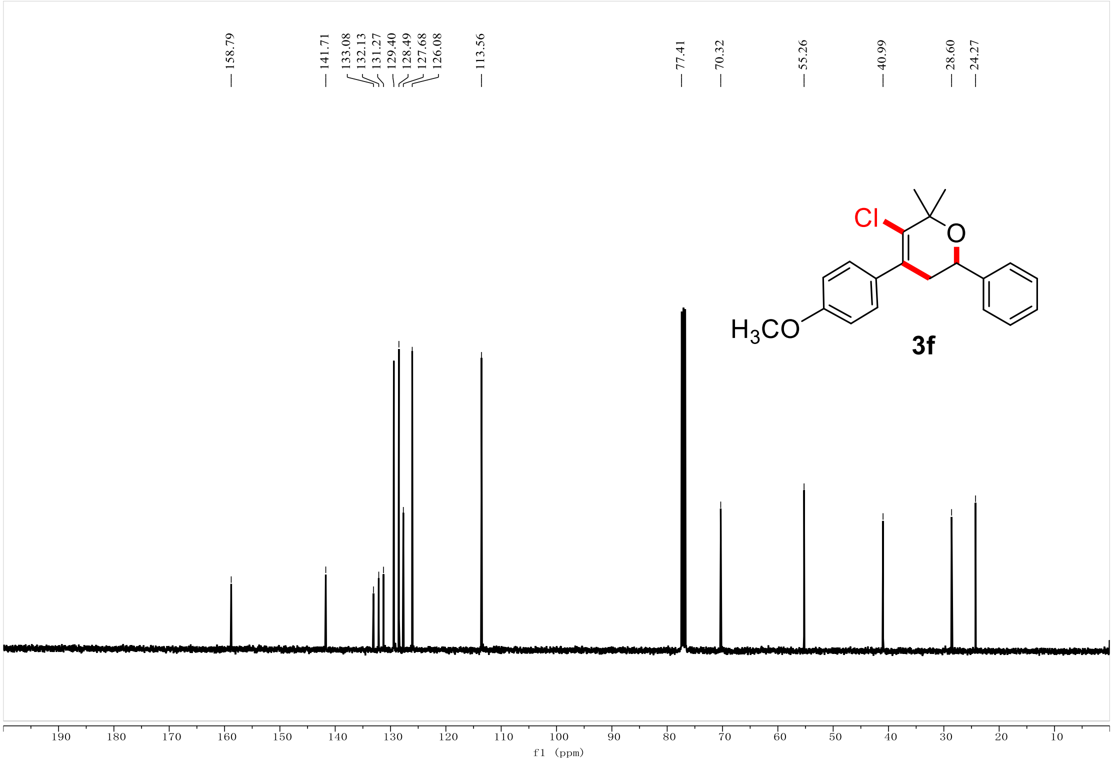

^{1}H NMR of 3g

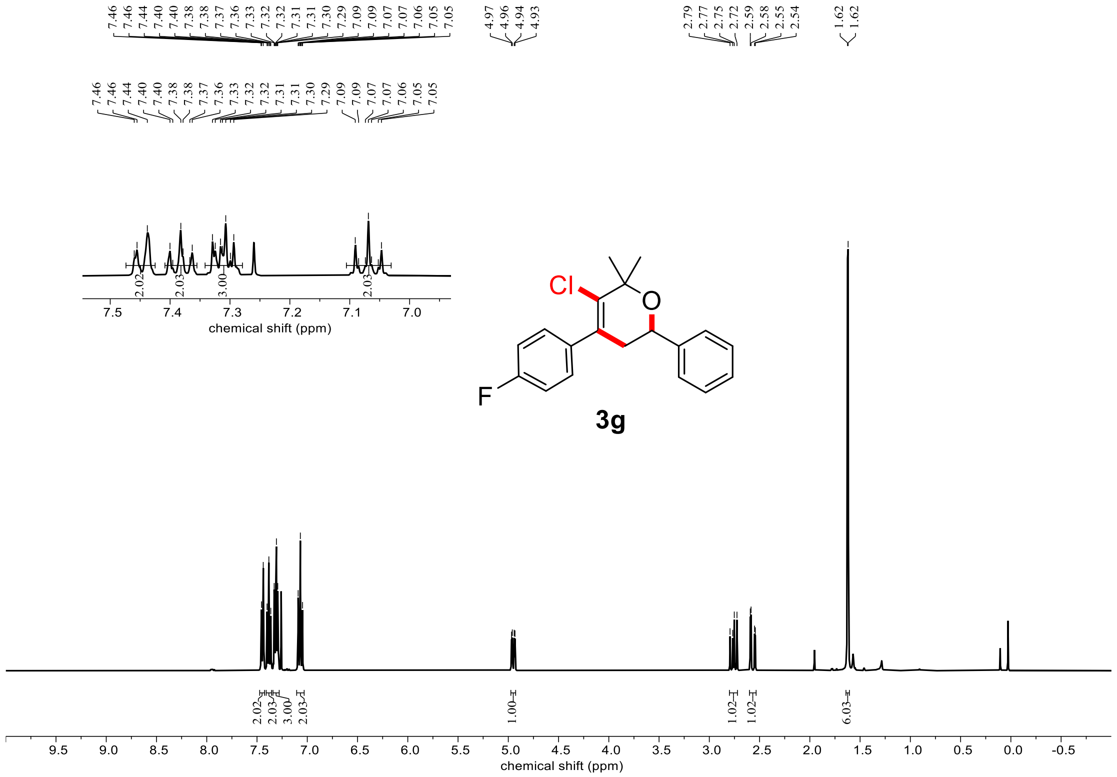

^{13}C NMR of 3g

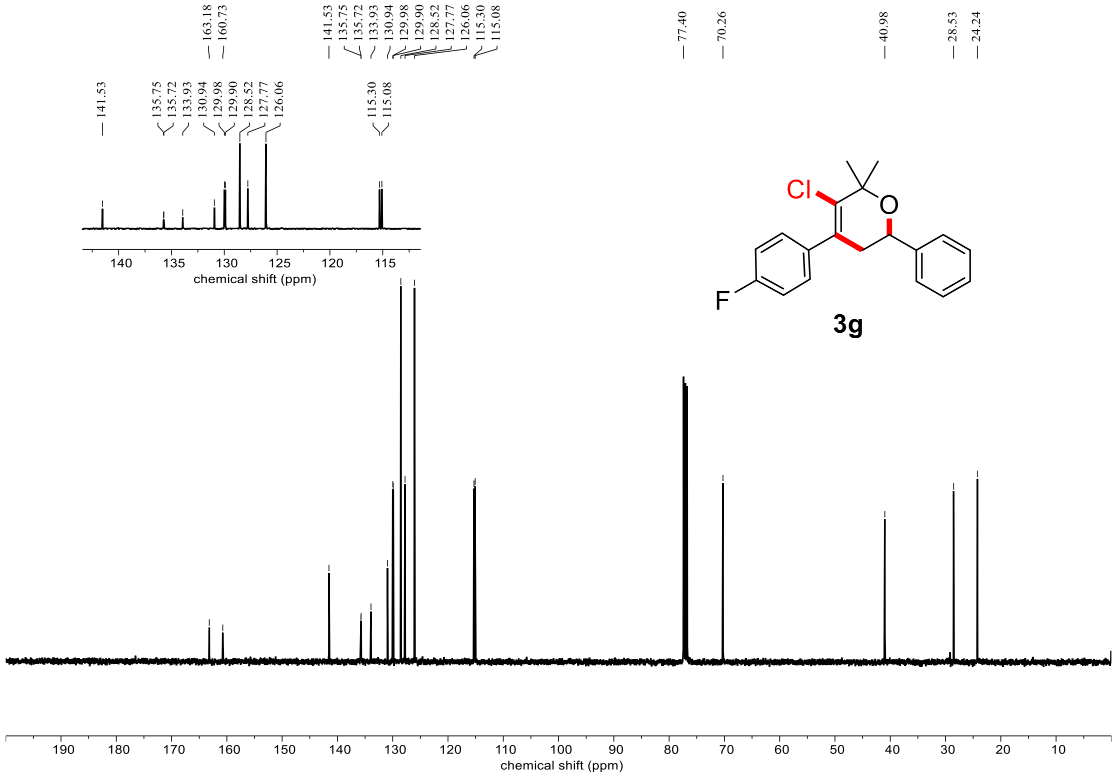

^{19}F NMR of 3g

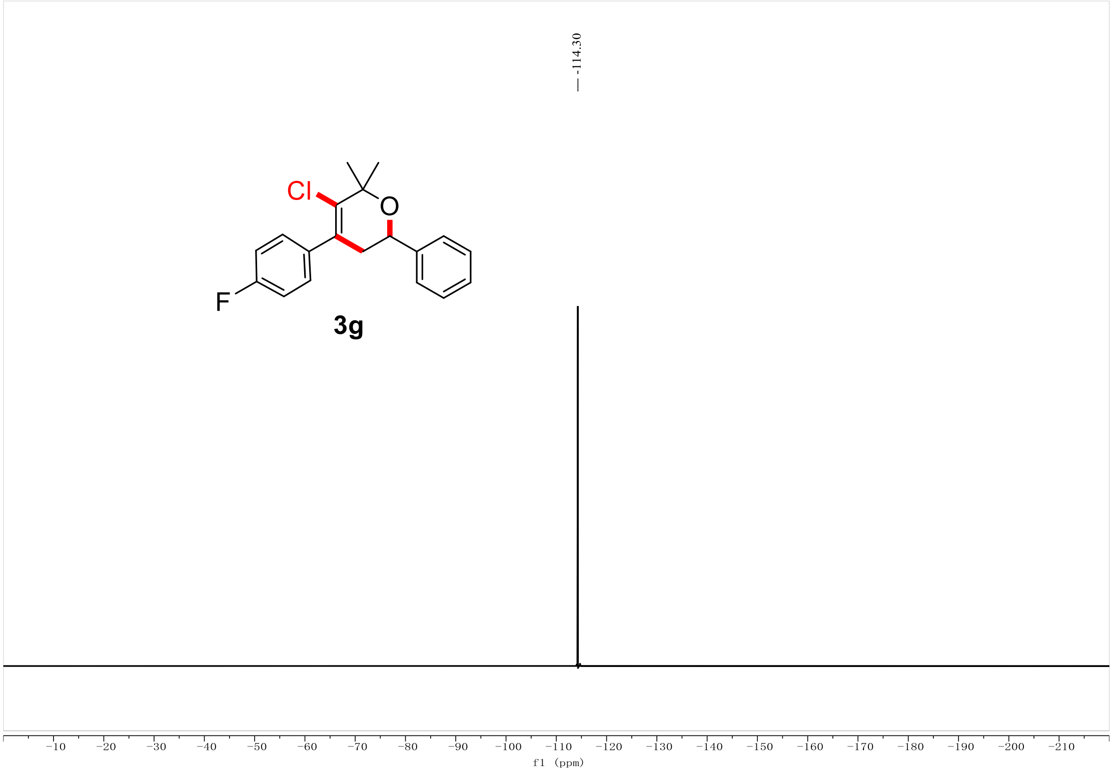
-114.30
Cl
O
F
3g
-10
-20
-30
-40
-50
-60
-70
-80
-90
-100
-110
-120
-130
-140
-150
-160
-170
-180
-190
-200
-210
f1 (ppm)

^{1}H NMR of 3h

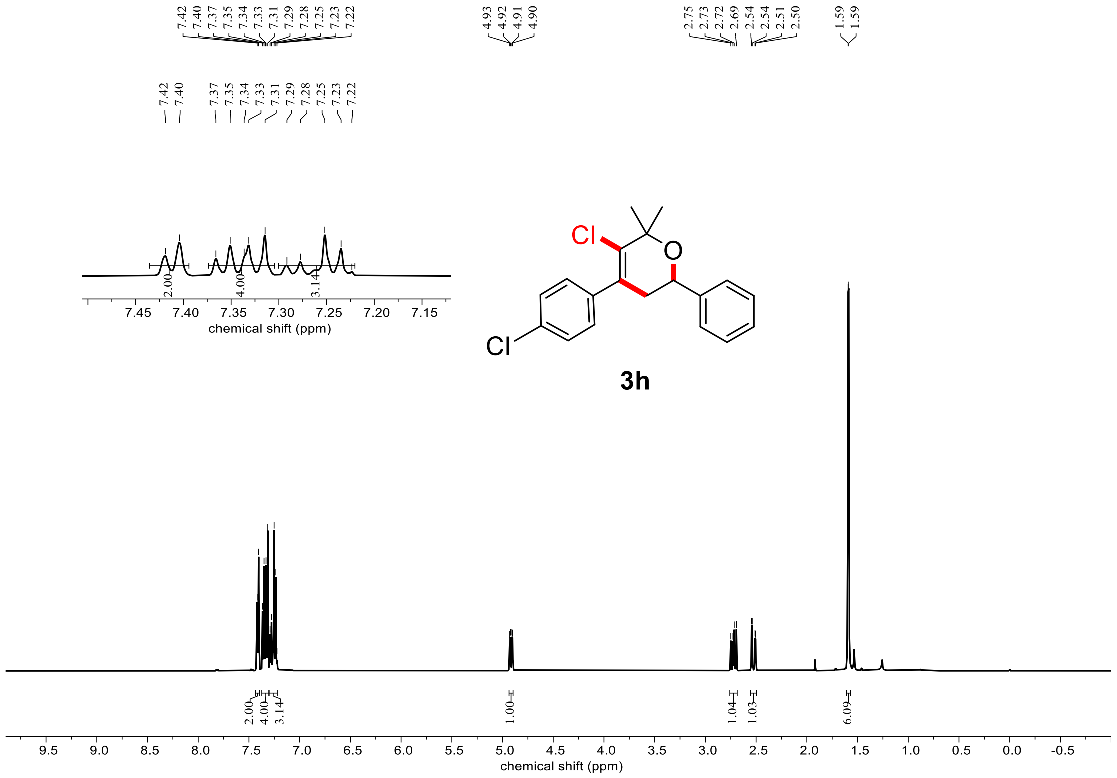

^{13}C NMR of 3h

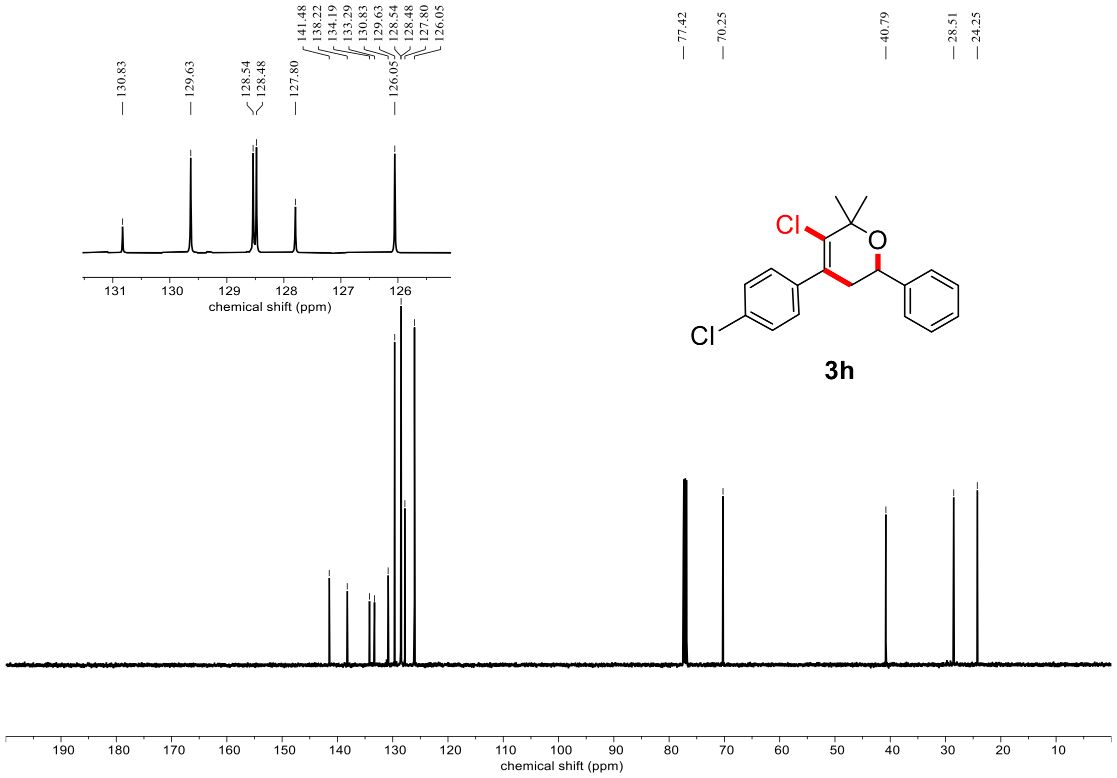

^{1}H NMR of 3i

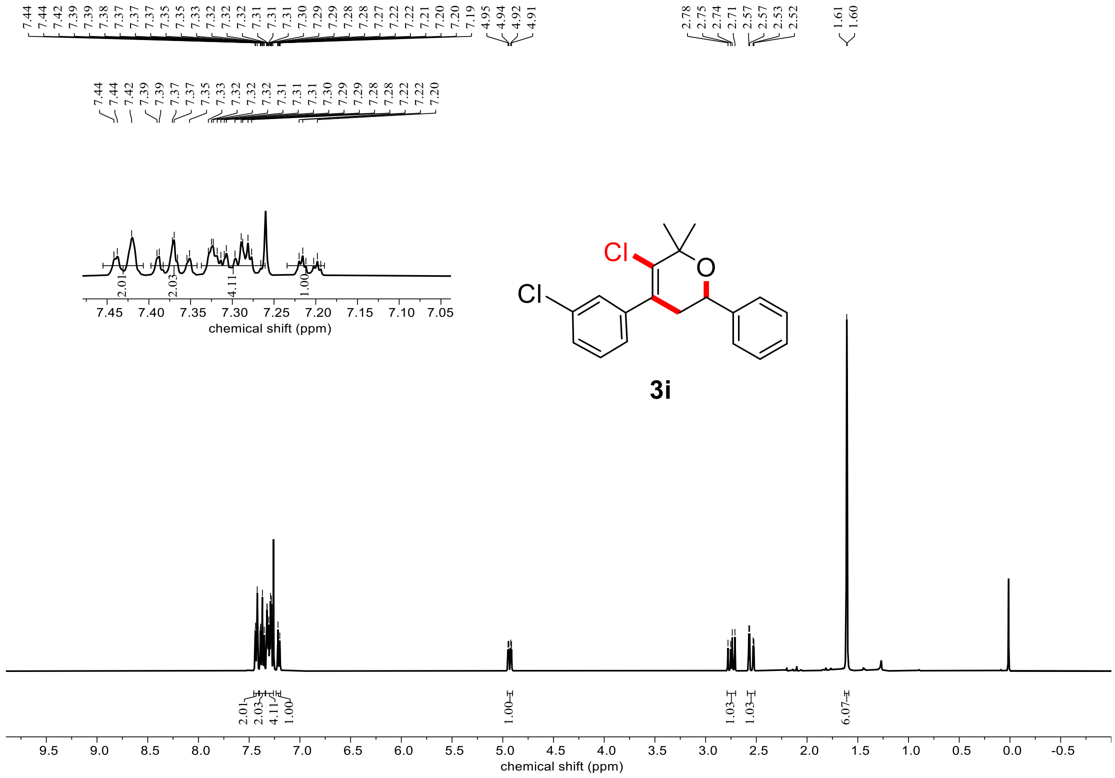

^{13}C NMR of 3i

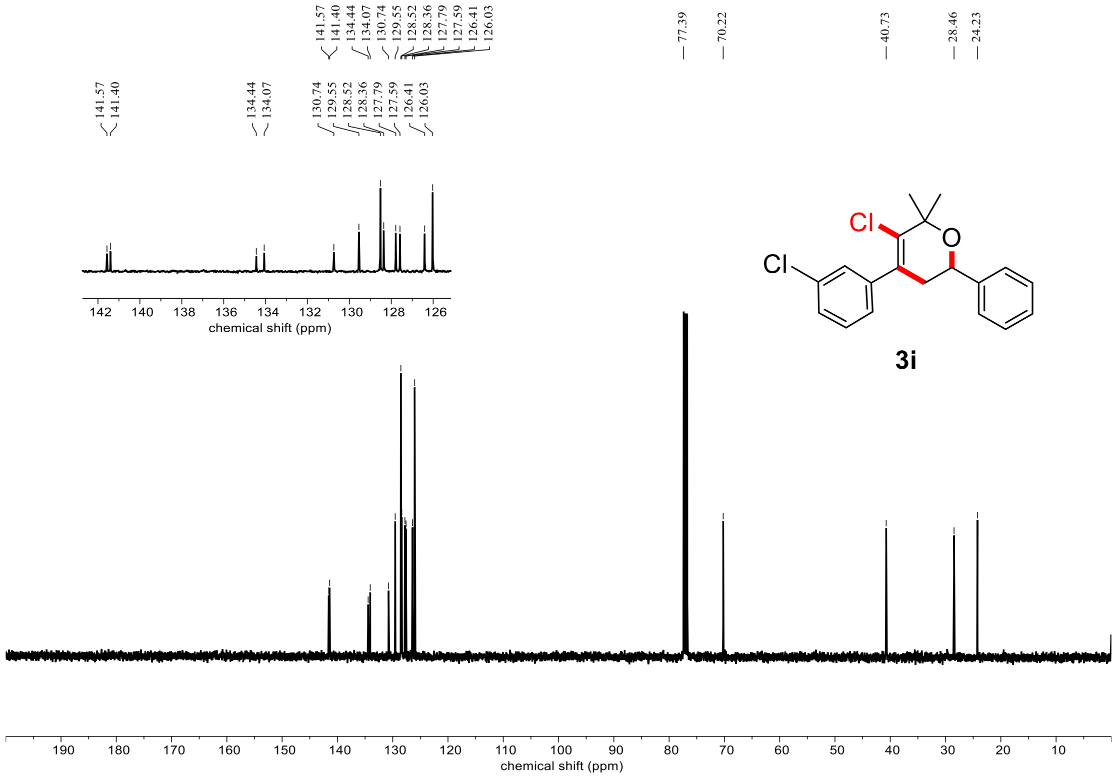

^{1}H NMR of 3k

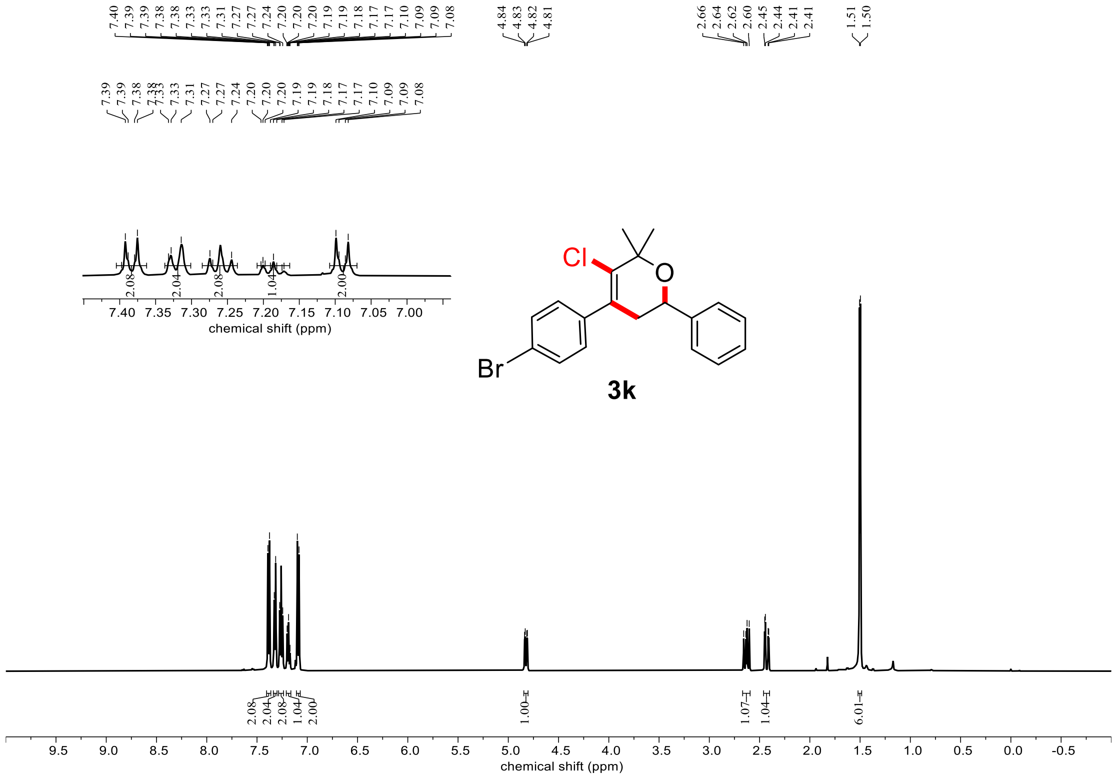

^{13}C NMR of 3k

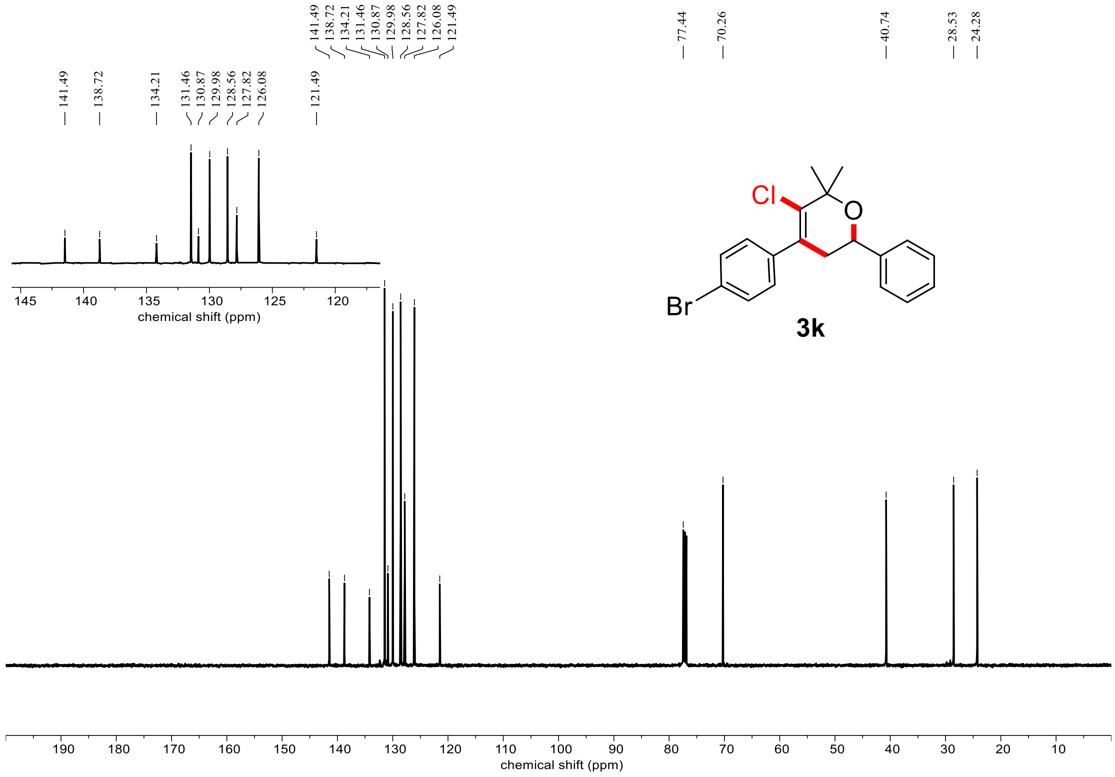

^{1}H NMR of 3l

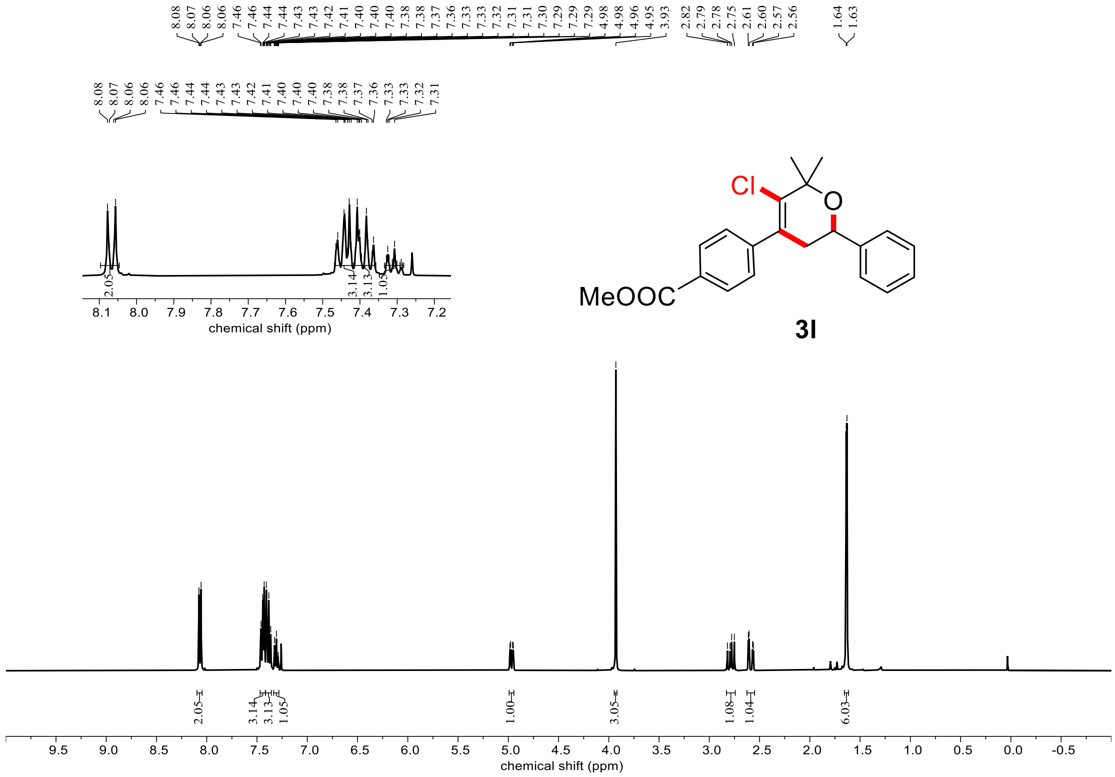

^{13}C NMR of 3l

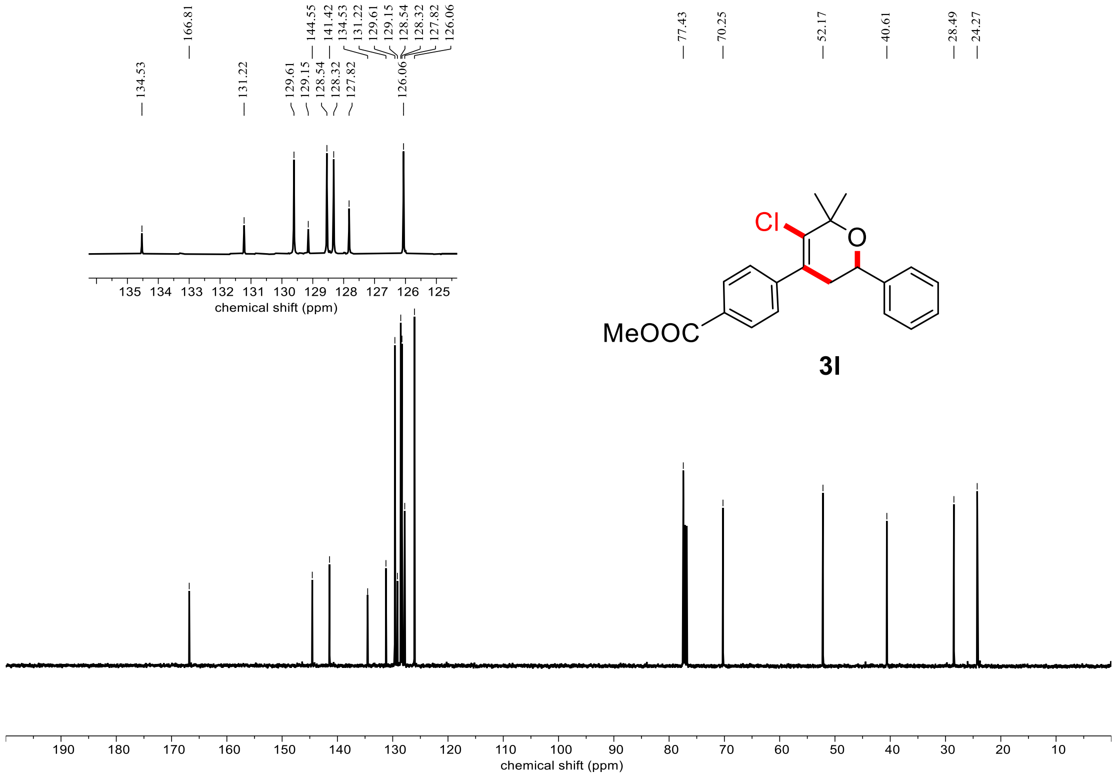

^{1}H NMR of 3m

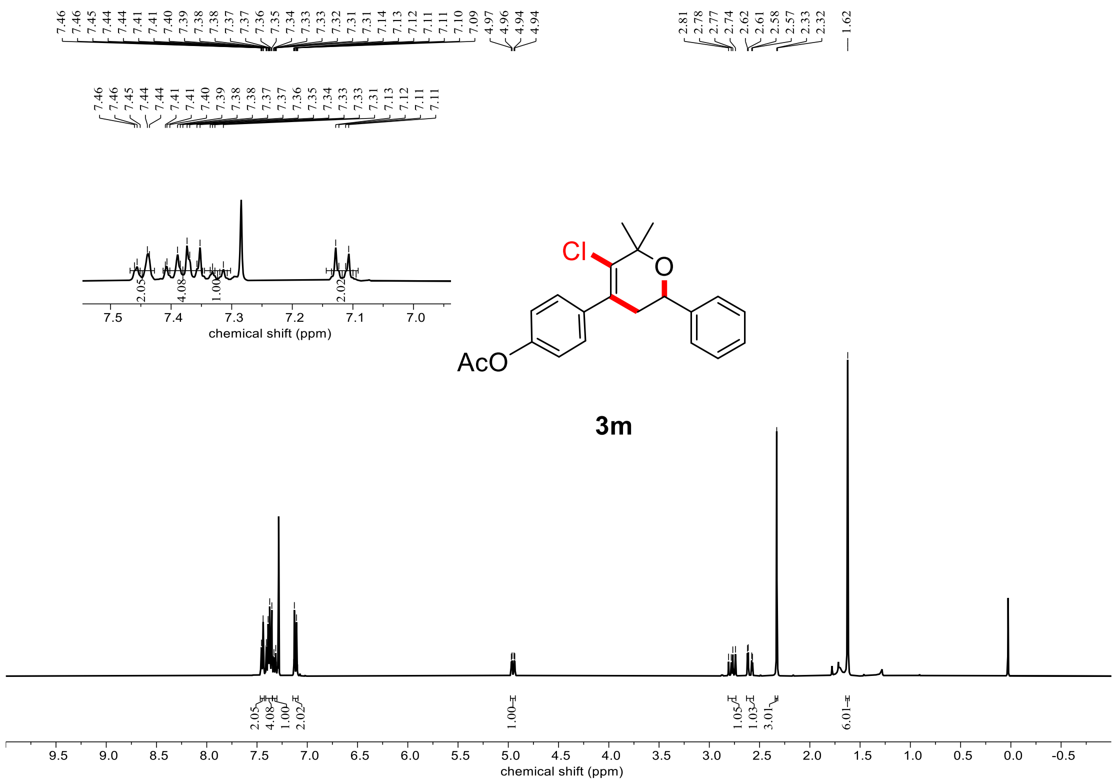

^{13}C NMR of 3m

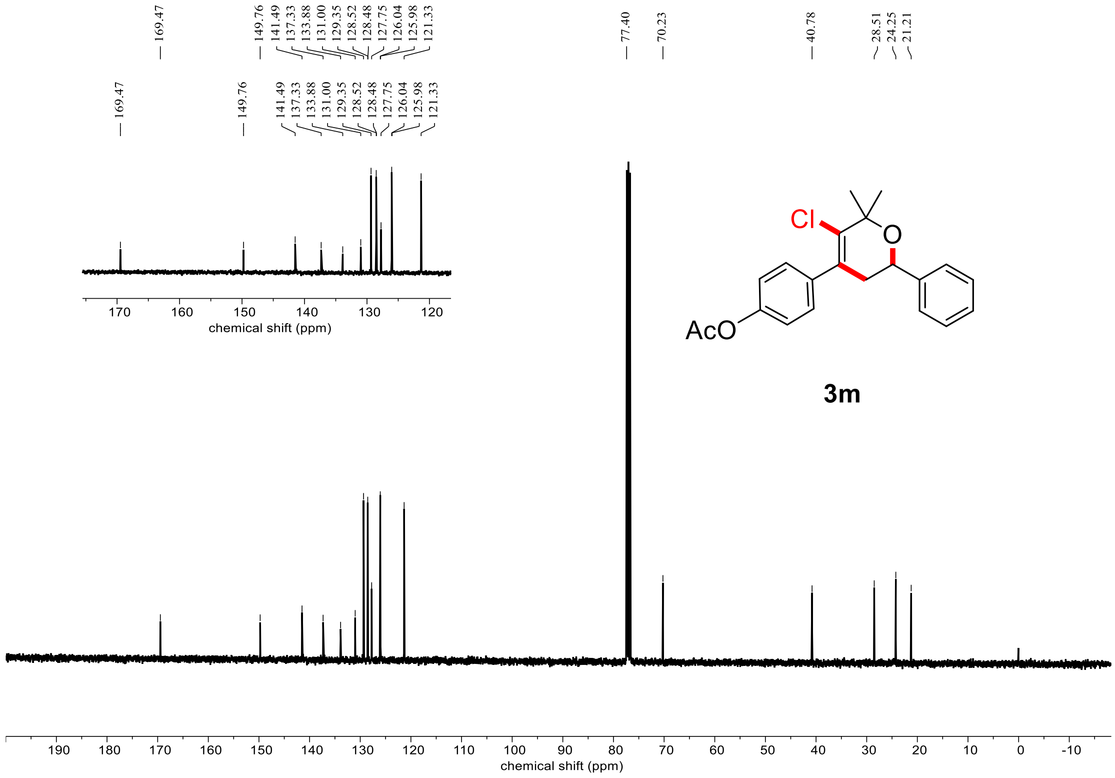

^{1}H NMR of 3n

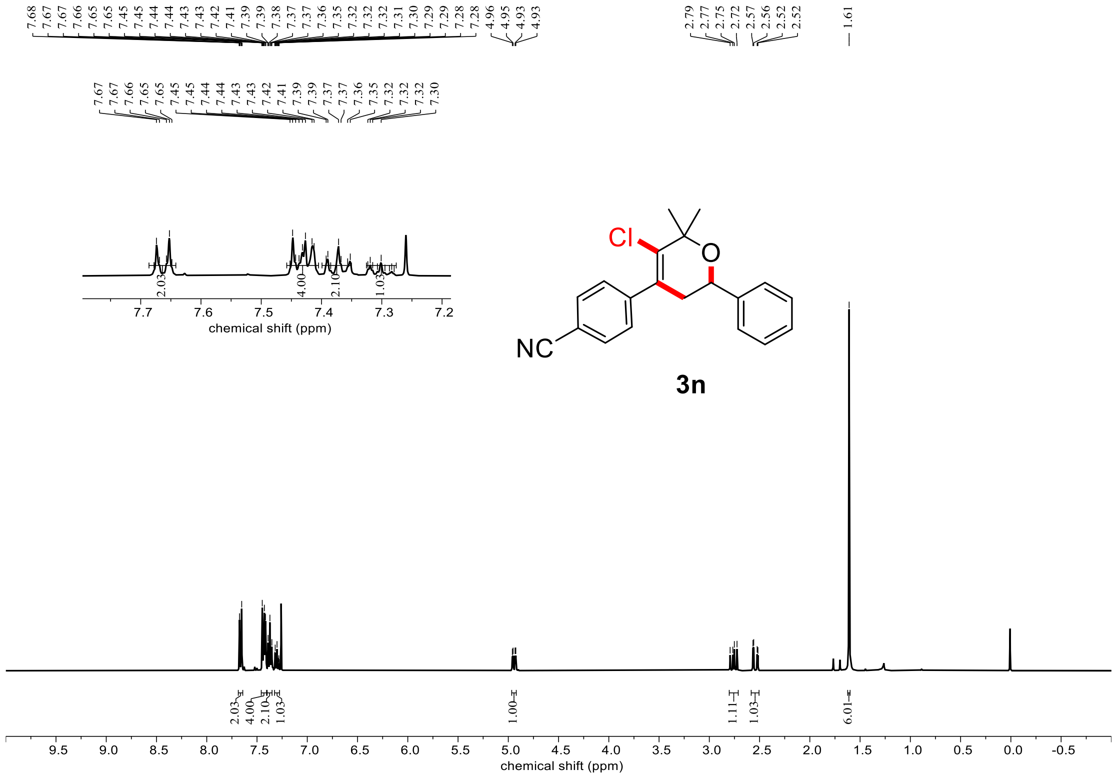

^{13}C NMR of 3n

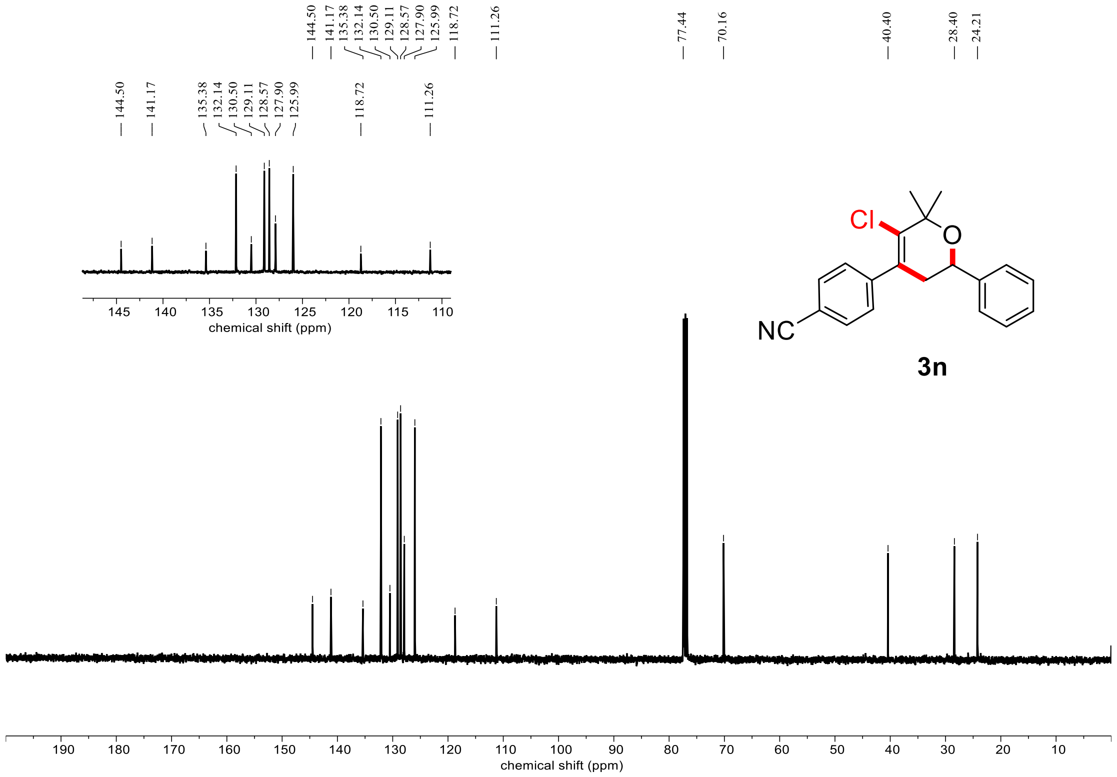

^{1}H NMR of 3o

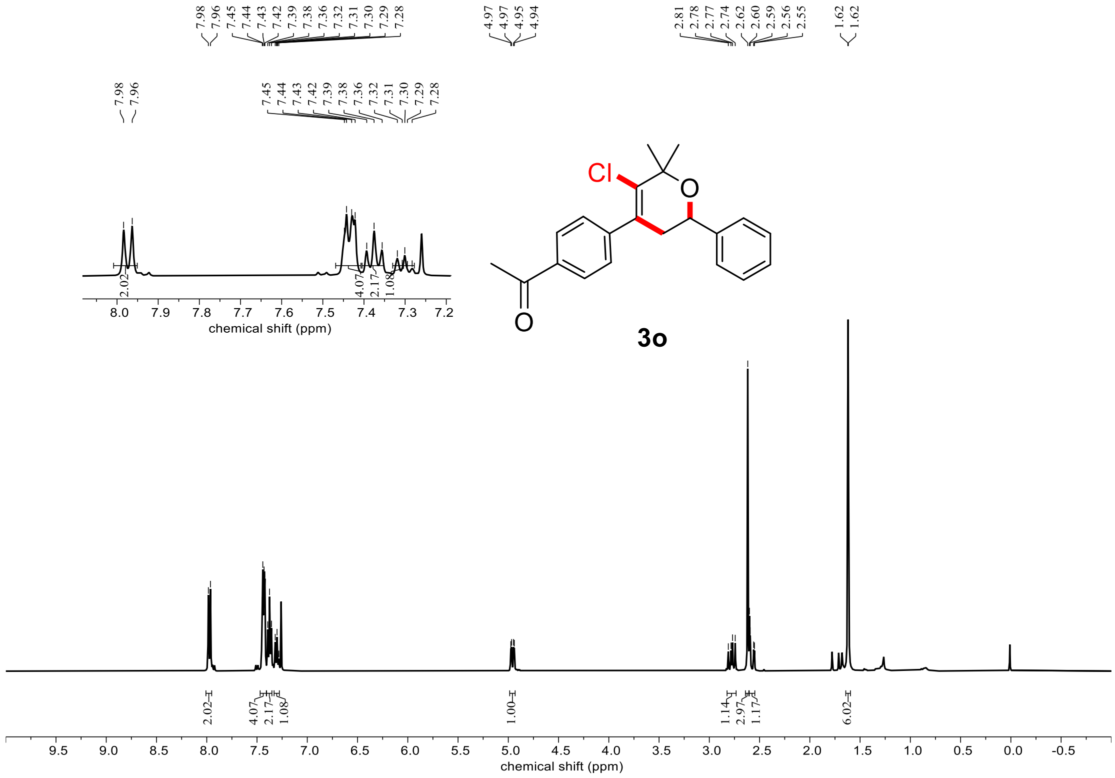

^{13}C NMR of 3o

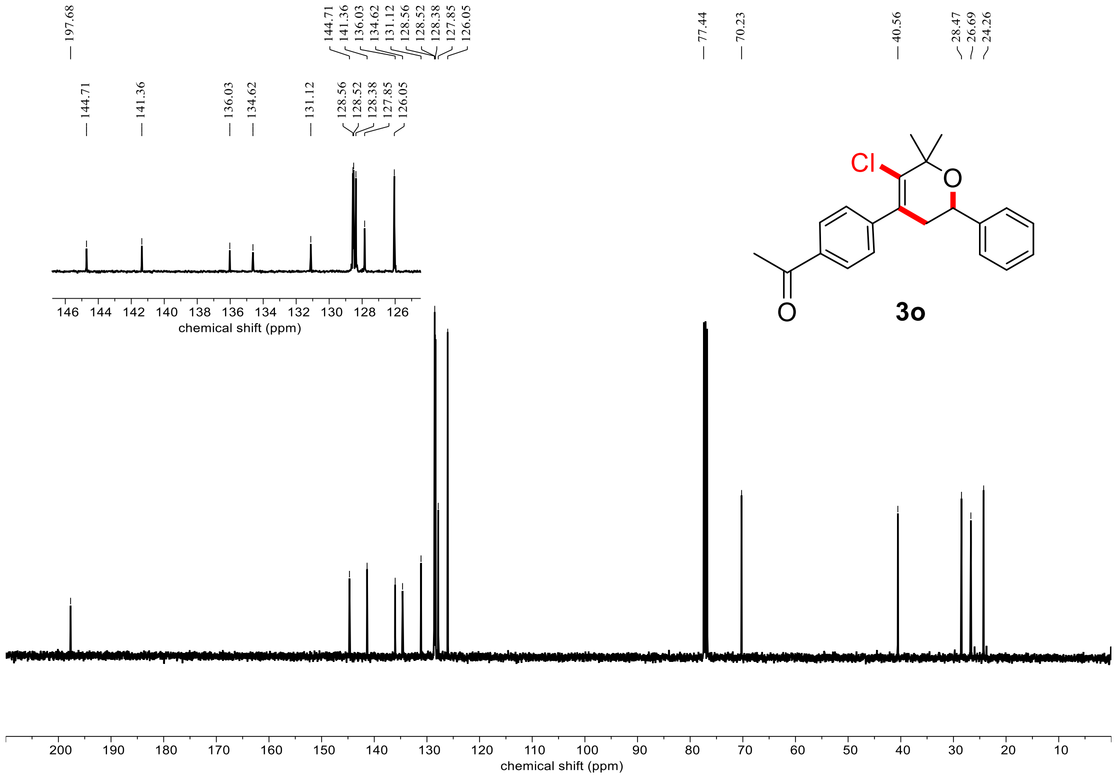

^{1}H NMR of 3p

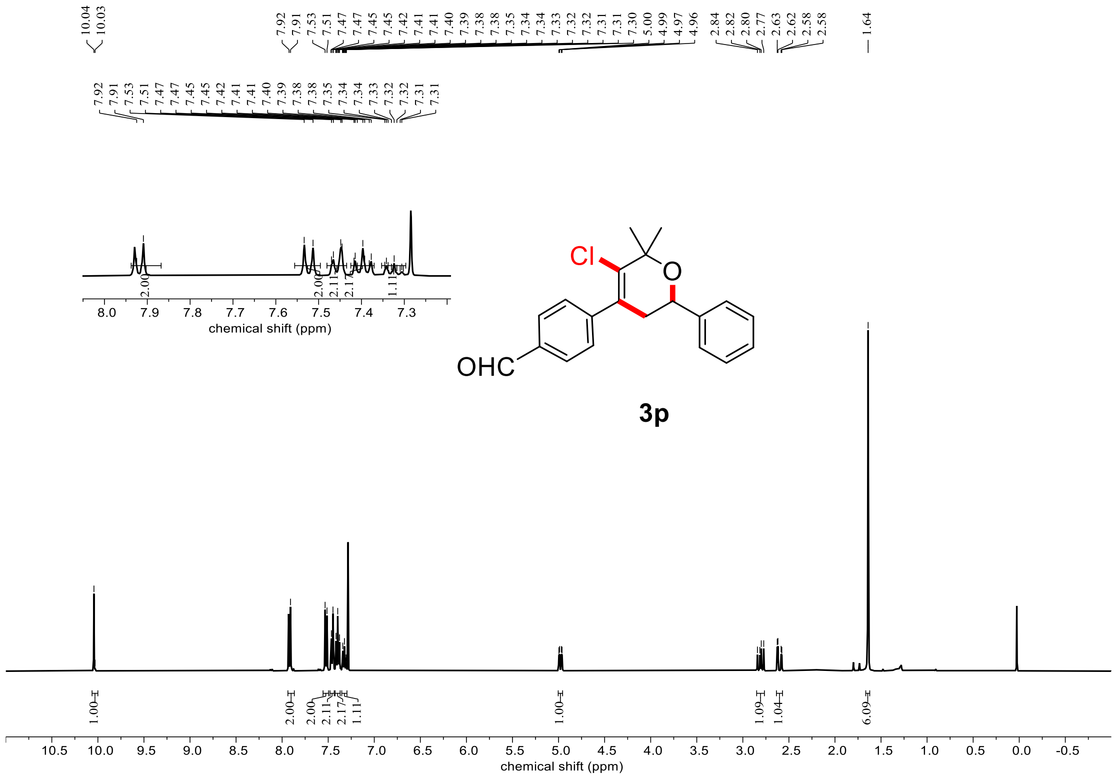

^{13}C NMR of 3p

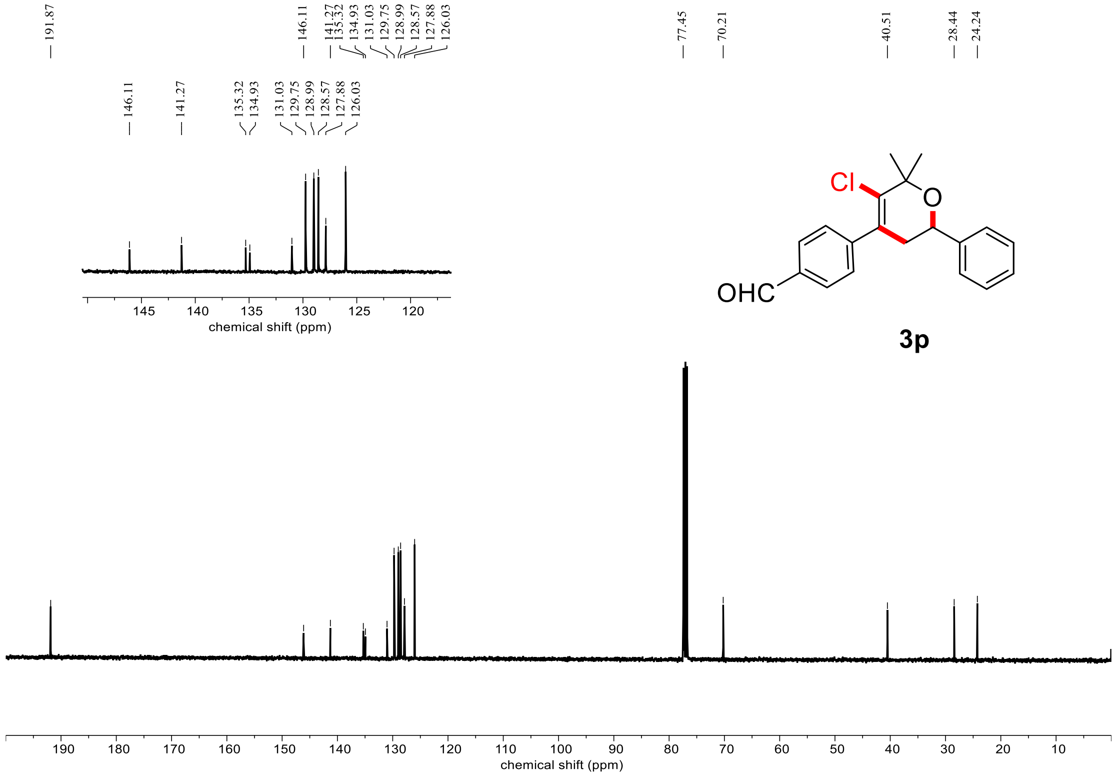

^{1}H NMR of 3q

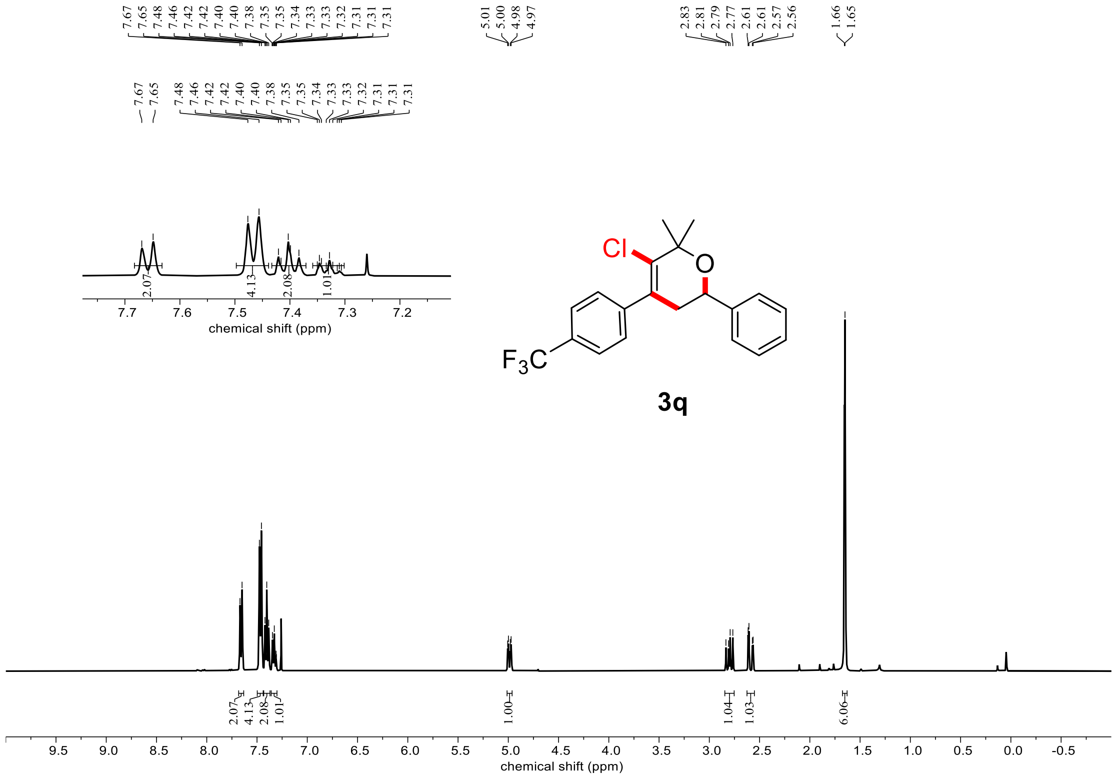

^{13}C NMR of 3q

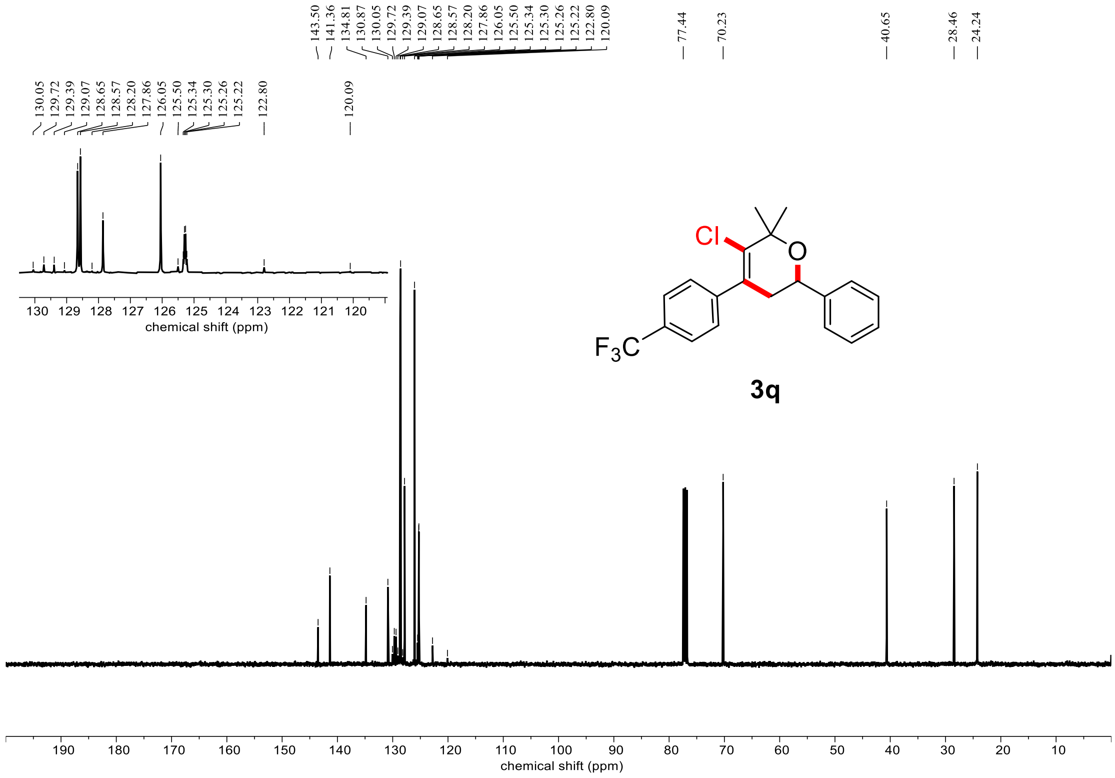

^{19}F NMR of 3q

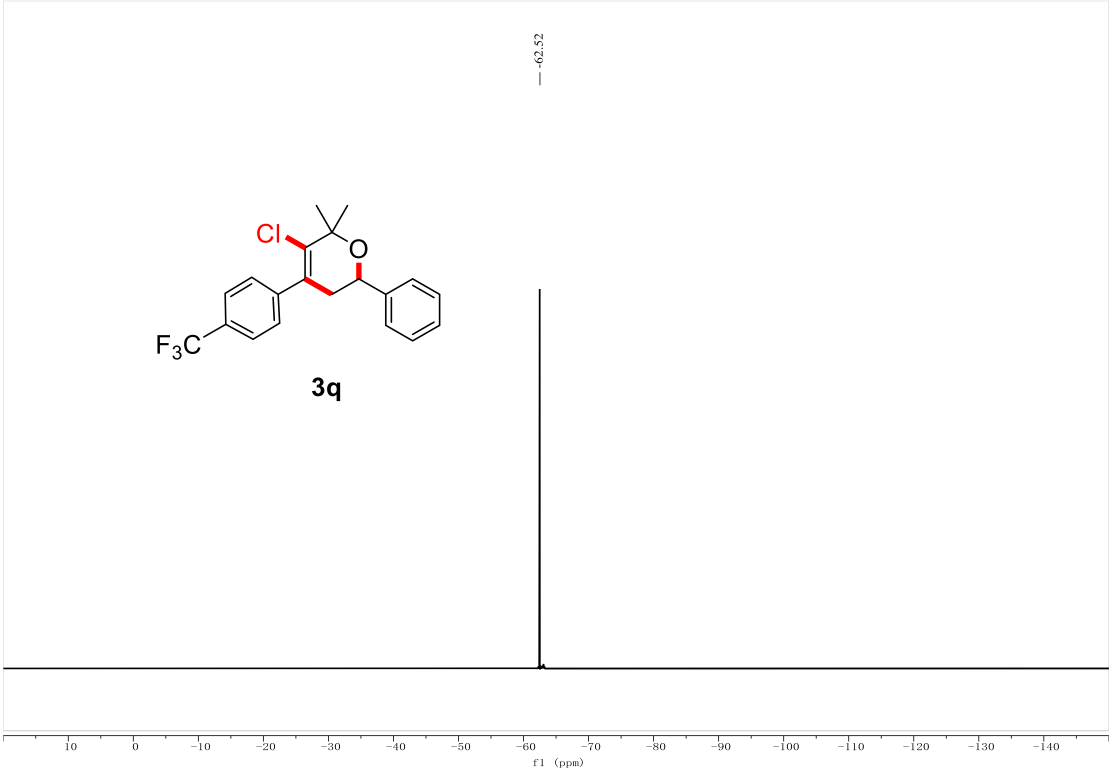

-62.52
Cl
O
F3C
3q
10
0
-10
-20
-30
-40
-50
-60
-70
-80
-90
-100
-110
-120
-130
-140
f1 (ppm)

^{1}H NMR of 3r

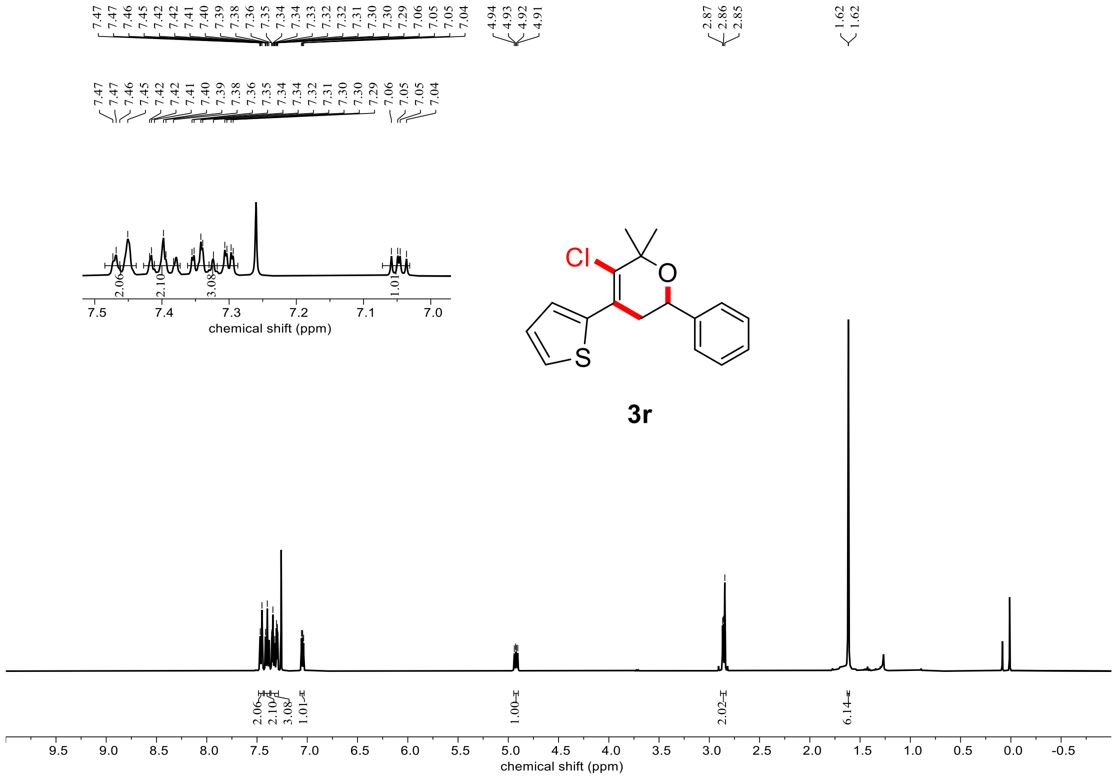

^{13}C NMR of 3r

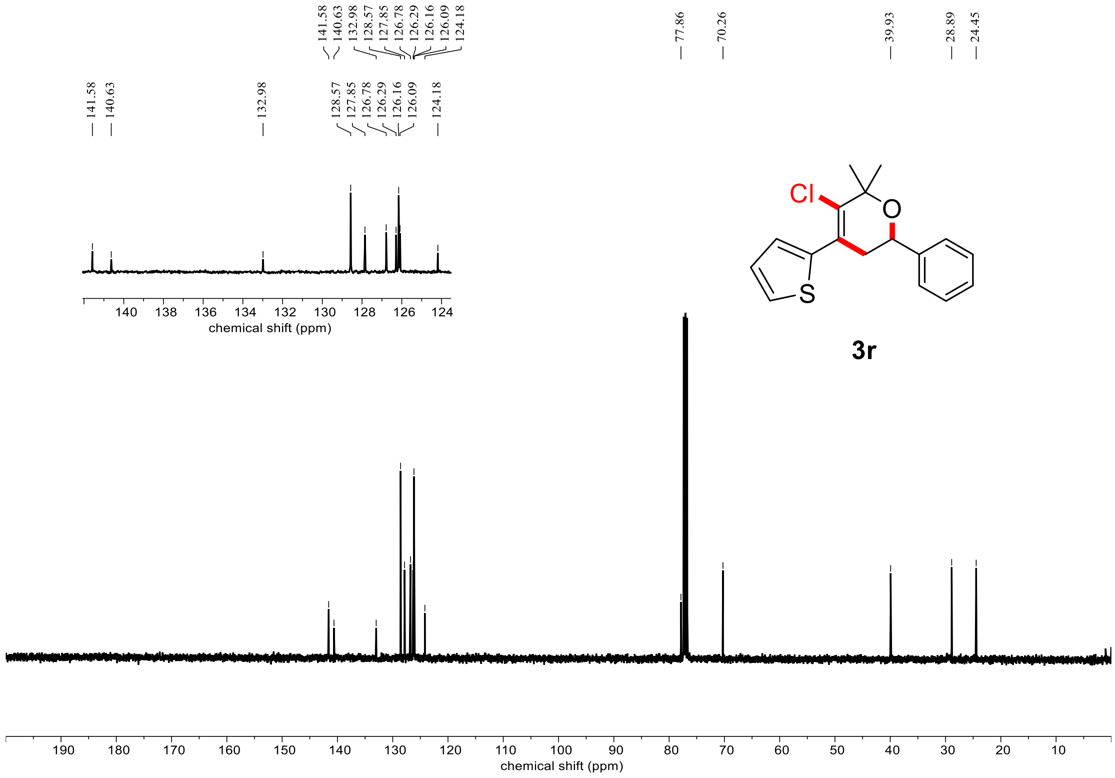

^{1}H NMR of 3v

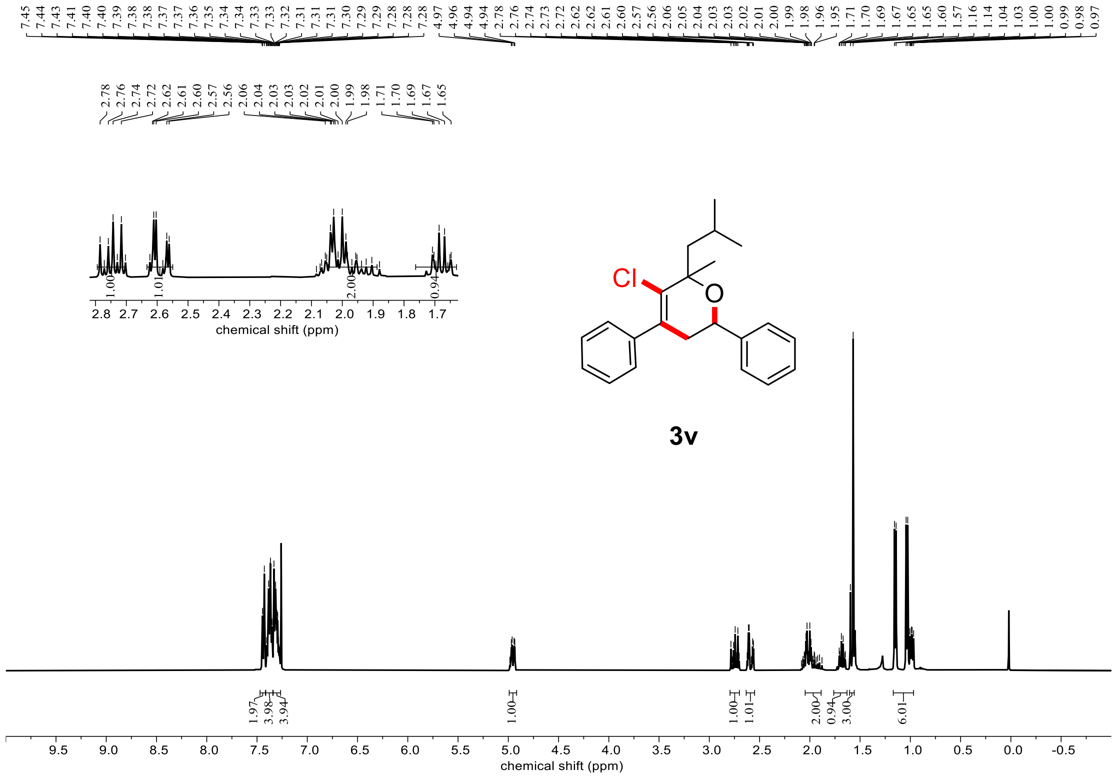

^{13}C NMR of 3v

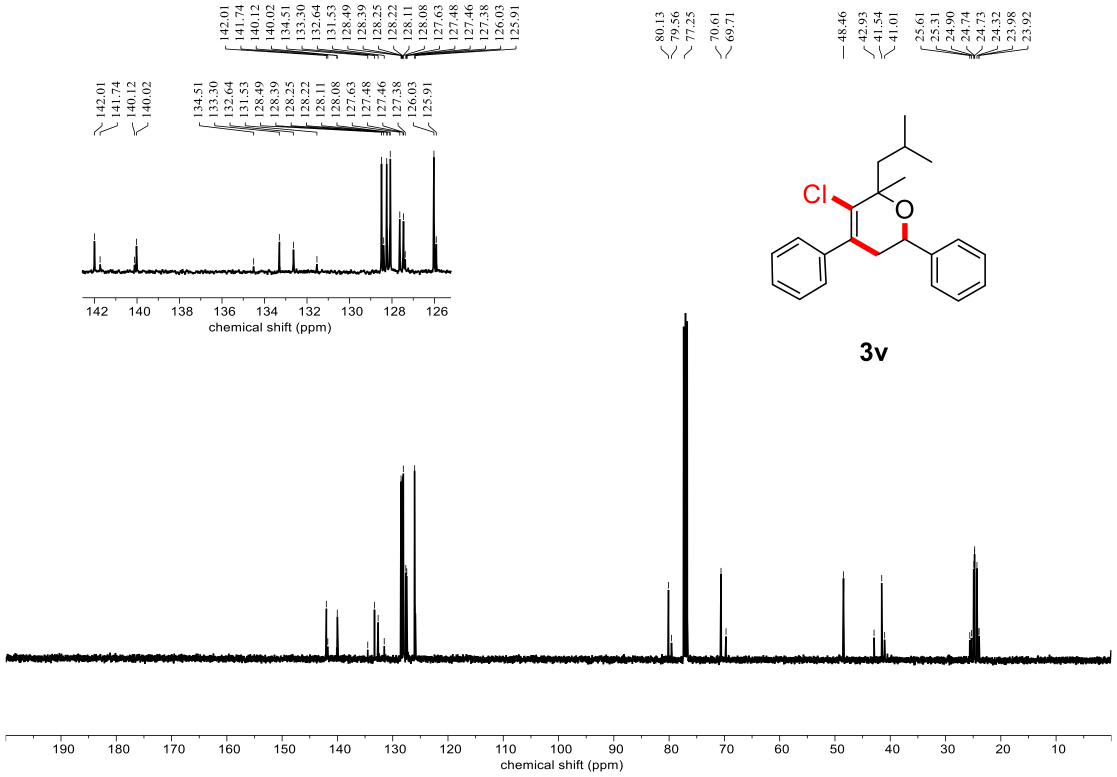

^{1}H NMR of 3w

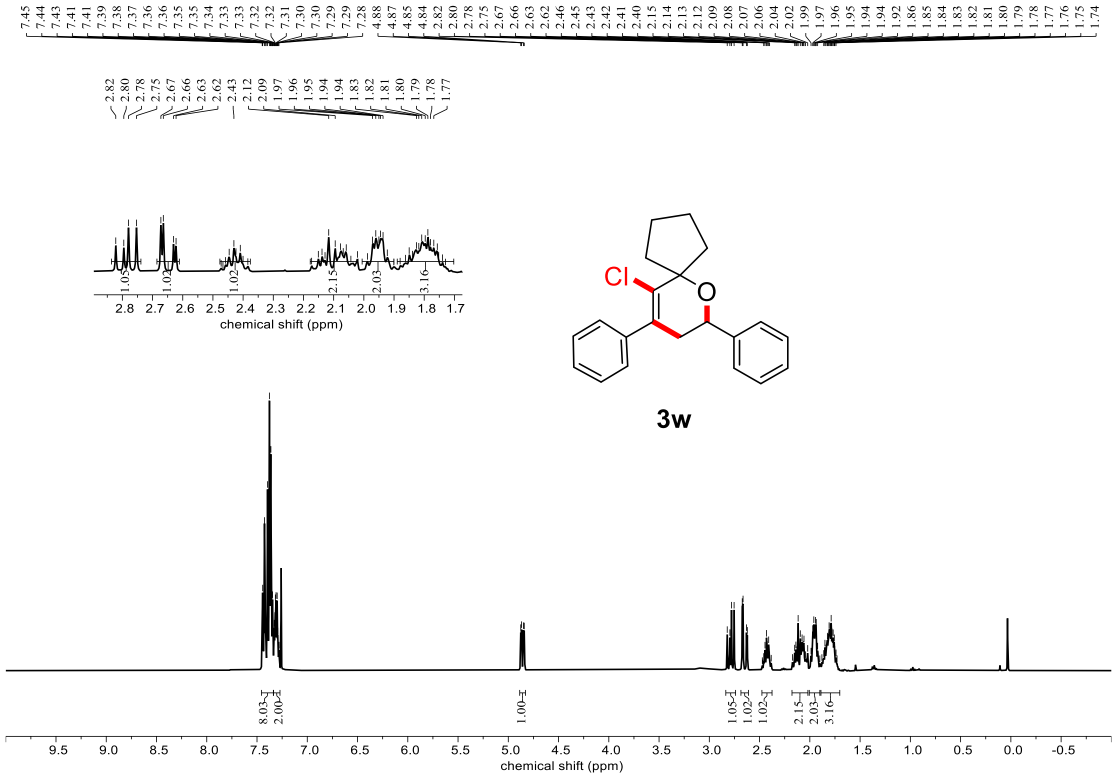

^{13}C NMR of 3w

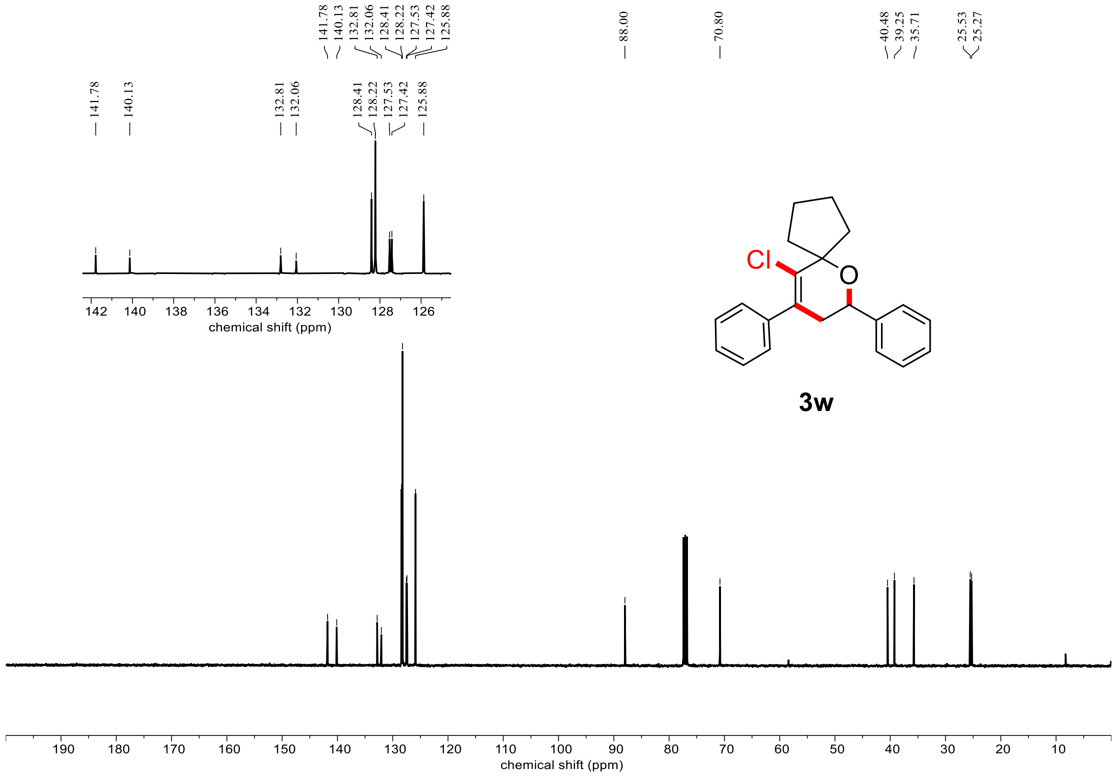

^{1}H NMR of 3x

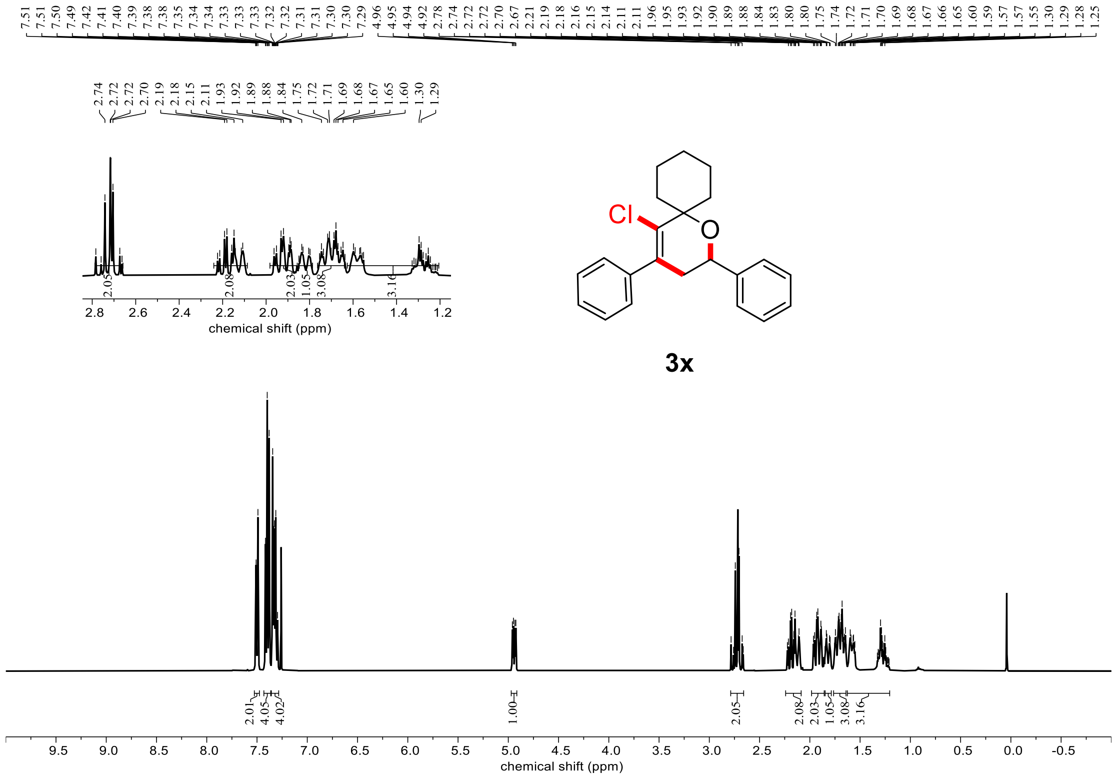

^{13}C NMR of 3x

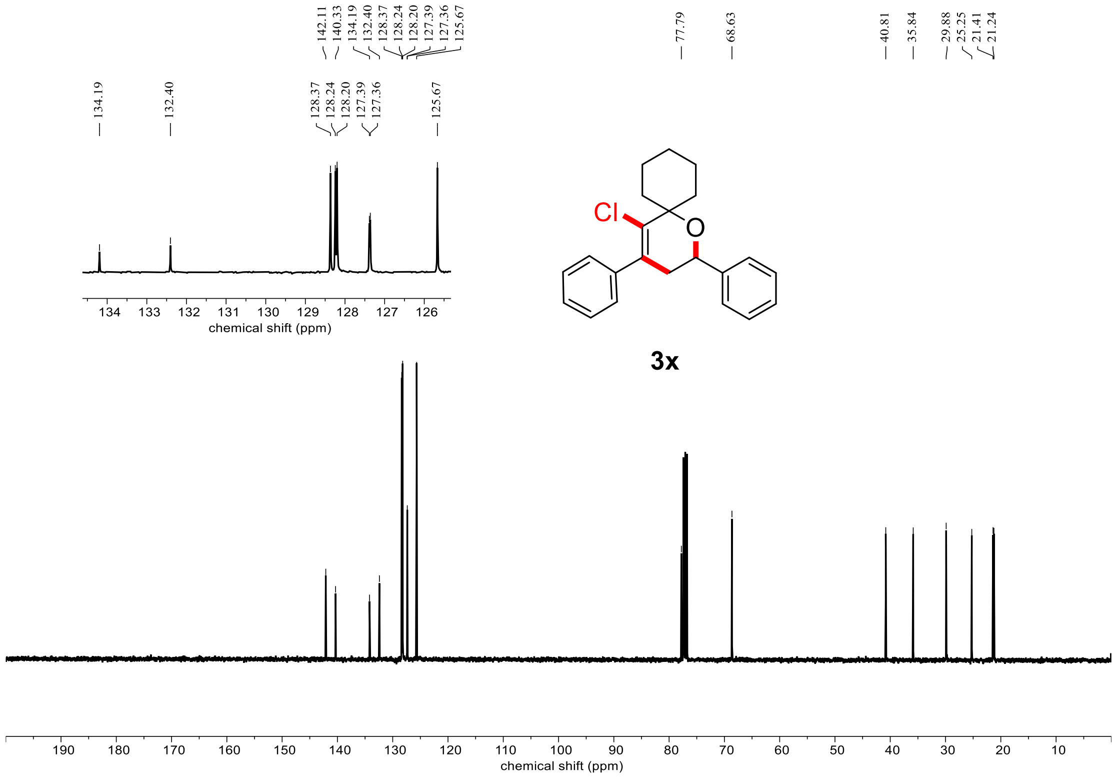

^{1}H and ^{13}C NMR spectra of compounds 4

^{1}H NMR of 4a

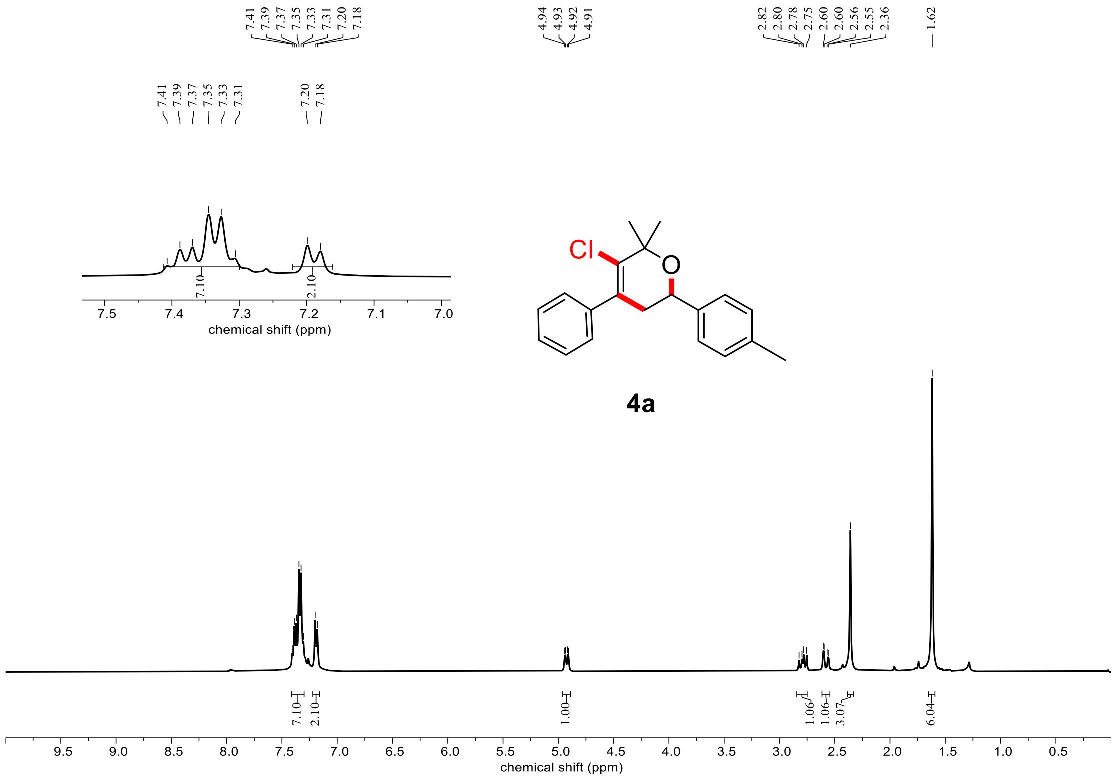

^{13}C NMR of 4a

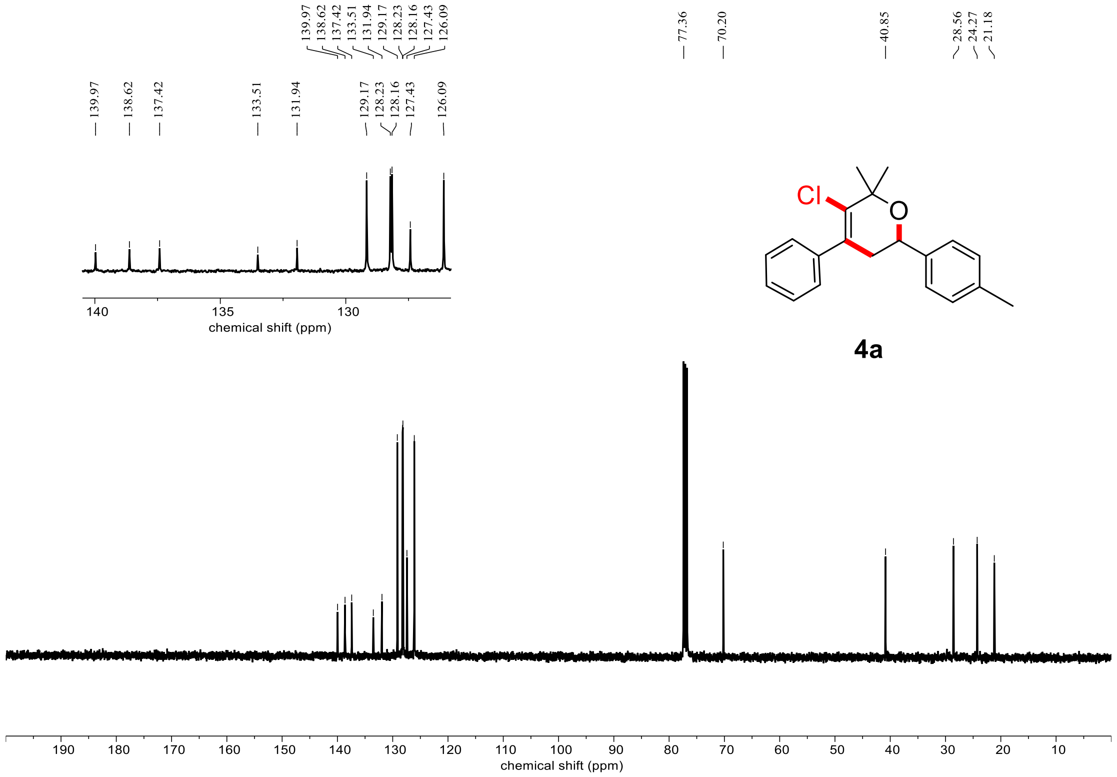

^{1}H NMR of 4b

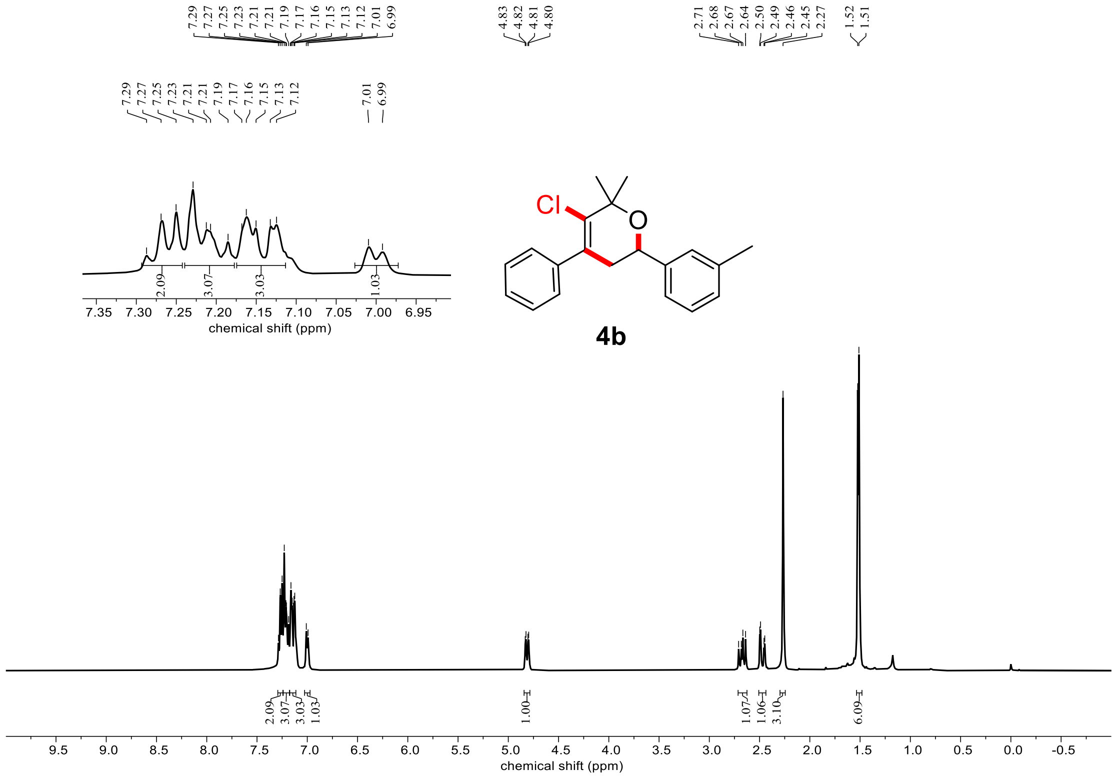

^{13}C NMR of 4b

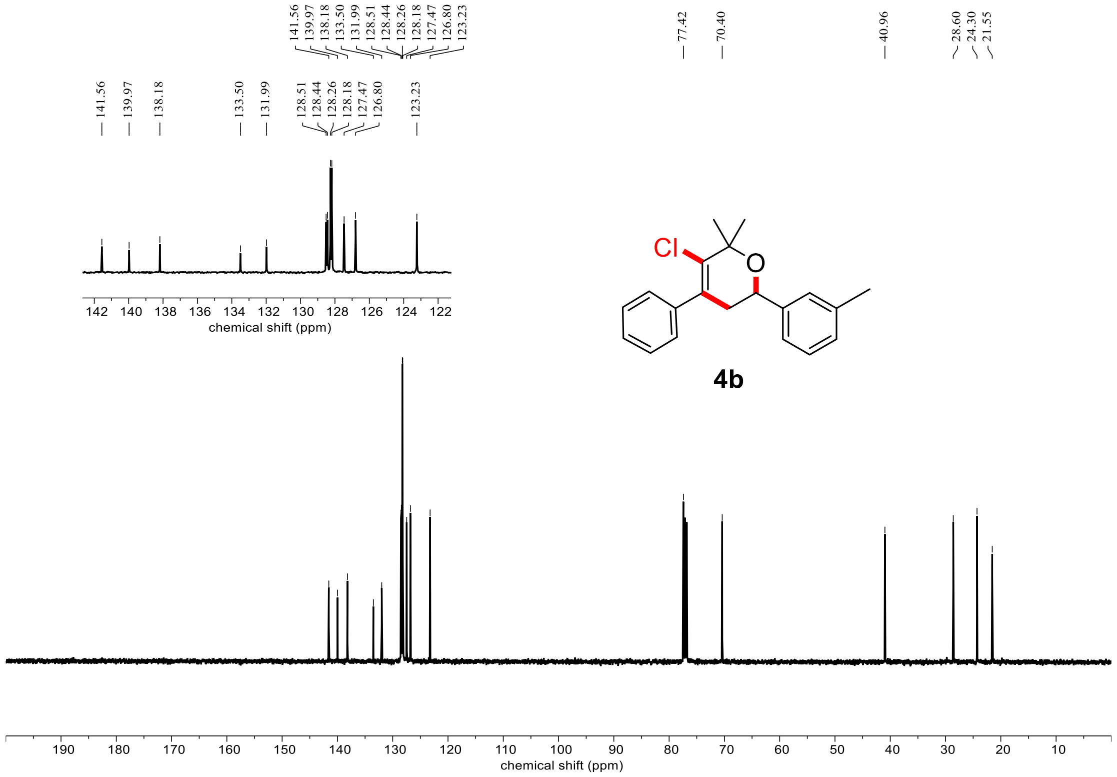

^{1}H NMR of 4c

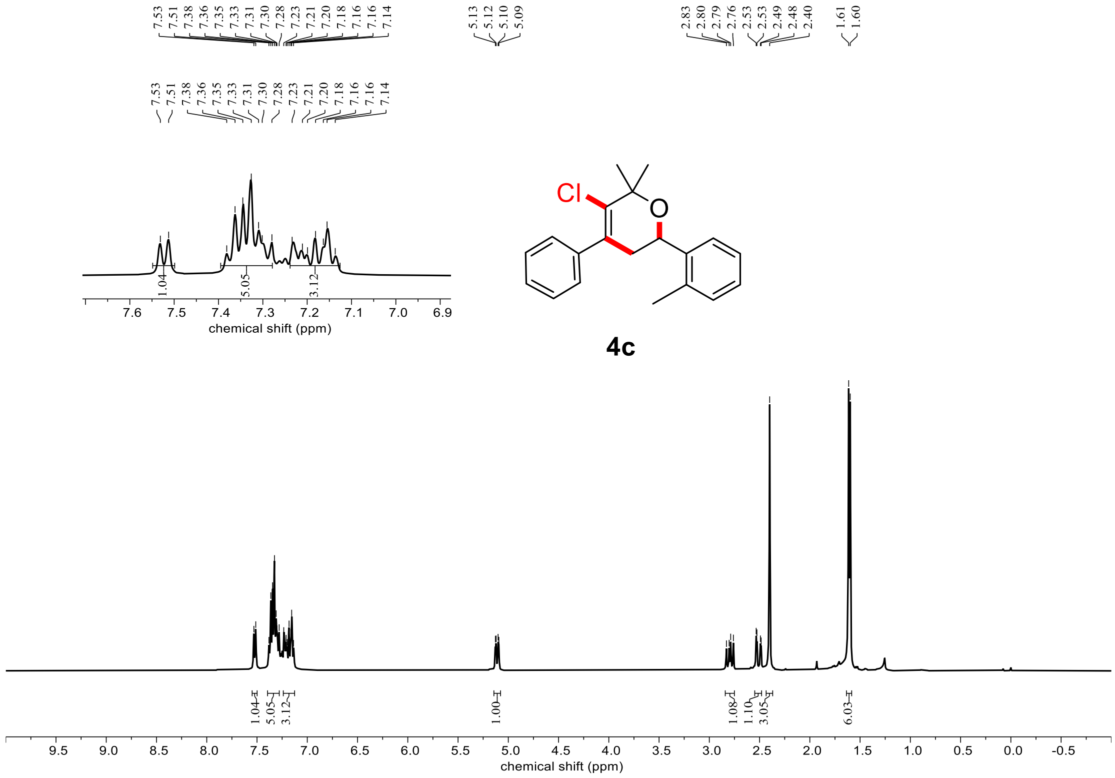

^{13}C NMR of 4c

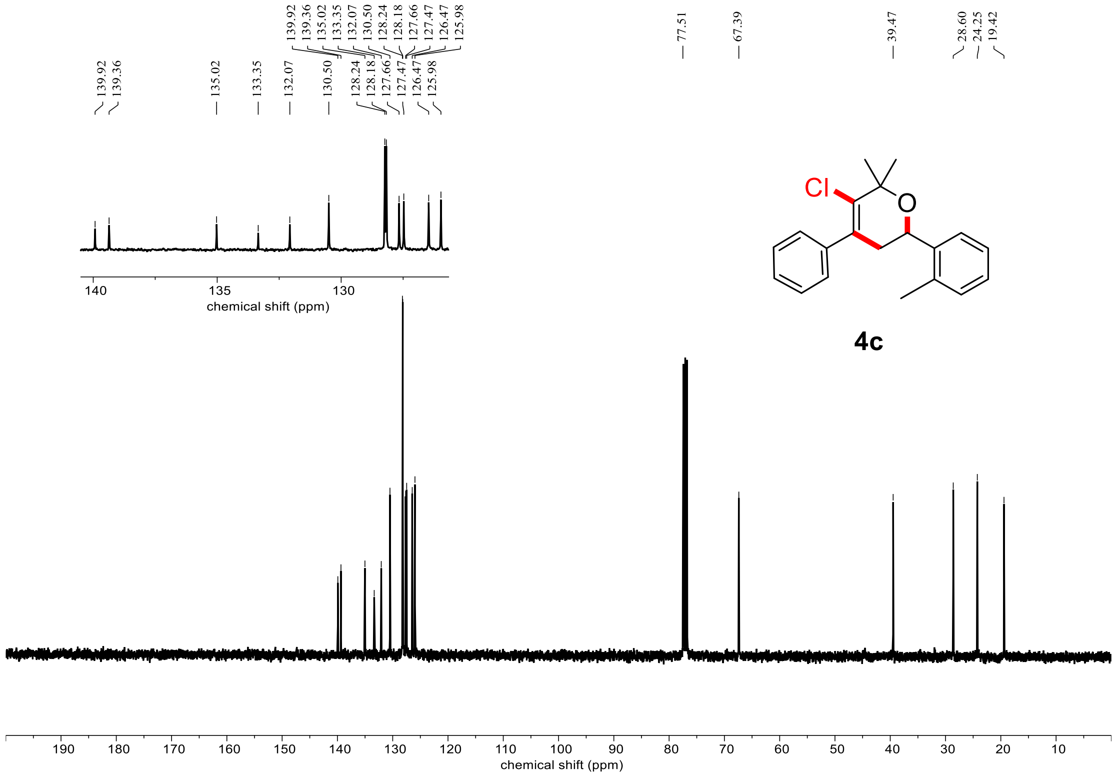

^{1}H NMR of 4d

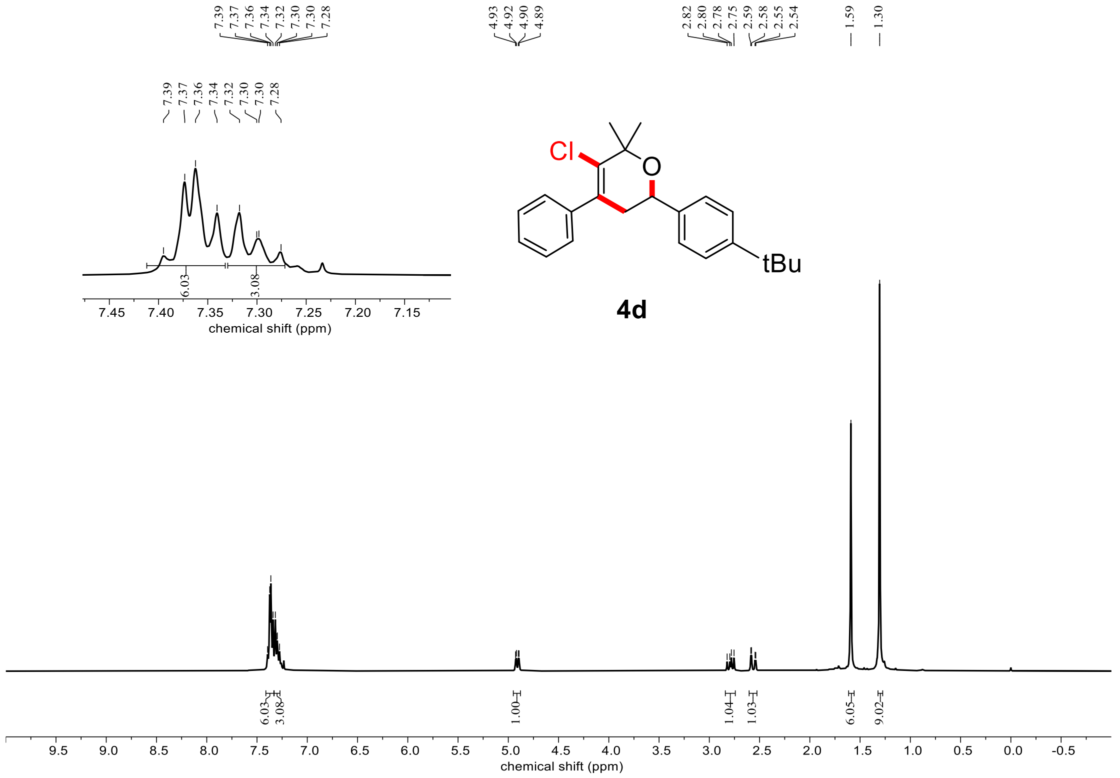

^{13}C NMR of 4d

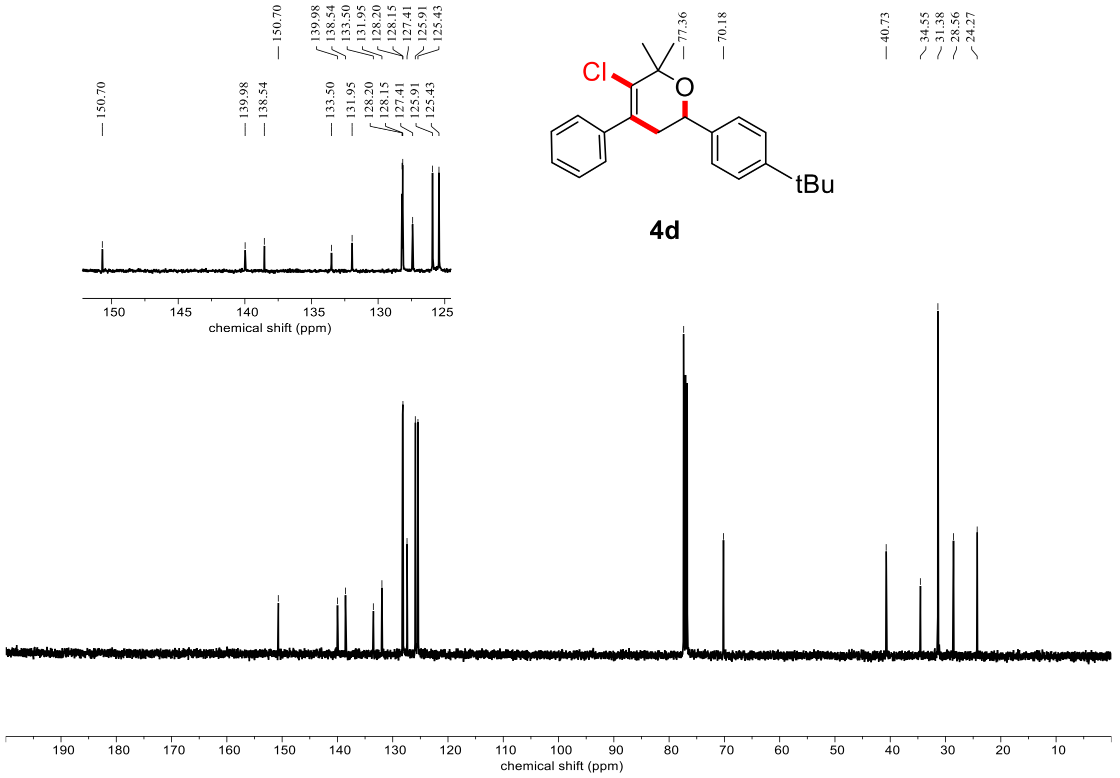

^{1}H NMR of 4e

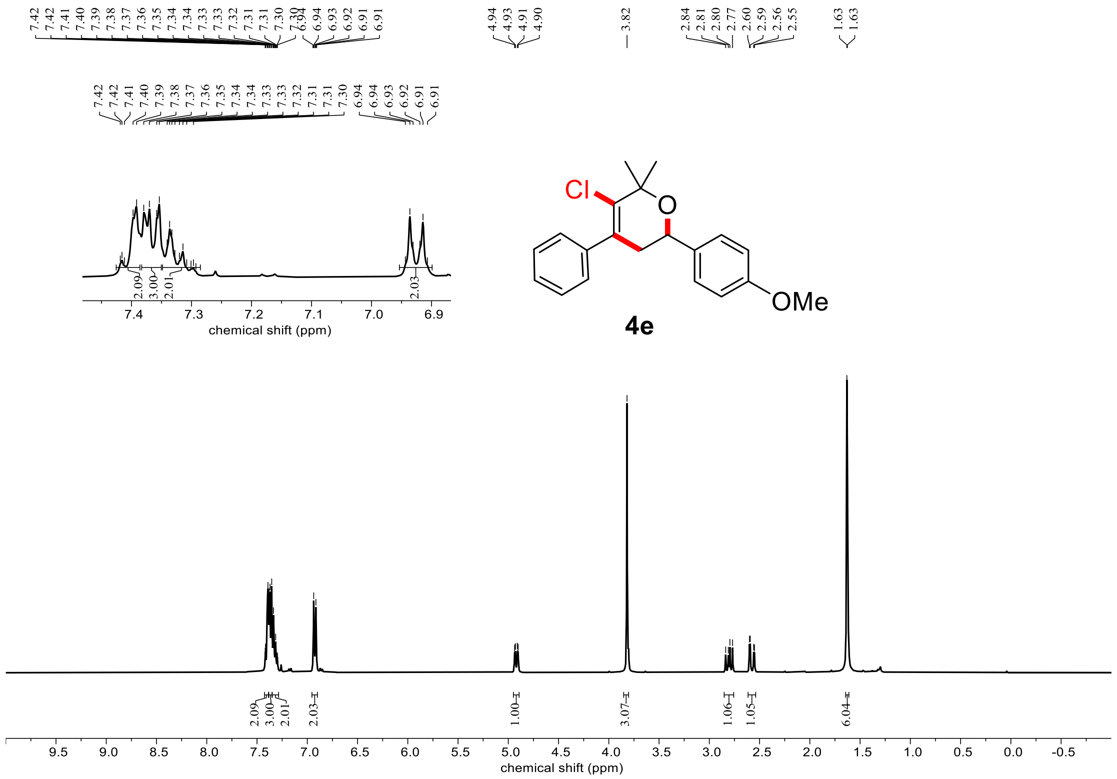

^{13}C NMR of 4e

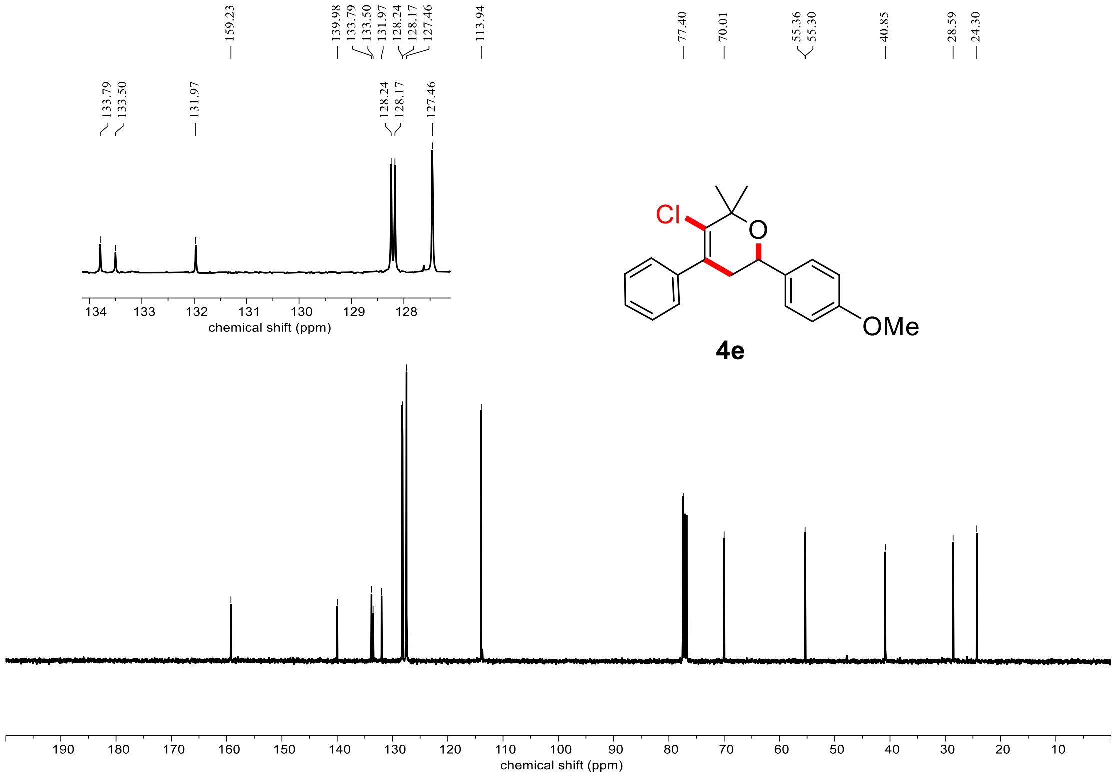

^{1}H NMR of 4f

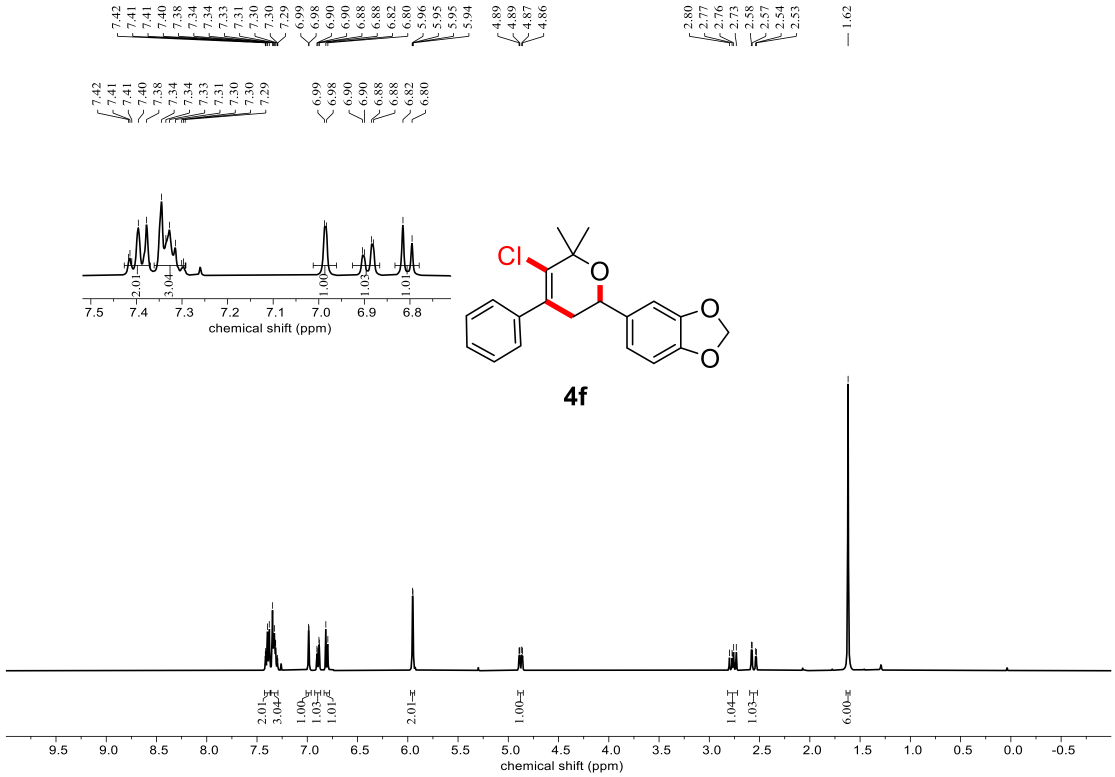

^{13}C NMR of 4f

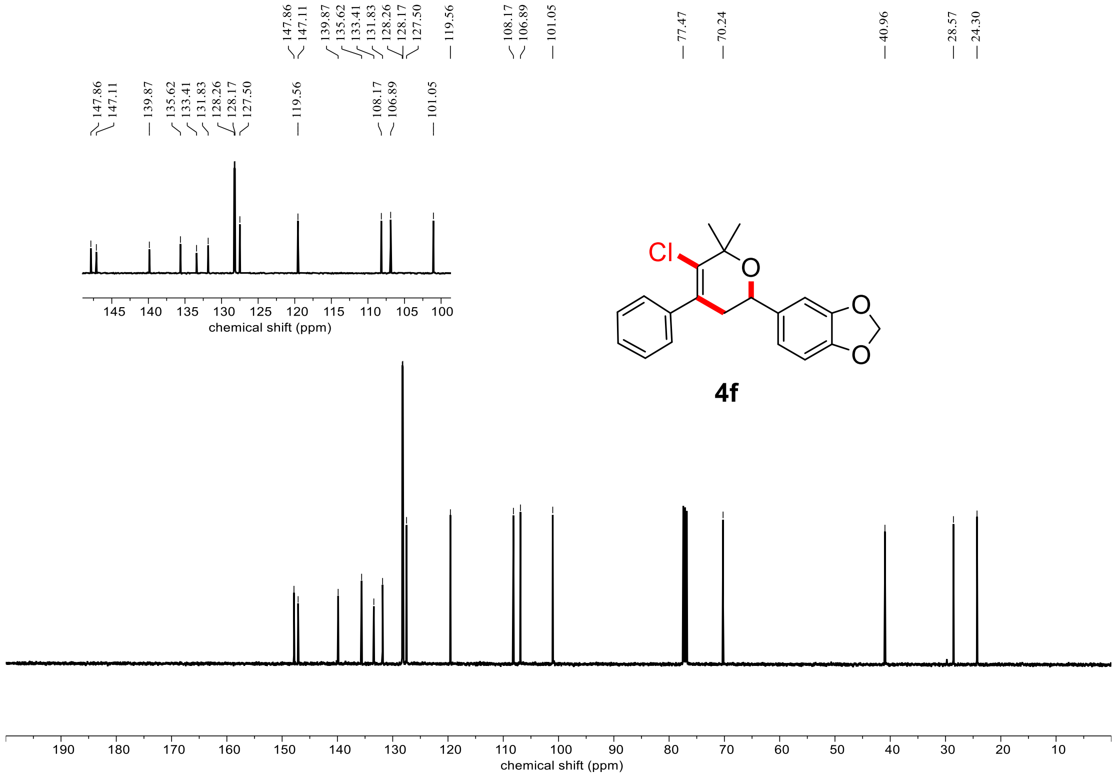

^{1}H NMR of 4g

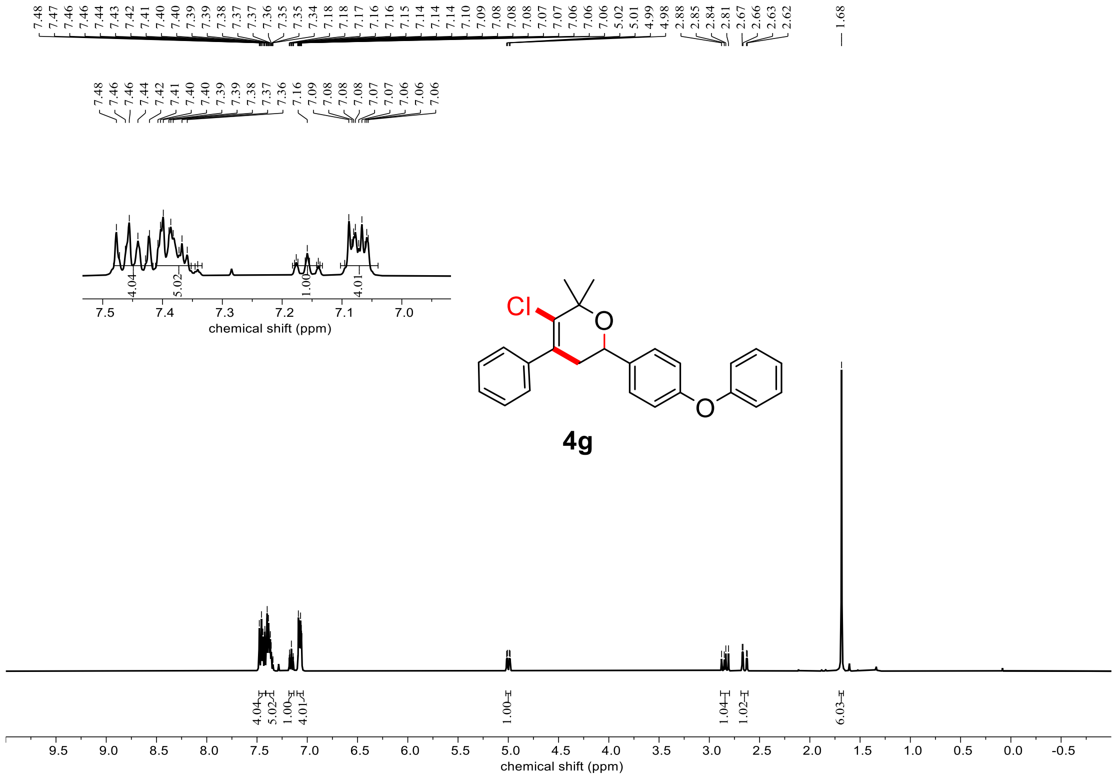

^{13}C NMR of 4g

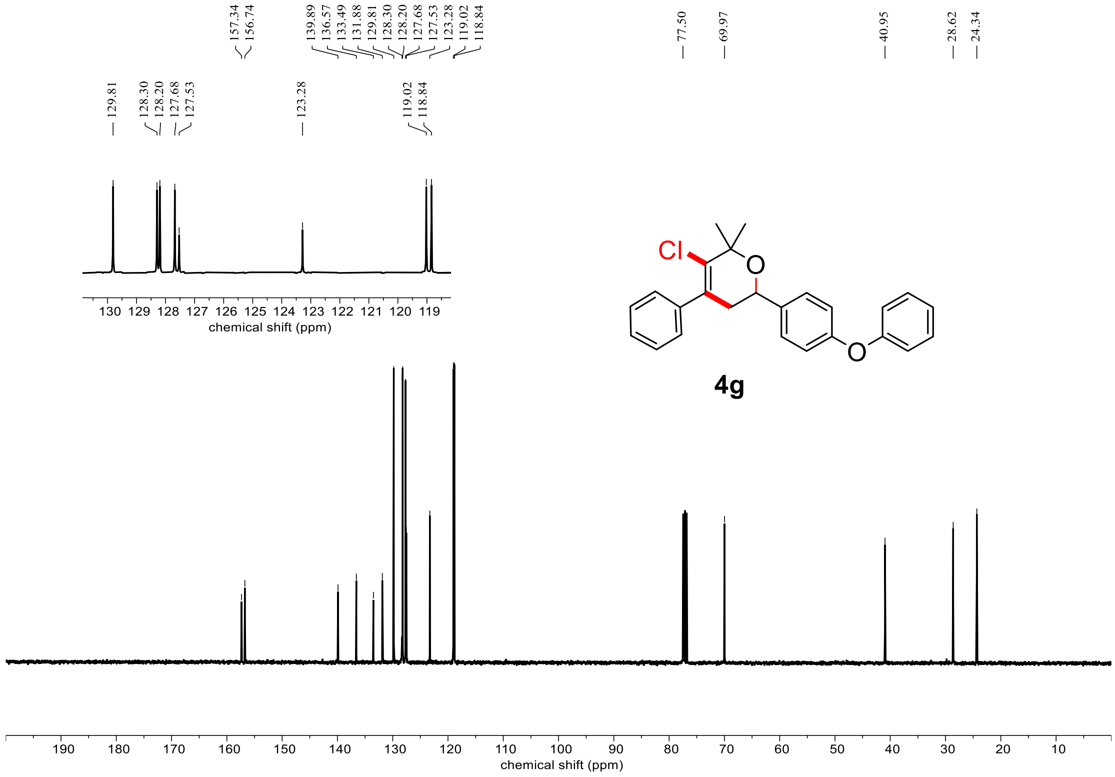

^{1}H NMR of 4h

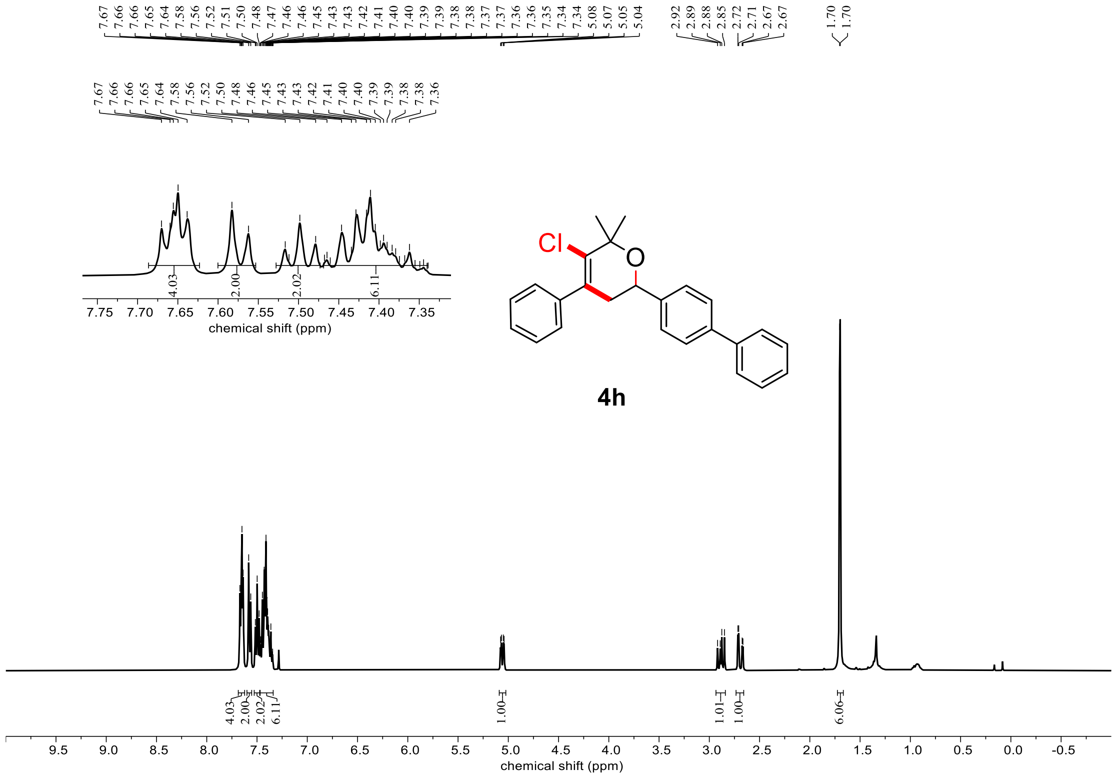

^{13}C NMR of 4h

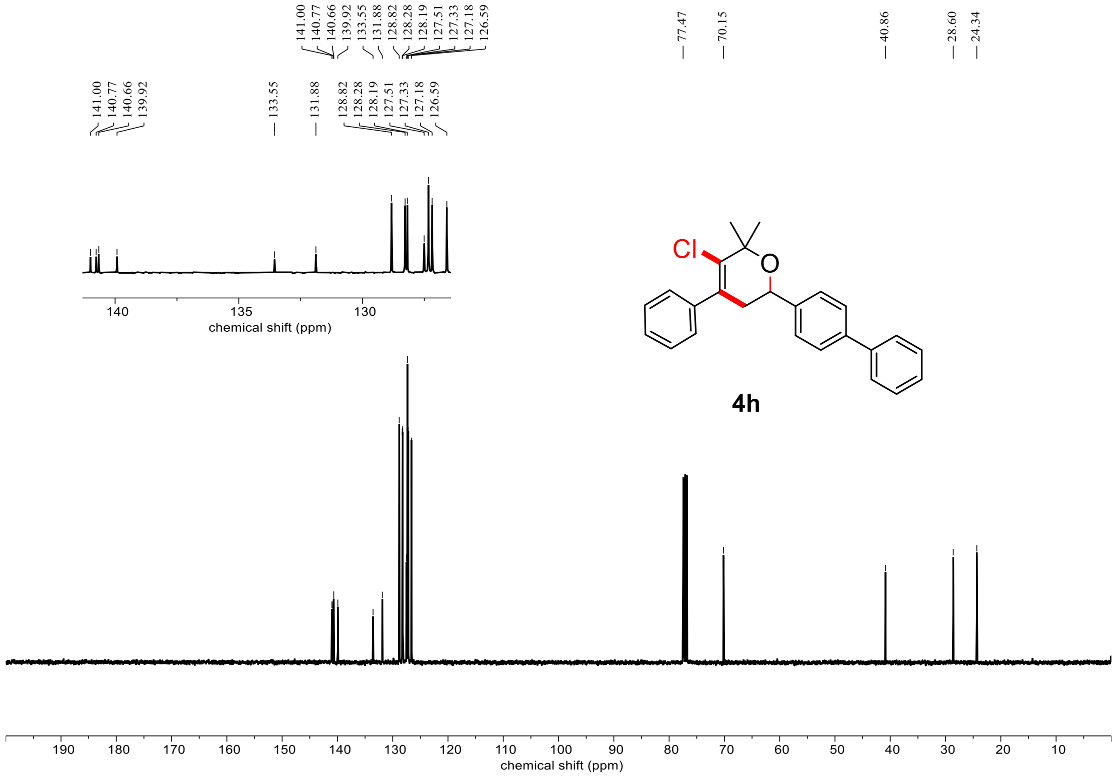

^{1}H NMR of 4i

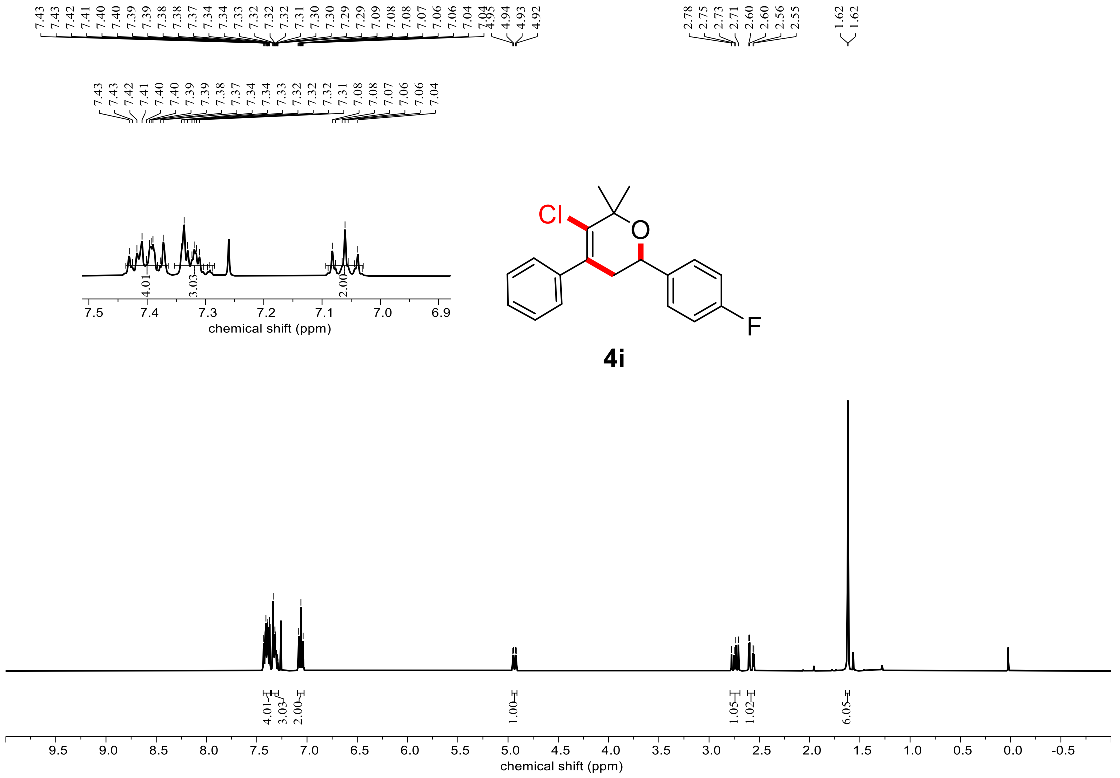

^{13}C NMR of 4i

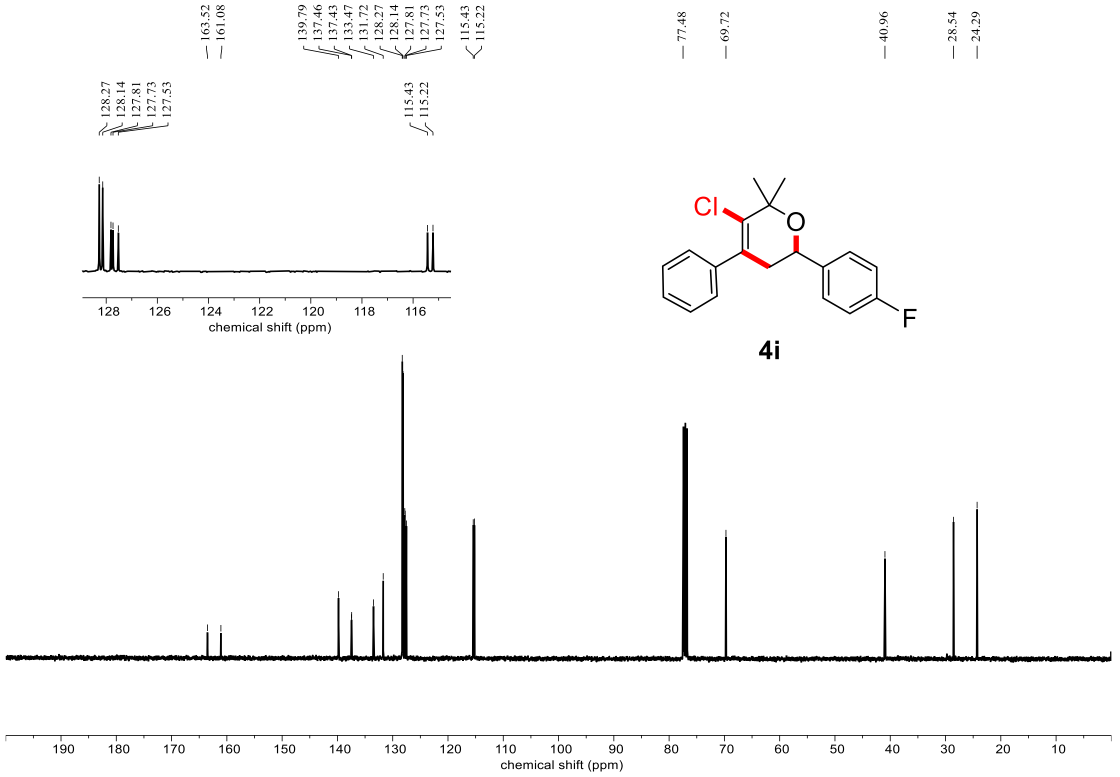

^{19}F NMR of 4i

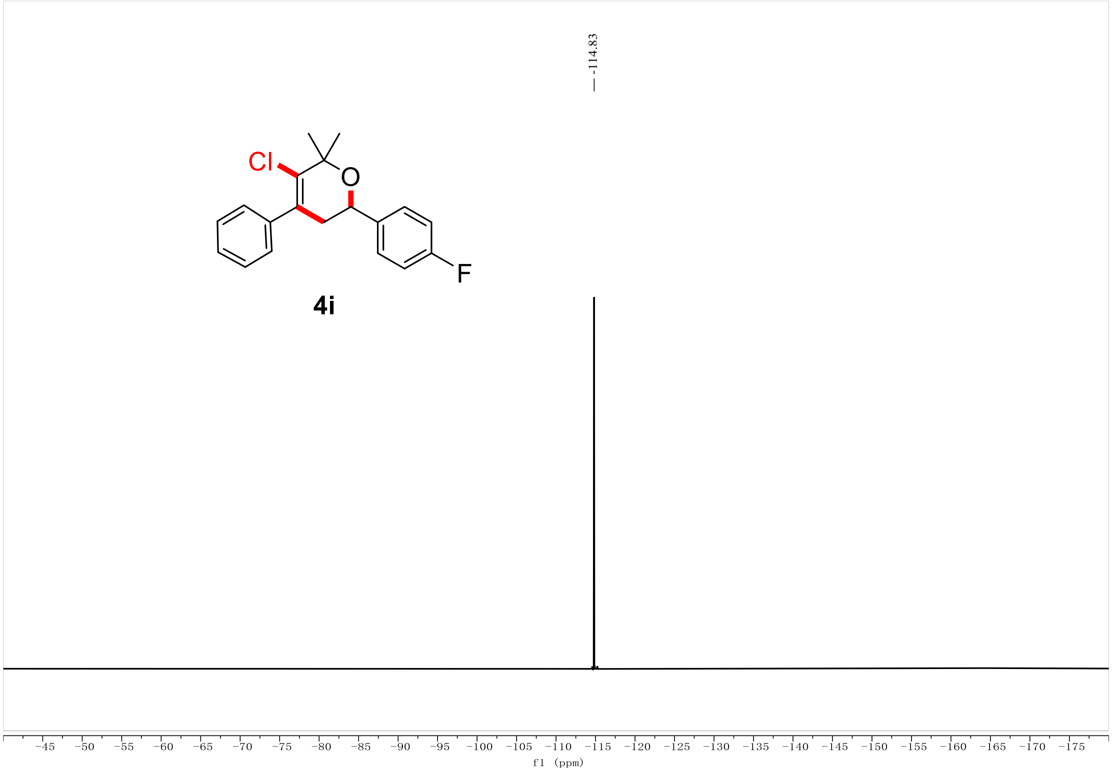
-114.83
Cl
O
F
4i
-45
-50
-55
-60
-65
-70
-75
-80
-85
-90
-95
-100
-105
-110
-115
-120
-125
-130
-135
-140
-145
-150
-155
-160
-165
-170
-175
f1 (ppm)

^{1}H NMR of 4j

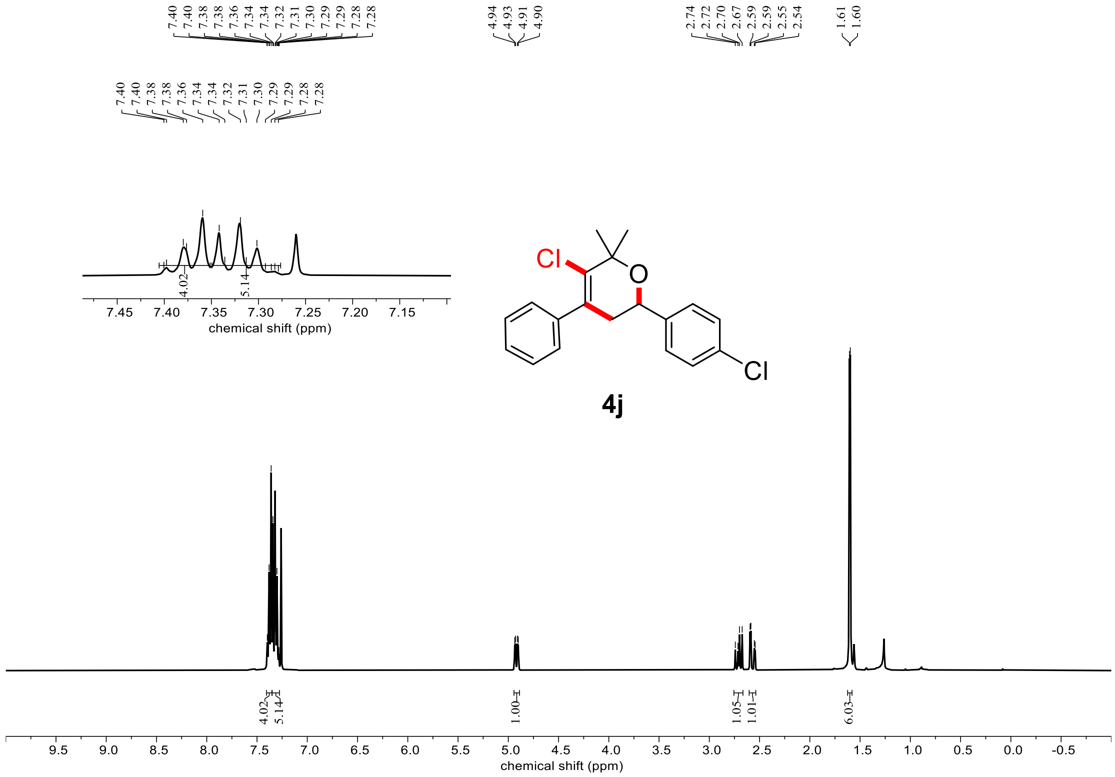

^{13}C NMR of 4j

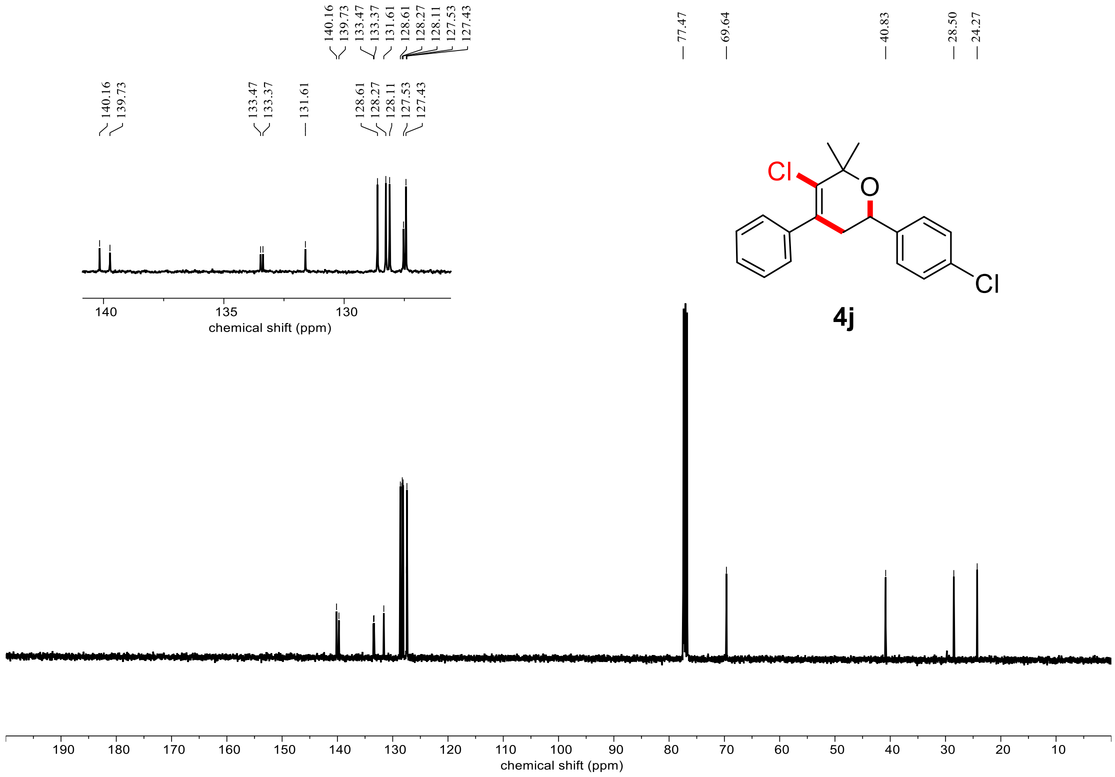

^{1}H NMR of 4k

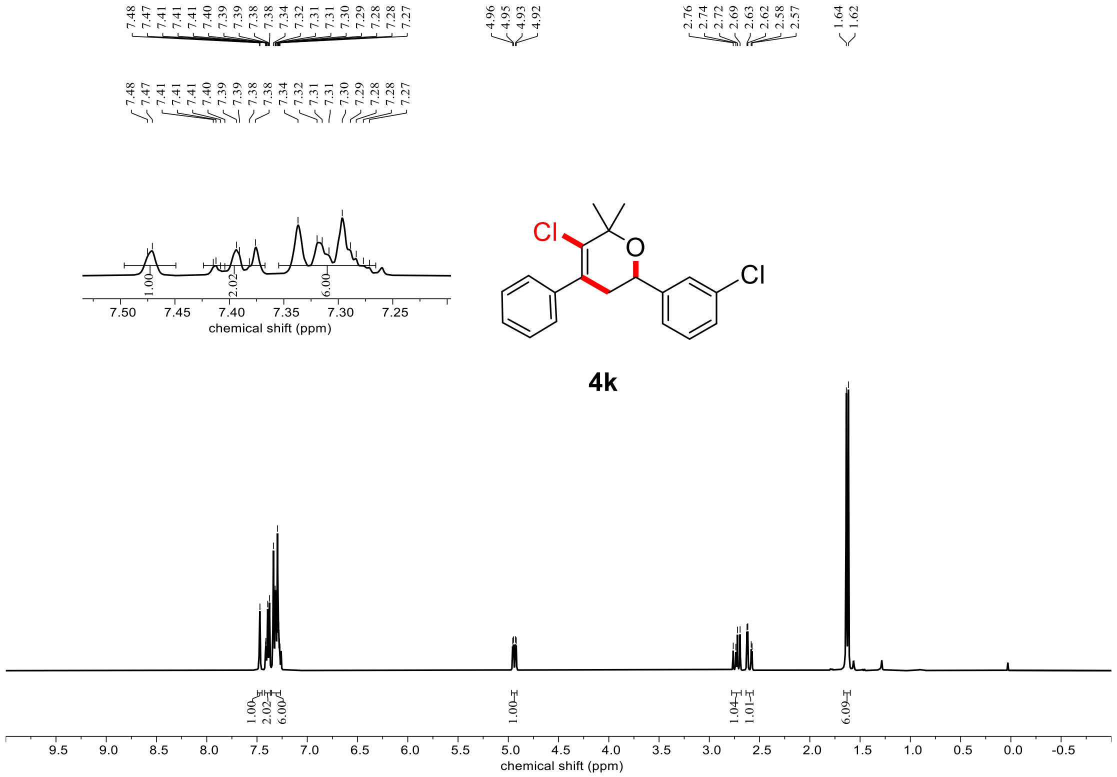

^{13}C NMR of 4k

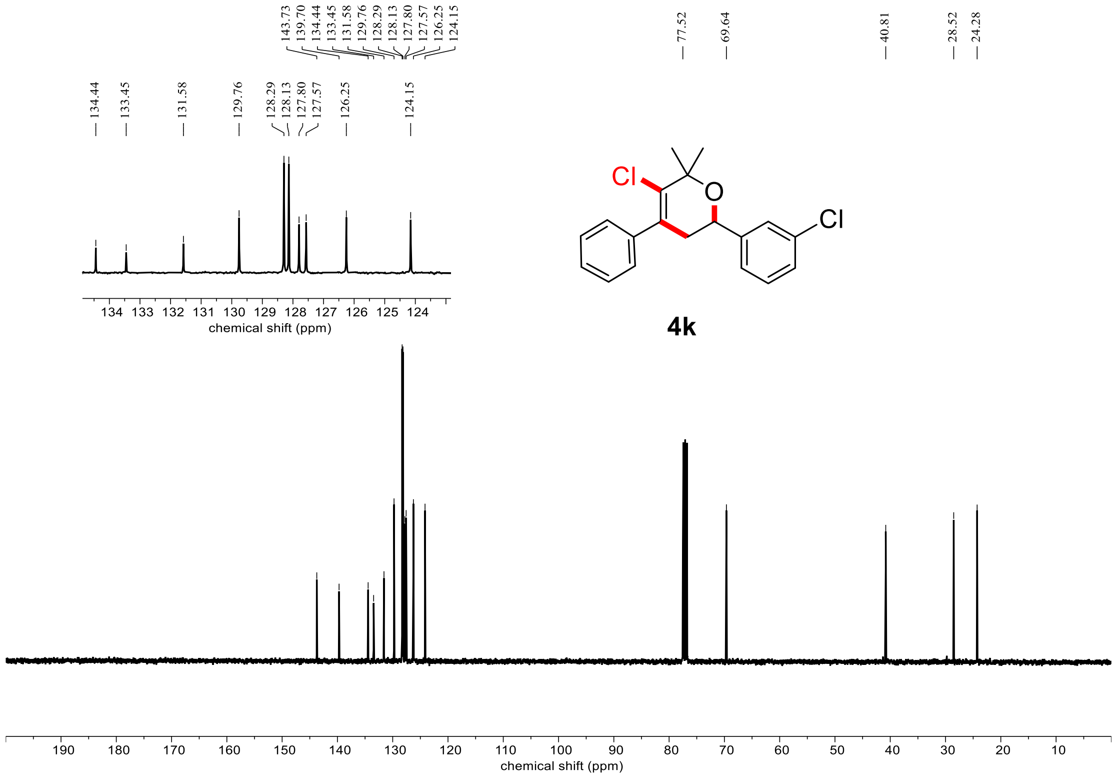

^{1}H NMR of 4l

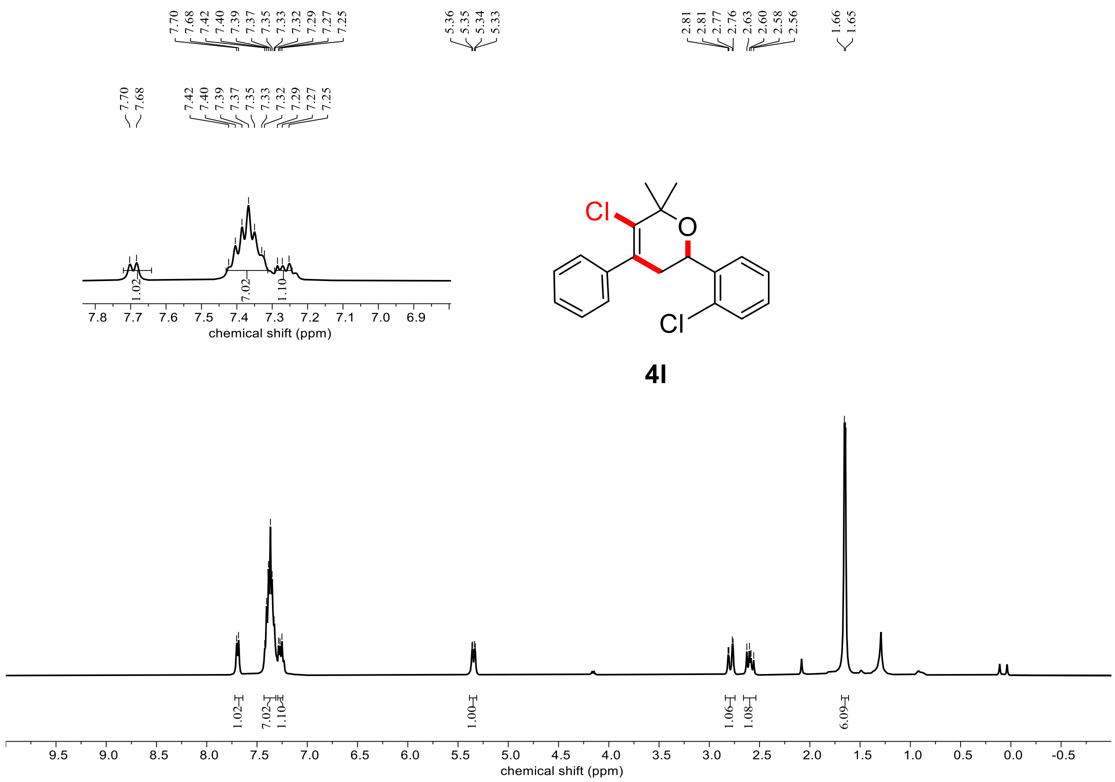

^{13}C NMR of 4l

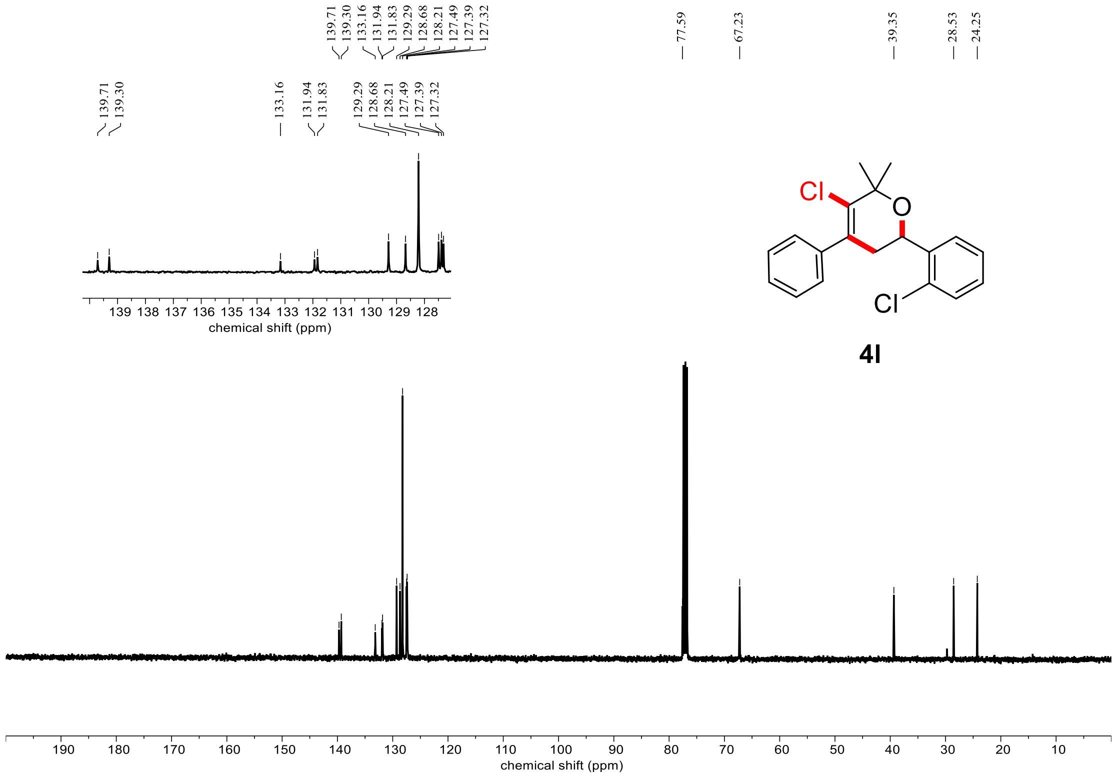

^{1}H NMR of 4m

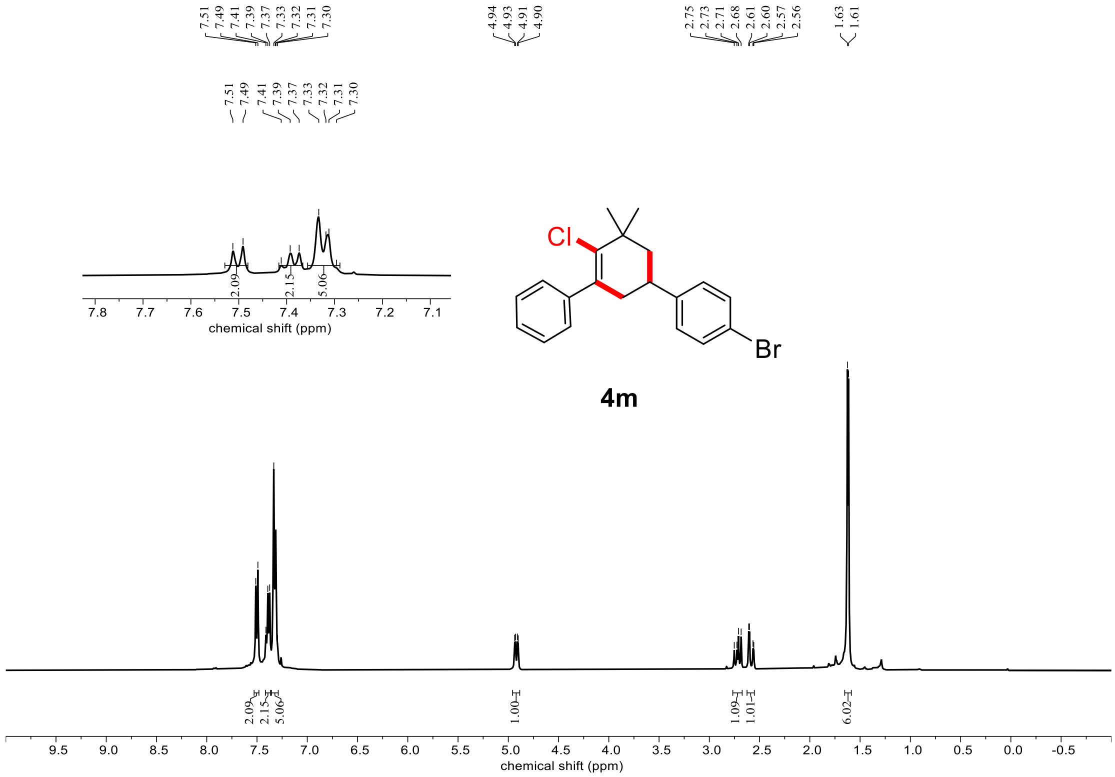

^{13}C NMR of 4m

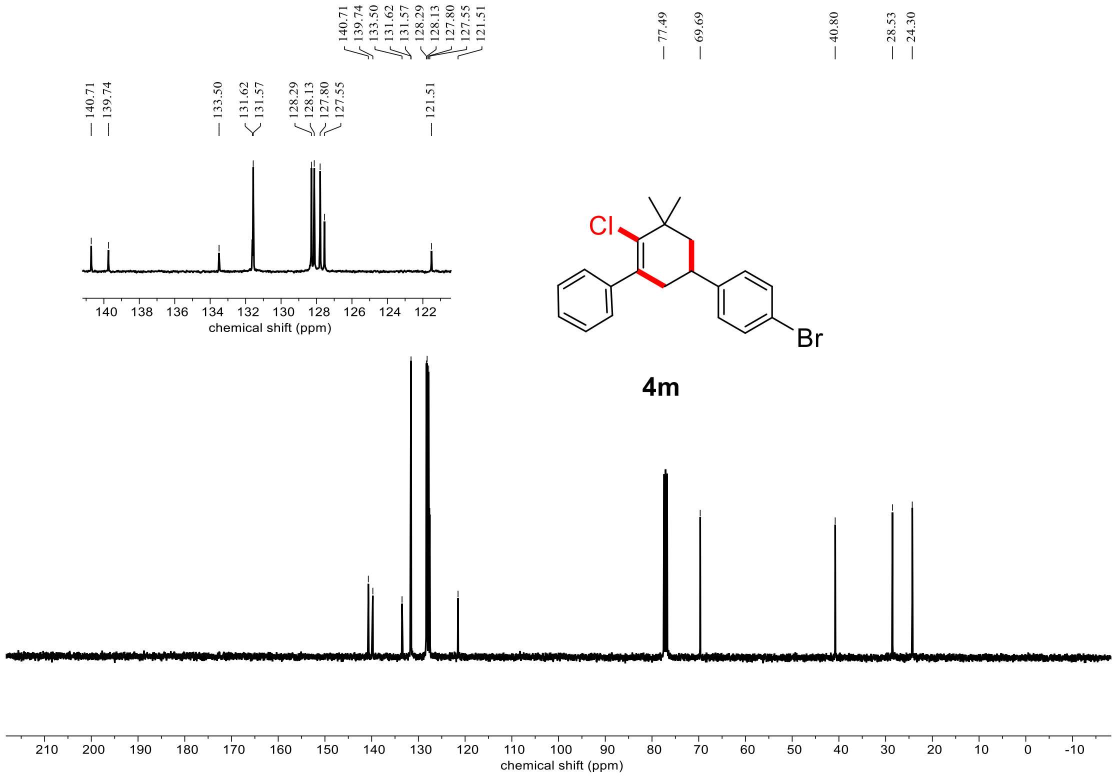

^{1}H NMR of 4n

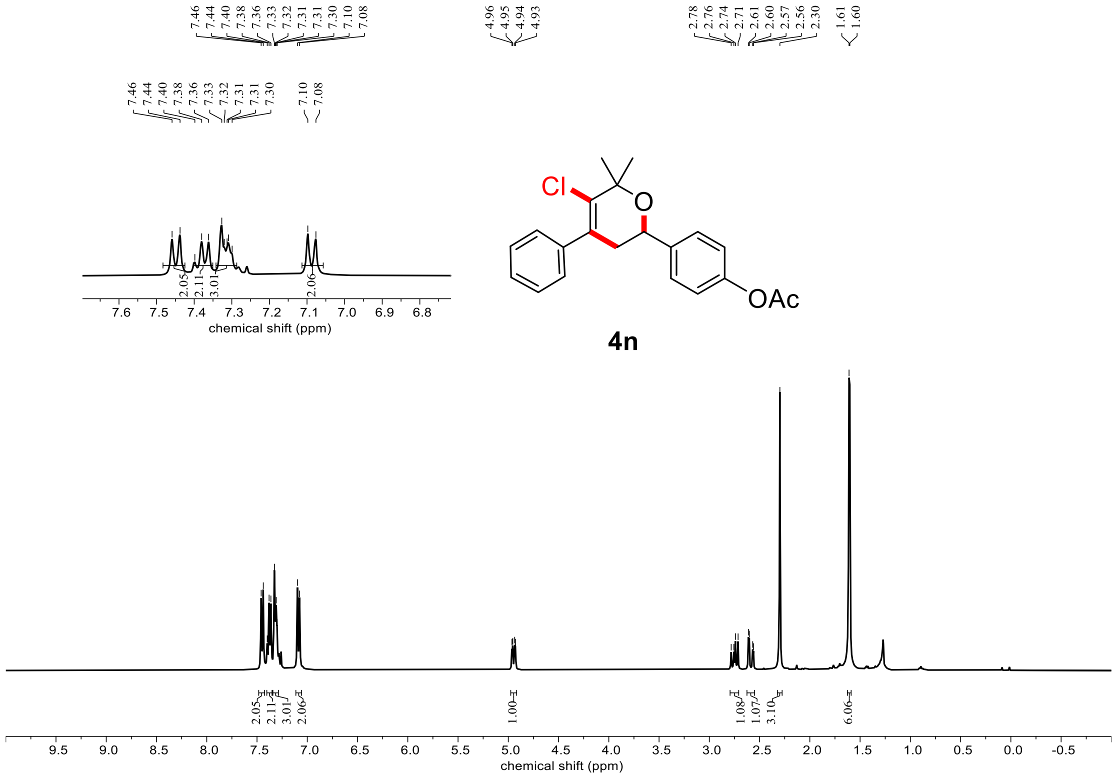

^{13}C NMR of 4n

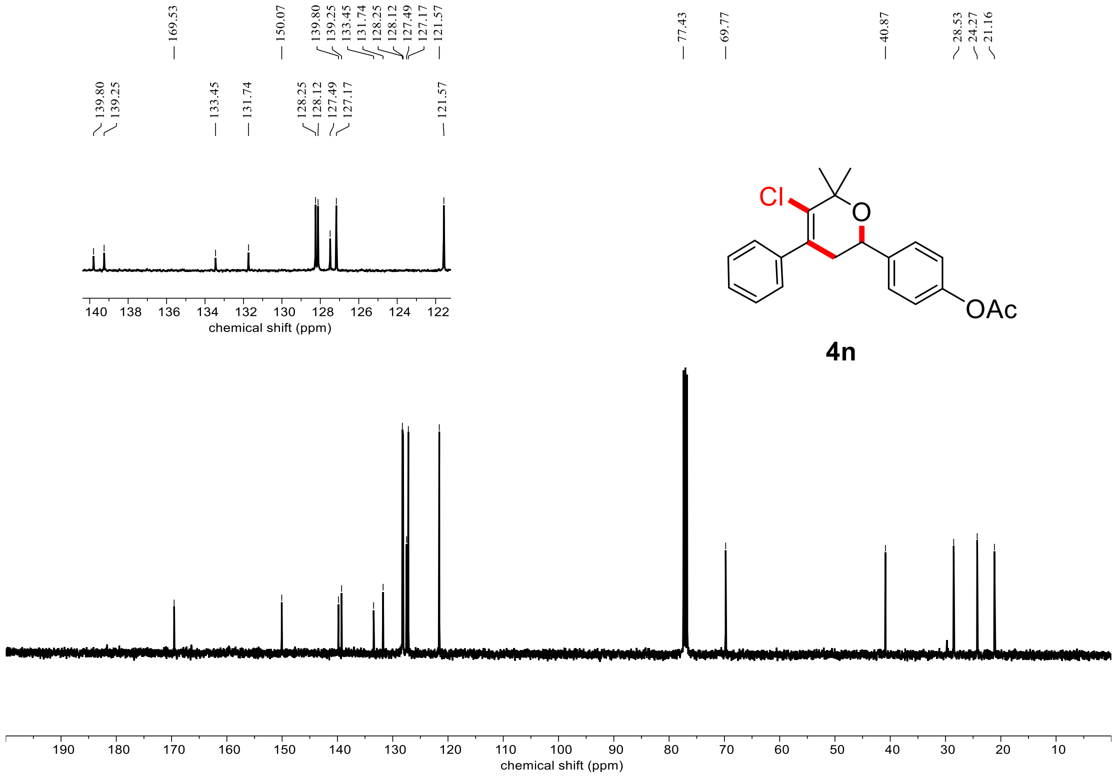

^{1}H NMR of 4o

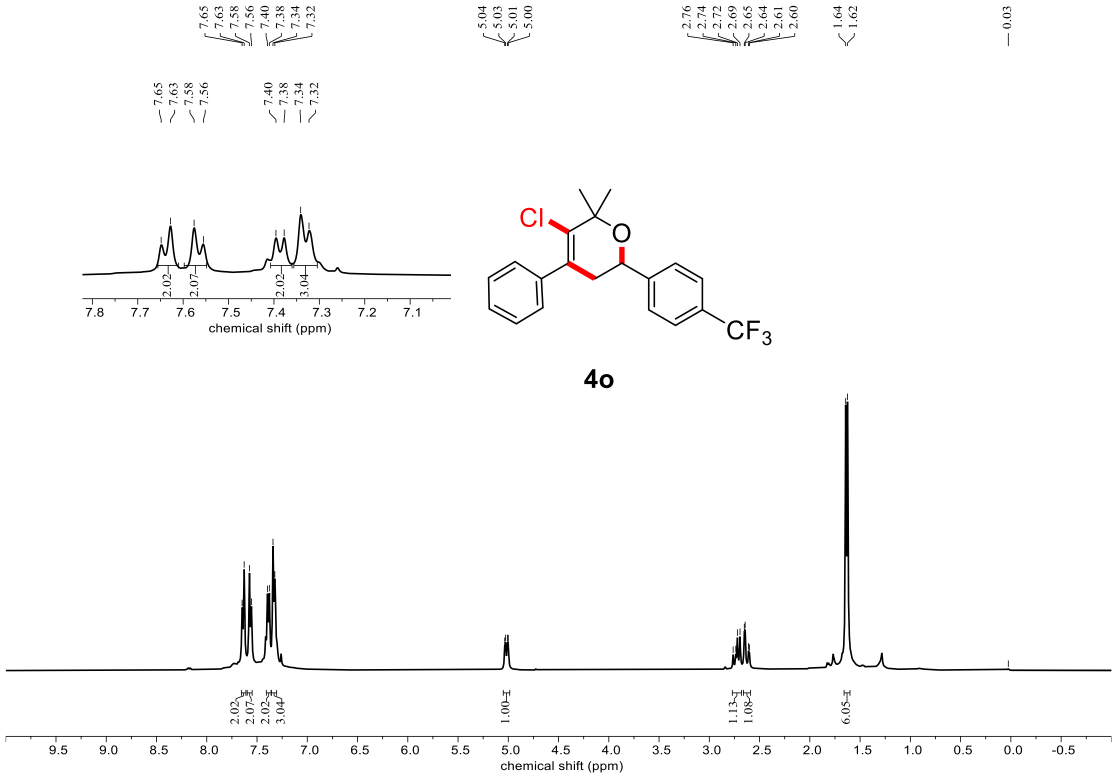

^{13}C NMR of 4o

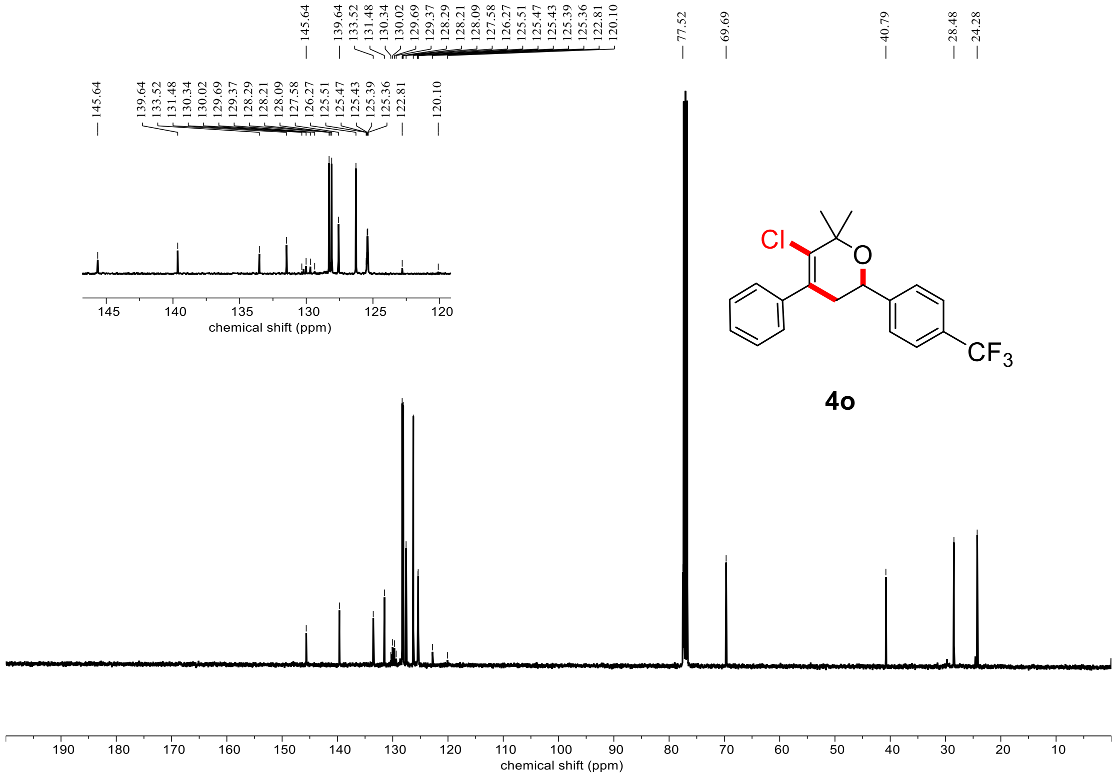

^{19}F NMR of 4o

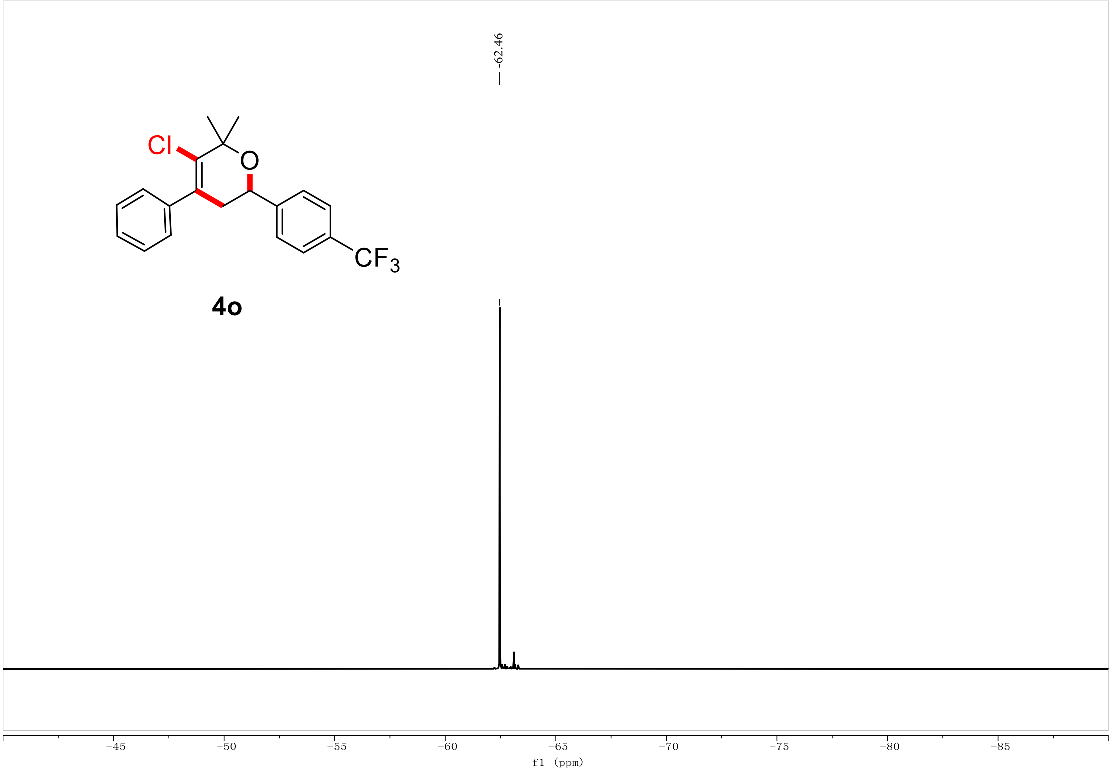

^{1}H NMR of 4p

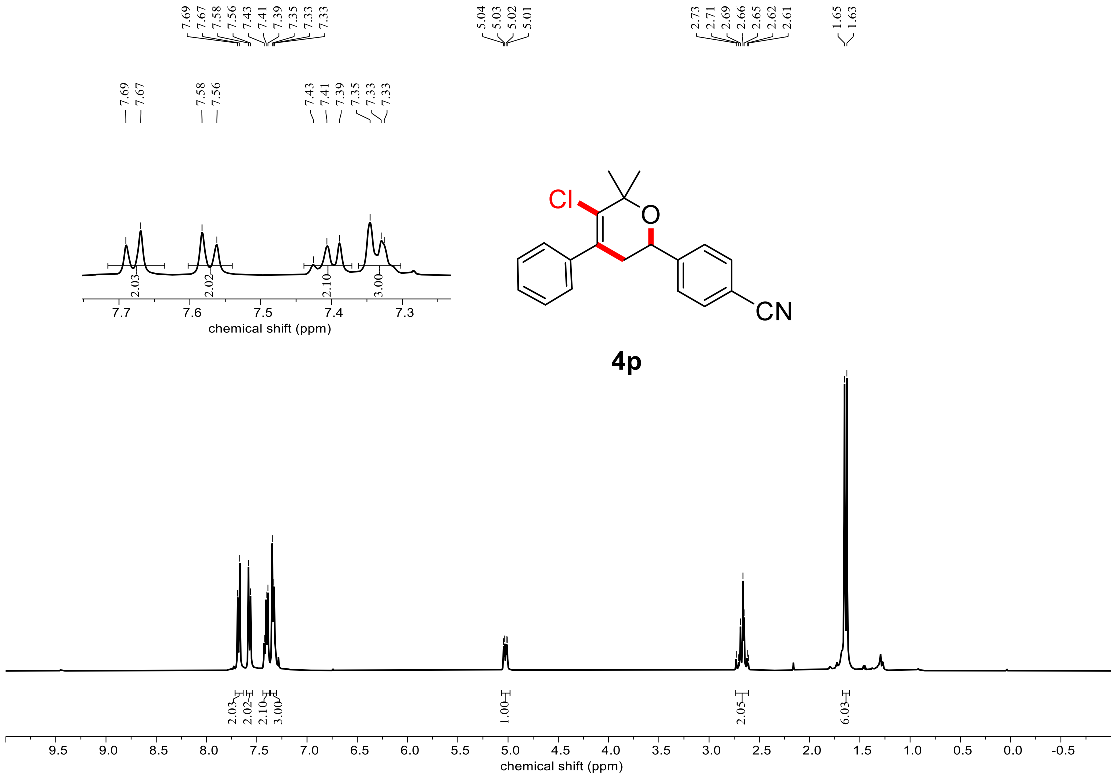

^{13}C NMR of 4p

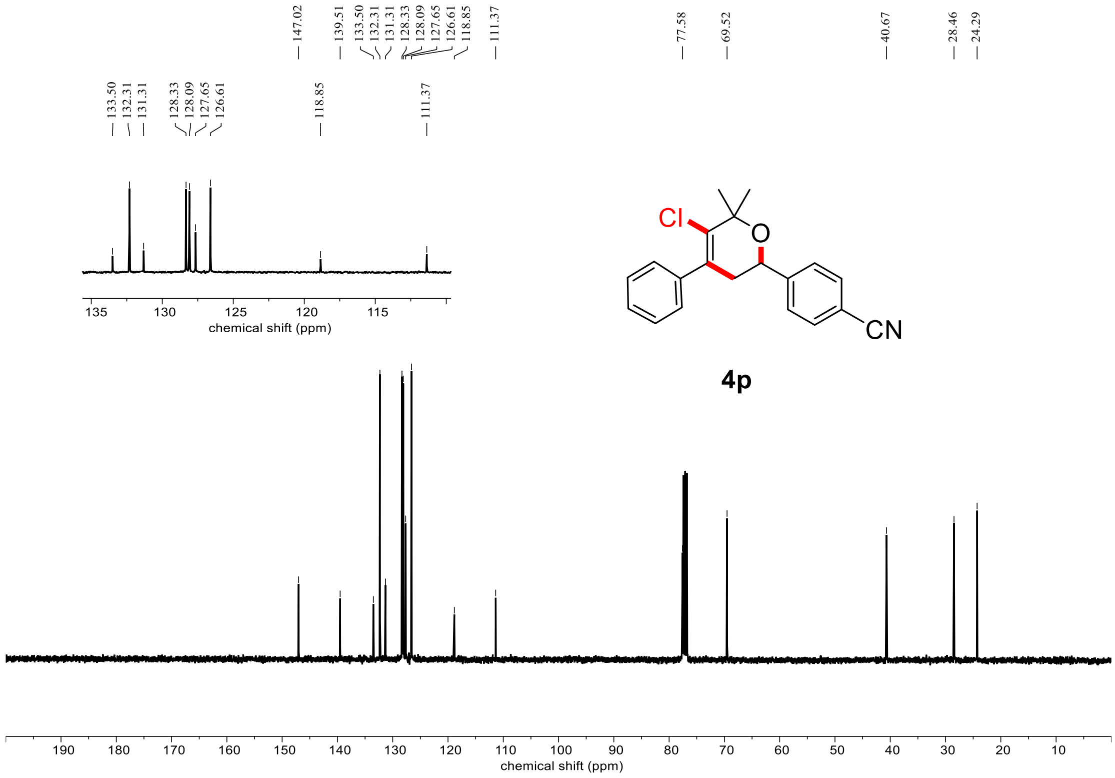

^{1}H NMR of 4q

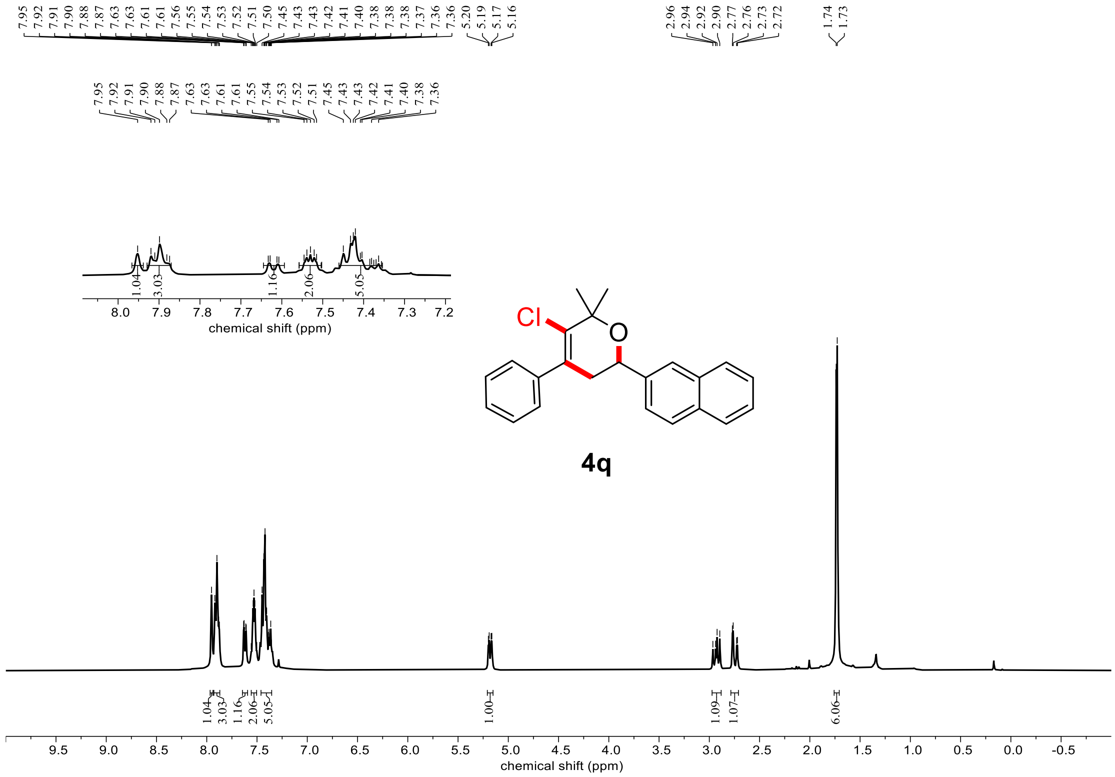

^{13}C NMR of 4q

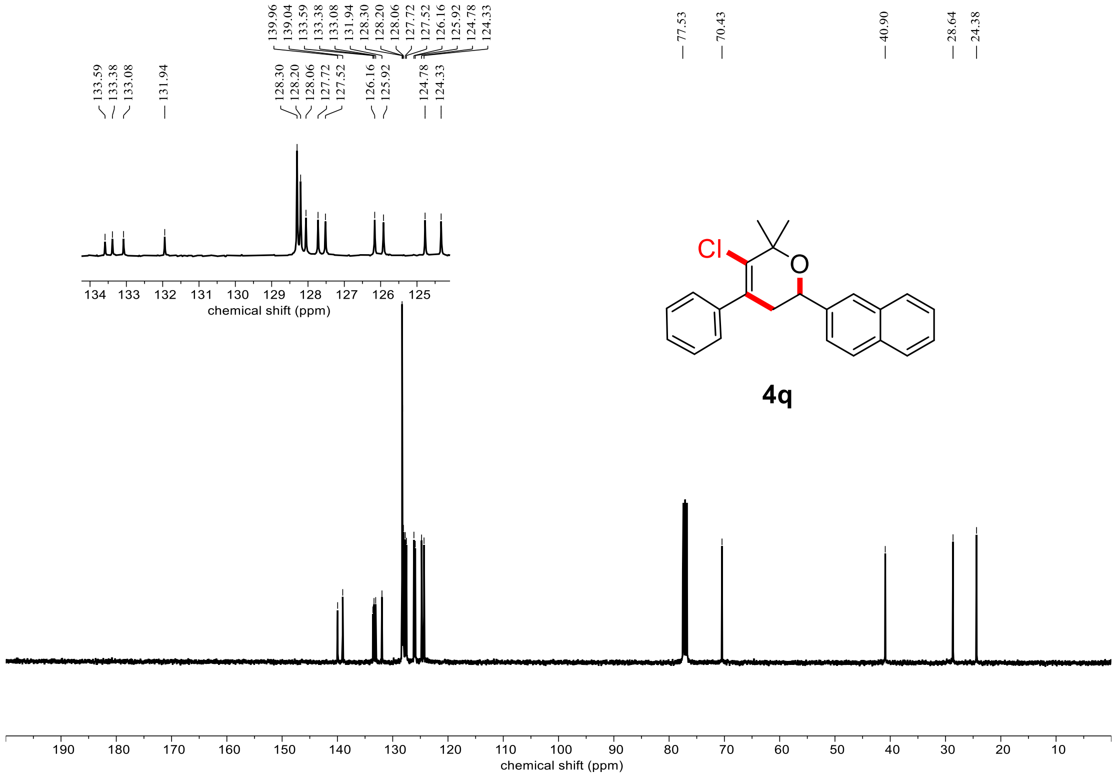

^{1}H NMR of 4r

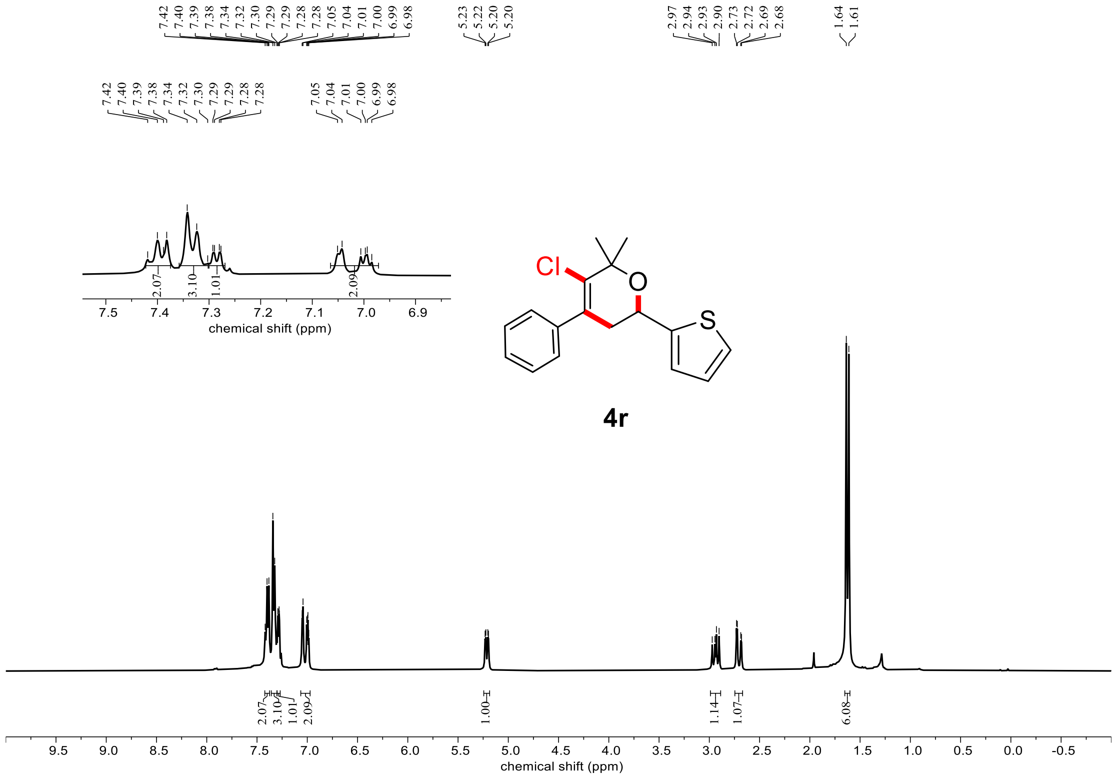

^{13}C NMR of 4r

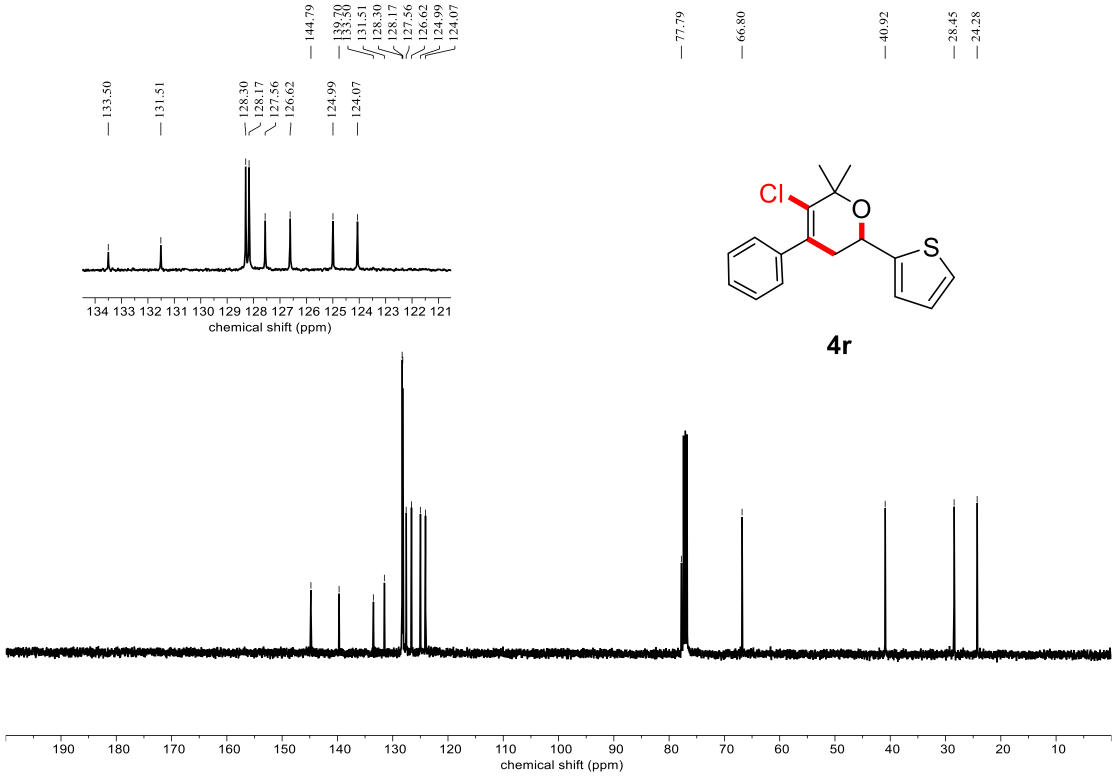

^{1}H and ^{13}C NMR spectra of compounds 5

^{1}H NMR of 5a

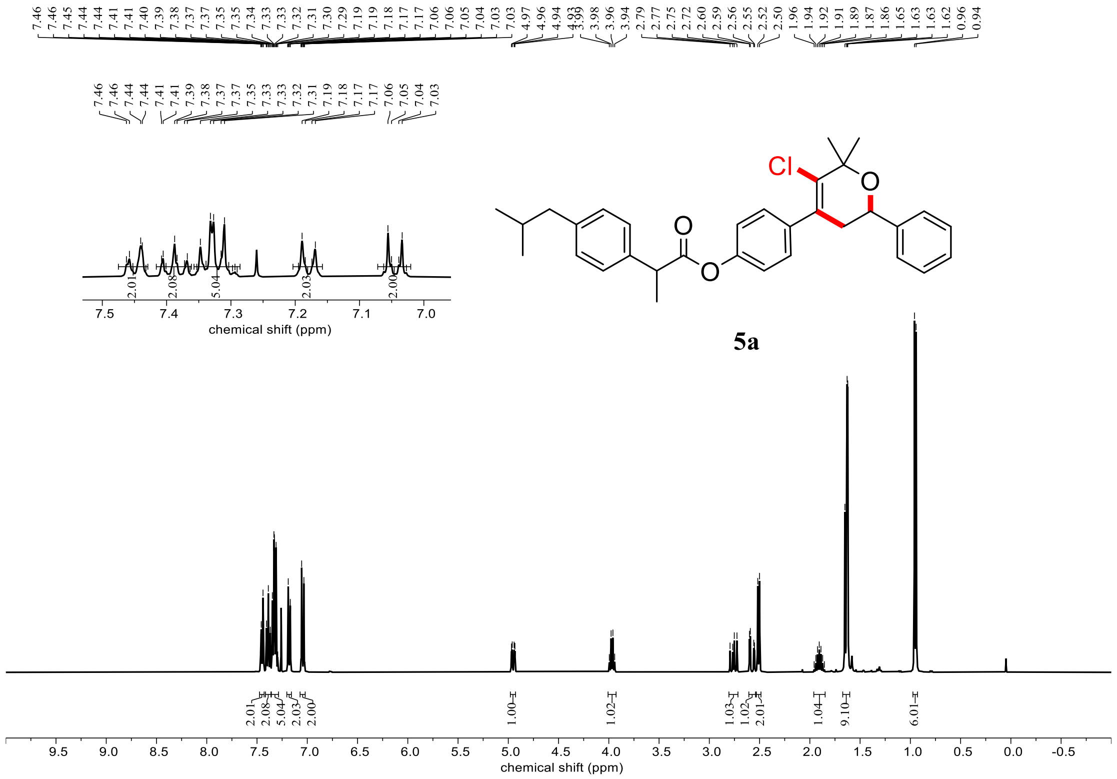

^{13}C NMR of 5a

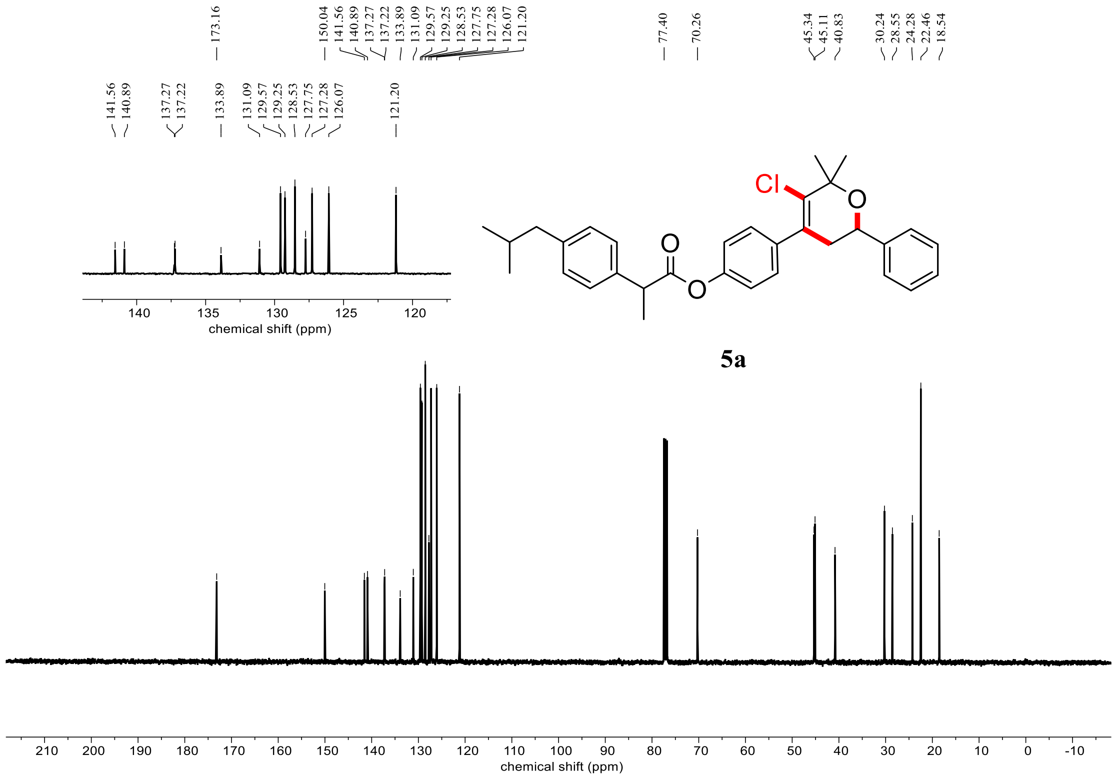

^{1}H NMR of 5b

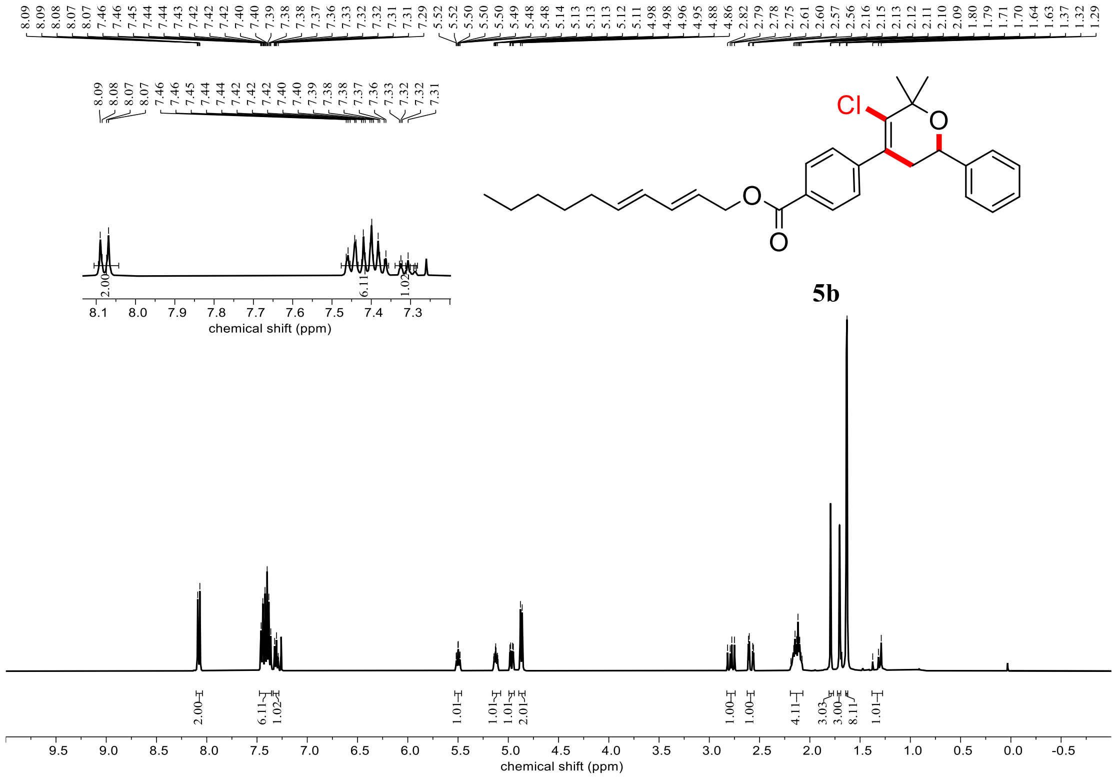

^{13}C NMR of 5b

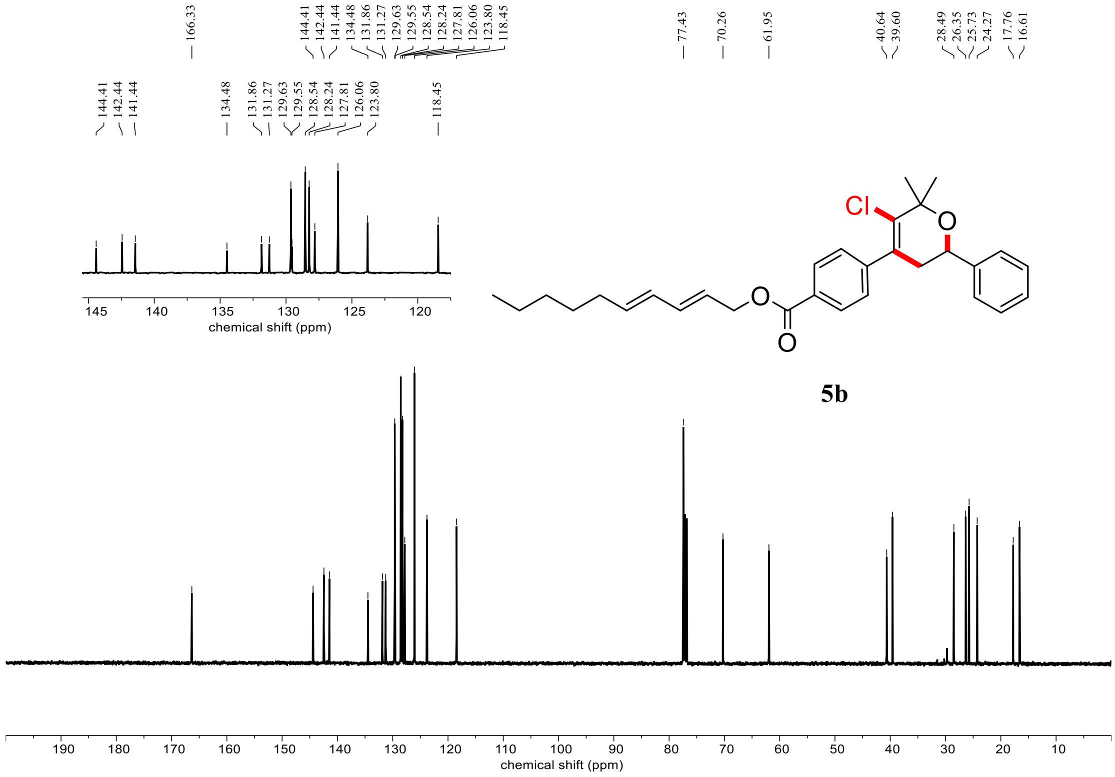

^{1}H NMR of 5c

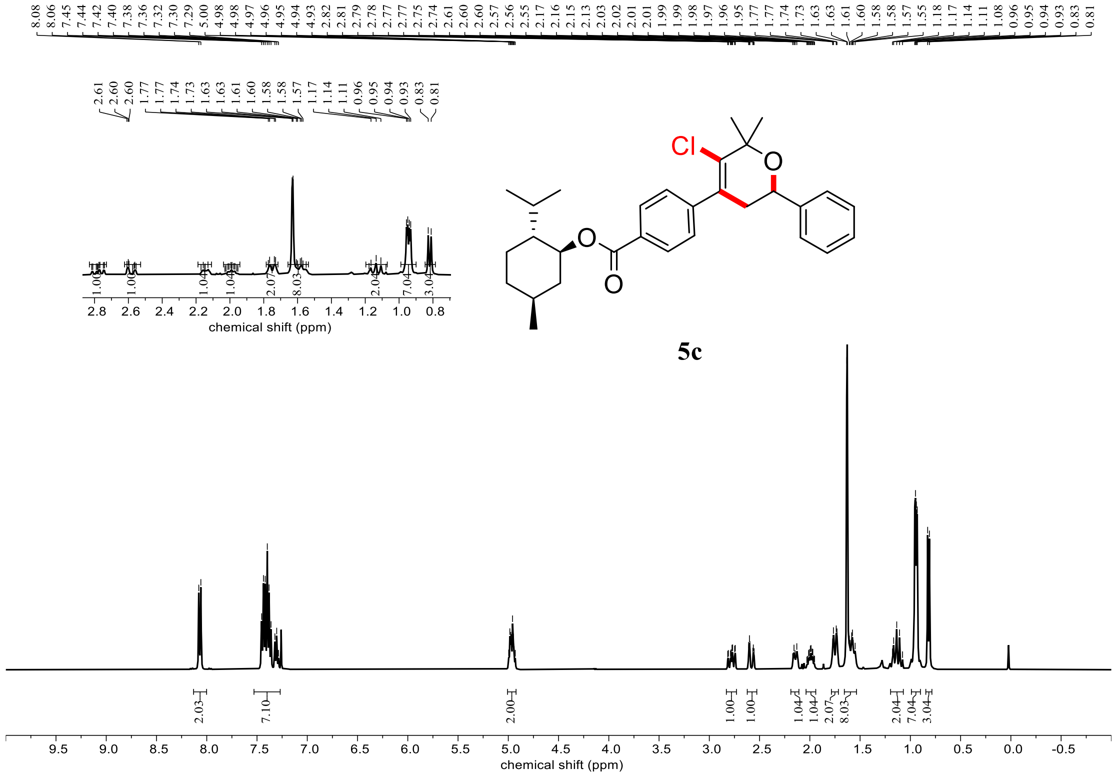

^{13}C NMR of 5c

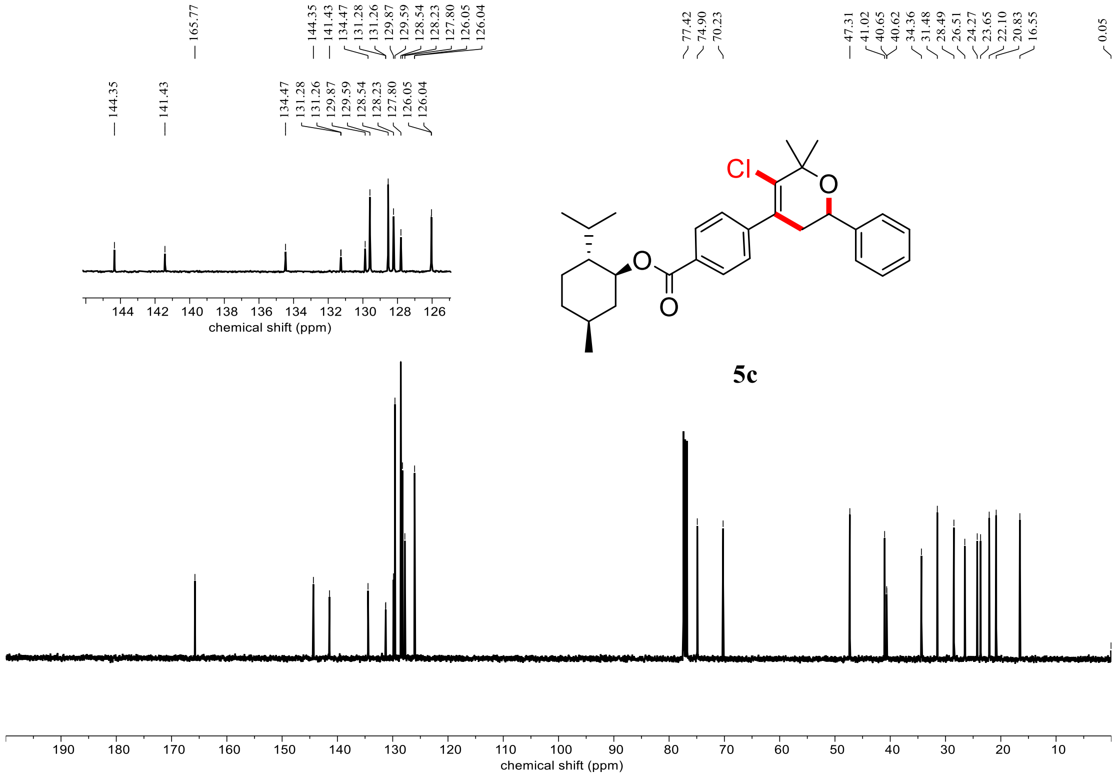

^{1}H NMR of 5d

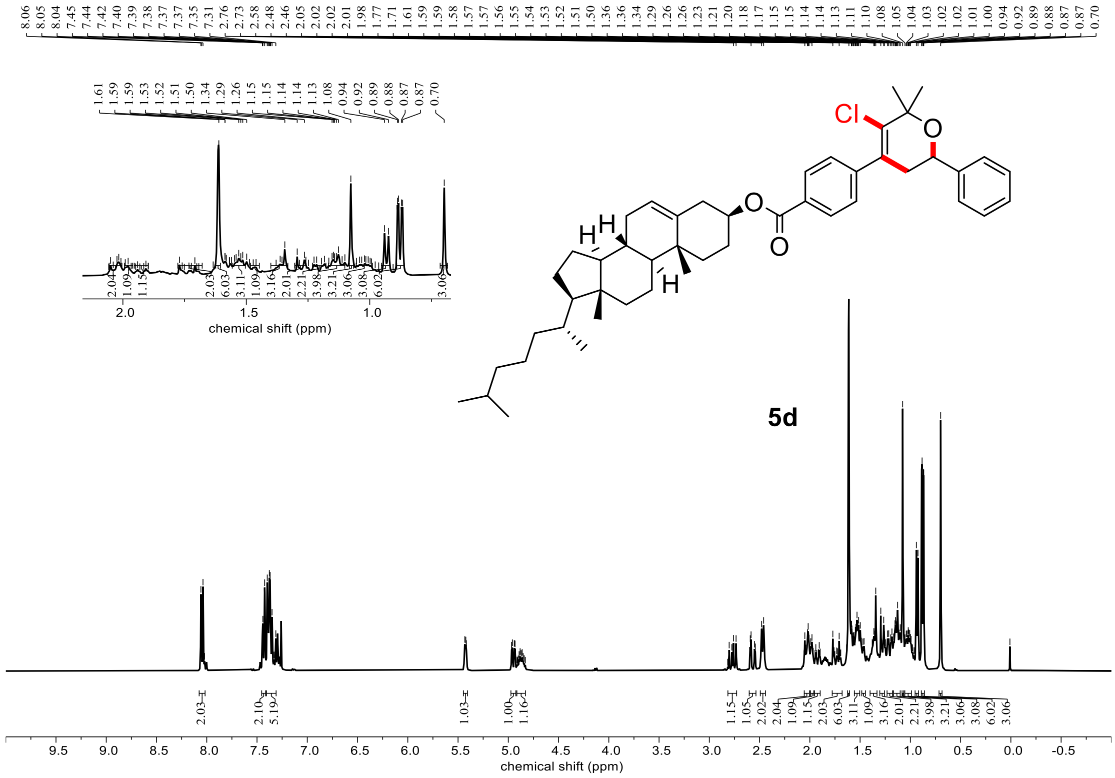

^{13}C NMR of 5d

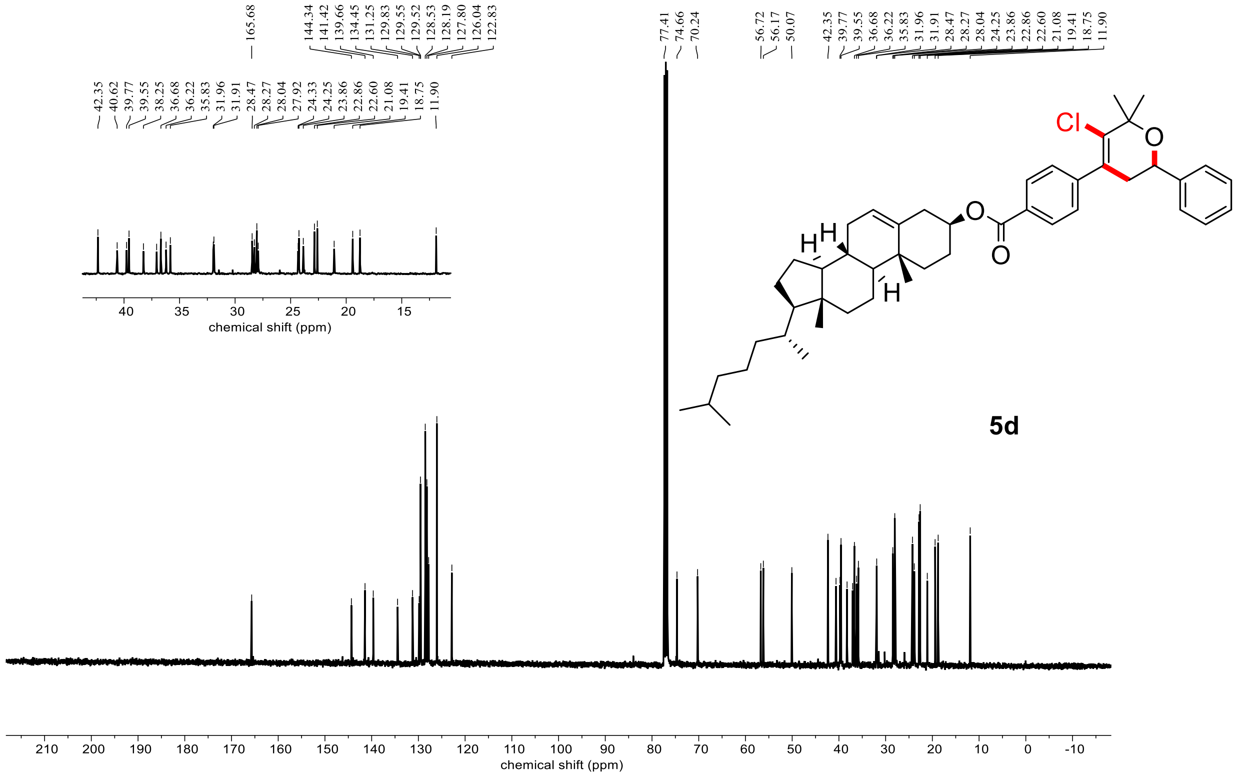

^{1}H NMR of 5e

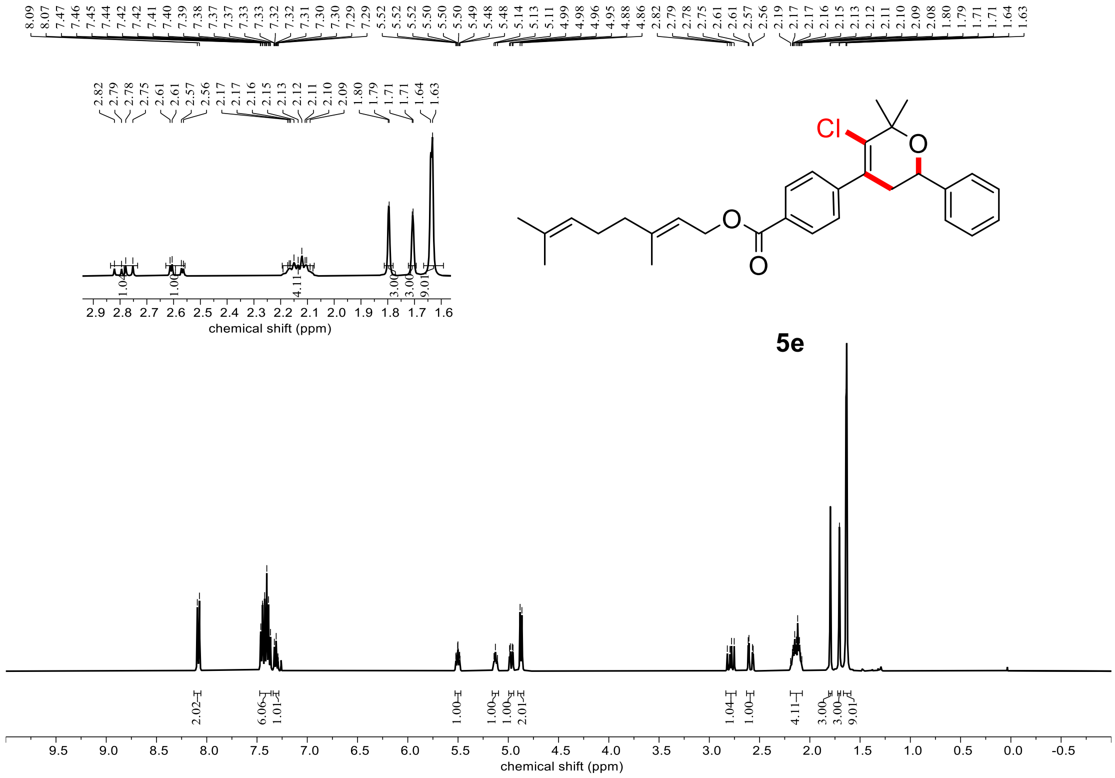

^{13}C NMR of 5e

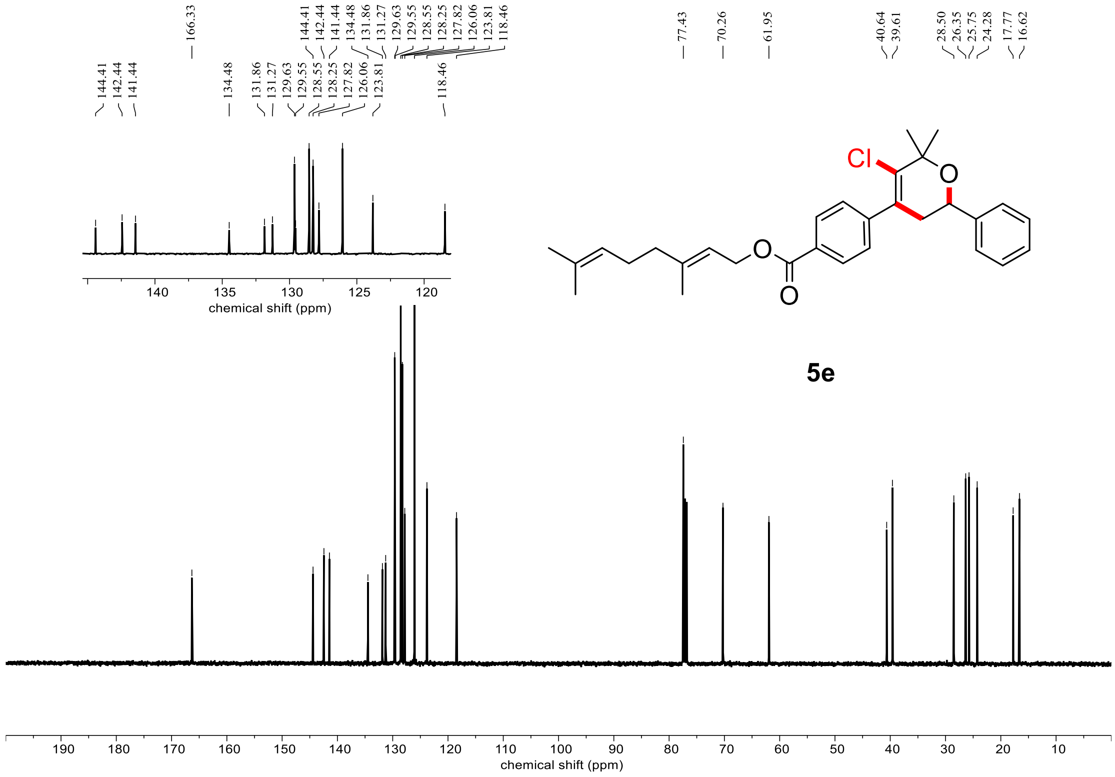

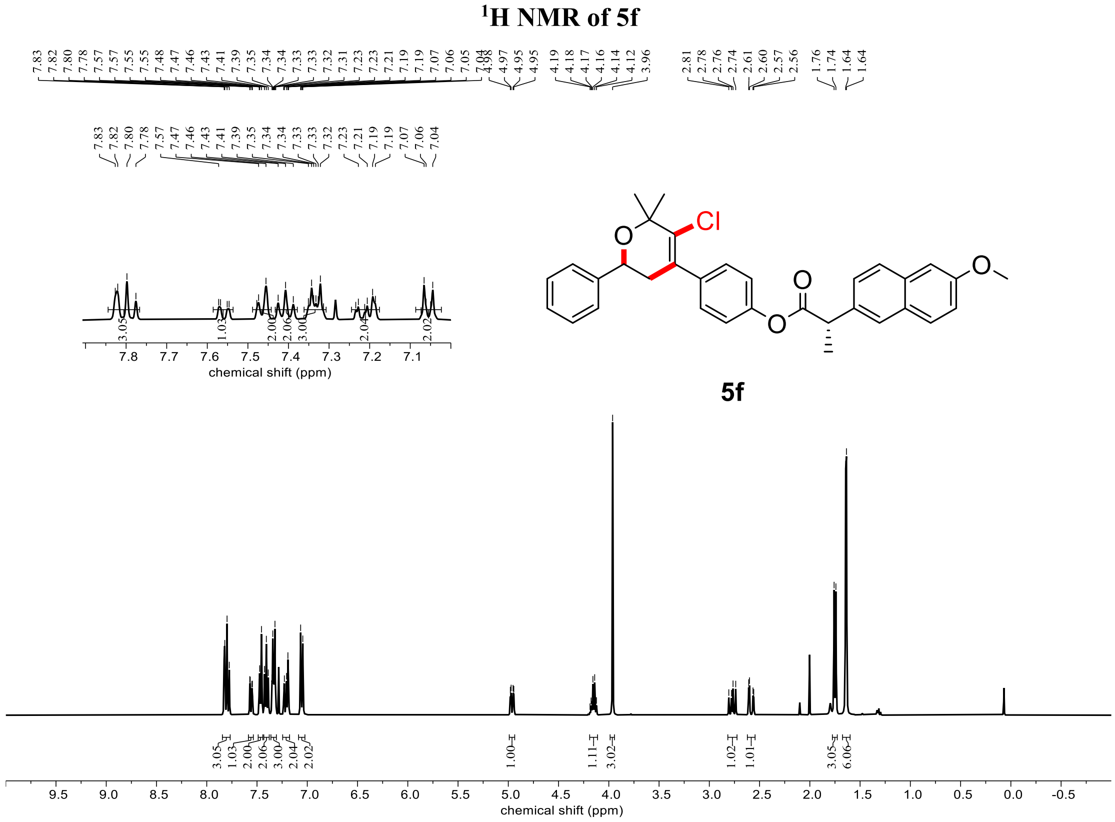
1H NMR of 5f
5f
chemical shift (ppm)

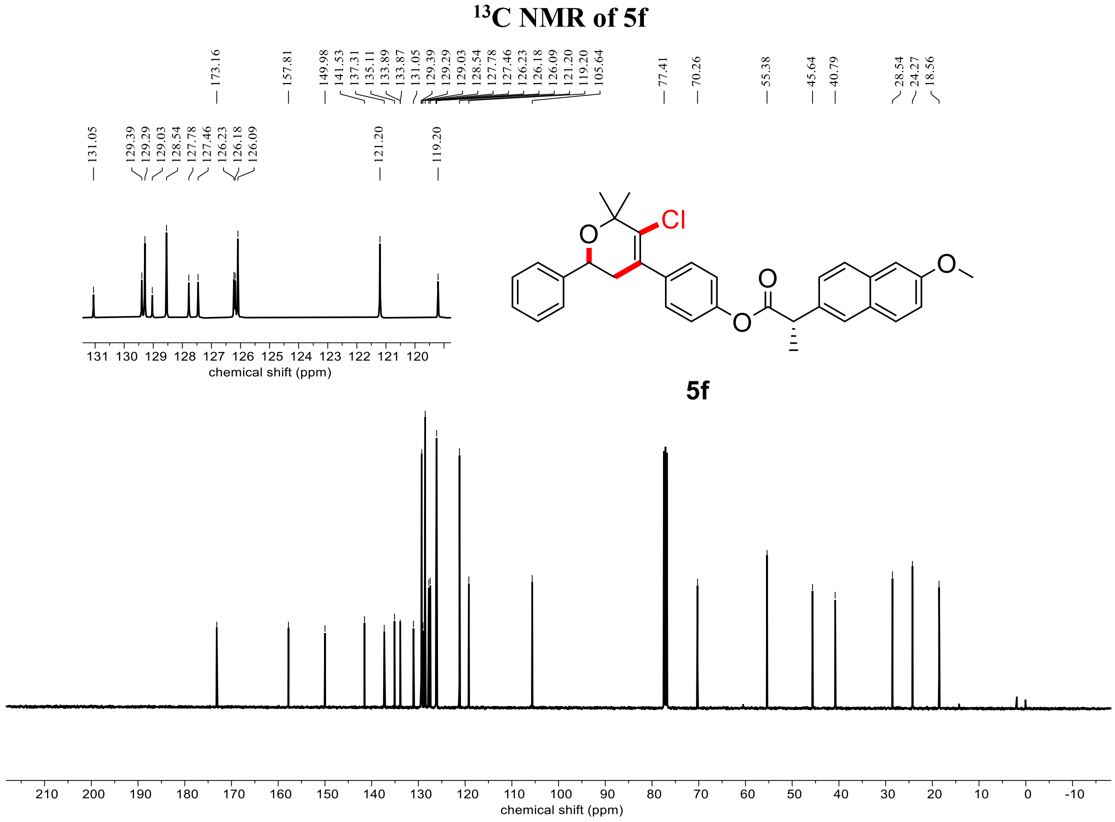
13C NMR of 5f
5f
chemical shift (ppm)

IR spectra of all compounds

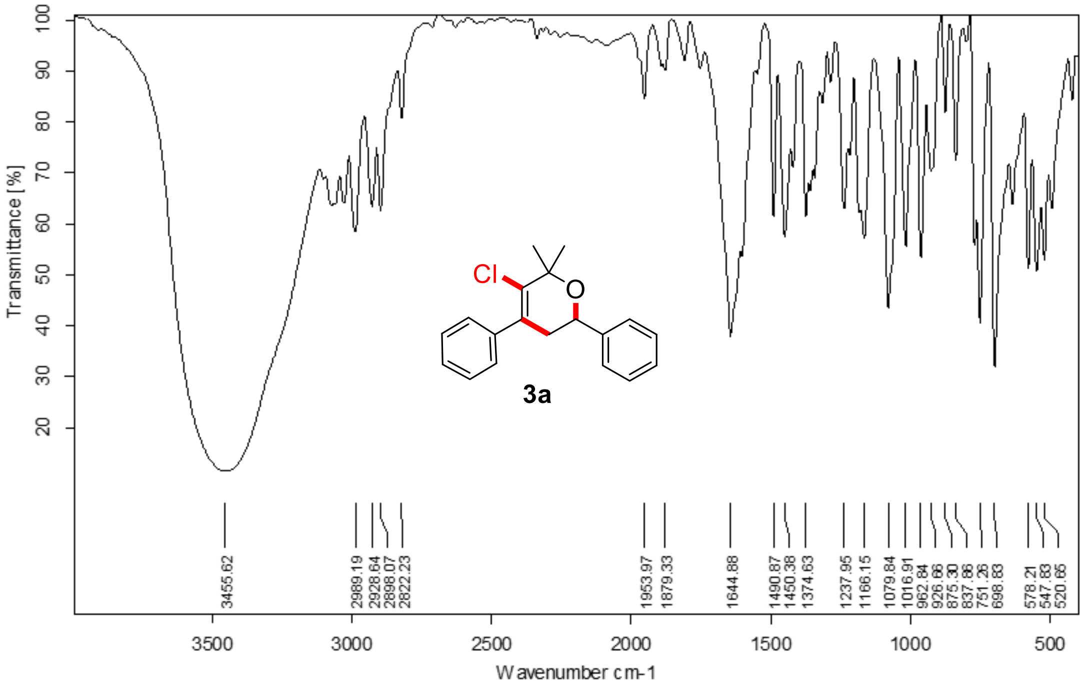

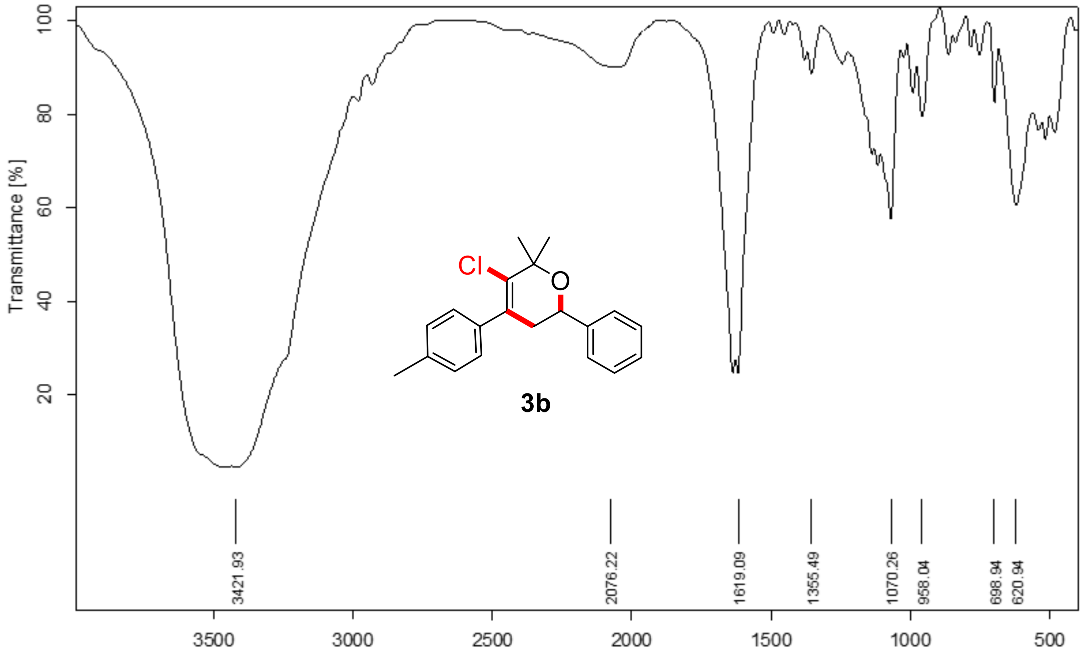

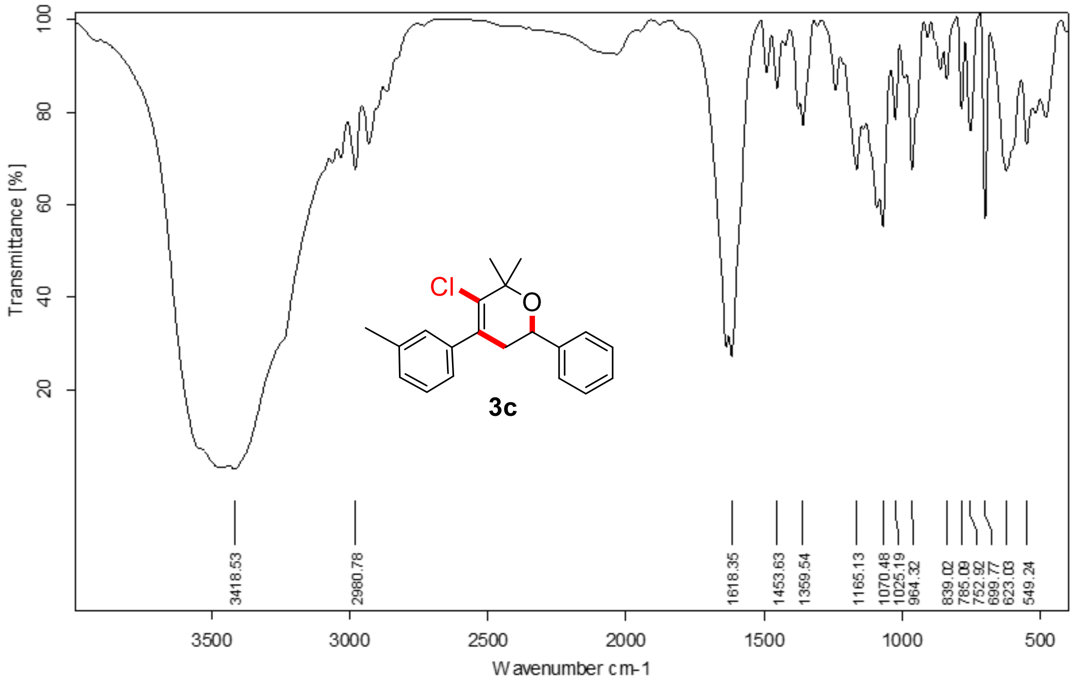

100
80
60
40
20
Transmittance [%]
3c
3418.53
2980.78
1618.35
1453.63
1359.54
1165.13
1070.48
1025.19
964.32
839.02
785.09
752.92
699.77
623.03
549.24
3500
3000
2500
2000
1500
1000
500
Wavenumber cm-1

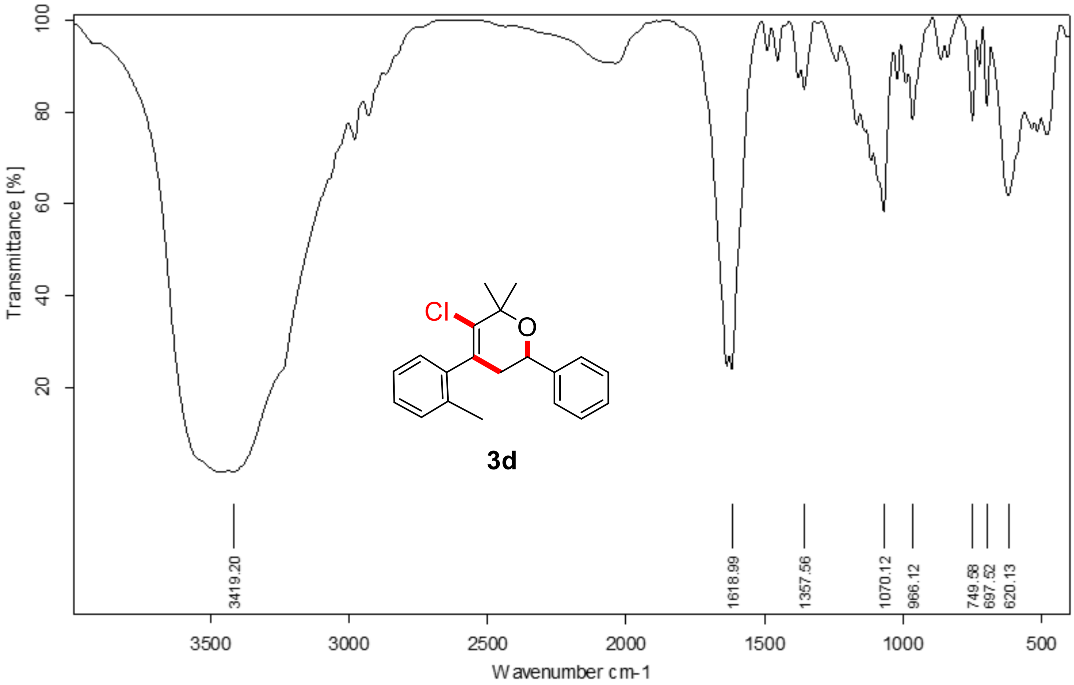

100
80
60
40
20
Transmittance [%]
3d
3419.20
1618.99
1357.56
1070.12
966.12
749.58
697.52
620.13
3500
3000
2500
2000
1500
1000
500
Wavenumber cm-1

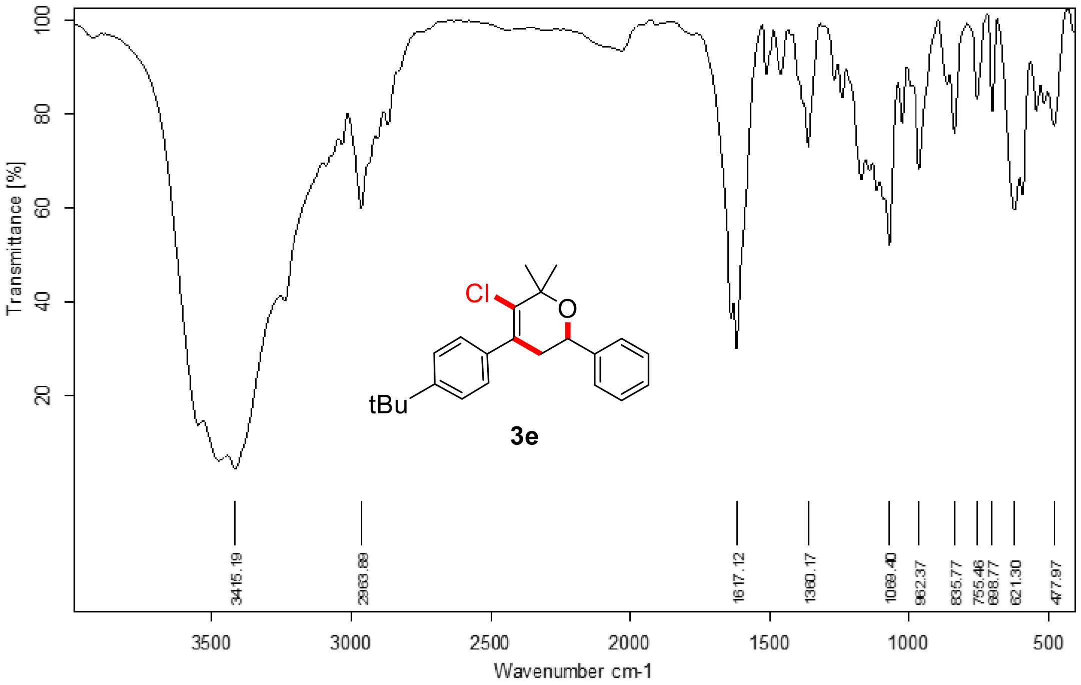
100
80
60
40
20
Transmittance [%]
Cl
O
tBu
3e
3415.19
2963.89
1617.12
1360.17
1069.40
962.37
835.77
755.46
698.77
621.30
477.97
3500
3000
2500
2000
1500
1000
500
Wavenumber cm-1

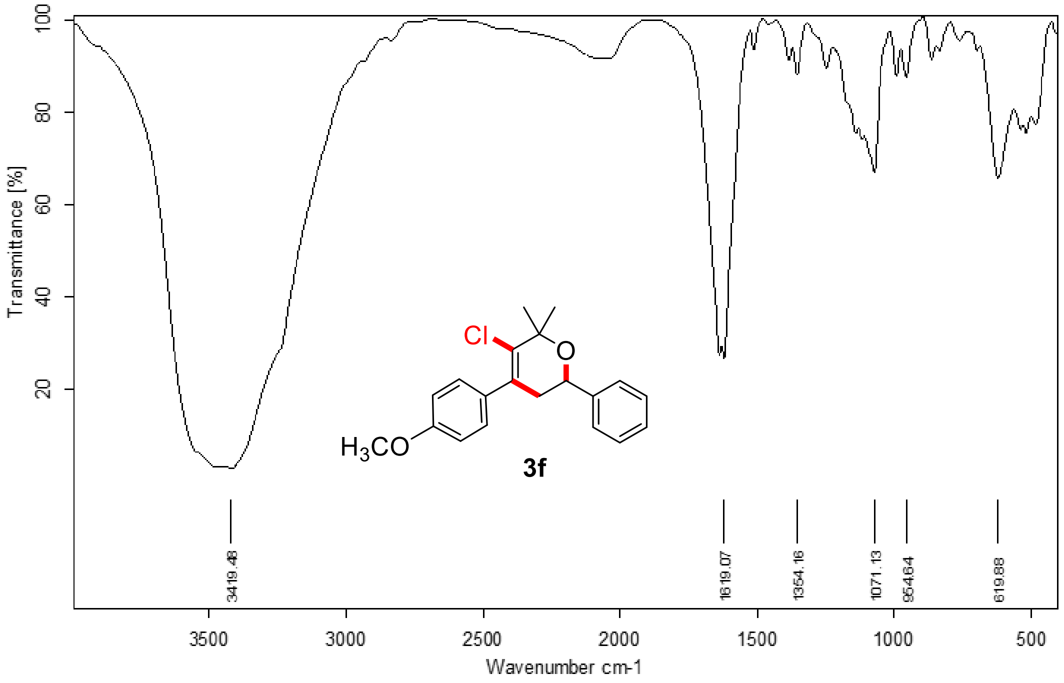
100
80
60
40
20
Transmittance [%]
Cl
O
H3CO
3f
3419.48
1619.07
1354.16
1071.13
954.64
619.88
3500
3000
2500
2000
1500
1000
500
Wavenumber cm-1

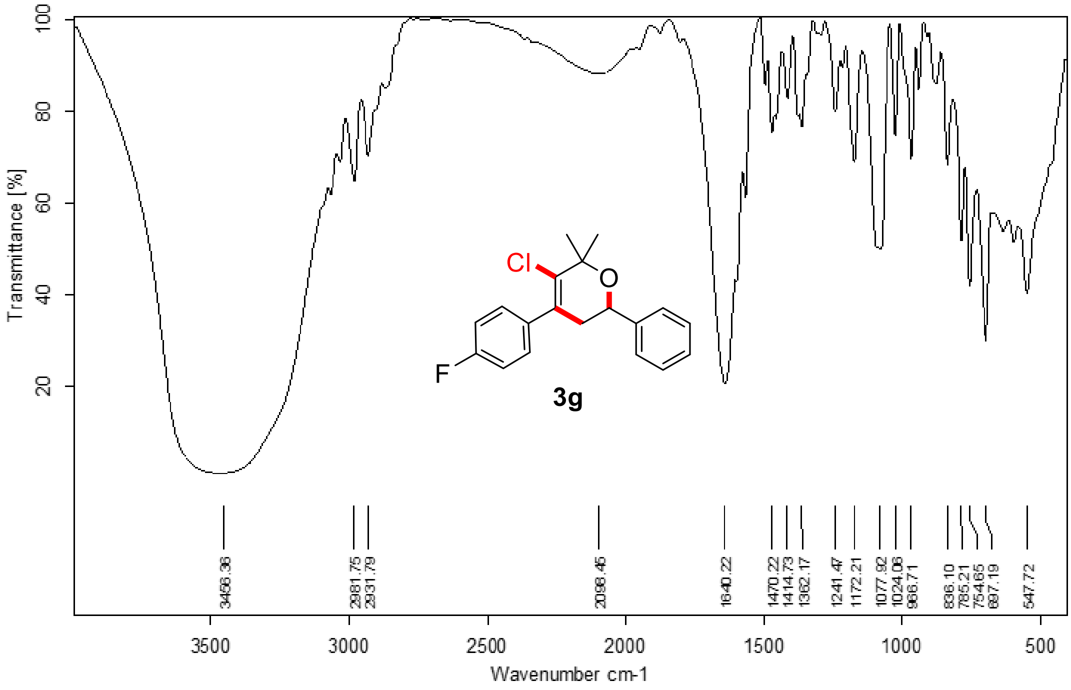

Transmittance [%]
Wavenumber cm-1
3g
3456.36
2981.75
2931.79
2098.45
1640.22
1470.22
1414.73
1362.17
1241.47
1172.21
1077.92
1024.06
986.71
836.10
785.21
754.65
697.19
547.72

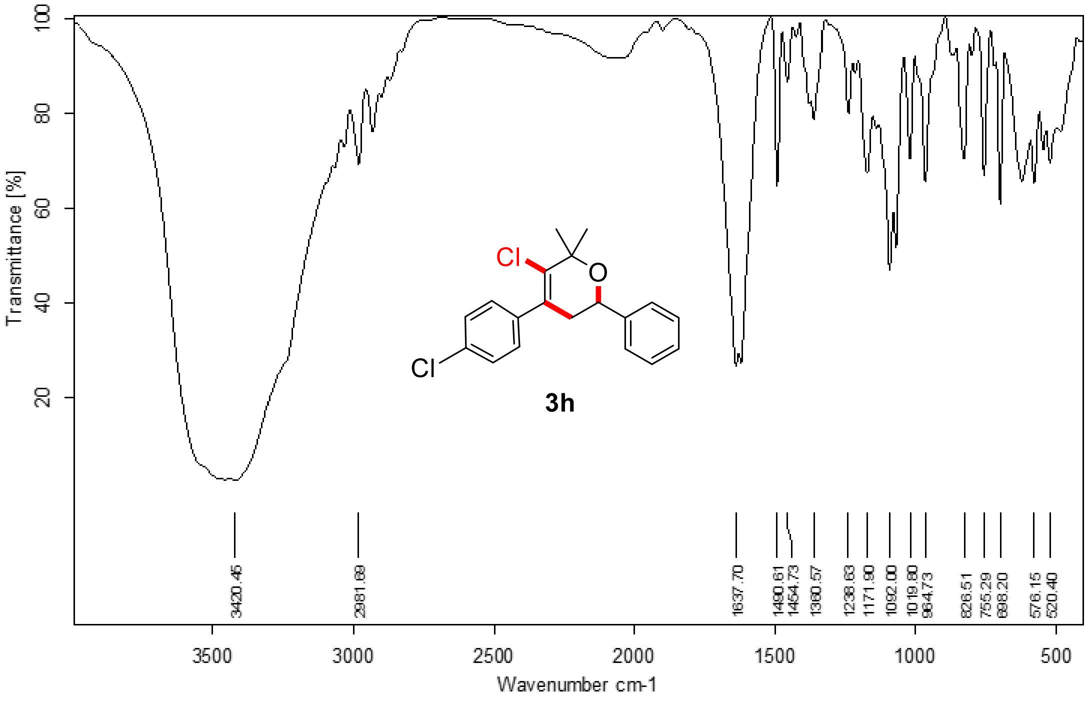

Transmittance [%]
Wavenumber cm-1
3h
3420.45
2981.89
1637.70
1490.61
1454.73
1360.57
1238.63
1171.90
1092.00
1019.80
964.73
826.51
755.29
698.20
576.15
520.40

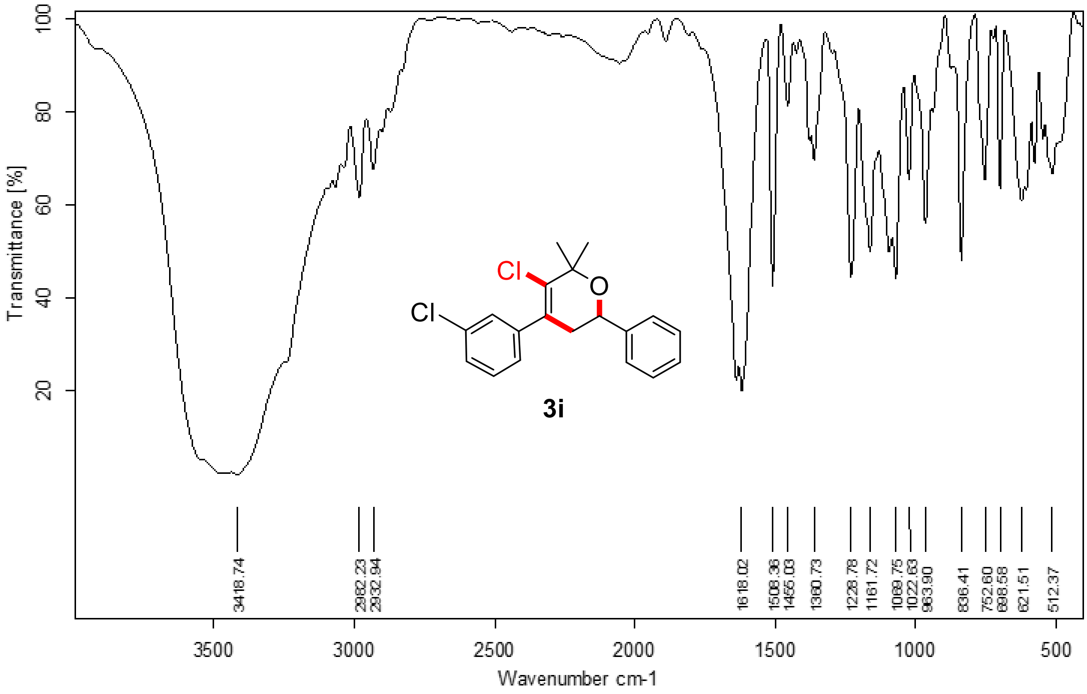
Transmittance [%]
100
80
60
40
20
Cl
O
Cl
3i
3418.74
2982.23
2932.94
1618.02
1508.36
1455.03
1360.73
1228.78
1161.72
1069.75
1022.63
963.90
836.41
752.60
698.58
621.51
512.37
3500
3000
2500
2000
1500
1000
500
Wavenumber cm-1

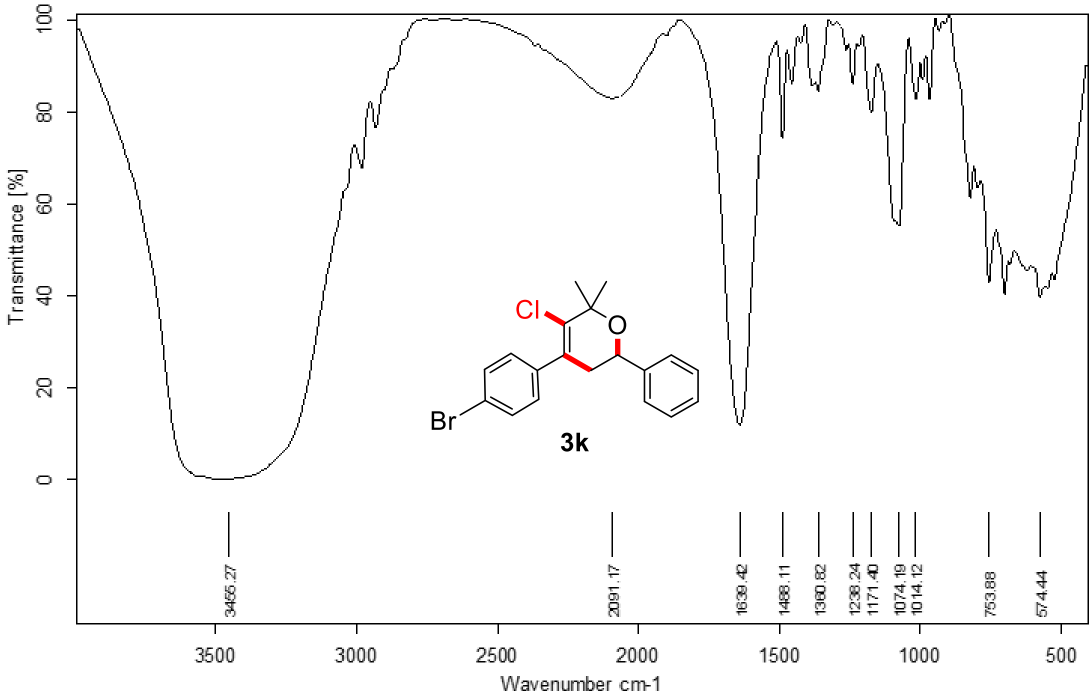
Transmittance [%]
100
80
60
40
20
0
Cl
O
Br
3k
3455.27
2091.17
1639.42
1488.11
1360.82
1238.24
1171.40
1074.19
1014.12
753.88
574.44
3500
3000
2500
2000
1500
1000
500
Wavenumber cm-1

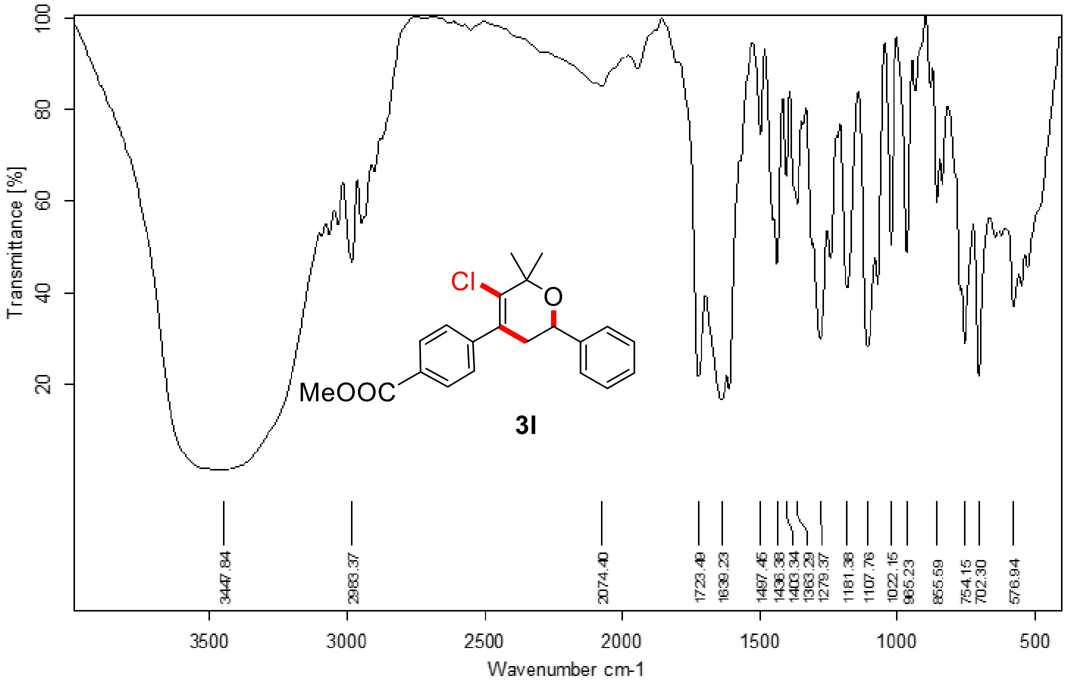
Transmittance [%]
Wavenumber cm-1
MeOOC
Cl
O
3447.84
2983.37
2074.40
1723.49
1639.23
1497.45
1436.38
1403.34
1363.29
1279.37
1181.38
1107.76
1022.15
965.23
855.59
754.15
702.30
576.94

3l

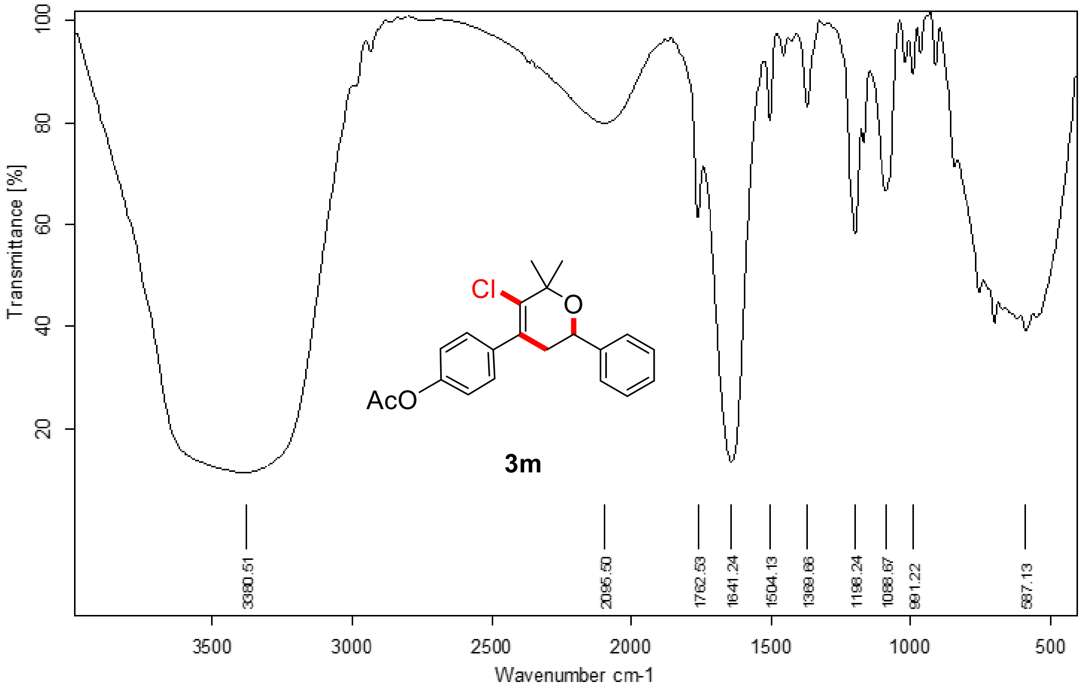
Transmittance [%]
Wavenumber cm-1
AcO
Cl
O
3380.51
2095.50
1762.53
1641.24
1504.13
1369.68
1198.24
1088.67
991.22
587.13

3m

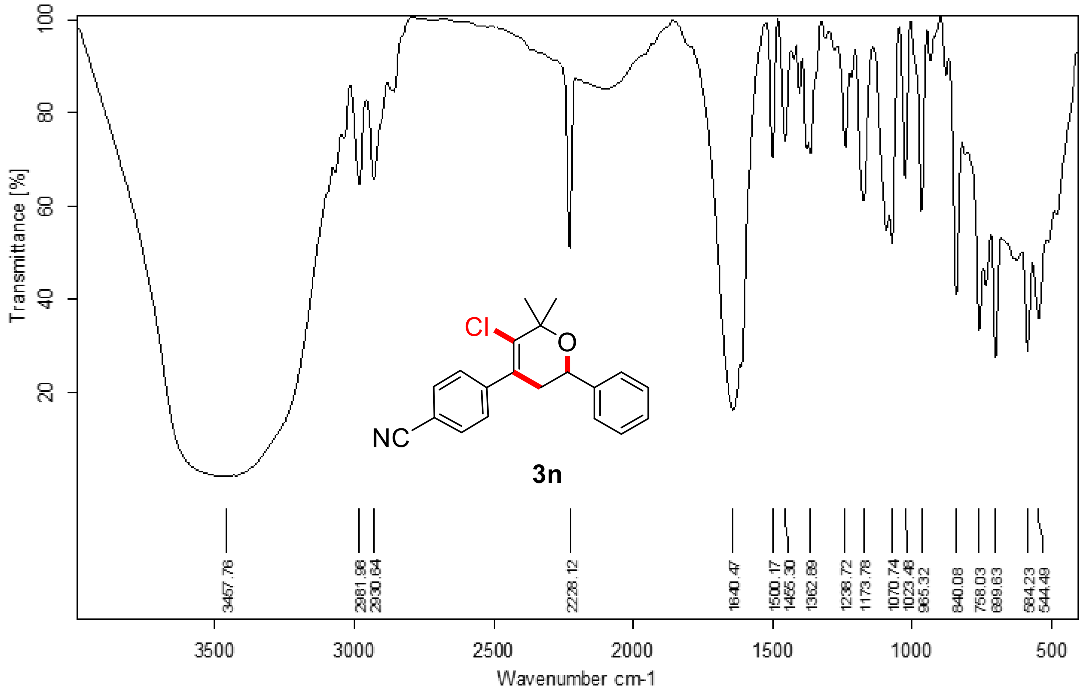
Transmittance [%]
Wavenumber cm-1
Cl
O
NC
3n
3457.76
2981.98
2930.64
2228.12
1640.47
1500.17
1455.30
1362.89
1238.72
1173.78
1070.74
1023.48
965.32
840.08
758.03
699.63
584.23
544.49

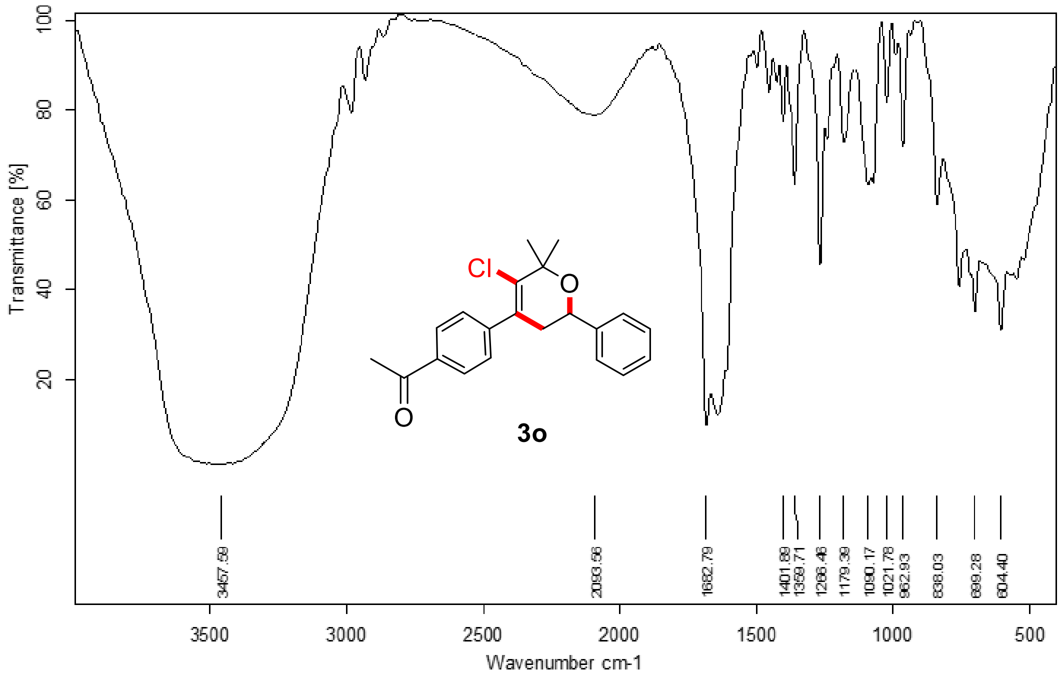
Transmittance [%]
Wavenumber cm-1
Cl
O
O
3o
3457.59
2093.56
1682.79
1401.89
1359.71
1266.48
1179.39
1090.17
1021.78
962.93
838.03
699.28
604.40

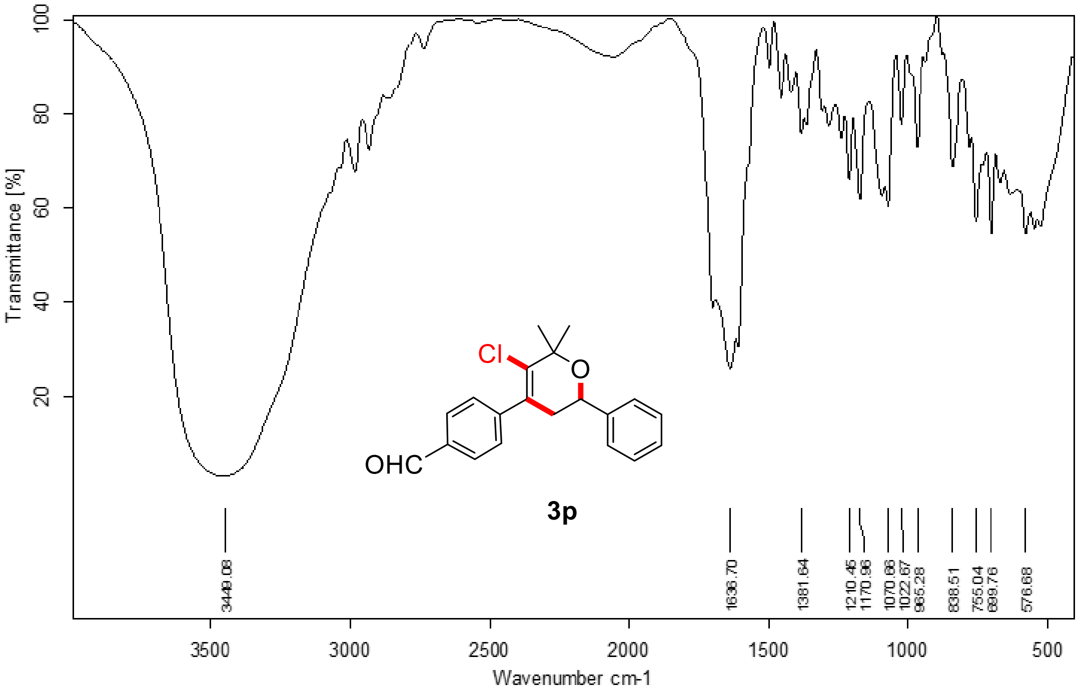
Transmittance [%]
100
80
60
40
20
Cl
O
OHC
3p
3449.08
1636.70
1381.64
1210.45
1170.96
1070.86
1022.67
965.28
838.51
755.04
699.76
576.68
3500
3000
2500
2000
1500
1000
500
Wavenumber cm-1

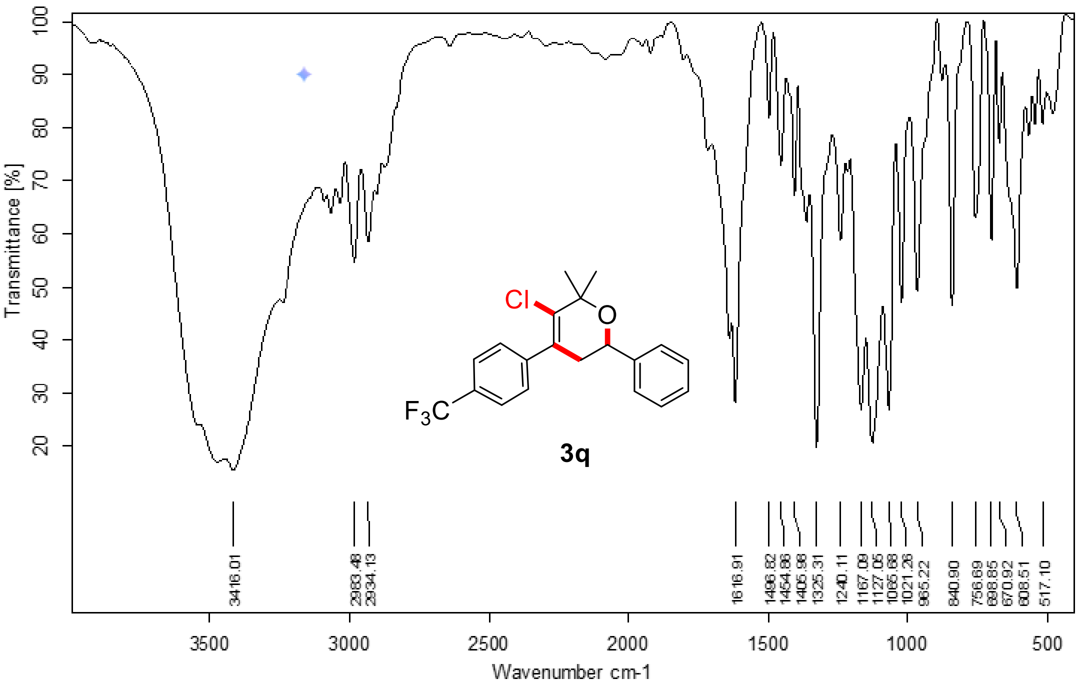
Transmittance [%]
100
90
80
70
60
50
40
30
20
Cl
O
F3C
3q
3416.01
2983.48
2934.13
1616.91
1496.82
1454.86
1405.98
1325.31
1240.11
1167.09
1127.05
1065.68
1021.26
965.22
840.90
756.69
698.85
670.92
608.51
517.10
3500
3000
2500
2000
1500
1000
500
Wavenumber cm-1

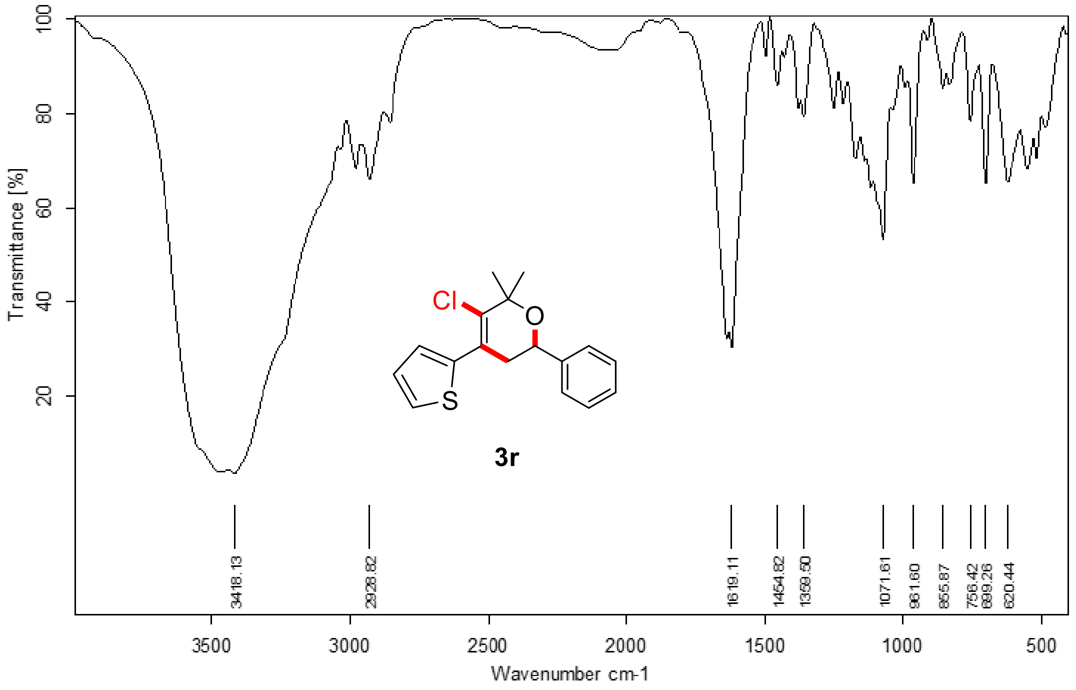
Transmittance [%]
100
80
60
40
20
3500
3000
2500
2000
1500
1000
500
Wavenumber cm-1
3418.13
2928.82
1619.11
1454.82
1359.50
1071.61
961.60
855.87
756.42
699.26
620.44
Cl
O
S
3r

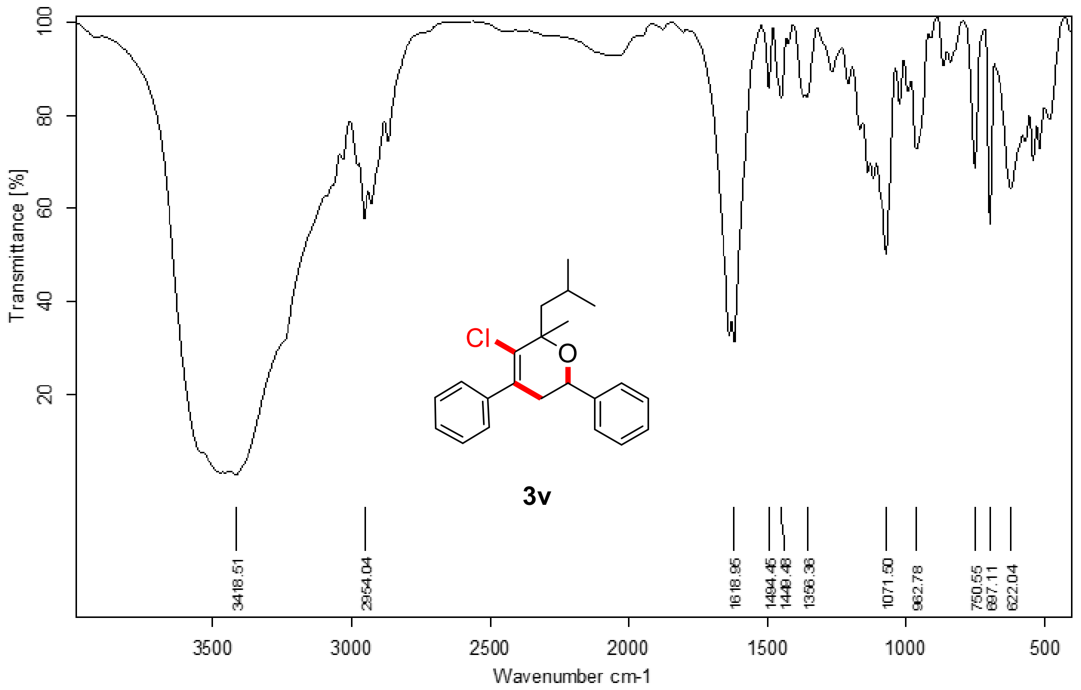
Transmittance [%]
100
80
60
40
20
3500
3000
2500
2000
1500
1000
500
Wavenumber cm-1
3418.51
2954.04
1618.95
1494.45
1449.48
1356.36
1071.50
962.78
750.55
697.11
622.04
Cl
O
3v

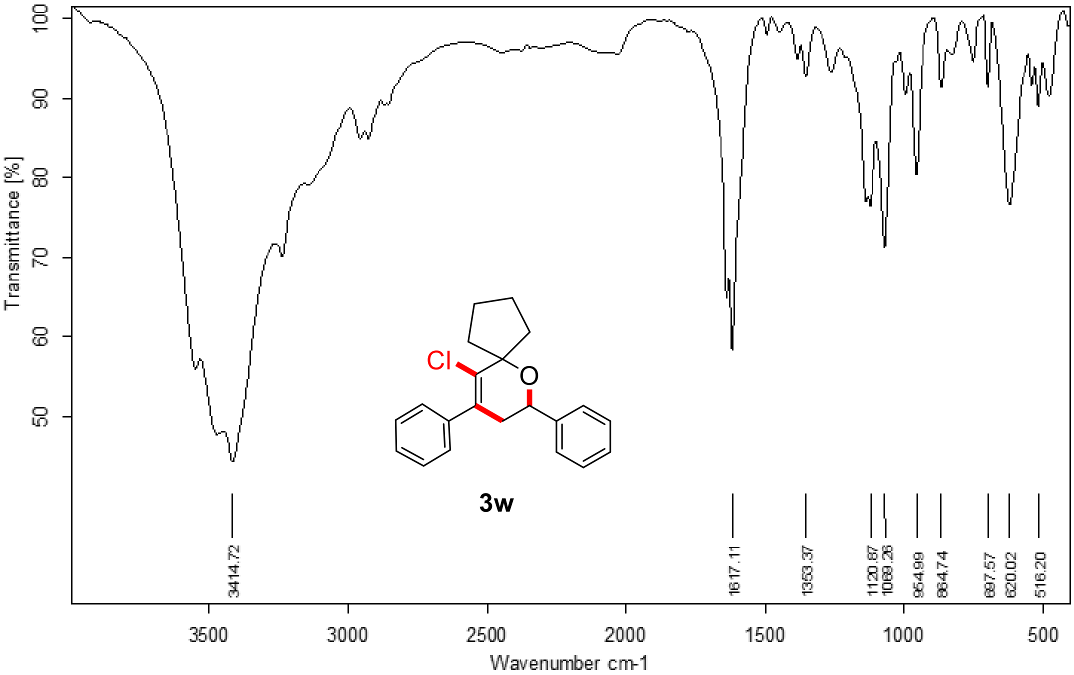
Transmittance [%]
100
90
80
70
60
50
Cl
O
3w
3414.72
1617.11
1353.37
1120.87
1069.26
954.99
864.74
697.57
620.02
516.20
3500
3000
2500
2000
1500
1000
500
Wavenumber cm-1

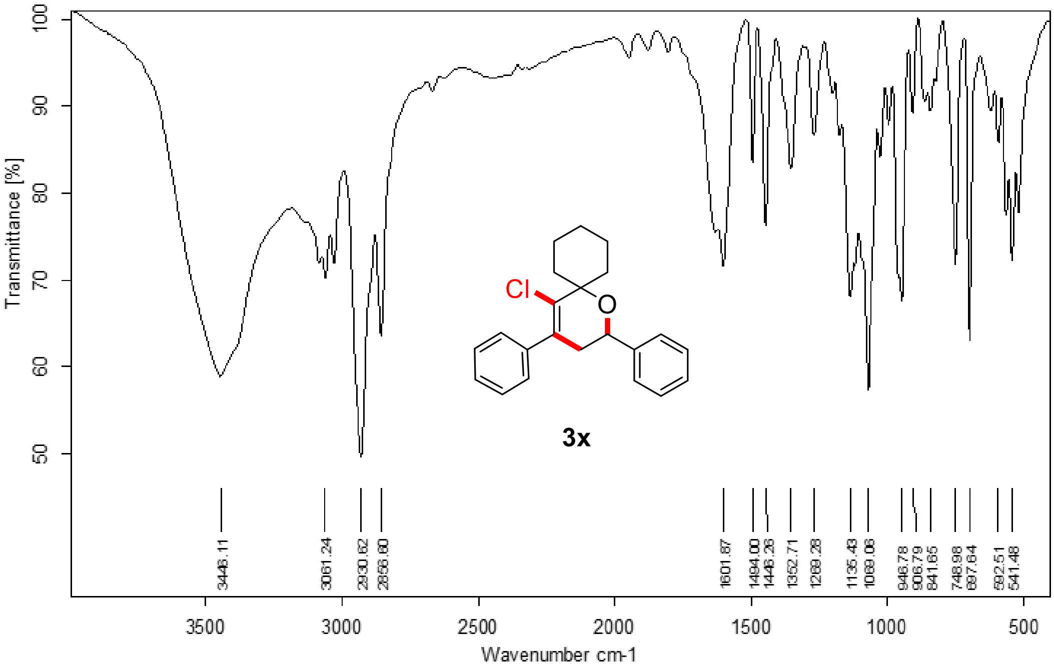
Transmittance [%]
100
90
80
70
60
50
Cl
O
3x
3446.11
3061.24
2930.62
2856.80
1601.87
1494.00
1446.28
1352.71
1269.28
1135.43
1069.06
946.78
906.79
841.65
748.98
697.64
592.51
541.48
3500
3000
2500
2000
1500
1000
500
Wavenumber cm-1

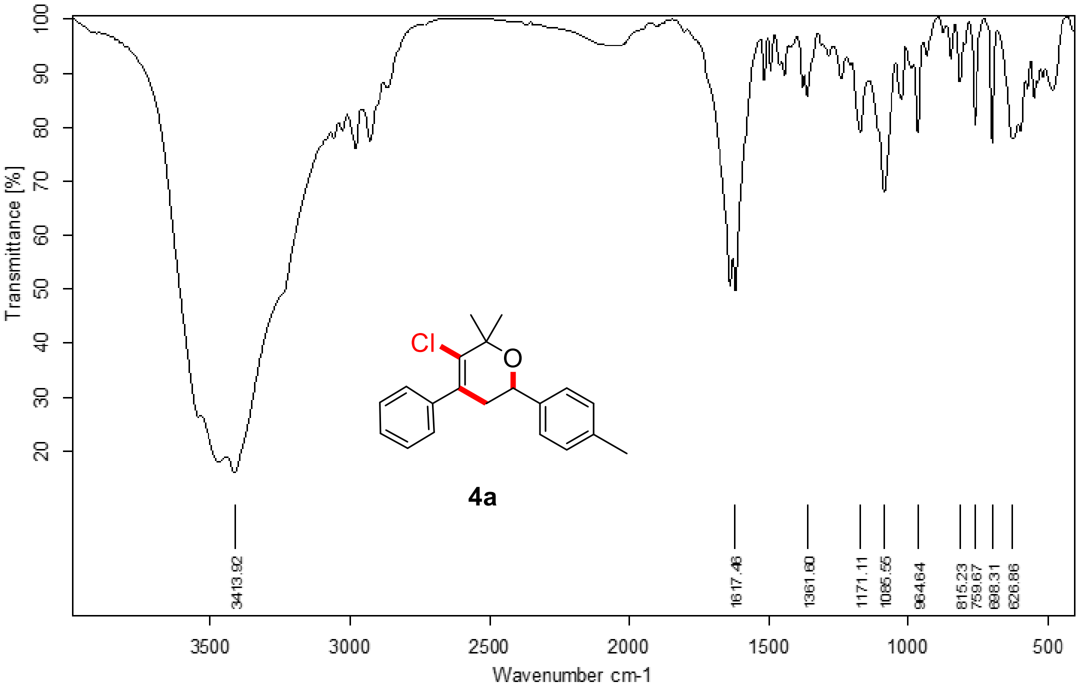
Transmittance [%]
Wavenumber cm-1
Cl
O
4a
3413.92
1617.48
1361.60
1171.11
1085.55
964.64
815.23
759.67
698.31
626.86

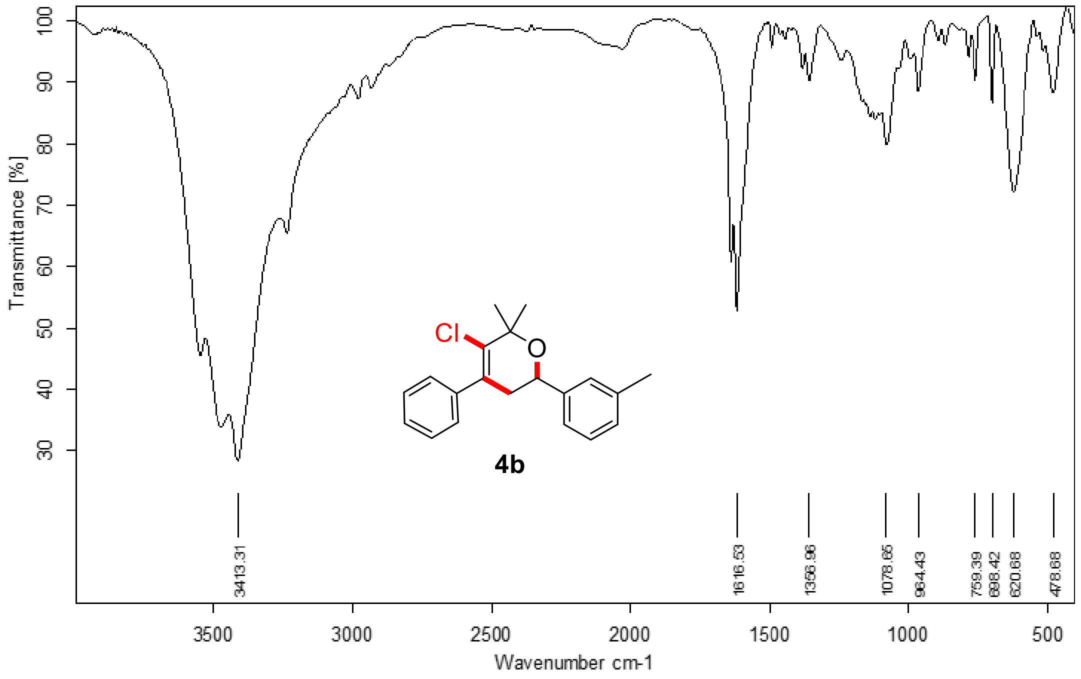
Transmittance [%]
Wavenumber cm-1
Cl
O
4b
3413.31
1616.53
1356.96
1078.65
964.43
759.39
698.42
620.68
478.68

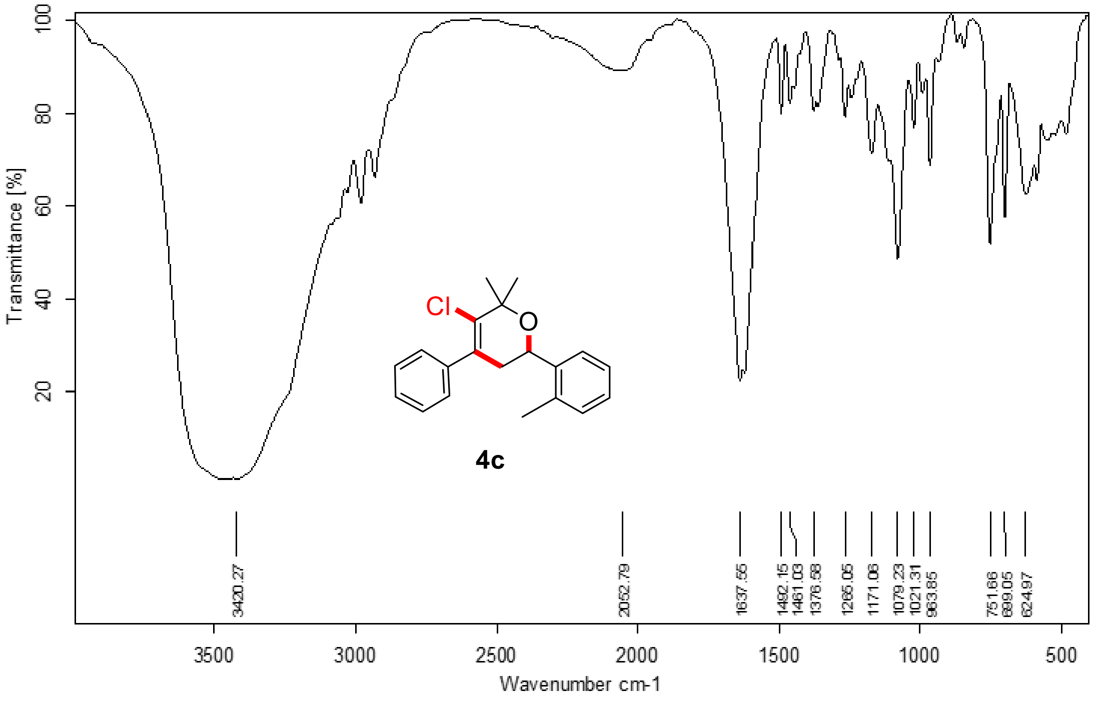
Transmittance [%]
Wavenumber cm-1
Cl
O
4c
3420.27
2052.79
1637.55
1492.15
1461.03
1376.58
1265.05
1171.06
1079.23
1021.31
963.85
751.66
699.05
624.97

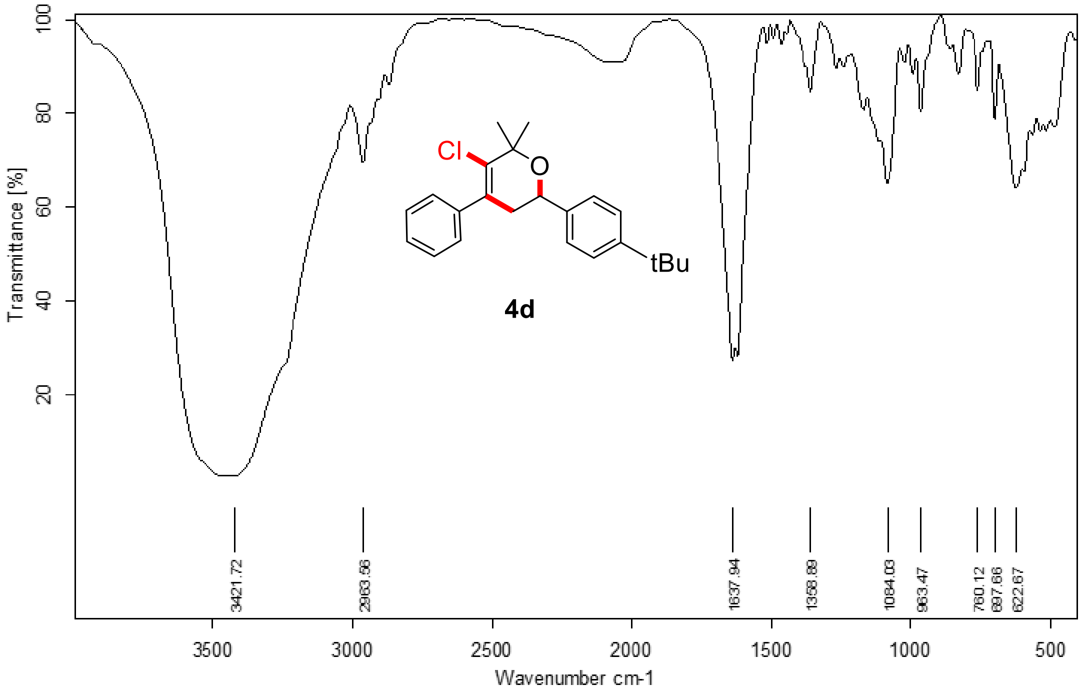
Transmittance [%]
Wavenumber cm-1
Cl
O
tBu
4d
3421.72
2963.56
1637.94
1358.89
1084.03
963.47
760.12
697.66
622.67

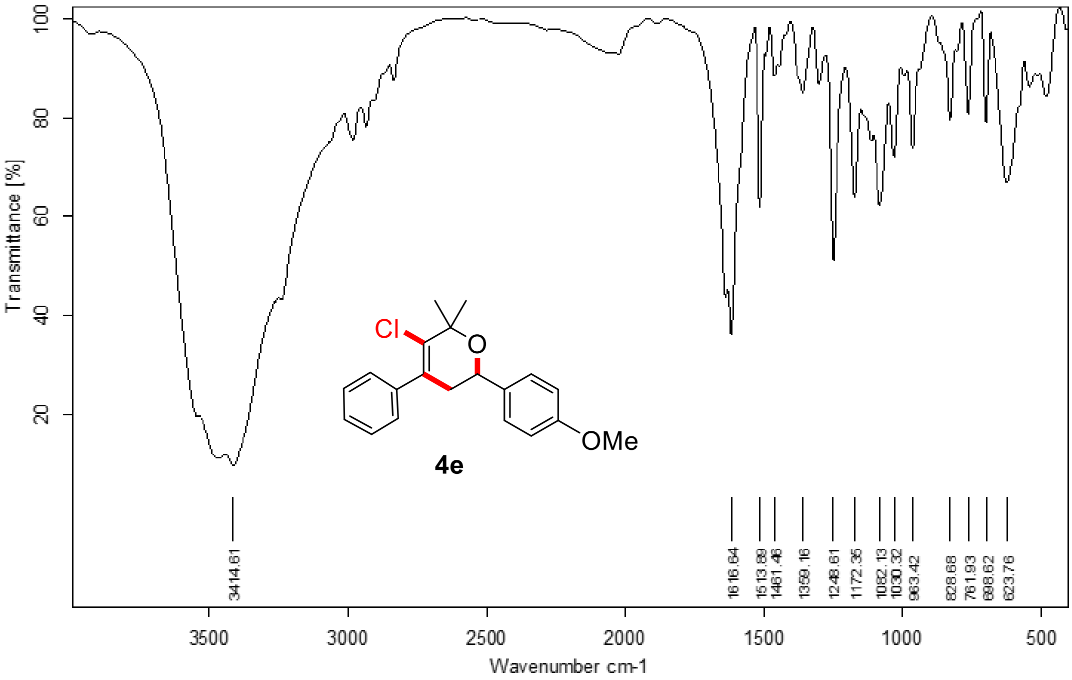
Transmittance [%]
100
80
60
40
20
Cl
O
OMe
4e
3414.61
1616.64
1513.89
1461.46
1359.16
1248.61
1172.35
1082.13
1030.32
963.42
828.68
761.93
698.62
623.76
3500
3000
2500
2000
1500
1000
500
Wavenumber cm-1

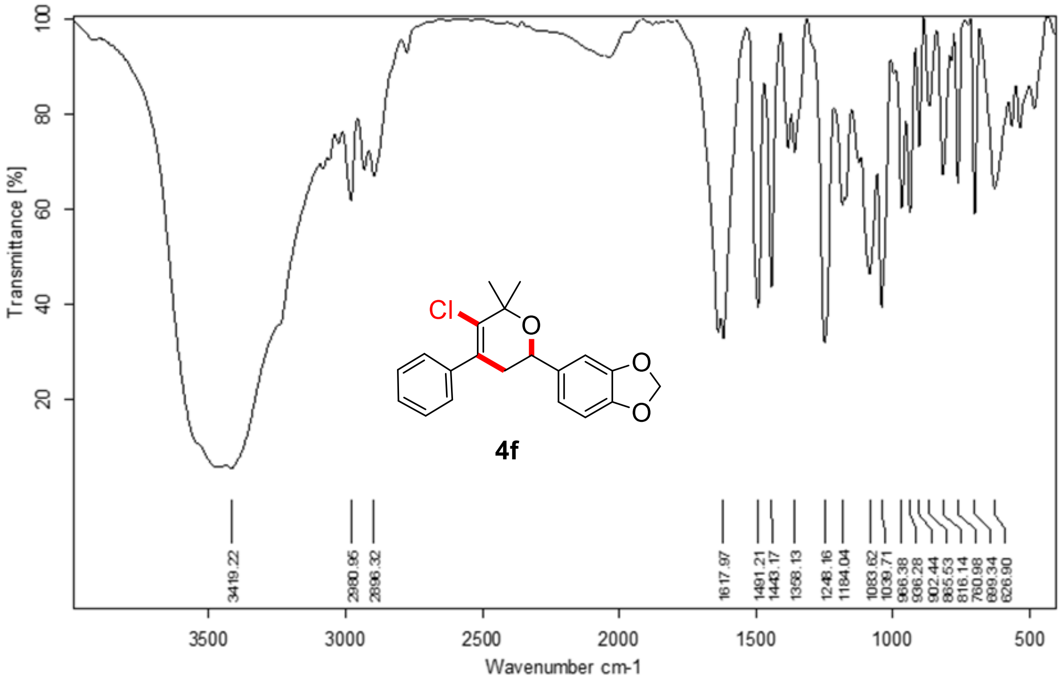
Transmittance [%]
100
80
60
40
20
Cl
O
O
O
4f
3419.22
2980.95
2896.32
1617.97
1491.21
1443.17
1358.13
1248.16
1184.04
1083.62
1039.71
966.38
936.28
902.44
865.53
816.14
760.98
699.34
626.90
3500
3000
2500
2000
1500
1000
500
Wavenumber cm-1

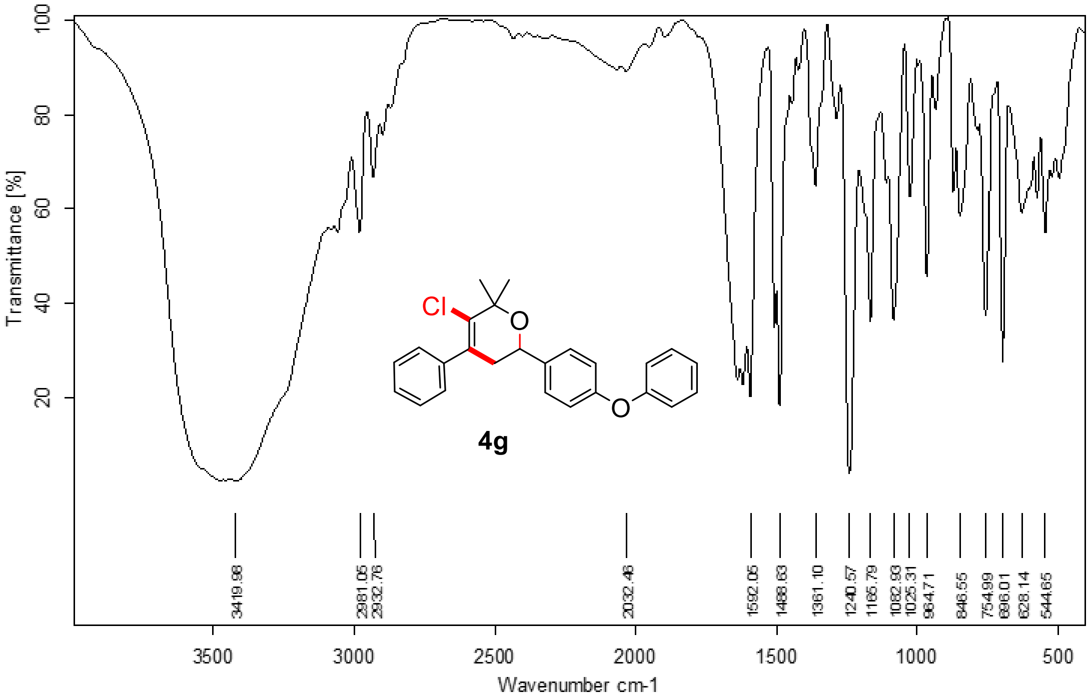
Transmittance [%]
100
80
60
40
20
Cl
O
O
4g
3419.98
2981.05
2932.76
2032.46
1592.05
1488.63
1361.10
1240.57
1165.79
1082.93
1025.31
964.71
846.55
754.99
696.01
628.14
544.65
3500
3000
2500
2000
1500
1000
500
Wavenumber cm-1

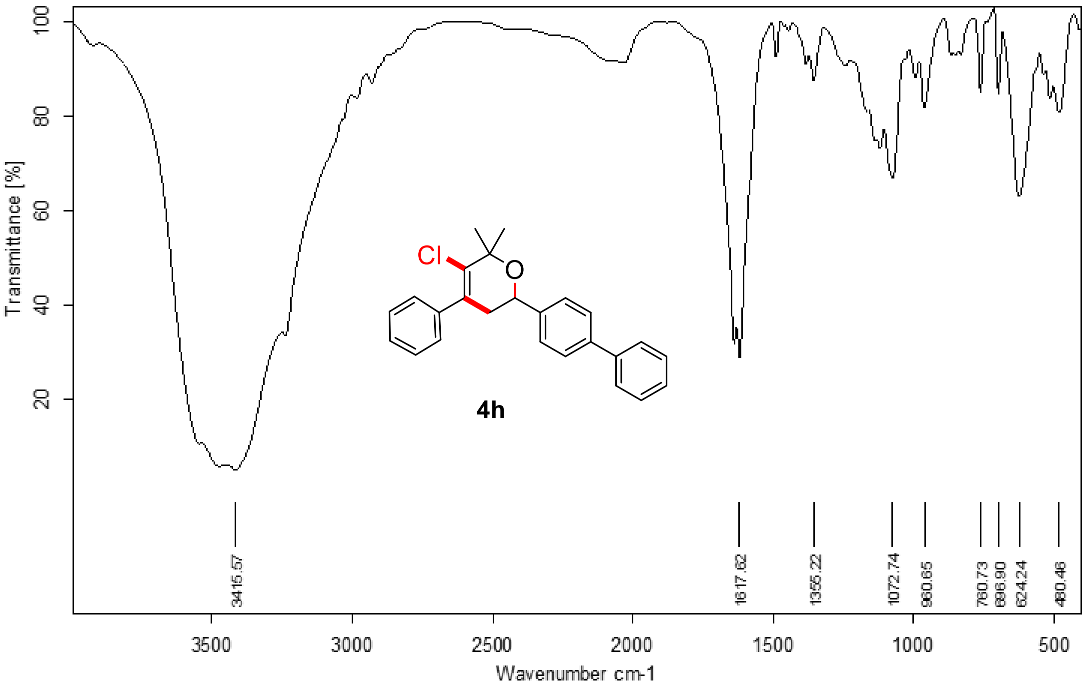
Transmittance [%]
100
80
60
40
20
Cl
O
4h
3415.57
1617.62
1355.22
1072.74
960.65
760.73
696.90
624.24
480.46
3500
3000
2500
2000
1500
1000
500
Wavenumber cm-1

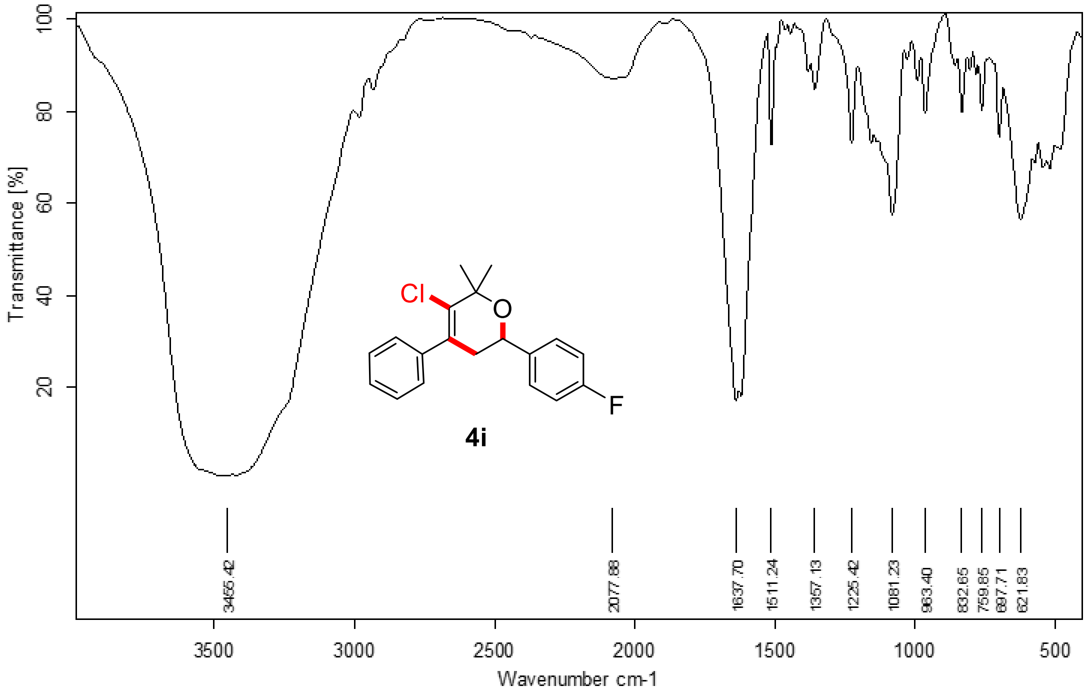
Transmittance [%]
100
80
60
40
20
Cl
O
F
4i
3455.42
2077.88
1637.70
1511.24
1357.13
1225.42
1081.23
963.40
832.65
759.85
697.71
621.83
3500
3000
2500
2000
1500
1000
500
Wavenumber cm-1

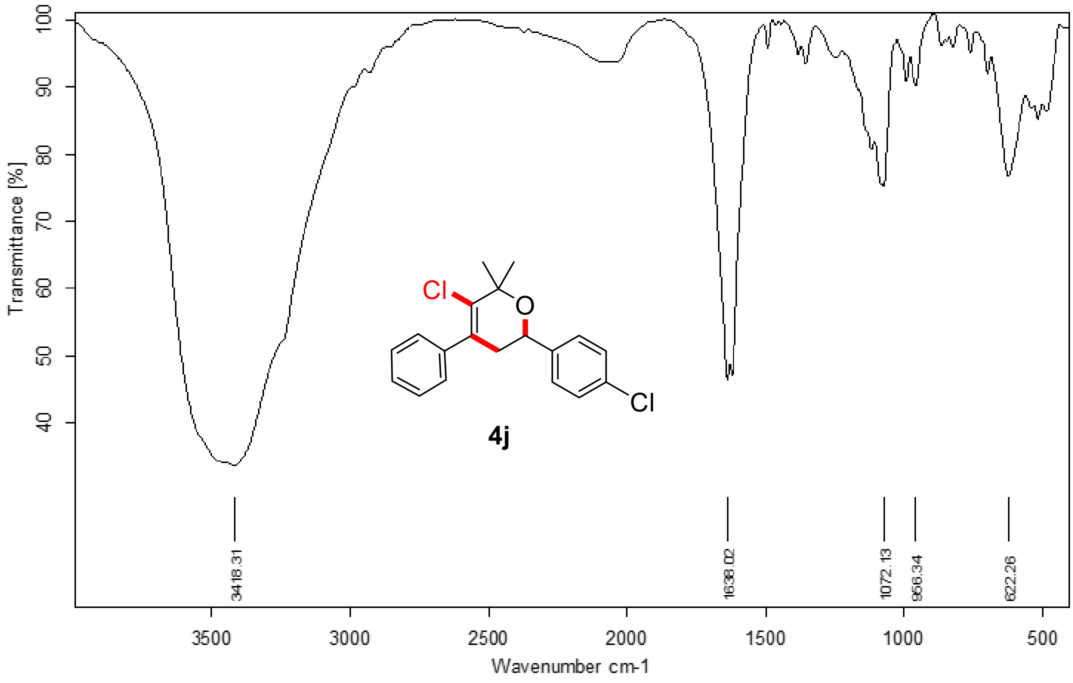
Transmittance [%]
100
90
80
70
60
50
40
Cl
O
Cl
4j
3418.31
1638.02
1072.13
956.34
622.26
3500
3000
2500
2000
1500
1000
500
Wavenumber cm-1

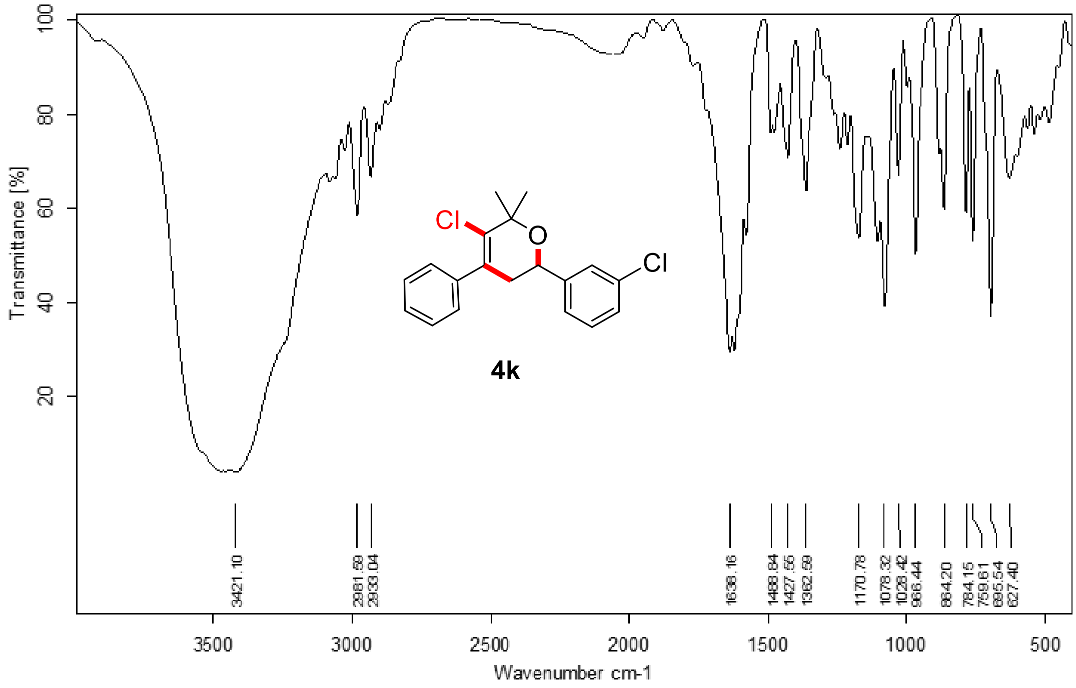
Transmittance [%]
Wavenumber cm-1
Cl
O
Cl
4k
3421.10
2981.59
2933.04
1638.16
1488.84
1427.56
1362.59
1170.78
1078.32
1028.42
986.44
884.20
784.15
759.61
695.54
627.40

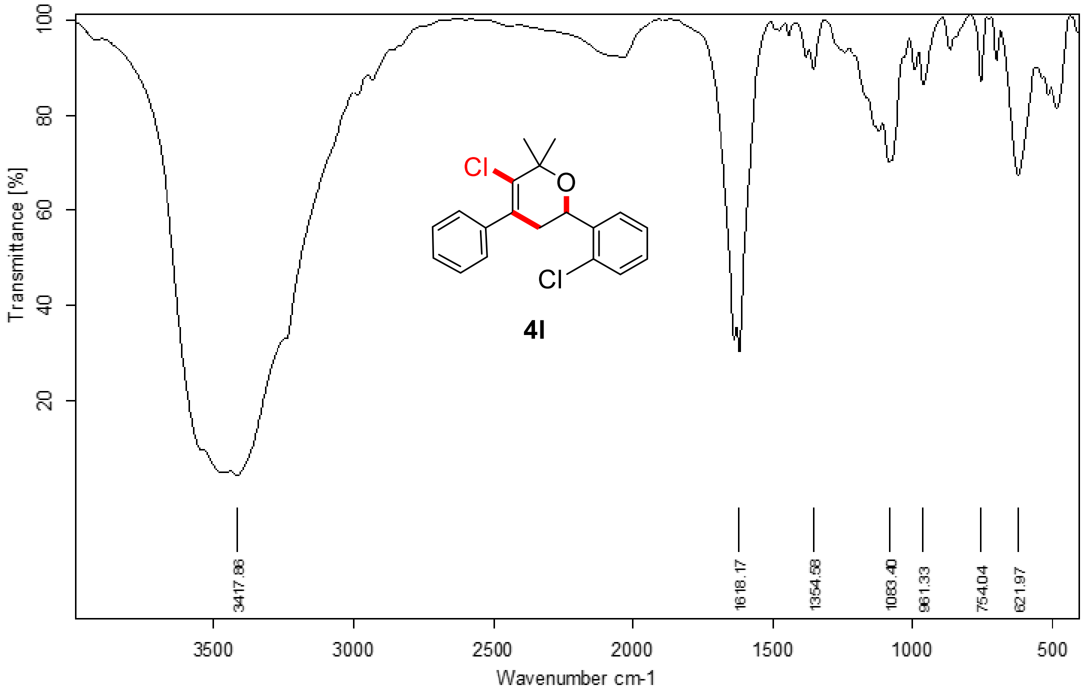
Transmittance [%]
Wavenumber cm-1
Cl
O
Cl
4l
3417.86
1618.17
1354.58
1083.40
961.33
754.04
621.97

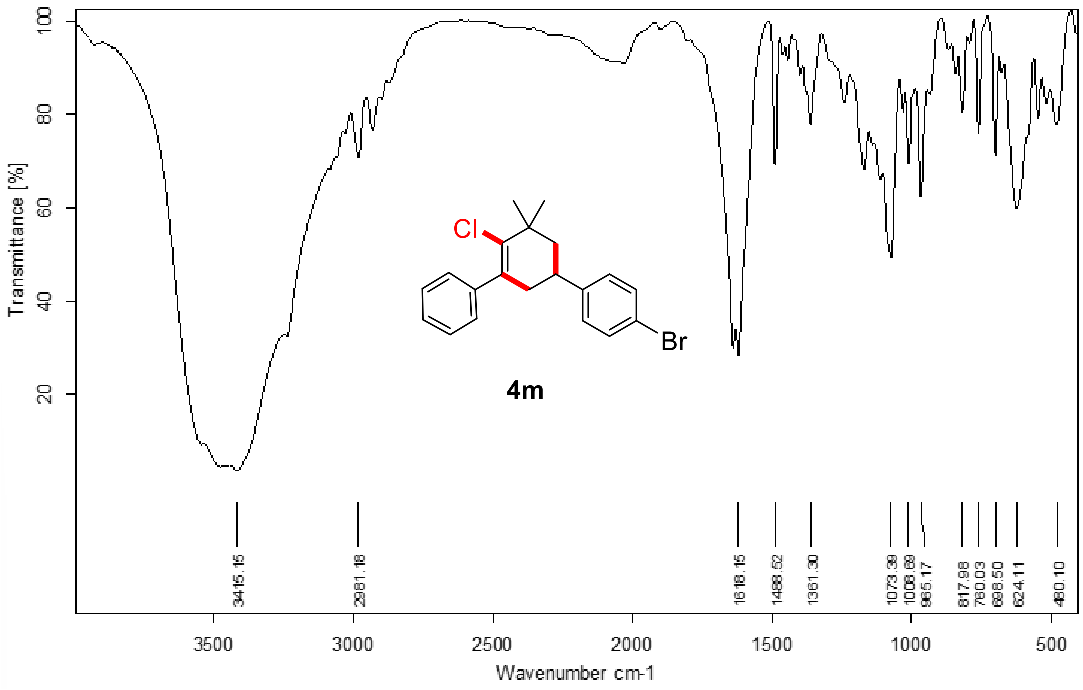
Transmittance [%]
Wavenumber cm-1
Cl
Br
3415.15
2981.18
1618.15
1488.52
1361.30
1073.39
1008.69
965.17
817.98
760.03
698.50
624.11
480.10

4m

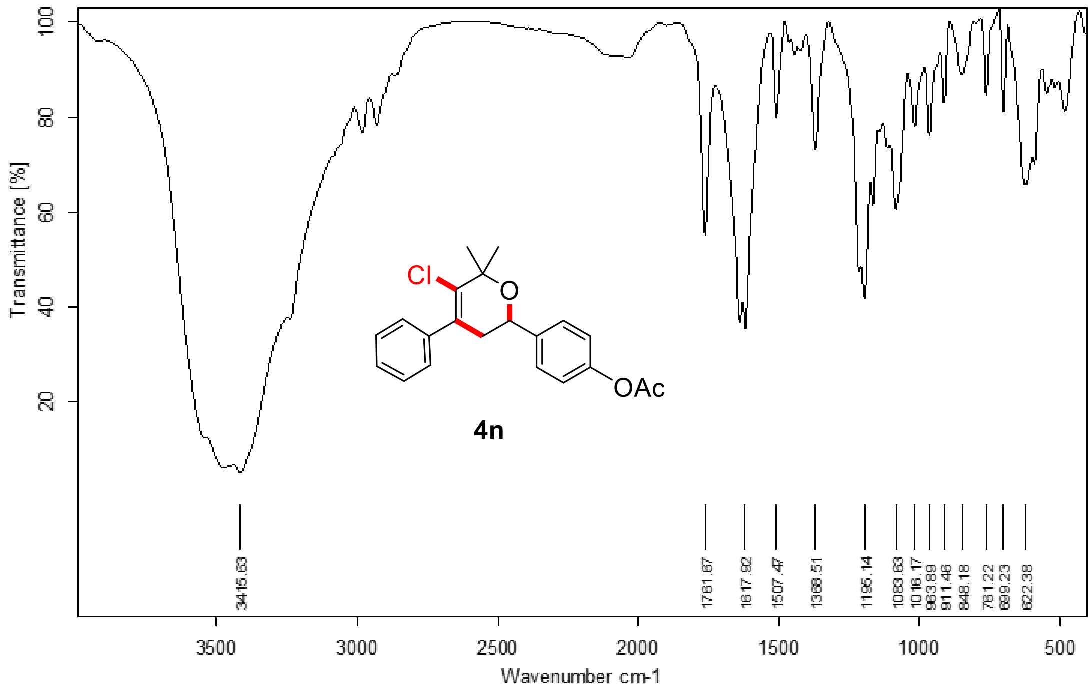
Transmittance [%]
Wavenumber cm-1
Cl
O
OAc
3415.63
1761.67
1617.92
1507.47
1368.51
1195.14
1083.63
1016.17
963.89
911.46
848.18
761.22
699.23
622.38

4n

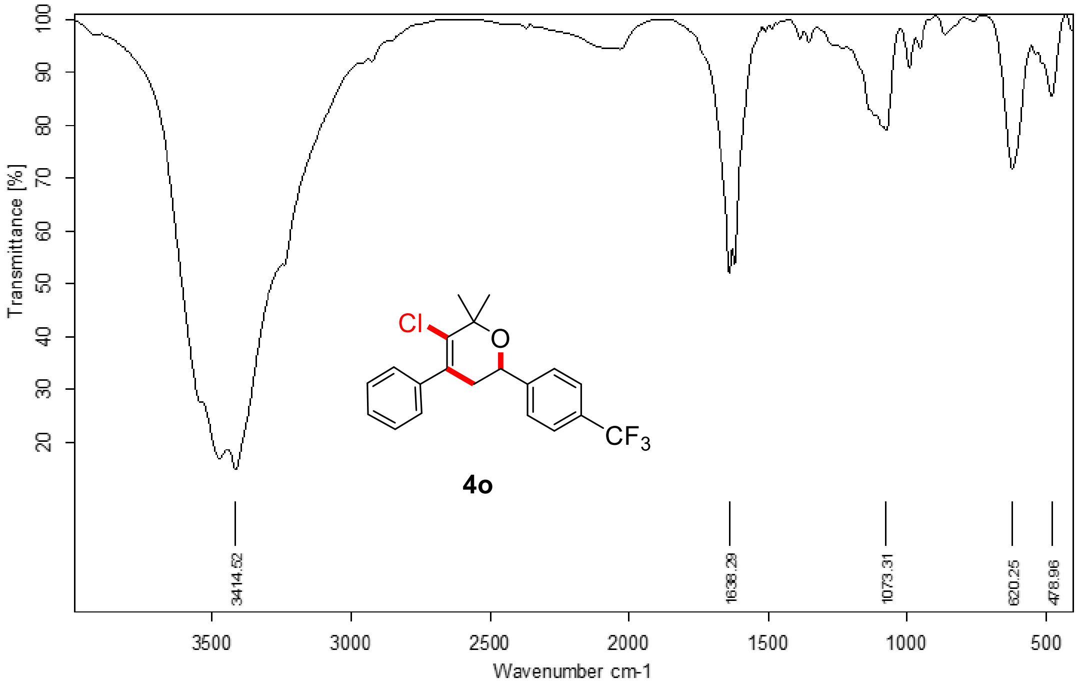
Transmittance [%]
Wavenumber cm-1
Cl
O
CF3
4o
3414.52
1638.29
1073.31
620.25
478.96

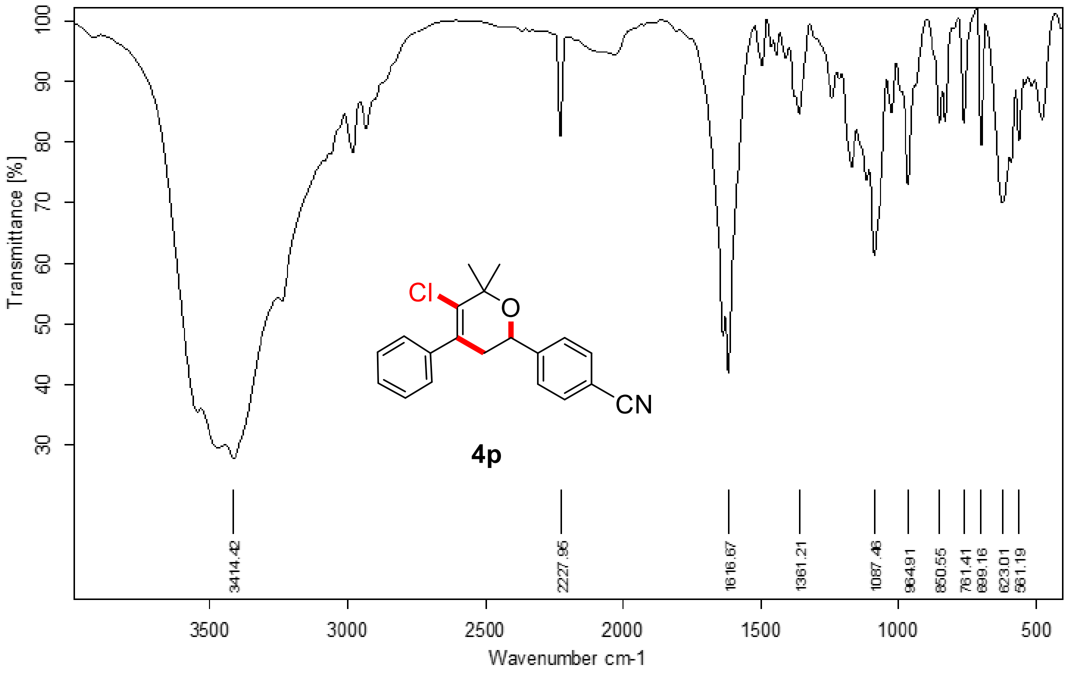
Transmittance [%]
Wavenumber cm-1
Cl
O
CN
4p
3414.42
2227.95
1616.67
1361.21
1087.48
964.91
850.55
761.41
699.16
623.01
561.19

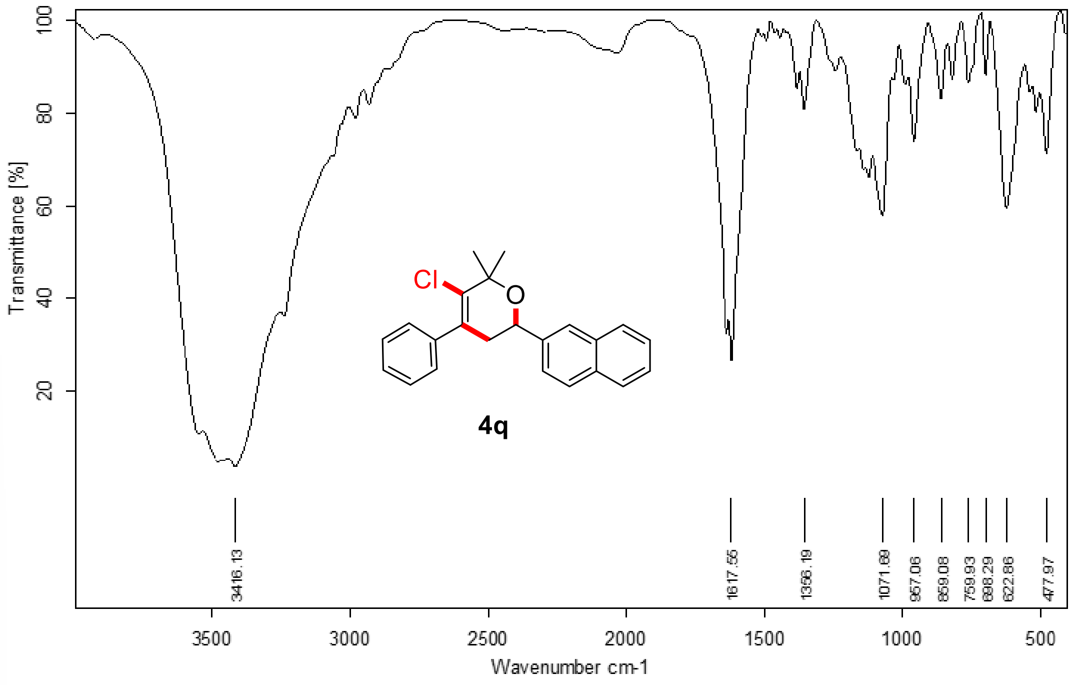
Transmittance [%]
Wavenumber cm-1
3416.13
1617.55
1366.19
1071.69
957.06
859.08
759.93
698.29
622.86
477.97
Cl
O
4q

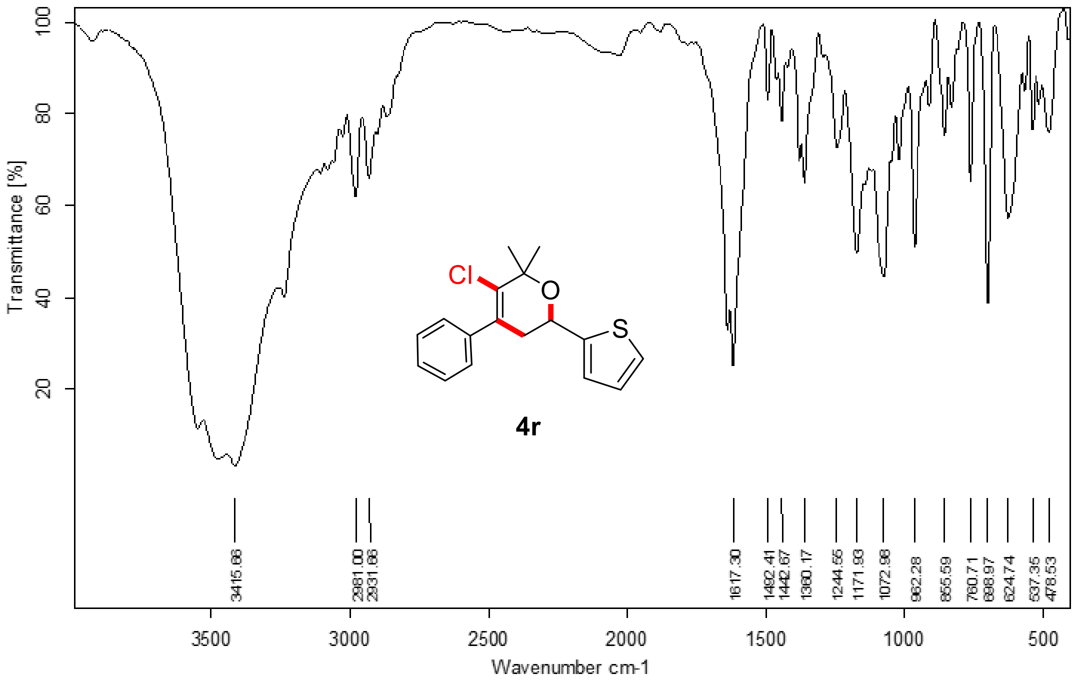
Transmittance [%]
Wavenumber cm-1
3415.66
2981.00
2931.66
1617.30
1492.41
1442.67
1360.17
1244.55
1171.93
1072.98
962.28
855.59
760.71
698.97
624.74
537.35
478.53
Cl
O
S
4r

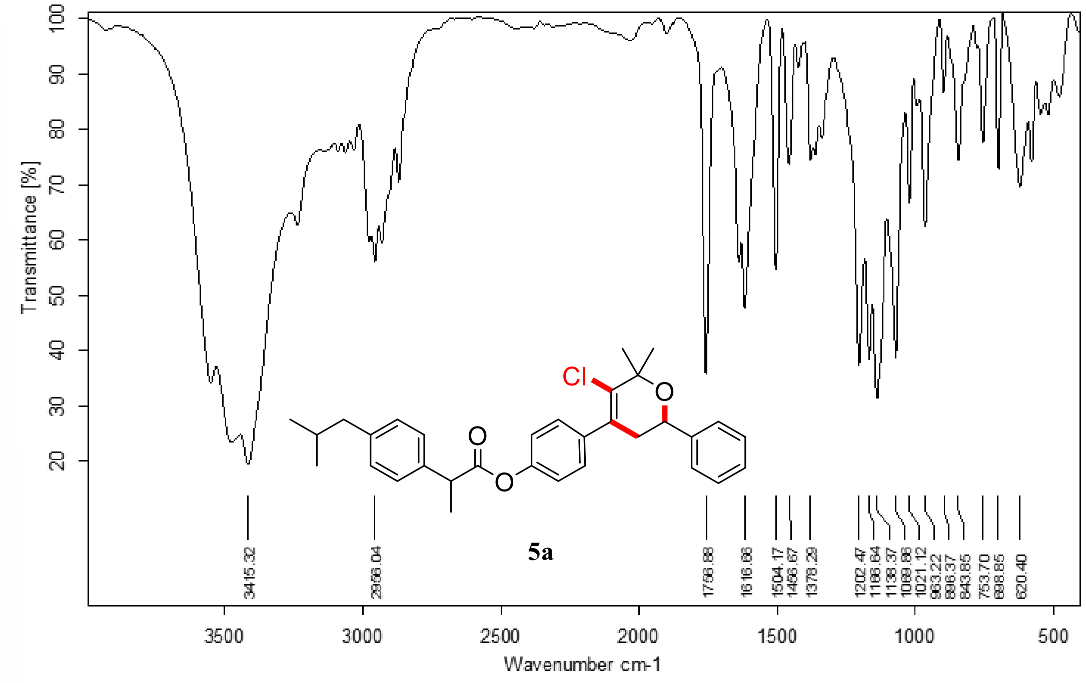
Transmittance [%]
Wavenumber cm-1
Cl
O
5a
3415.32
2956.04
1756.88
1616.66
1504.17
1456.67
1378.29
1202.47
1166.64
1138.37
1089.88
1021.12
963.22
896.37
843.85
753.70
698.85
620.40

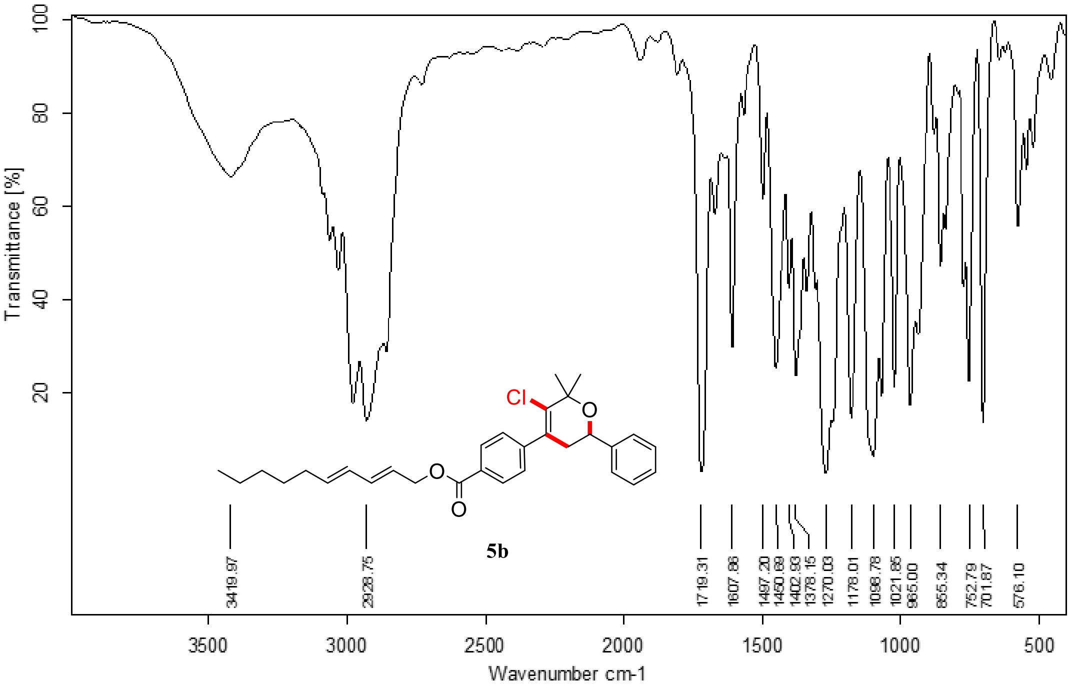
Transmittance [%]
Wavenumber cm-1
Cl
O
5b
3419.97
2928.75
1719.31
1607.86
1497.20
1450.69
1402.93
1378.15
1270.03
1178.01
1098.78
1021.85
965.00
865.34
752.79
701.87
576.10

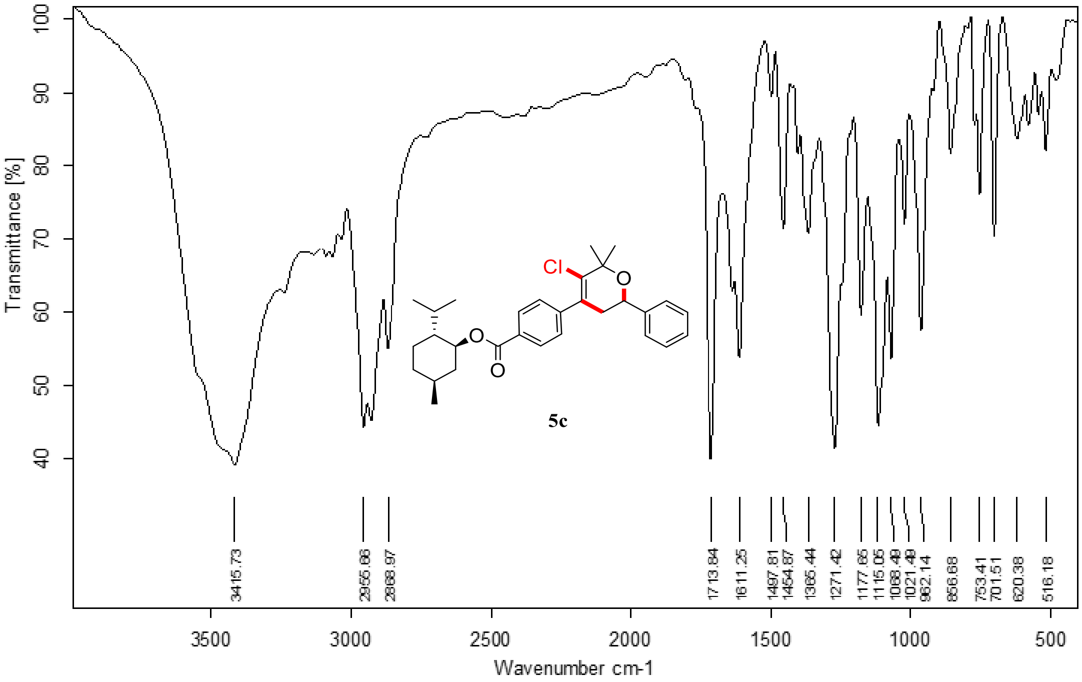
Transmittance [%]
Wavenumber cm-1
5c
3415.73
2955.86
2868.97
1713.84
1611.25
1497.81
1454.87
1365.44
1271.42
1177.65
1115.05
1068.49
1021.49
962.14
856.68
753.41
701.51
620.38
516.18

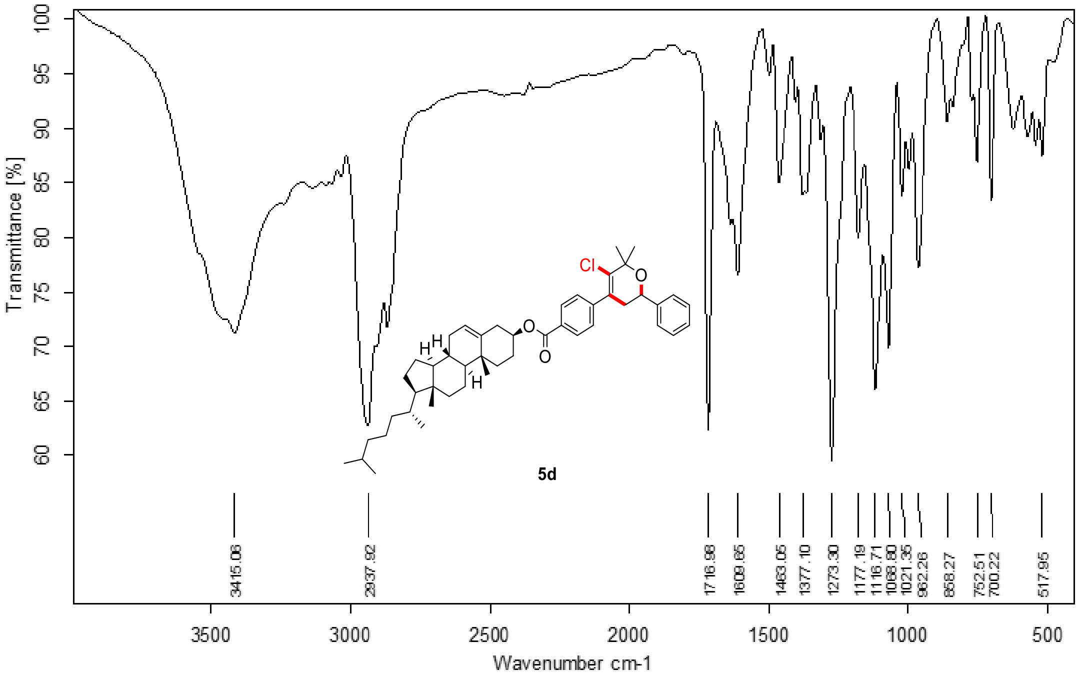
Transmittance [%]
Wavenumber cm-1
5d
3415.06
2937.92
1716.98
1609.65
1463.05
1377.10
1273.30
1177.19
1116.71
1068.80
1021.35
962.26
858.27
752.51
700.22
517.95

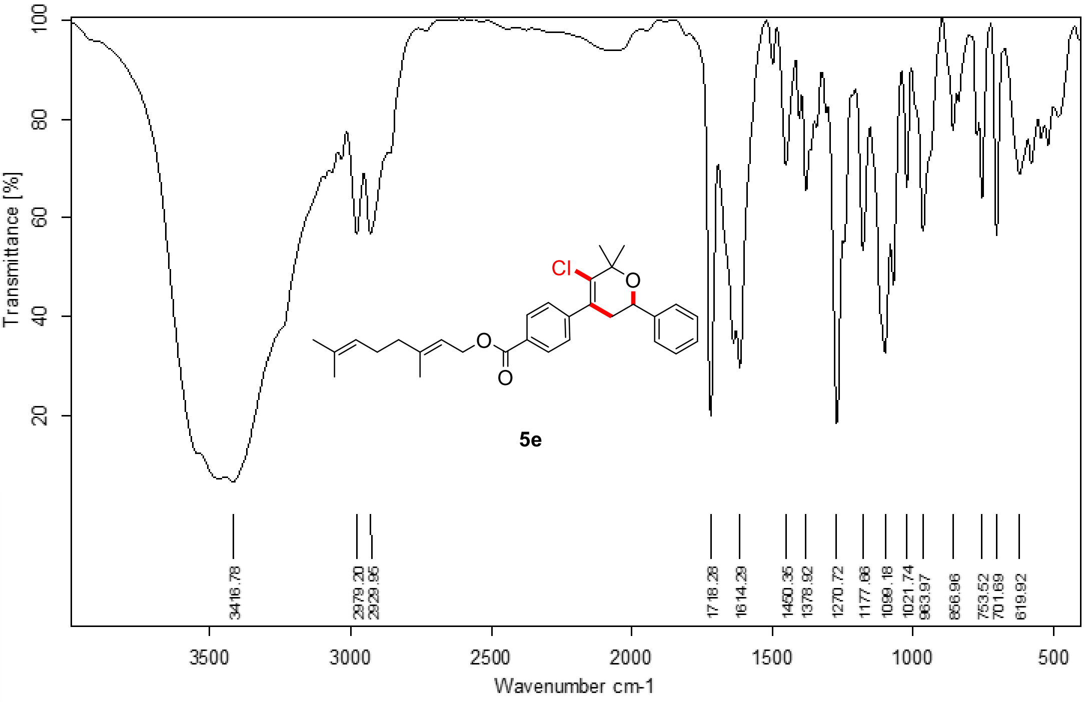
Transmittance [%]
100
80
60
40
20
Cl
O
O
O
5e
3416.78
2979.20
2929.95
1718.28
1614.29
1450.35
1378.92
1270.72
1177.66
1099.18
1021.74
963.97
856.96
753.52
701.69
619.92
3500
3000
2500
2000
1500
1000
500
Wavenumber cm-1

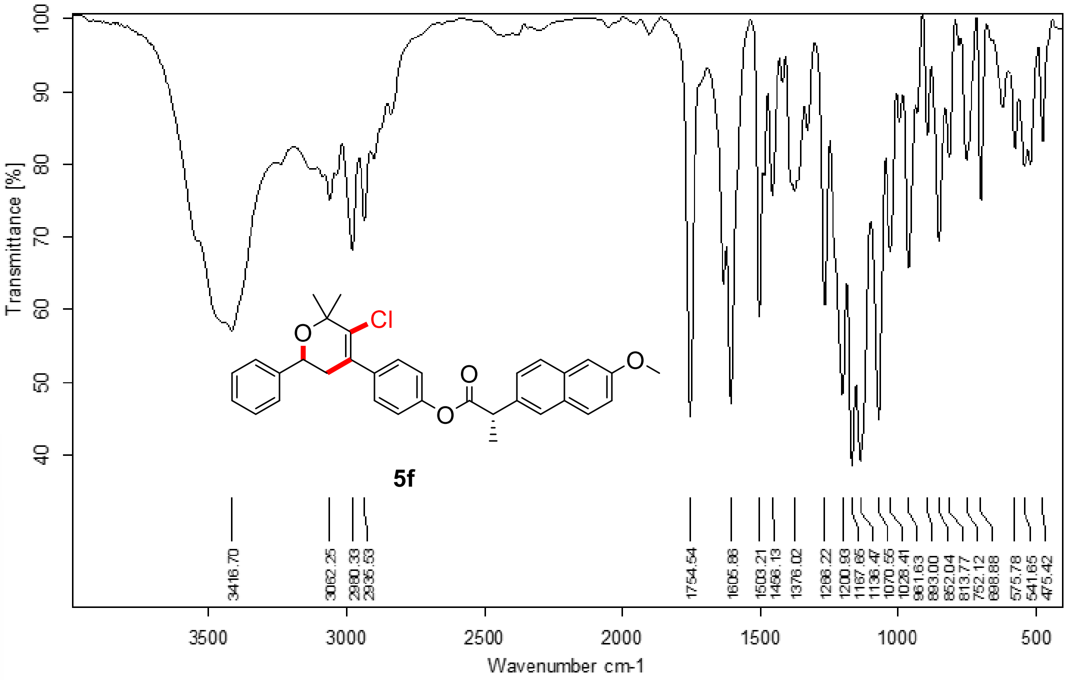
Transmittance [%]
100
90
80
70
60
50
40
O
Cl
O
O
O
5f
3416.70
3062.25
2980.33
2935.53
1754.54
1605.88
1503.21
1456.13
1376.02
1266.22
1200.93
1167.65
1136.47
1070.55
1028.41
961.63
893.00
852.04
813.77
752.12
698.88
575.78
541.65
475.42
3500
3000
2500
2000
1500
1000
500
Wavenumber cm-1

HRMS spectra of all compounds

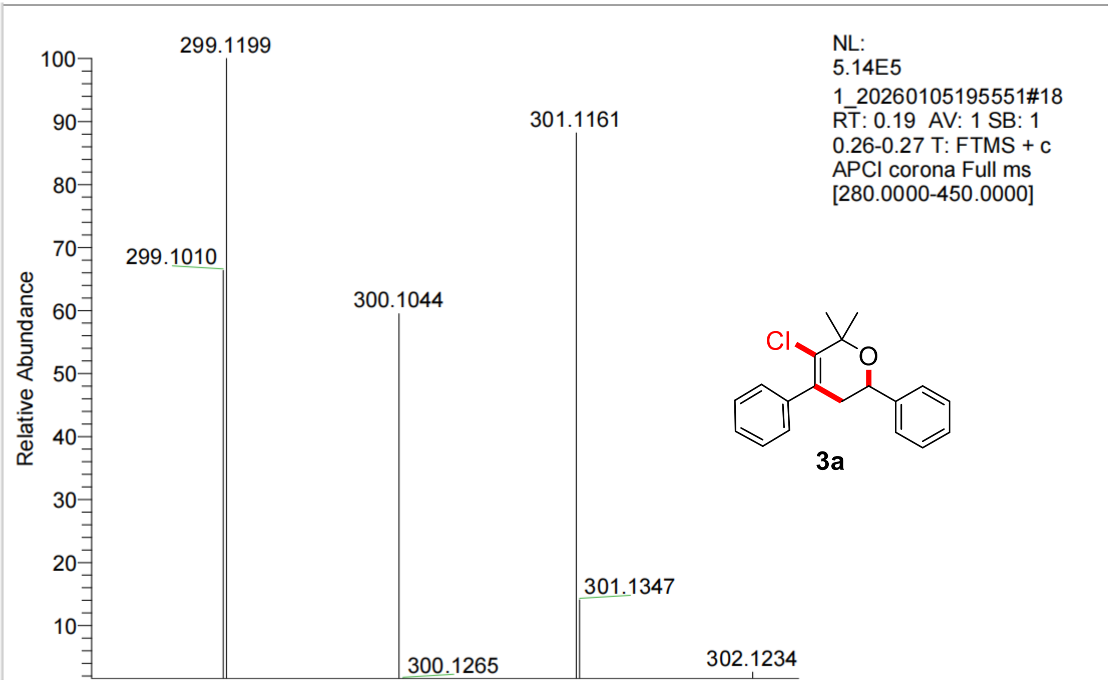

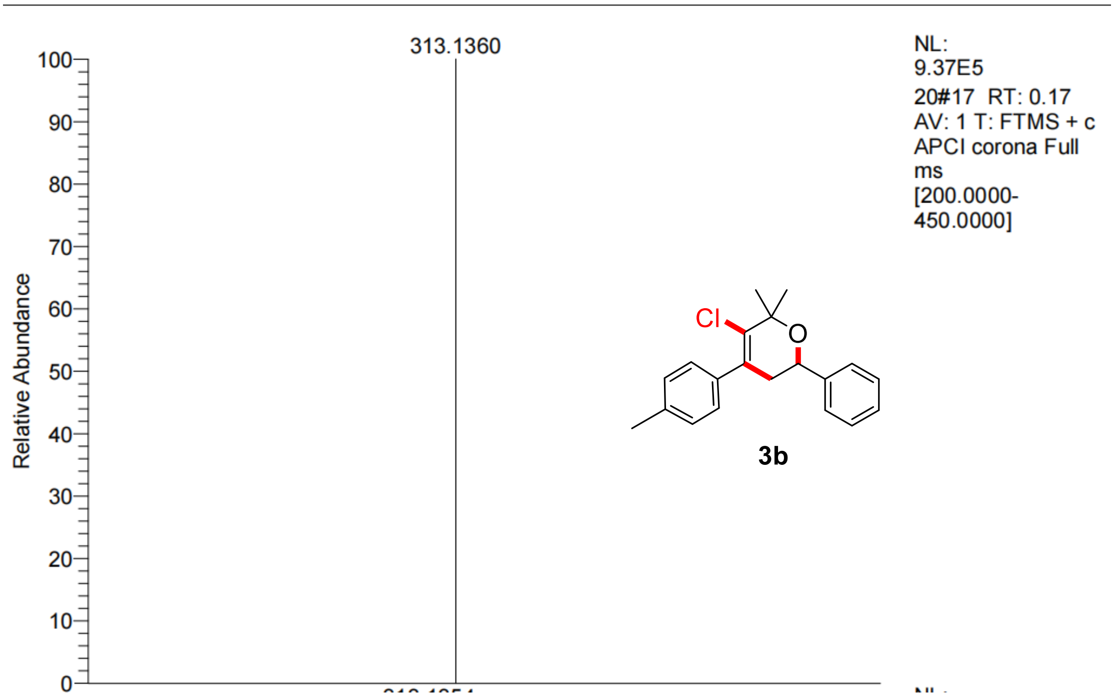

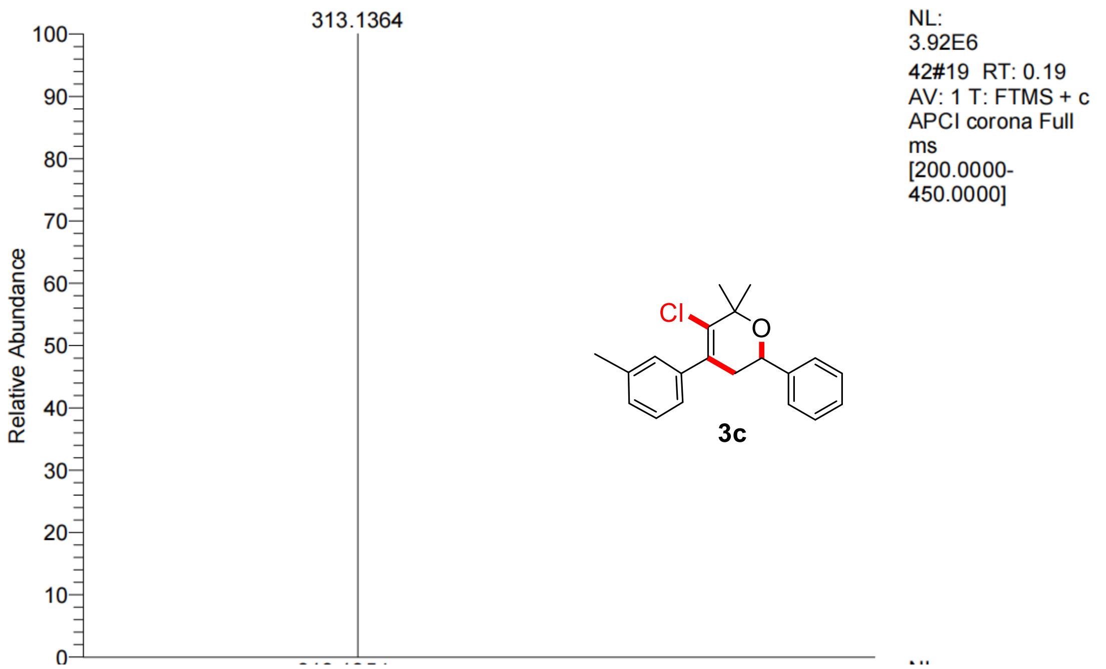
313.1364
NL:
3.92E6
42#19 RT: 0.19
AV: 1 T: FTMS + c
APCI corona Full
ms
[200.0000-
450.0000]
Relative Abundance
100
90
80
70
60
50
40
30
20
10
0
Cl
O
3c

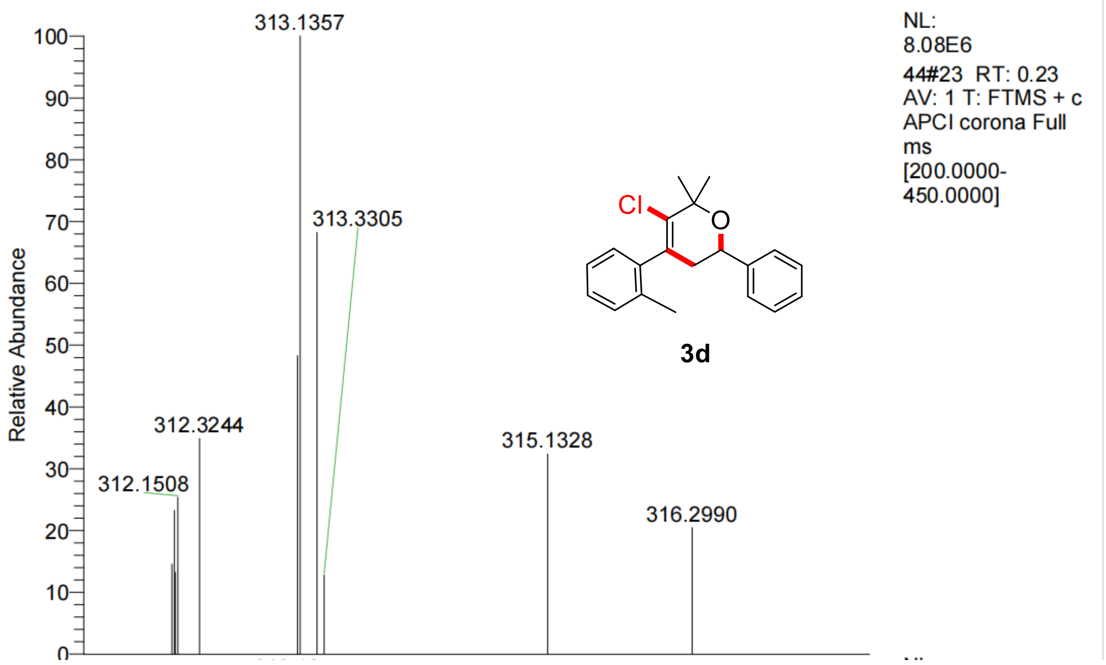
313.1357
313.3305
312.3244
312.1508
315.1328
316.2990
NL:
8.08E6
44#23 RT: 0.23
AV: 1 T: FTMS + c
APCI corona Full
ms
[200.0000-
450.0000]
Relative Abundance
100
90
80
70
60
50
40
30
20
10
0
Cl
O
3d

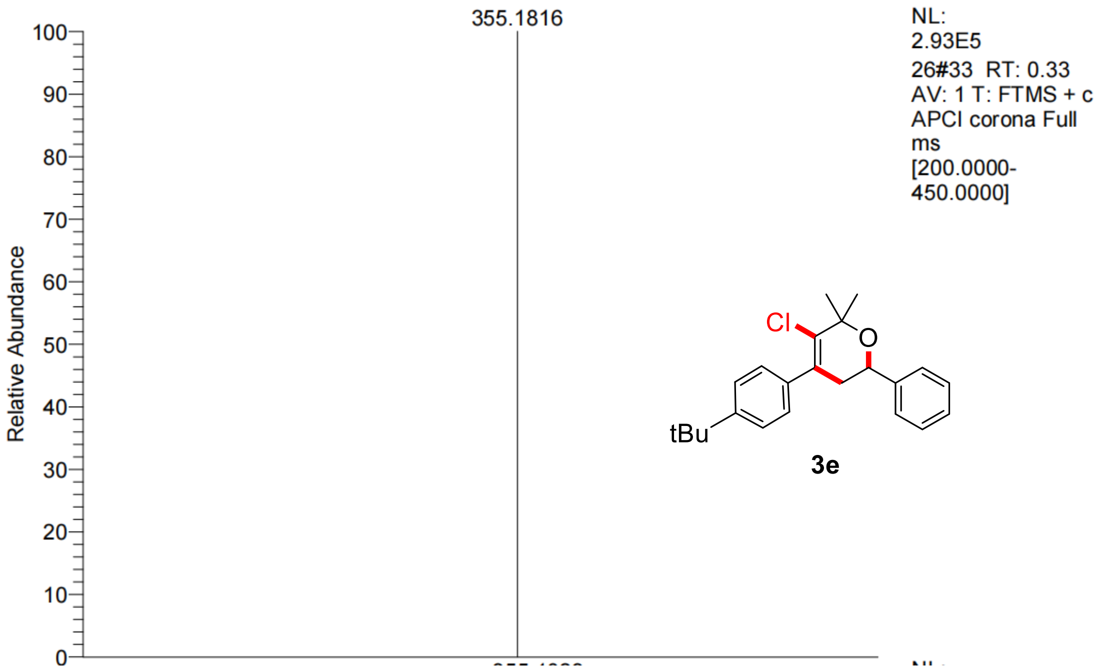
355.1816
NL:
2.93E5
26#33 RT: 0.33
AV: 1 T: FTMS + c
APCI corona Full
ms
[200.0000-
450.0000]
Relative Abundance
100
90
80
70
60
50
40
30
20
10
0
Cl
O
tBu
3e

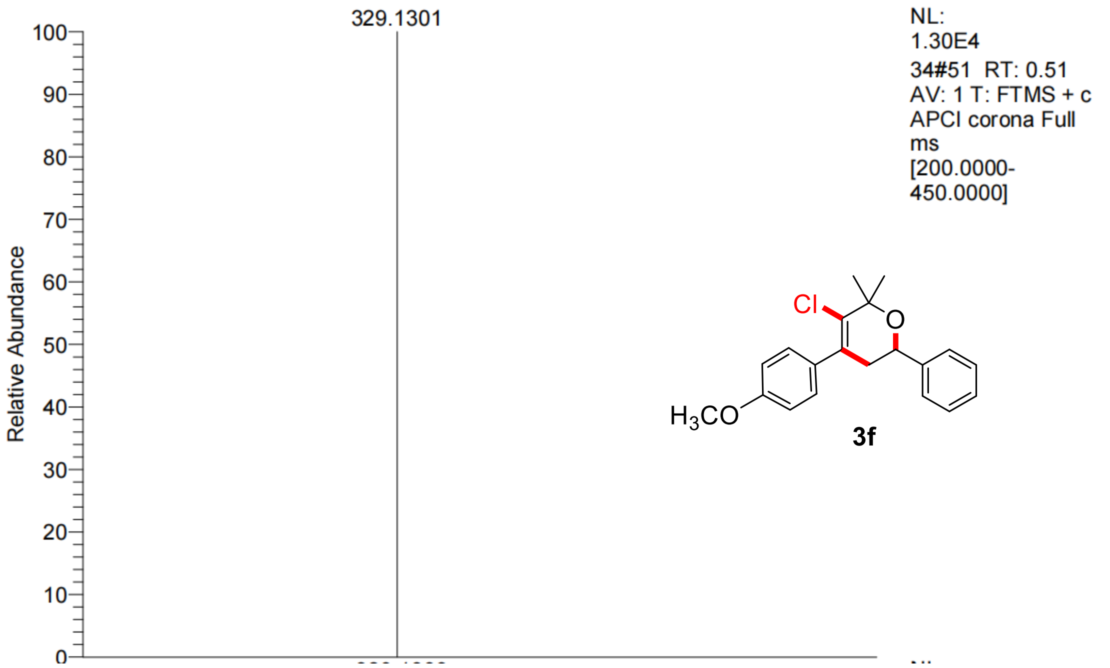
329.1301
NL:
1.30E4
34#51 RT: 0.51
AV: 1 T: FTMS + c
APCI corona Full
ms
[200.0000-
450.0000]
Relative Abundance
100
90
80
70
60
50
40
30
20
10
0
Cl
O
H3CO
3f

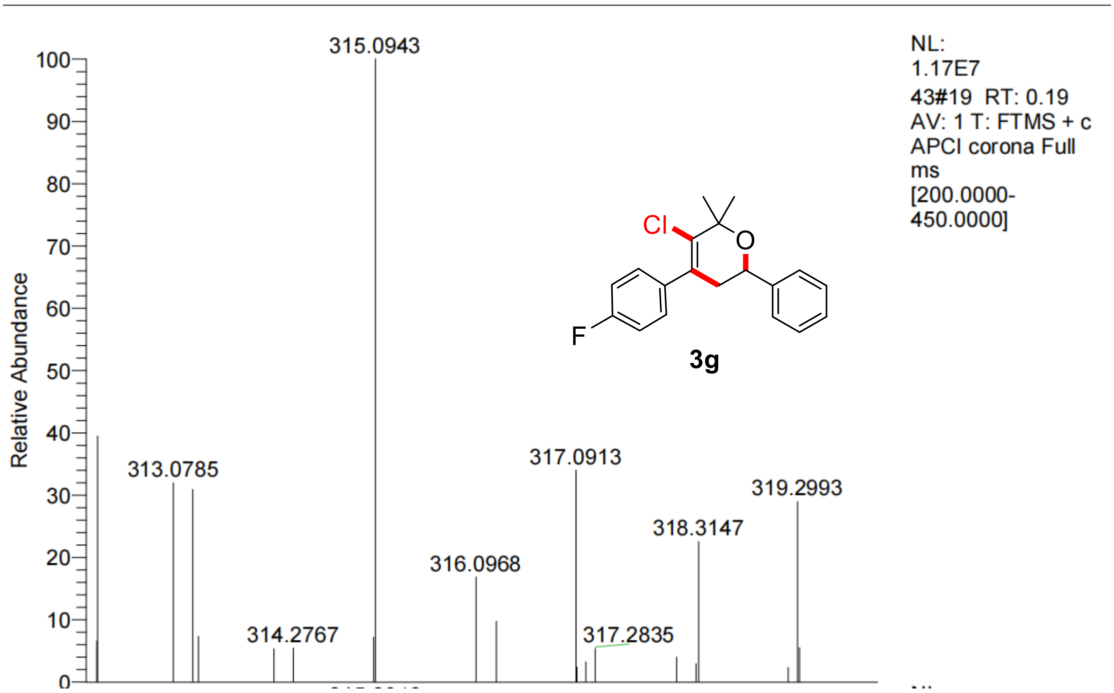
315.0943
NL:
1.17E7
43#19 RT: 0.19
AV: 1 T: FTMS + c
APCI corona Full
ms
[200.0000-
450.0000]
Relative Abundance
313.0785
314.2767
316.0968
317.0913
317.2835
318.3147
319.2993
Cl
O
F
3g

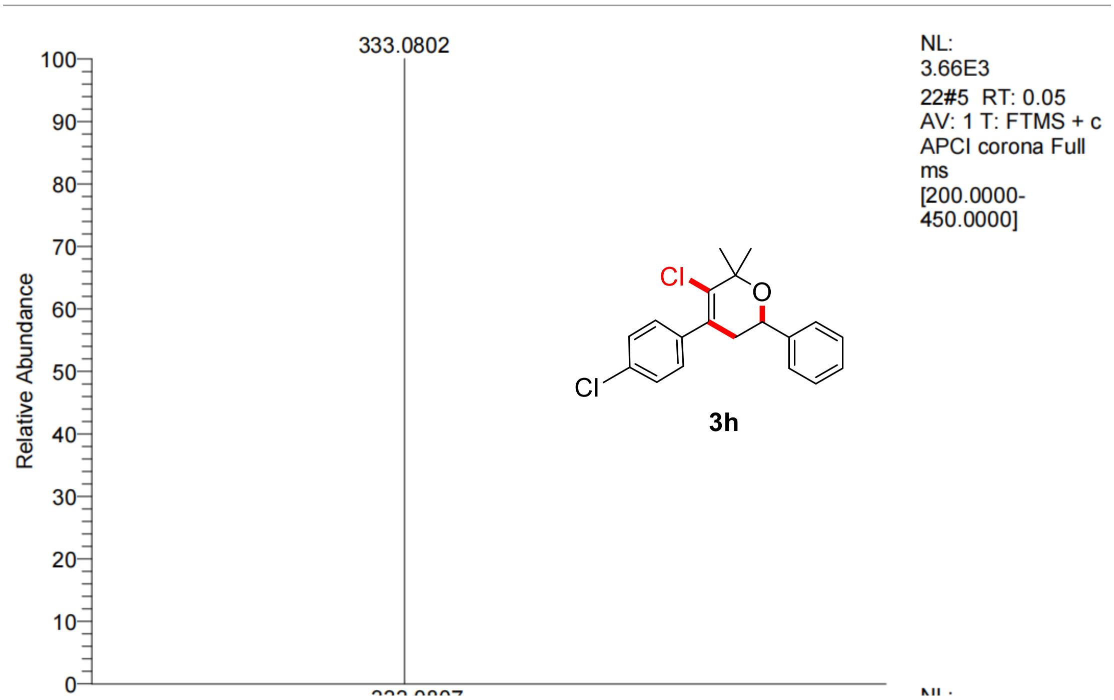
333.0802
NL:
3.66E3
22#5 RT: 0.05
AV: 1 T: FTMS + c
APCI corona Full
ms
[200.0000-
450.0000]
Relative Abundance
Cl
O
Cl
3h

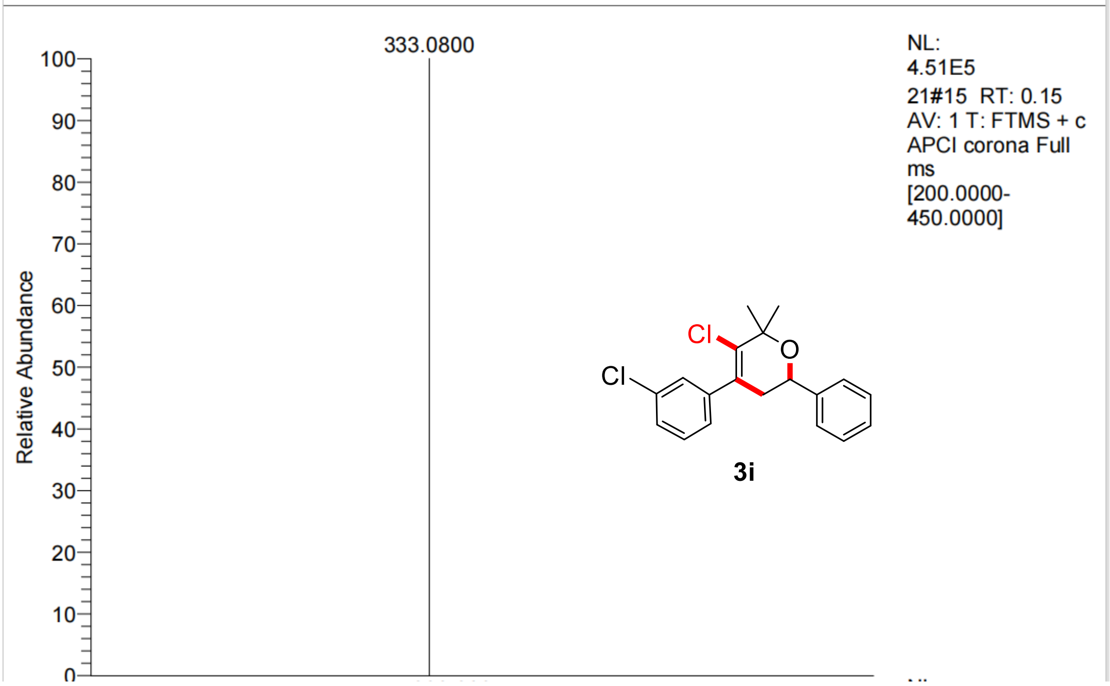
333.0800
NL:
4.51E5
21#15 RT: 0.15
AV: 1 T: FTMS + c
APCI corona Full
ms
[200.0000-
450.0000]
Relative Abundance
Cl
Cl
O
3i

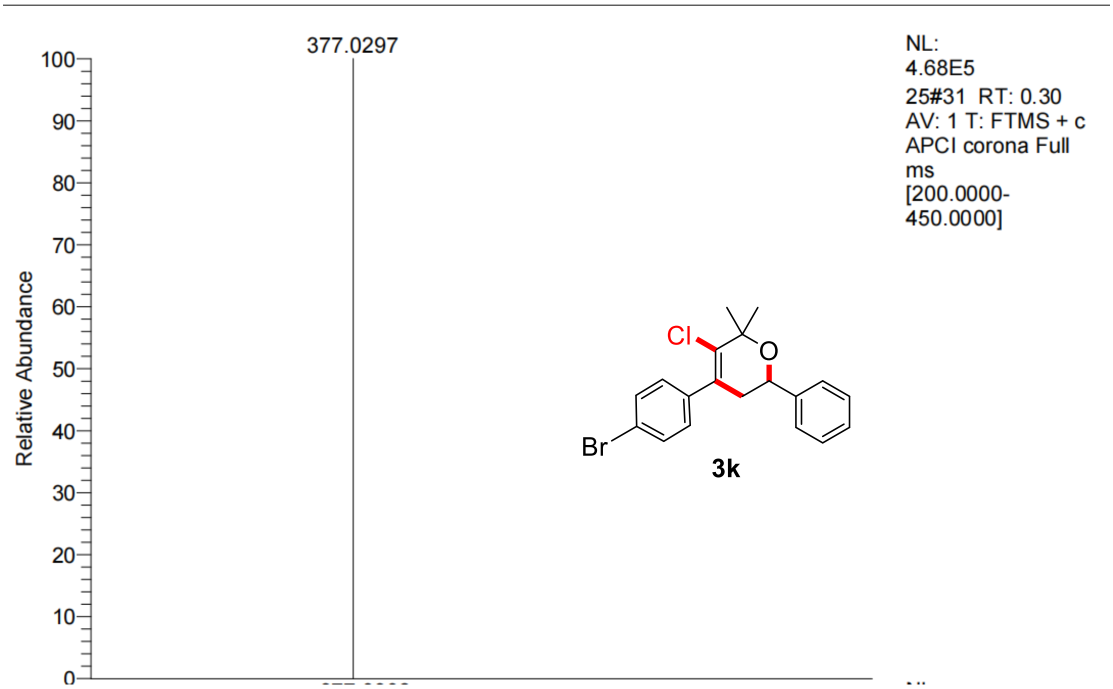
377.0297
NL:
4.68E5
25#31 RT: 0.30
AV: 1 T: FTMS + c
APCI corona Full
ms
[200.0000-
450.0000]
Relative Abundance
Cl
O
Br
3k

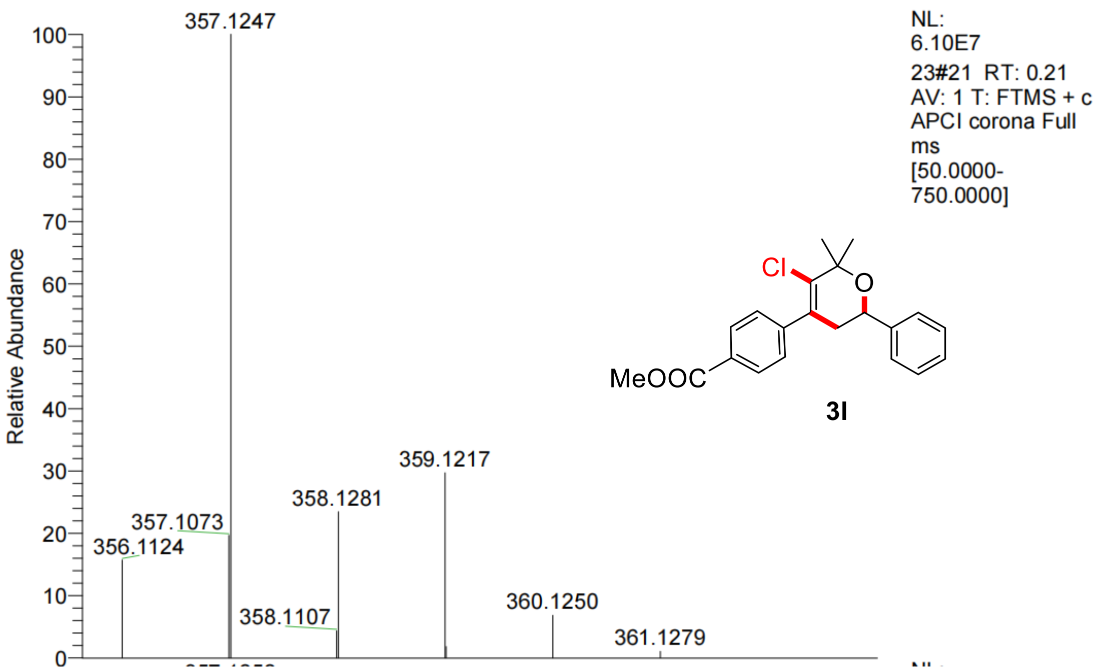

NL:
6.10E7
23#21 RT: 0.21
AV: 1 T: FTMS + c
APCI corona Full
ms
[50.0000-
750.0000]
Relative Abundance
357.1247
359.1217
358.1281
357.1073
356.1124
358.1107
360.1250
361.1279
Cl
O
MeOOC
3l

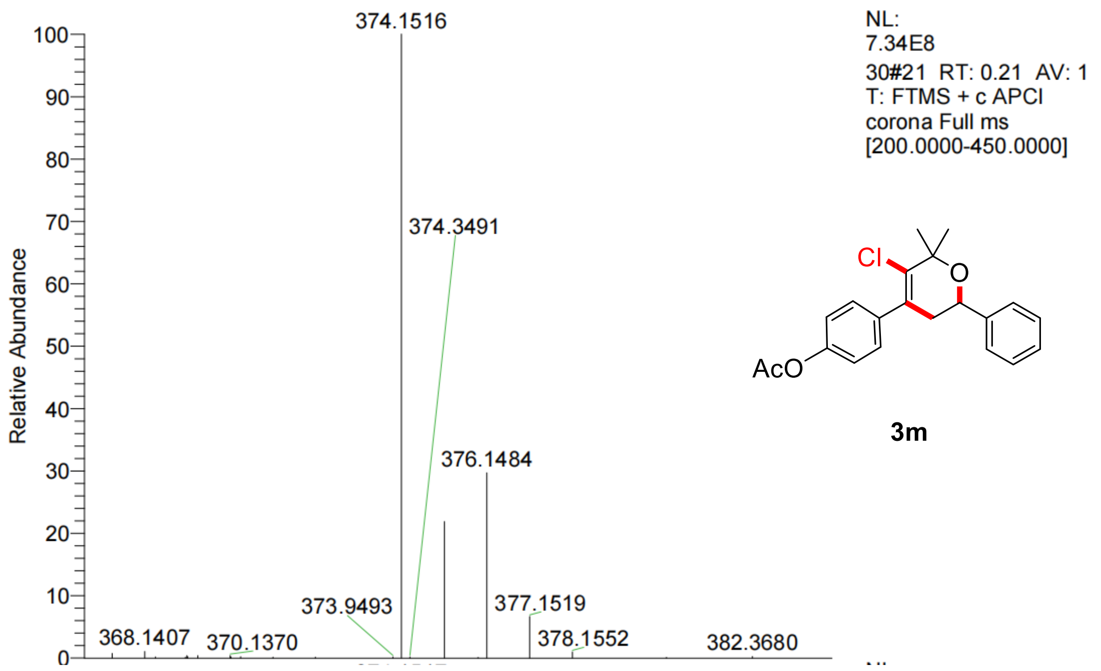

NL:
7.34E8
30#21 RT: 0.21 AV: 1
T: FTMS + c APCI
corona Full ms
[200.0000-450.0000]
Relative Abundance
374.1516
374.3491
376.1484
373.9493
377.1519
368.1407
370.1370
378.1552
382.3680
Cl
O
AcO
3m

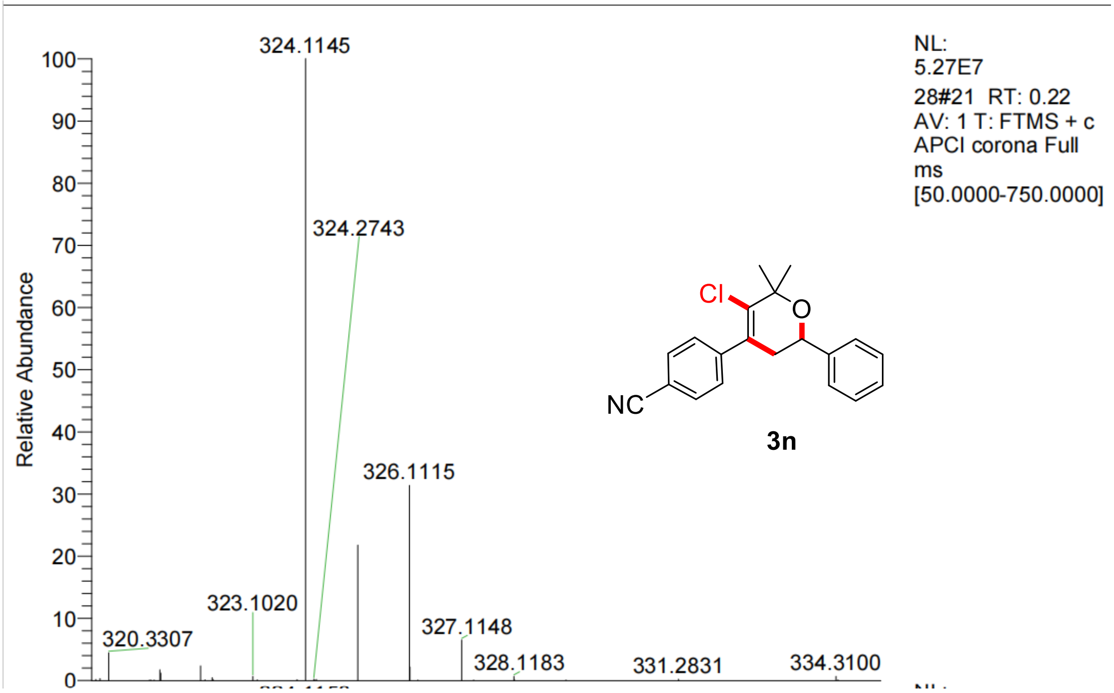

324.1145
324.2743
326.1115
323.1020
320.3307
327.1148
328.1183
331.2831
334.3100
Relative Abundance
NL:
5.27E7
28#21 RT: 0.22
AV: 1 T: FTMS + c
APCI corona Full
ms
[50.0000-750.0000]
Cl
O
NC
3n

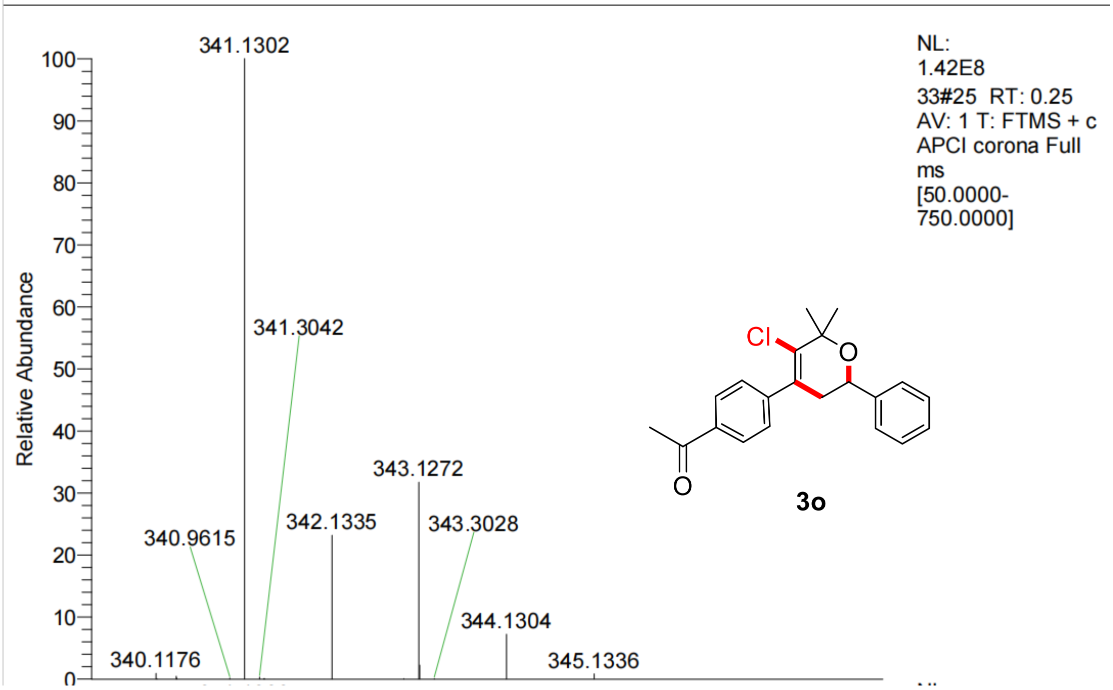

341.1302
341.3042
343.1272
342.1335
343.3028
340.9615
344.1304
340.1176
345.1336
Relative Abundance
NL:
1.42E8
33#25 RT: 0.25
AV: 1 T: FTMS + c
APCI corona Full
ms
[50.0000-
750.0000]
Cl
O
O
3o

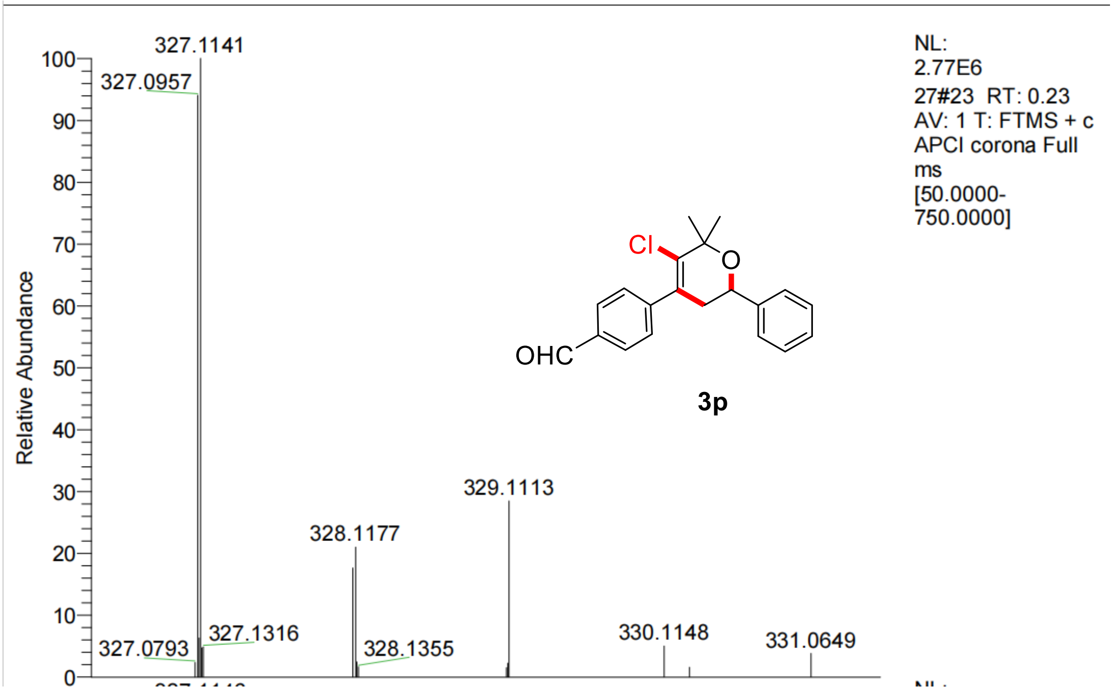
327.1141
327.0957
100
90
80
70
60
50
40
30
20
10
0
Relative Abundance
329.1113
328.1177
327.1316
327.0793
328.1355
330.1148
331.0649
NL:
2.77E6
27#23 RT: 0.23
AV: 1 T: FTMS + c
APCI corona Full
ms
[50.0000-
750.0000]
Cl
O
OHC
3p

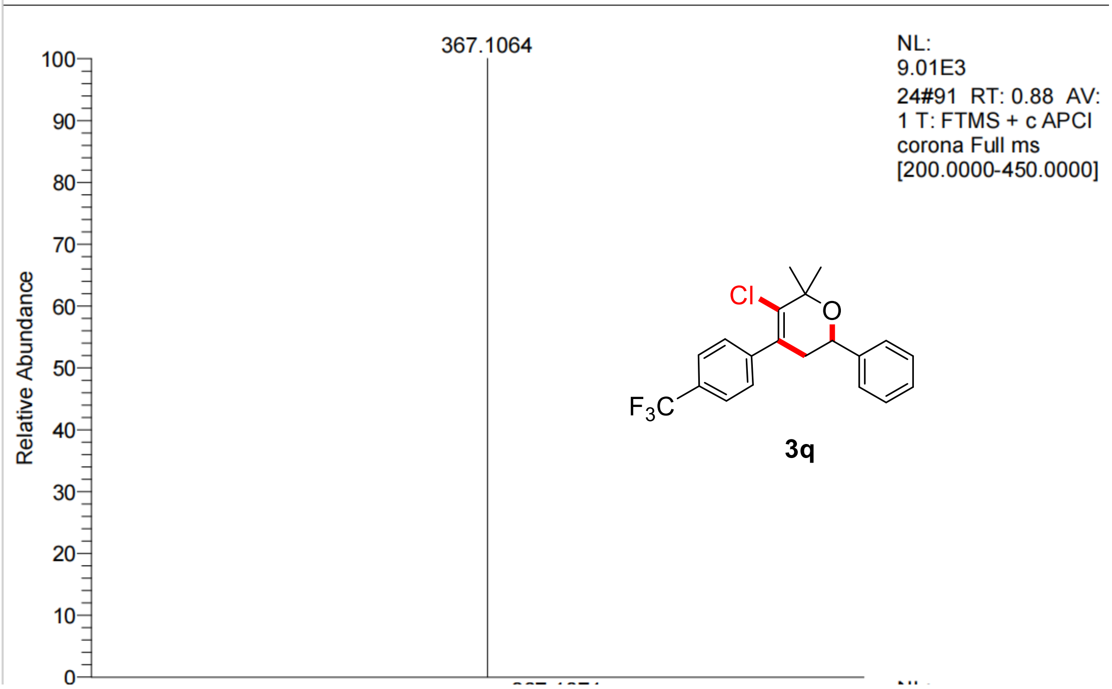
367.1064
100
90
80
70
60
50
40
30
20
10
0
Relative Abundance
NL:
9.01E3
24#91 RT: 0.88 AV:
1 T: FTMS + c APCI
corona Full ms
[200.0000-450.0000]
Cl
O
F3C
3q

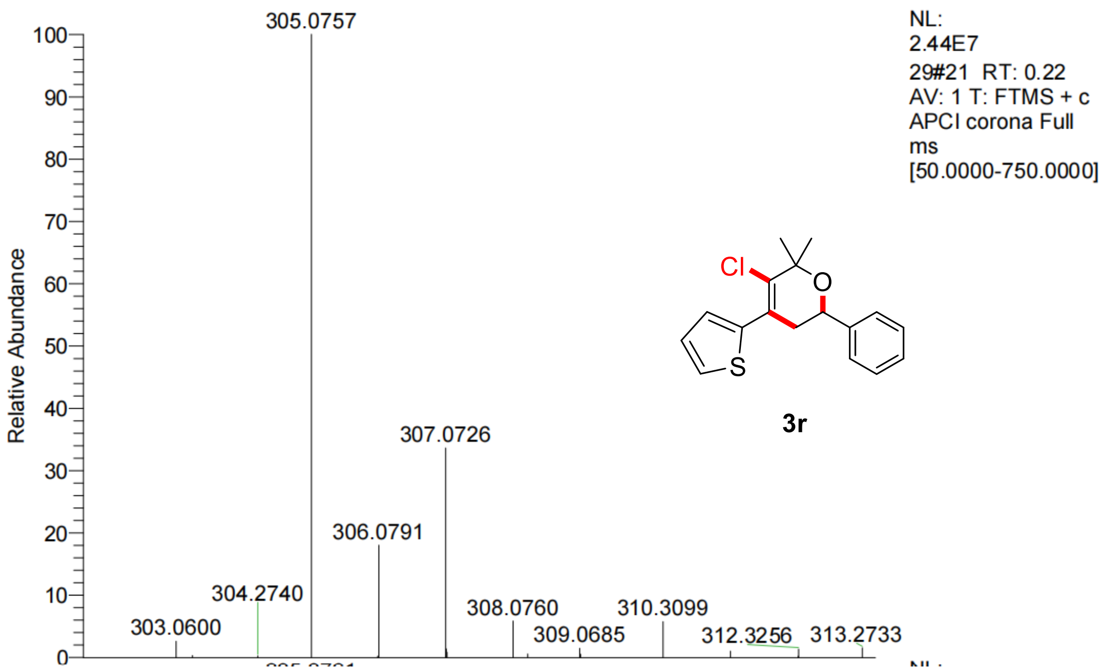
305.0757
307.0726
306.0791
304.2740
303.0600
308.0760
309.0685
310.3099
312.3256
313.2733
Relative Abundance
100
90
80
70
60
50
40
30
20
10
0
NL:
2.44E7
29#21 RT: 0.22
AV: 1 T: FTMS + c
APCI corona Full
ms
[50.0000-750.0000]
Cl
O
S
3r

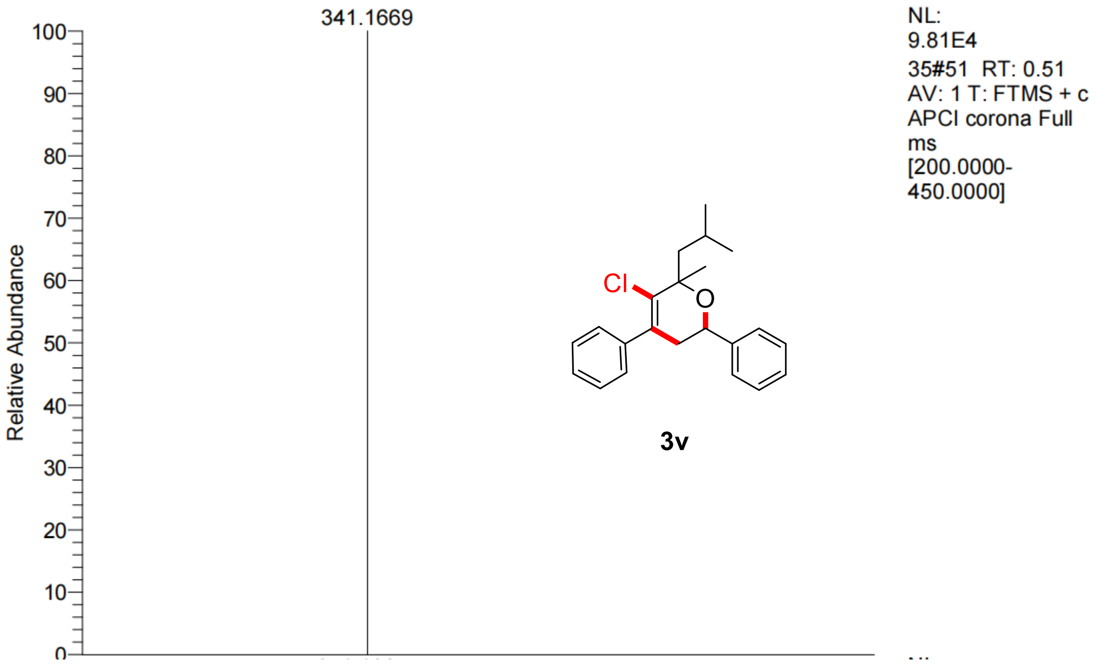
341.1669
Relative Abundance
100
90
80
70
60
50
40
30
20
10
0
NL:
9.81E4
35#51 RT: 0.51
AV: 1 T: FTMS + c
APCI corona Full
ms
[200.0000-
450.0000]
Cl
O
3v

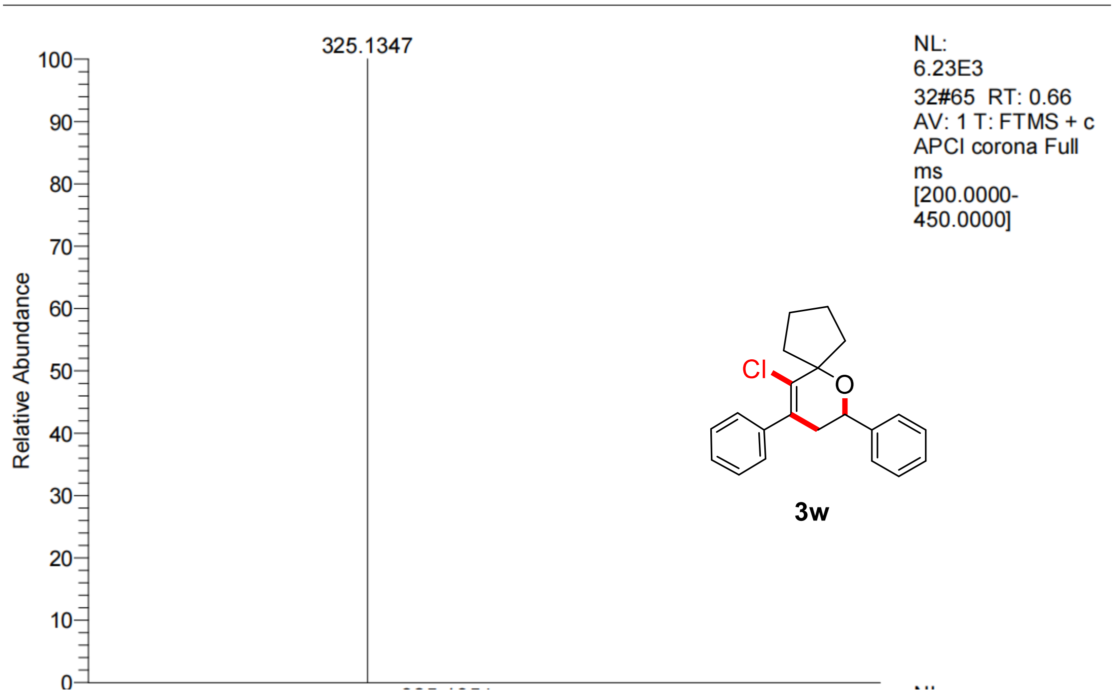
325.1347
NL:
6.23E3
32#65 RT: 0.66
AV: 1 T: FTMS + c
APCI corona Full
ms
[200.0000-
450.0000]
Relative Abundance
100
90
80
70
60
50
40
30
20
10
0
Cl
O
3w

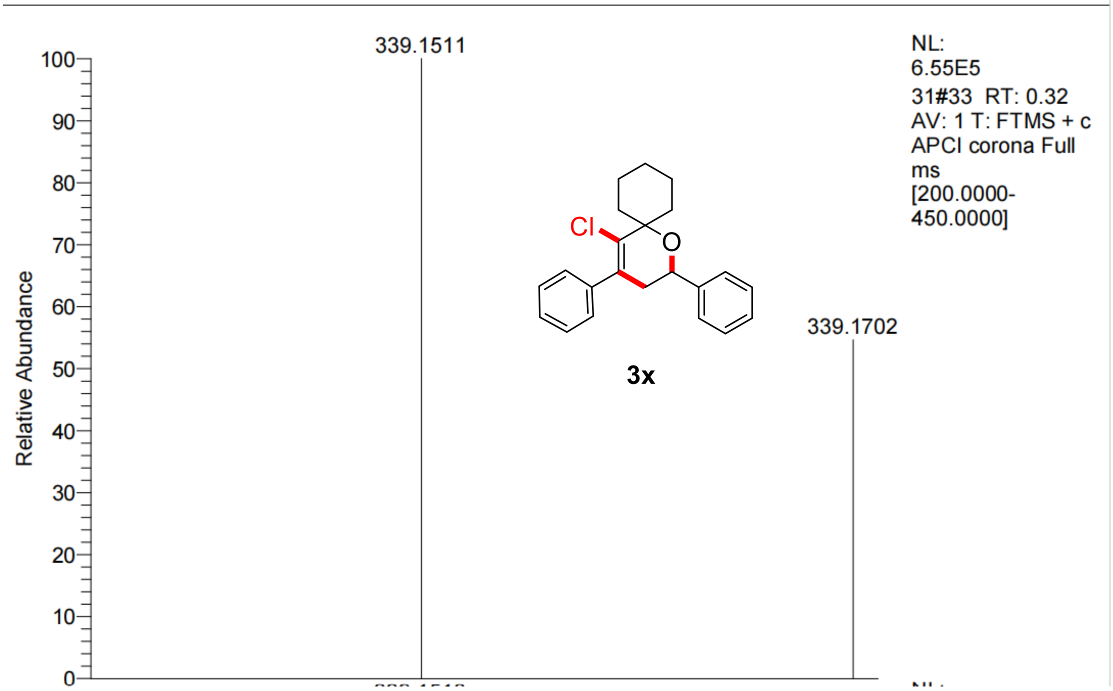
339.1511
NL:
6.55E5
31#33 RT: 0.32
AV: 1 T: FTMS + c
APCI corona Full
ms
[200.0000-
450.0000]
339.1702
Relative Abundance
100
90
80
70
60
50
40
30
20
10
0
Cl
O
3x

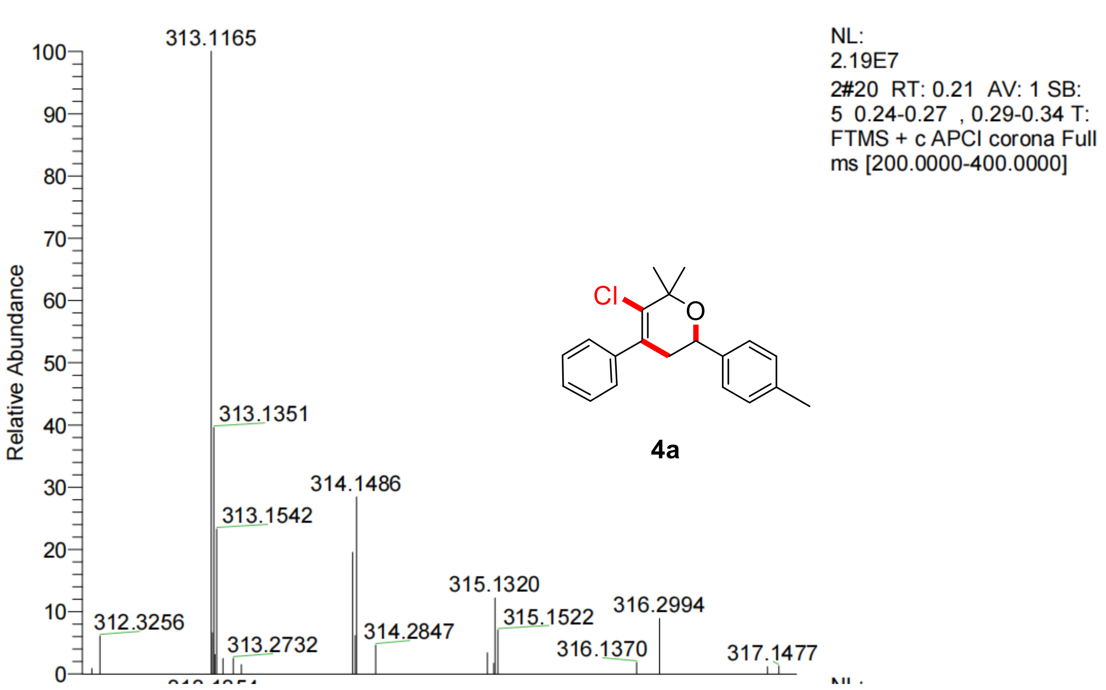
313.1165
NL:
2.19E7
2#20 RT: 0.21 AV: 1 SB:
5 0.24-0.27 , 0.29-0.34 T:
FTMS + c APCI corona Full
ms [200.0000-400.0000]
Relative Abundance
100
90
80
70
60
50
40
30
20
10
0
Cl
O
4a
313.1351
314.1486
313.1542
315.1320
312.3256
315.1522
316.2994
313.2732
314.2847
316.1370
317.1477

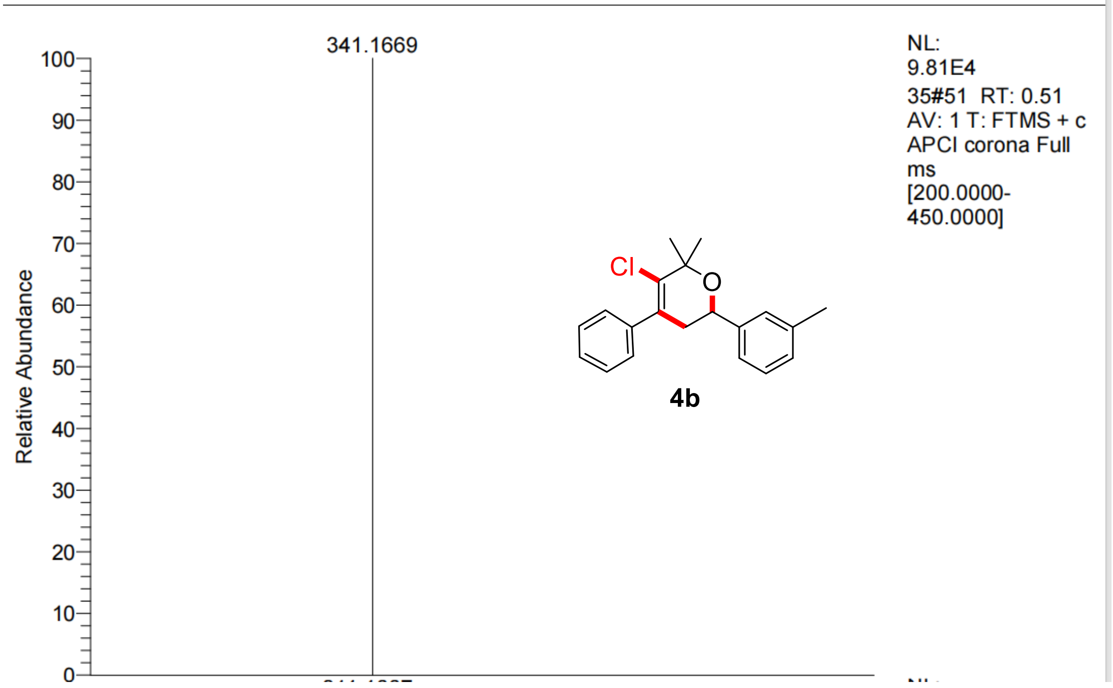
341.1669
NL:
9.81E4
35#51 RT: 0.51
AV: 1 T: FTMS + c
APCI corona Full
ms
[200.0000-
450.0000]
Relative Abundance
100
90
80
70
60
50
40
30
20
10
0
Cl
O
4b

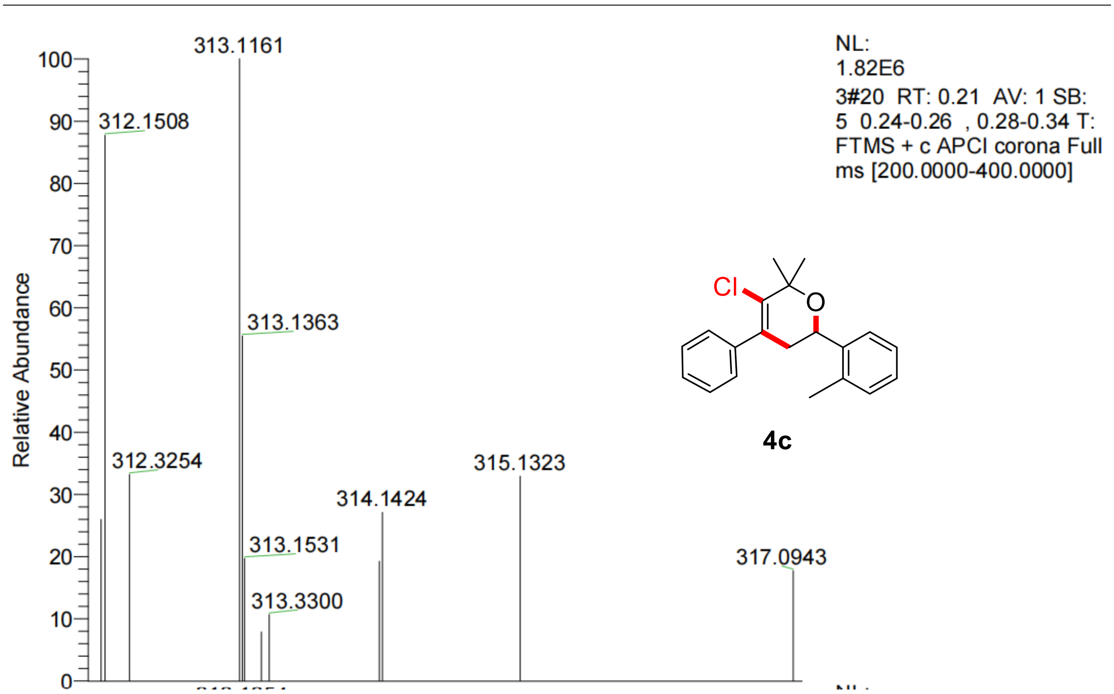
313.1161
100
90
312.1508
80
70
60
313.1363
50
40
312.3254
315.1323
30
314.1424
20
313.1531
317.0943
10
313.3300
0
Relative Abundance
NL:
1.82E6
3#20 RT: 0.21 AV: 1 SB:
5 0.24-0.26 , 0.28-0.34 T:
FTMS + c APCI corona Full
ms [200.0000-400.0000]
Cl
O
4c

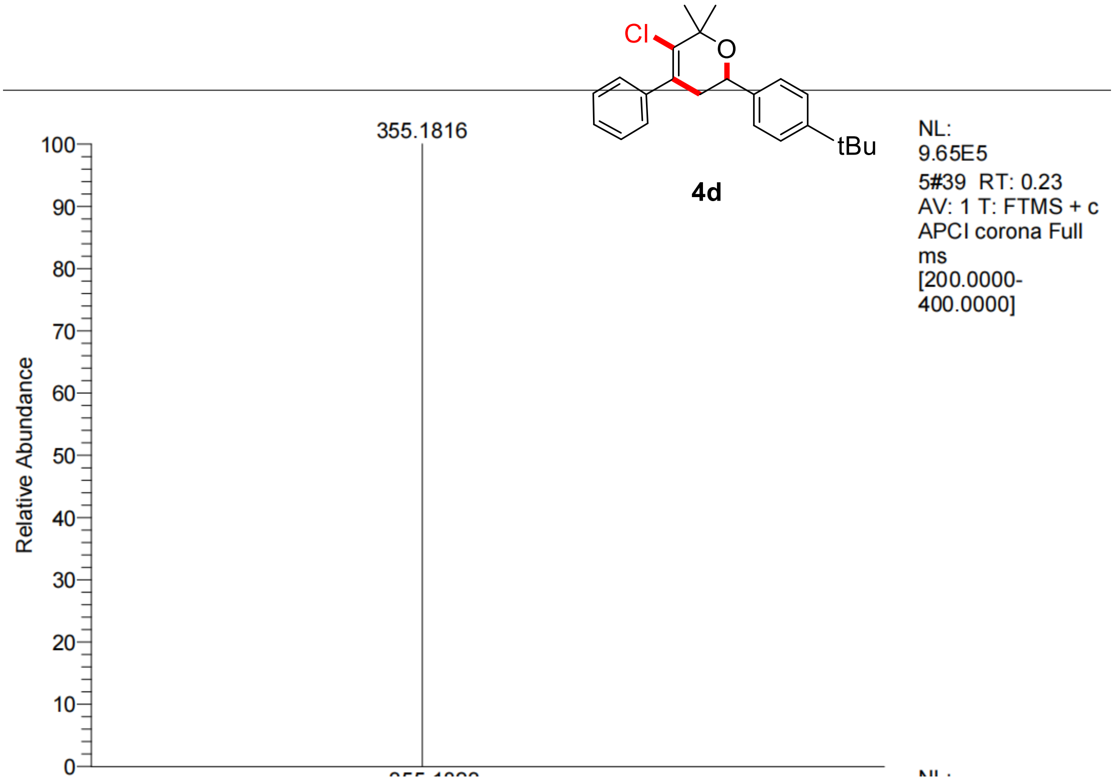
Cl
O
tBu
4d
355.1816
100
90
80
70
60
50
40
30
20
10
0
Relative Abundance
NL:
9.65E5
5#39 RT: 0.23
AV: 1 T: FTMS + c
APCI corona Full
ms
[200.0000-
400.0000]

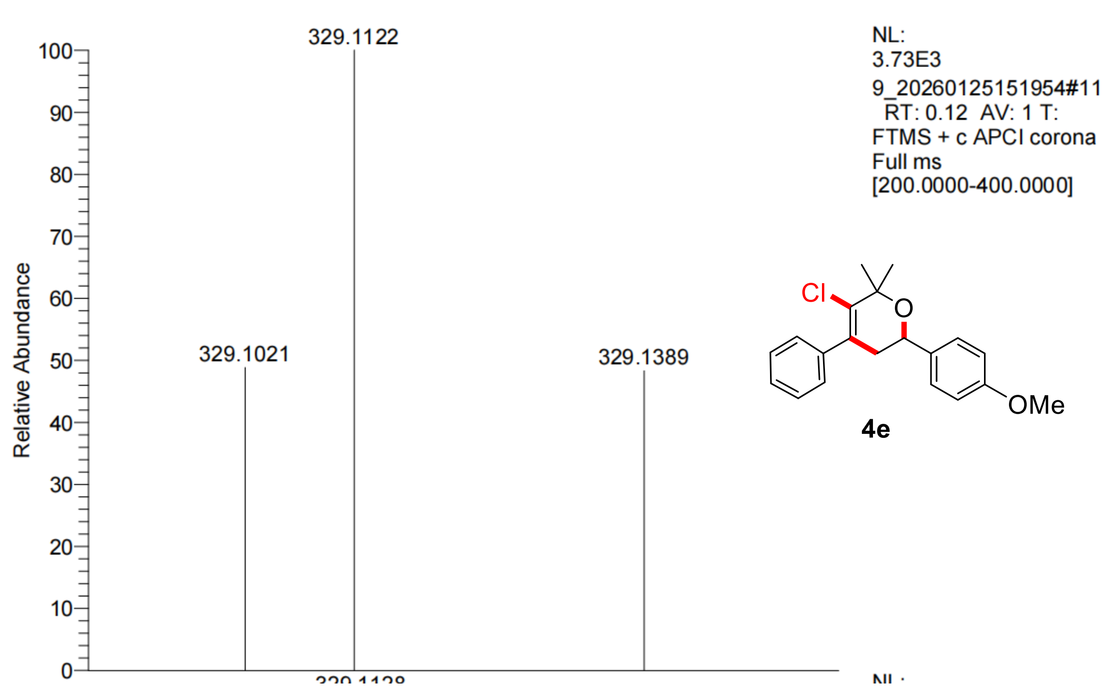

NL:
3.73E3
9_20260125151954#11
RT: 0.12 AV: 1 T:
FTMS + c APCI corona
Full ms
[200.0000-400.0000]
Relative Abundance
329.1122
329.1021
329.1389
Cl
O
OMe
4e

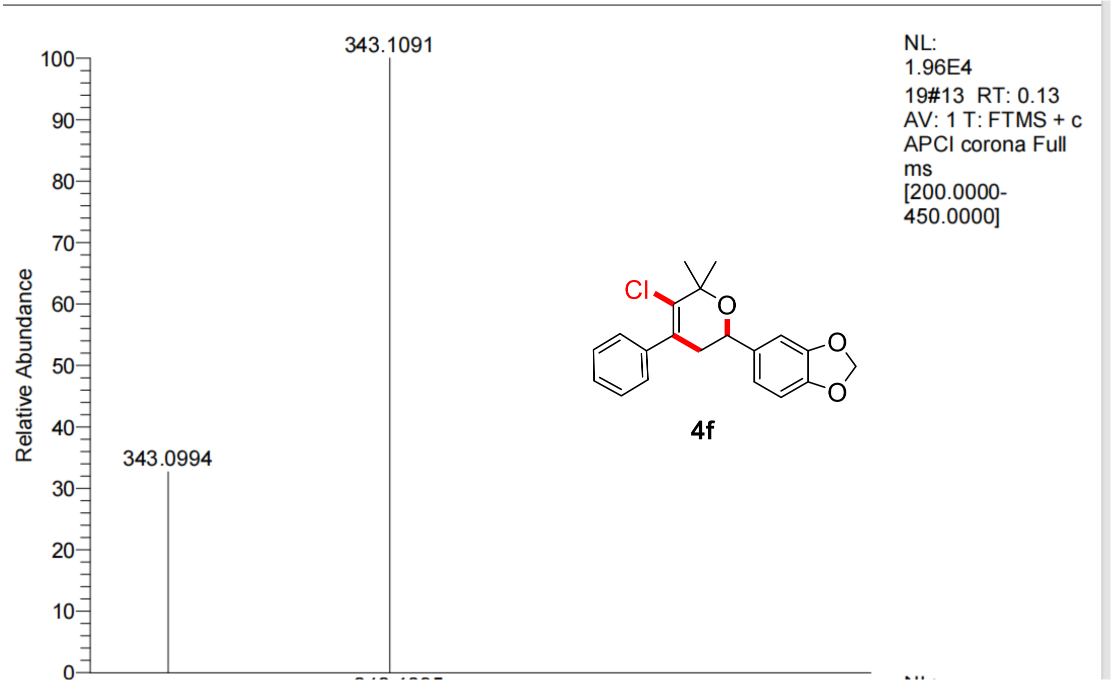

NL:
1.96E4
19#13 RT: 0.13
AV: 1 T: FTMS + c
APCI corona Full
ms
[200.0000-
450.0000]
Relative Abundance
343.1091
343.0994
Cl
O
O
O
4f

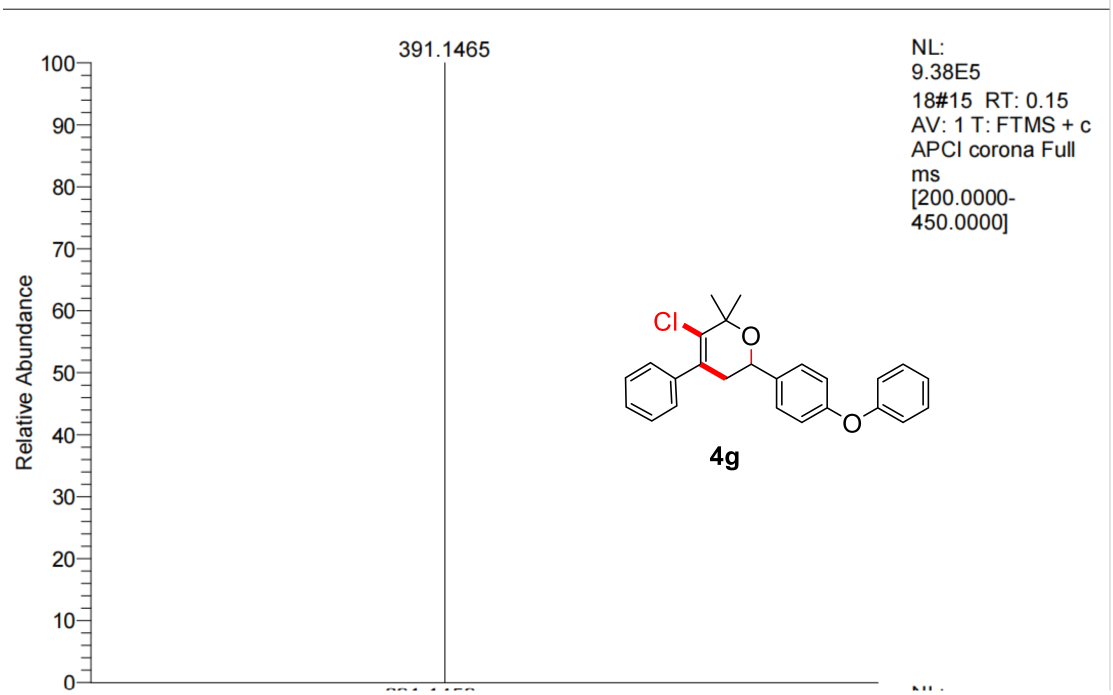
391.1465
NL:
9.38E5
18#15 RT: 0.15
AV: 1 T: FTMS + c
APCI corona Full
ms
[200.0000-
450.0000]
Relative Abundance
100
90
80
70
60
50
40
30
20
10
0
Cl
O
O
4g

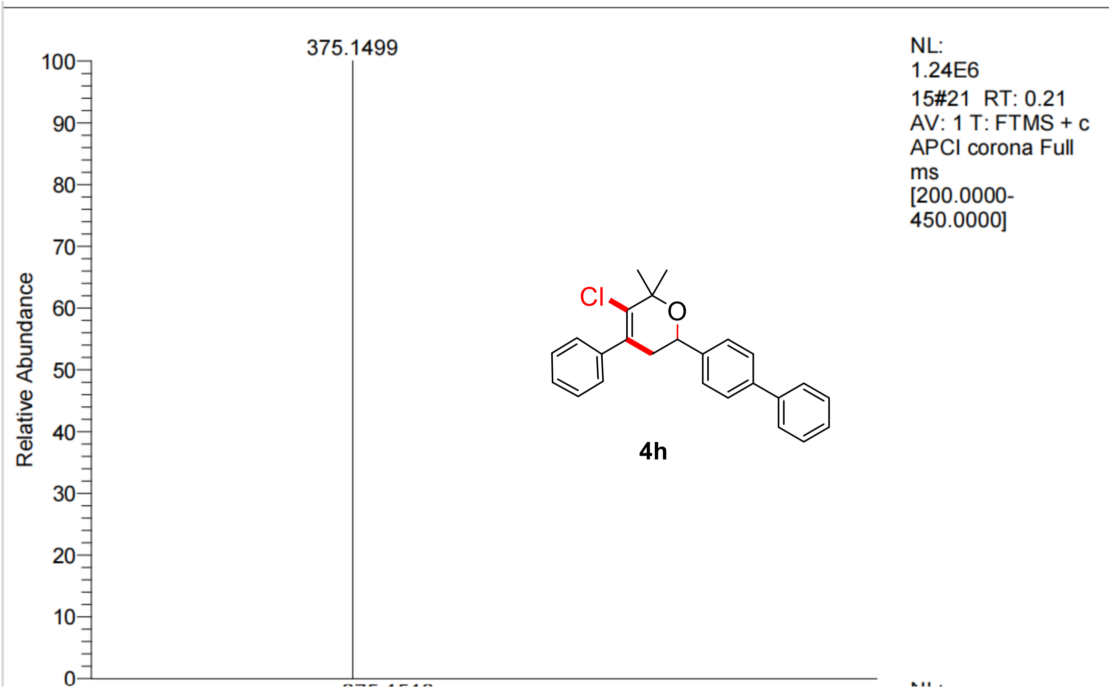
375.1499
NL:
1.24E6
15#21 RT: 0.21
AV: 1 T: FTMS + c
APCI corona Full
ms
[200.0000-
450.0000]
Relative Abundance
100
90
80
70
60
50
40
30
20
10
0
Cl
O
4h

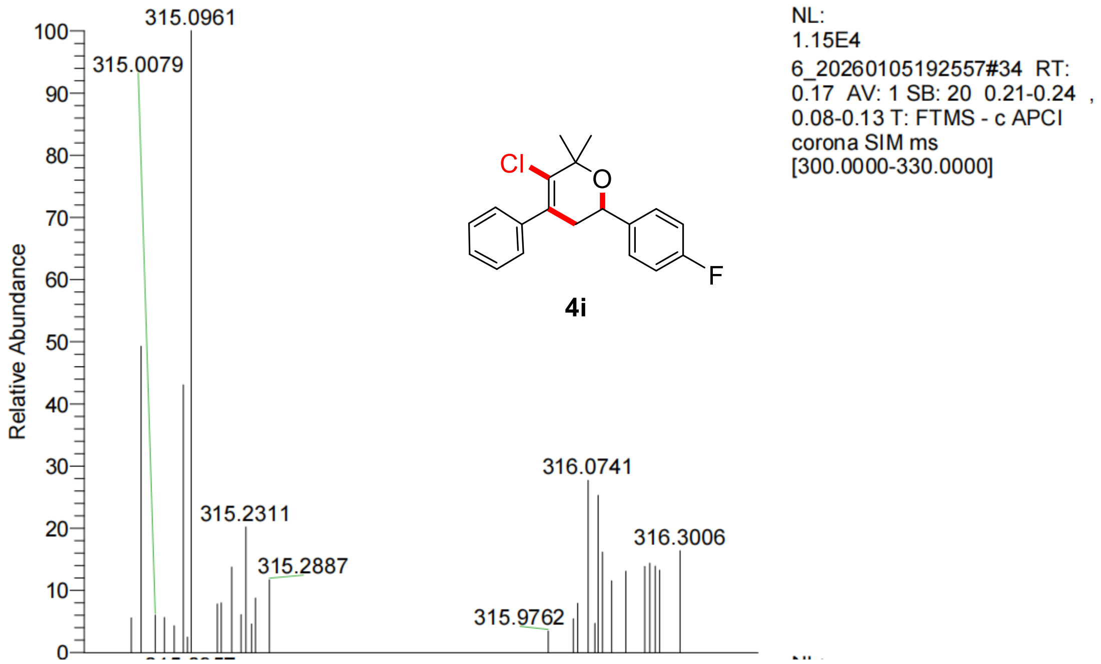
NL:
1.15E4
6_20260105192557#34 RT: 0.17 AV: 1 SB: 20 0.21-0.24 , 0.08-0.13 T: FTMS - c APCI corona SIM ms [300.0000-330.0000]
Relative Abundance
315.0961
315.0079
315.2311
315.2887
315.9762
316.0741
316.3006
Cl
O
F
4i

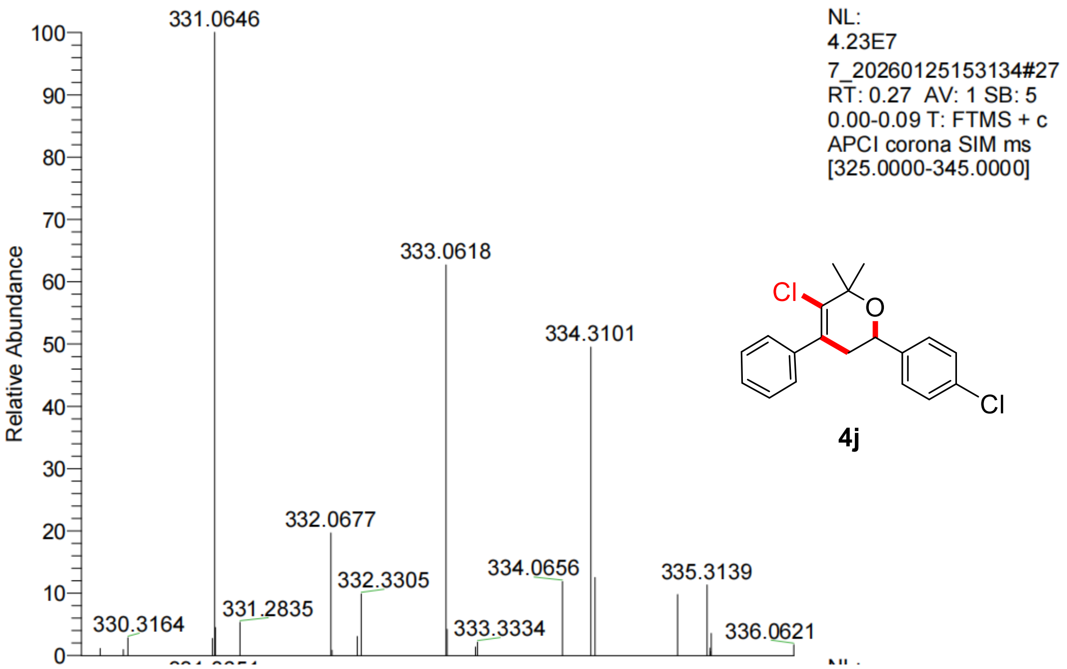
NL:
4.23E7
7_20260125153134#27 RT: 0.27 AV: 1 SB: 5 0.00-0.09 T: FTMS + c APCI corona SIM ms [325.0000-345.0000]
Relative Abundance
331.0646
333.0618
334.3101
332.0677
332.3305
334.0656
335.3139
330.3164
331.2835
333.3334
336.0621
Cl
O
Cl
4j

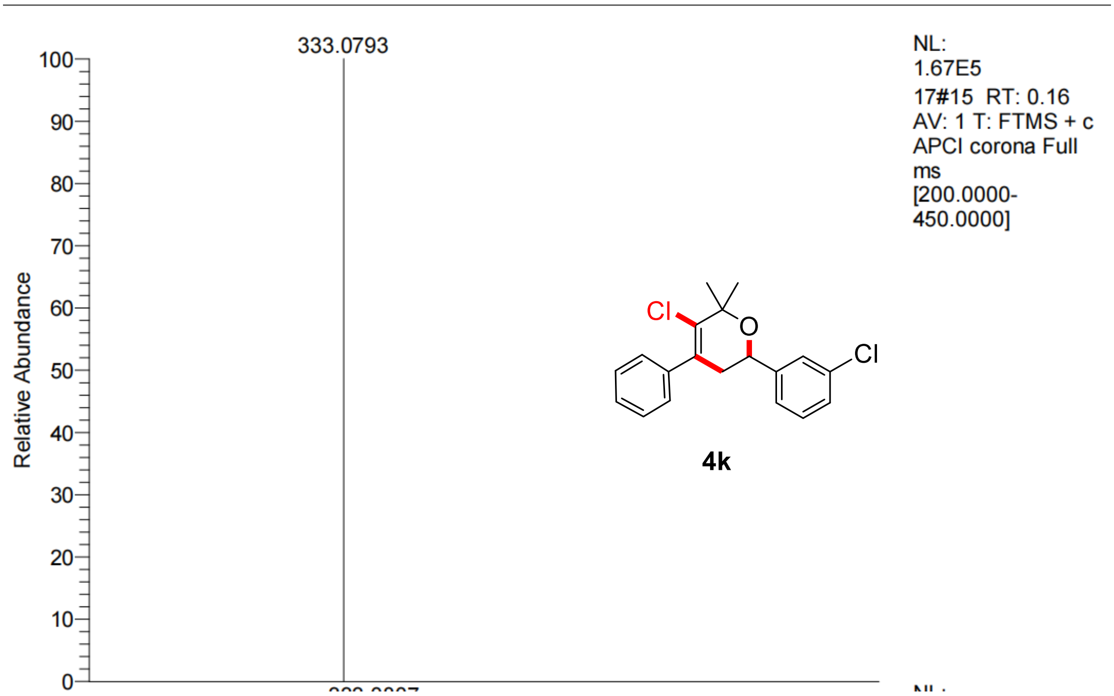
333.0793
NL:
1.67E5
17#15 RT: 0.16
AV: 1 T: FTMS + c
APCI corona Full
ms
[200.0000-
450.0000]
Relative Abundance
100
90
80
70
60
50
40
30
20
10
0
Cl
O
Cl
4k

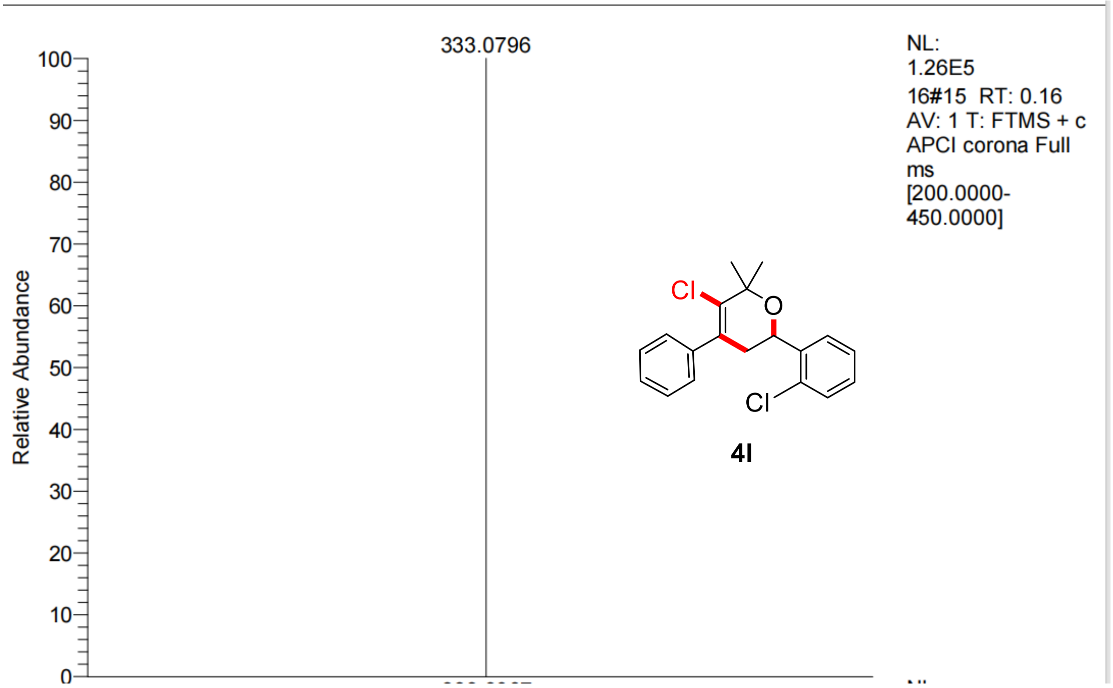
333.0796
NL:
1.26E5
16#15 RT: 0.16
AV: 1 T: FTMS + c
APCI corona Full
ms
[200.0000-
450.0000]
Relative Abundance
100
90
80
70
60
50
40
30
20
10
0
Cl
O
Cl
4l

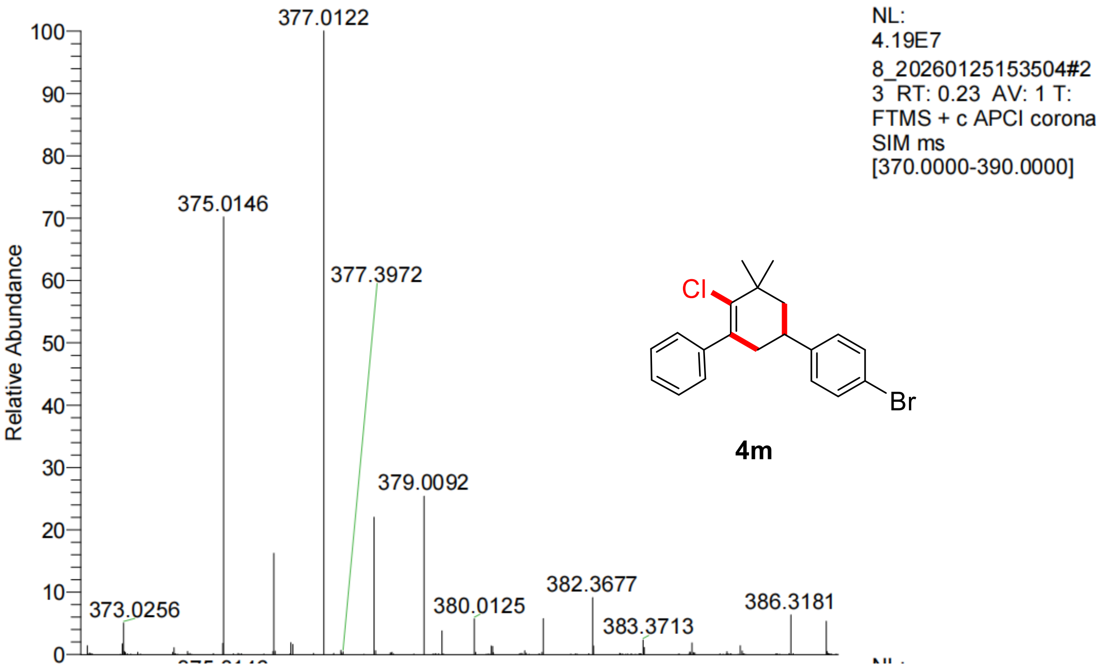
NL:
4.19E7
8_20260125153504#2
3 RT: 0.23 AV: 1 T:
FTMS + c APCI corona
SIM ms
[370.0000-390.0000]
Relative Abundance
377.0122
375.0146
377.3972
379.0092
373.0256
380.0125
382.3677
383.3713
386.3181
Cl
Br
4m

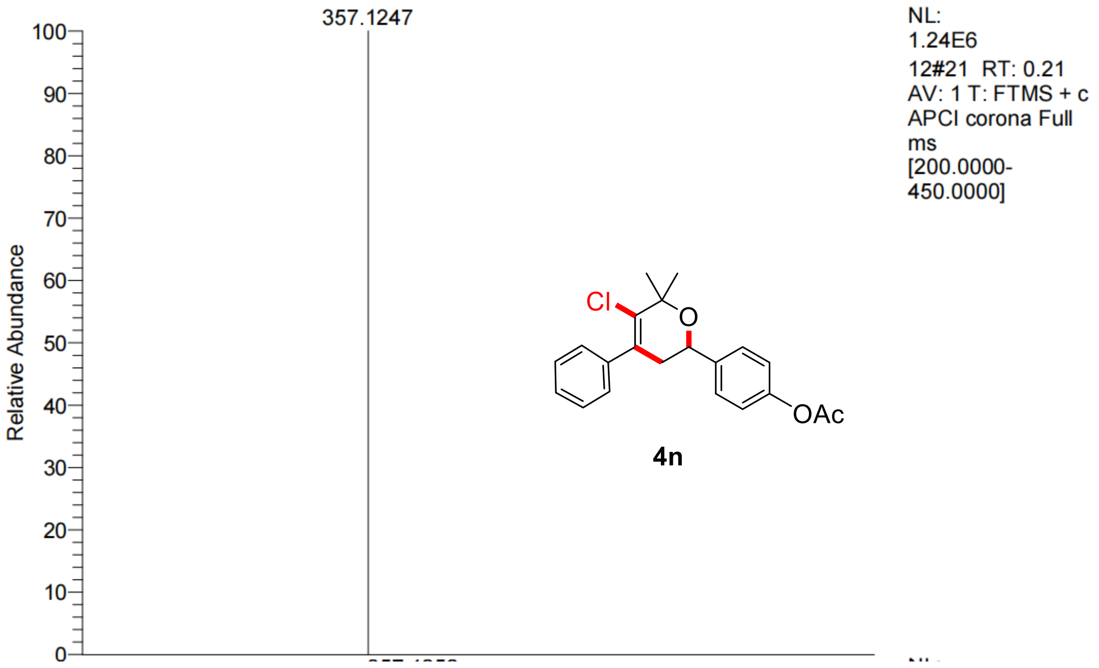
NL:
1.24E6
12#21 RT: 0.21
AV: 1 T: FTMS + c
APCI corona Full
ms
[200.0000-
450.0000]
Relative Abundance
357.1247
Cl
O
OAc
4n

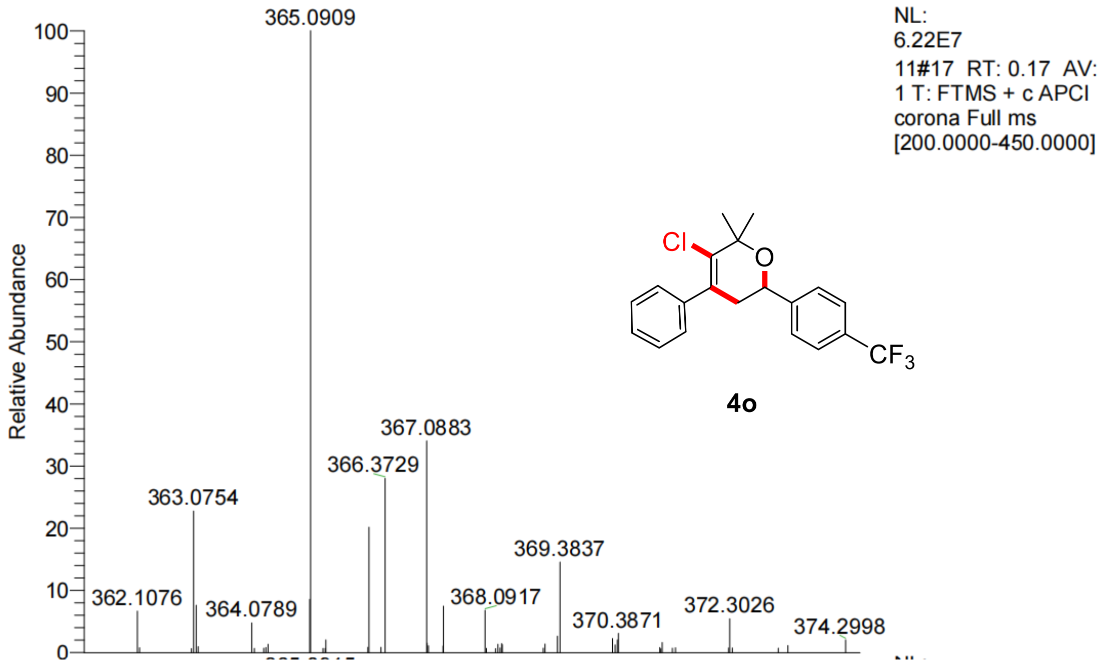
NL:
6.22E7
11#17 RT: 0.17 AV: 1 T: FTMS + c APCI corona Full ms [200.0000-450.0000]
Relative Abundance
365.0909
367.0883
366.3729
363.0754
369.3837
362.1076
364.0789
368.0917
370.3871
372.3026
374.2998
Cl
O
CF_3
4o

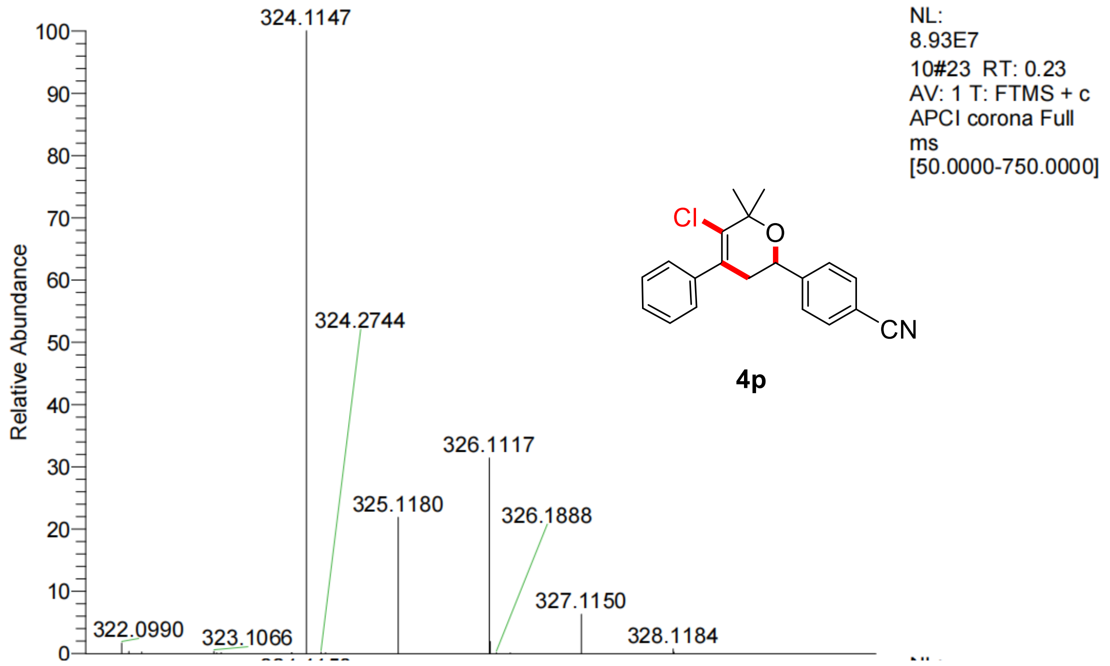
NL:
8.93E7
10#23 RT: 0.23 AV: 1 T: FTMS + c APCI corona Full ms [50.0000-750.0000]
Relative Abundance
324.1147
324.2744
326.1117
325.1180
326.1888
327.1150
322.0990
323.1066
328.1184
Cl
O
CN
4p

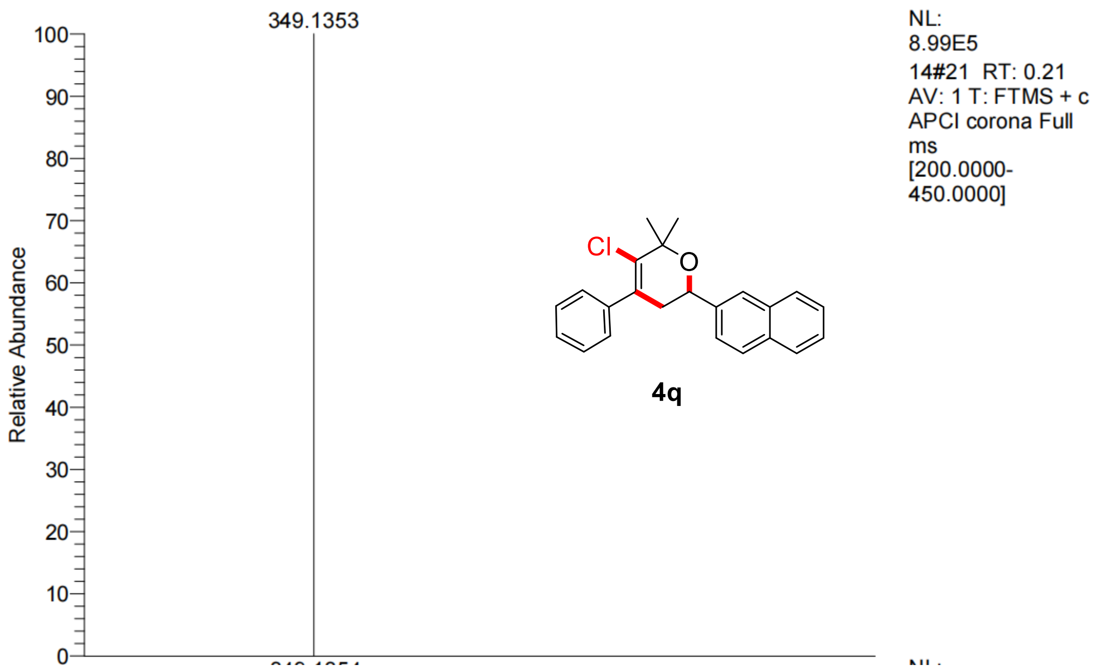

349.1353
NL:
8.99E5
14#21 RT: 0.21
AV: 1 T: FTMS + c
APCI corona Full
ms
[200.0000-
450.0000]
Relative Abundance
100
90
80
70
60
50
40
30
20
10
0
Cl
O
4q

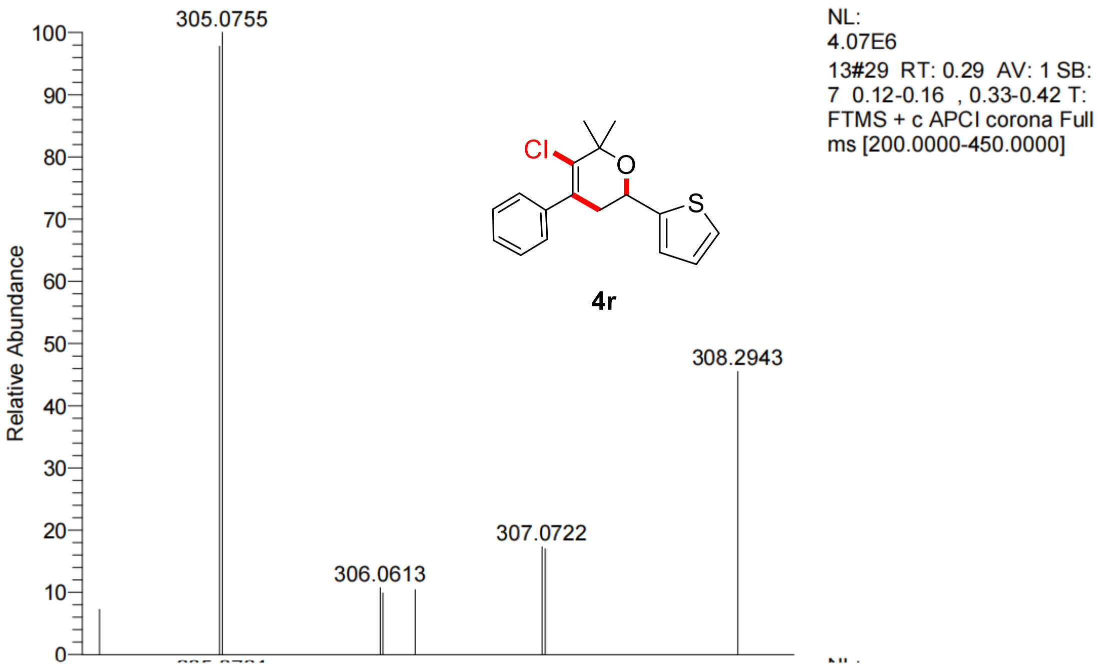

305.0755
NL:
4.07E6
13#29 RT: 0.29 AV: 1 SB:
7 0.12-0.16 , 0.33-0.42 T:
FTMS + c APCI corona Full
ms [200.0000-450.0000]
Relative Abundance
100
90
80
70
60
50
40
30
20
10
0
Cl
O
S
4r
308.2943
307.0722
306.0613

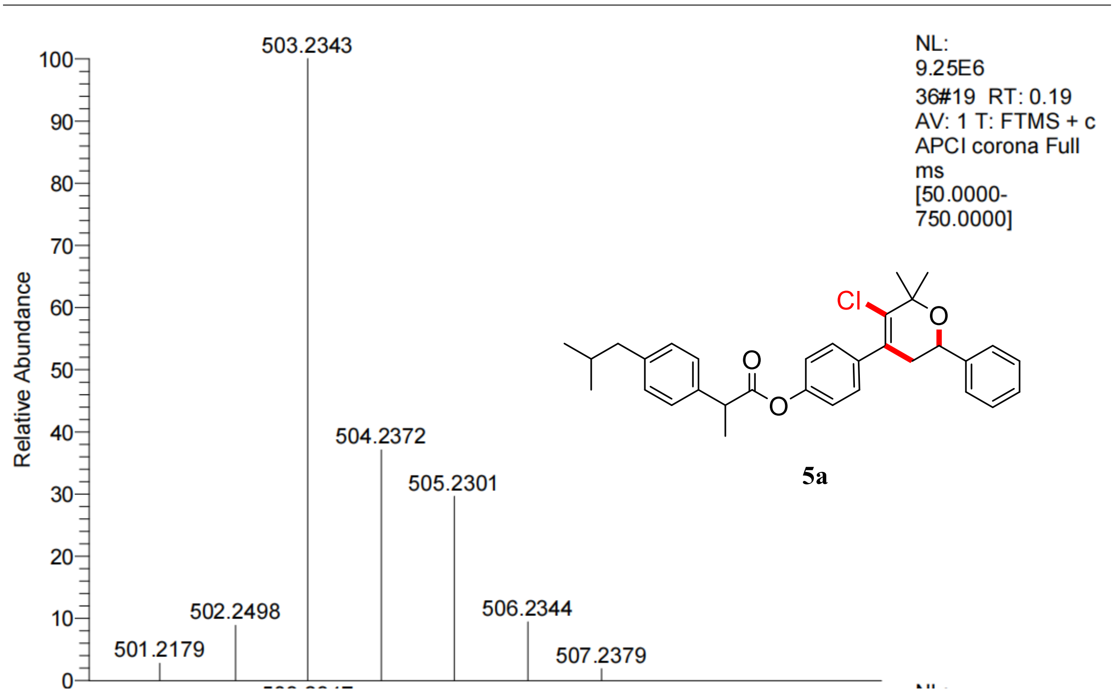
503.2343
504.2372
505.2301
506.2344
507.2379
502.2498
501.2179
Relative Abundance
100
90
80
70
60
50
40
30
20
10
0
NL:
9.25E6
36#19 RT: 0.19
AV: 1 T: FTMS + c
APCI corona Full
ms
[50.0000-
750.0000]
Cl
O
O
O
5a

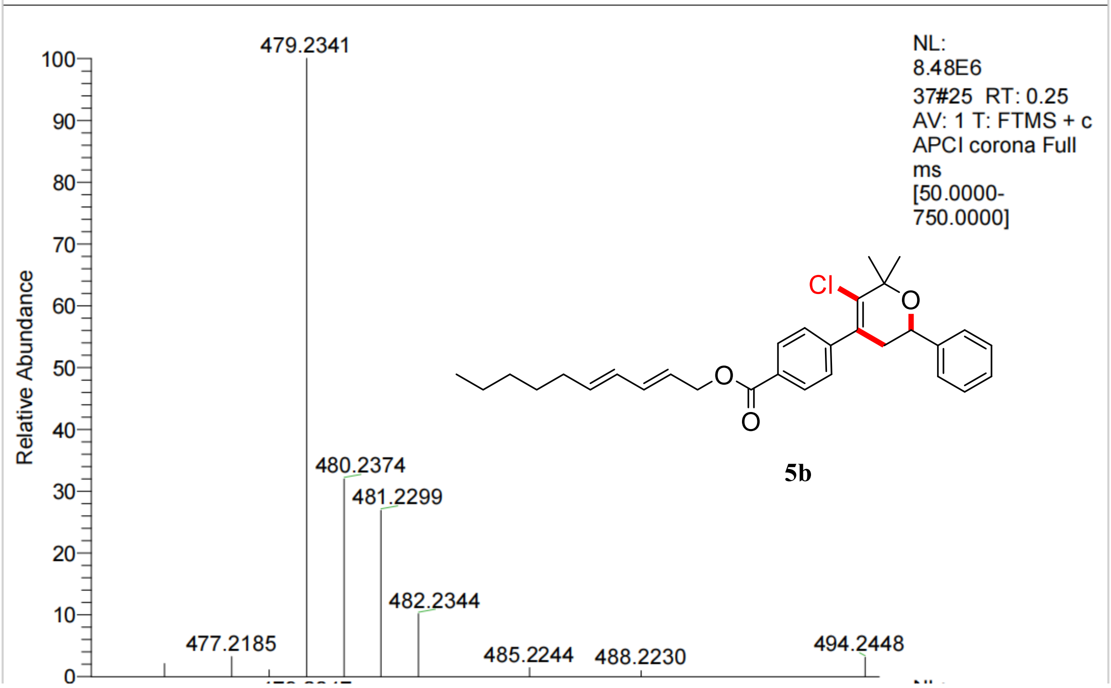
479.2341
480.2374
481.2299
482.2344
477.2185
485.2244
488.2230
494.2448
Relative Abundance
100
90
80
70
60
50
40
30
20
10
0
NL:
8.48E6
37#25 RT: 0.25
AV: 1 T: FTMS + c
APCI corona Full
ms
[50.0000-
750.0000]
Cl
O
O
O
5b

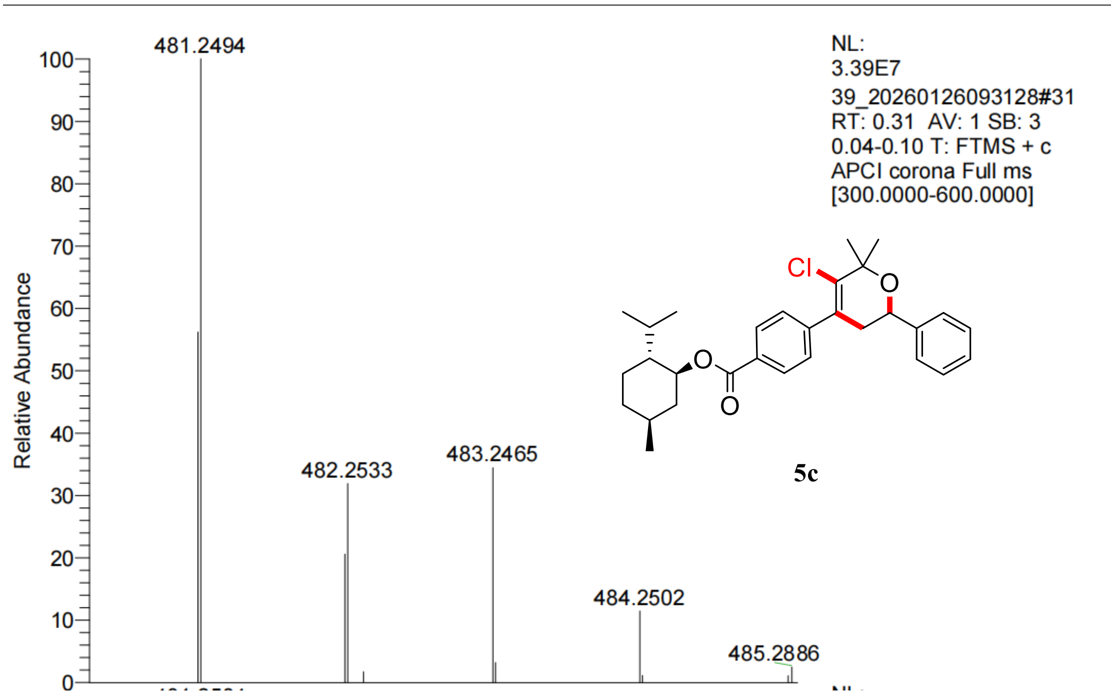
481.2494
482.2533
483.2465
484.2502
485.2886
Relative Abundance
100
90
80
70
60
50
40
30
20
10
0
NL:
3.39E7
39_20260126093128#31
RT: 0.31 AV: 1 SB: 3
0.04-0.10 T: FTMS + c
APCI corona Full ms
[300.0000-600.0000]
Cl
O
O
O
5c

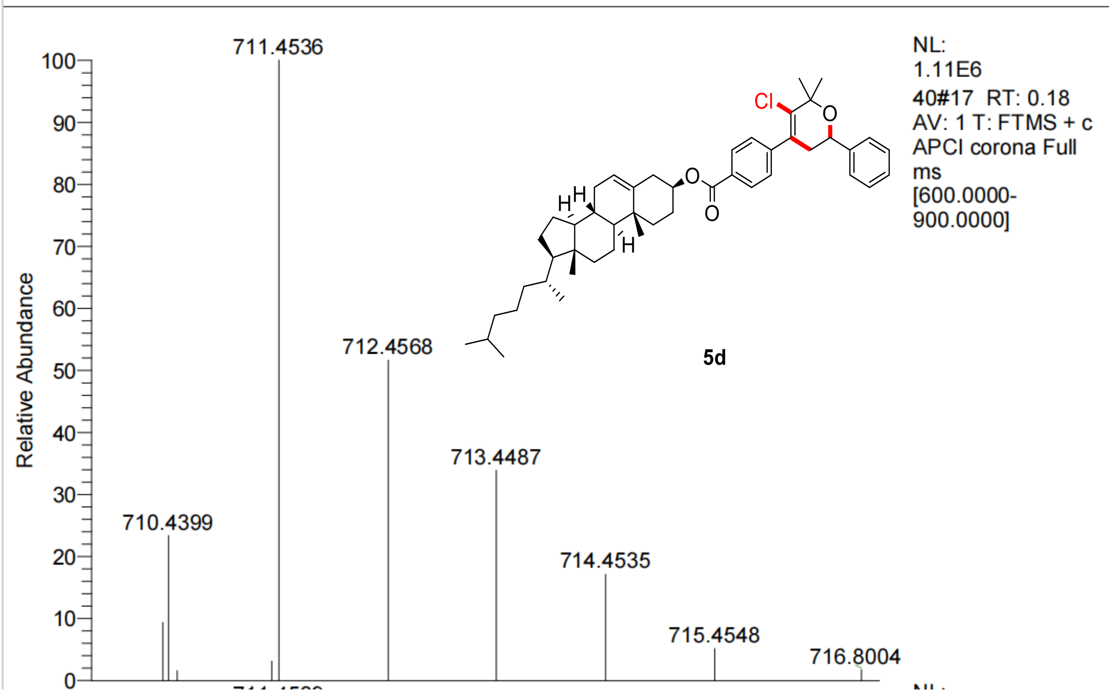
711.4536
712.4568
713.4487
710.4399
714.4535
715.4548
716.8004
Relative Abundance
100
90
80
70
60
50
40
30
20
10
0
NL:
1.11E6
40#17 RT: 0.18
AV: 1 T: FTMS + c
APCI corona Full
ms
[600.0000-
900.0000]
Cl
O
O
O
H
H
H
5d

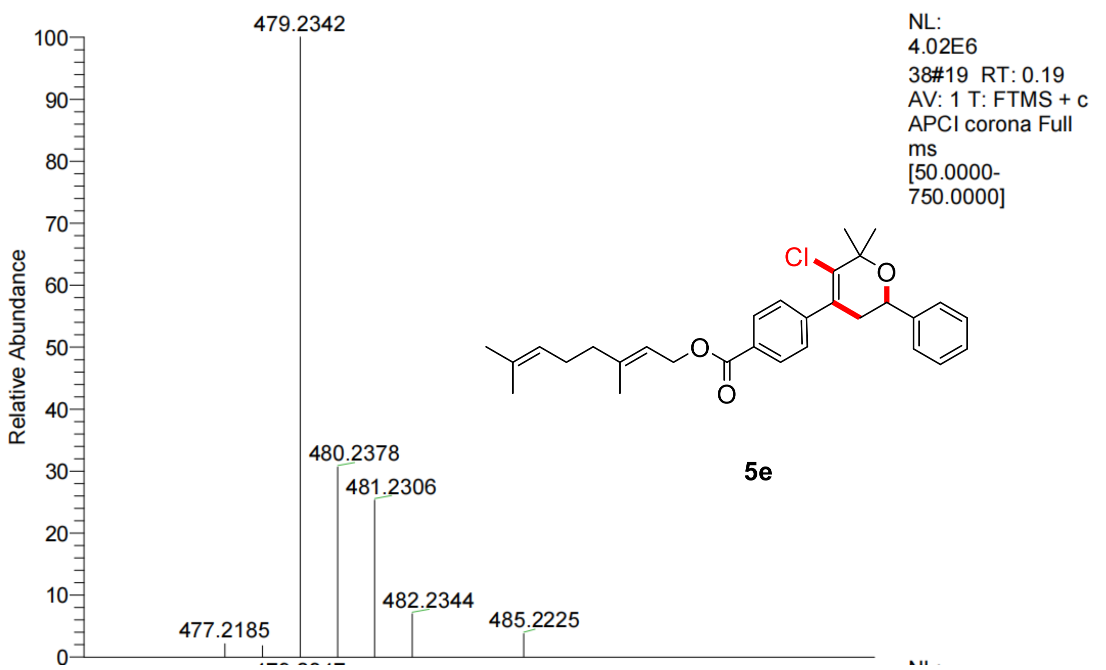
479.2342
NL:
4.02E6
38#19 RT: 0.19
AV: 1 T: FTMS + c
APCI corona Full
ms
[50.0000-
750.0000]
Relative Abundance
480.2378
481.2306
482.2344
485.2225
477.2185
Cl
O
O
O
5e

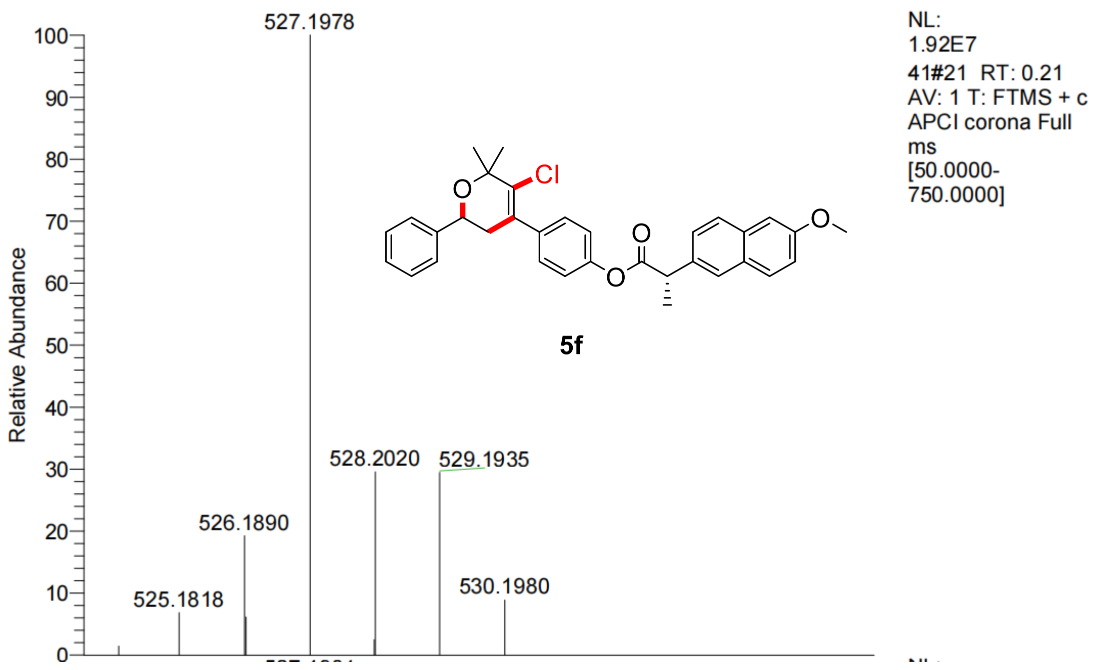
527.1978
NL:
1.92E7
41#21 RT: 0.21
AV: 1 T: FTMS + c
APCI corona Full
ms
[50.0000-
750.0000]
Relative Abundance
528.2020
529.1935
526.1890
525.1818
530.1980
Cl
O
O
O
O
5f
